# No Promoter Left Behind (NPLB): learn *de novo* promoter architectures from genome-wide transcription start sites

**DOI:** 10.1093/bioinformatics/btv645

**Published:** 2015-11-02

**Authors:** Sneha Mitra, Leelavati Narlikar

**Affiliations:** Chemical Engineering Division, CSIR-National Chemical Laboratory, Pune 411008, India

## Abstract

**Summary:** Promoters have diverse regulatory architectures and thus activate genes differently. For example, some have a TATA-box, many others do not. Even the ones with it can differ in its position relative to the transcription start site (TSS). No Promoter Left Behind (NPLB) is an efficient, organism-independent method for characterizing such diverse architectures directly from experimentally identified genome-wide TSSs, without relying on known promoter elements. As a test case, we show its application in identifying novel architectures in the fly genome.

**Availability and implementation:** Web-server at http://nplb.ncl.res.in. Standalone also at https://github.com/computationalBiology/NPLB/ (Mac OSX/Linux).

**Contact:** l.narlikar@ncl.res.in

**Supplementary information:** Supplementary data are available at *Bioinformatics* online.

## 1 Introduction

Promoters play a key role in transcription initiation by harbouring specific DNA elements, which act as transcription factor recognition sites. But how these promoter elements (PEs) contribute to the diversity in transcriptional regulation is not yet clear. While high-throughput technologies are increasingly used to produce accurate maps of transcription start sites (TSSs) (Ohler and Wassarman, 2010), the subsequent step of characterizing promoters and their functions is still done using two rather dated approaches. The first involves classifying them based on known PEs such as the INR motif or TATA-box. Unfortunately, a majority of promoters and their activities cannot be explained by the presence or absence of these few PEs. Alternatively, *de novo* motif discovery methods are used to identify overrepresented elements directly from the sequences. These can miss PEs present only in a small fraction of promoters. Since promoters have diverse mechanisms of activation, most PEs fall in this category (Juven-Gershon *etal.*, 2008). Even methods that identify *cis*-regulatory modules fail here, since although they look for motif-combinations, these are still required to be common across the full set (Van Loo and Marynen, 2009).

No Promoter Left Behind (NPLB) is a new method modelled along the lines of unsupervised learning with feature selection that partitions TSS-aligned promoter sequences into distinct promoter architectures (PAs), each characterized by its own set of PEs, all learned *de novo* (Narlikar, 2014). Since it explicitly allows for diversity, NPLB can be applied to the *full* dataset, leaving out no promoter, in contrast to the standard approach of presorting/preselecting promoters on the basis of criteria such as presence of known PEs (Chen *etal.*, 2014) or TSS peak characteristics (Ni *etal.*, 2010). In this new parallel software, the number of PAs and PEs are determined automatically using a mix of Bayesian modelling and cross validation.

## 2 Methods

### 2.1 Overview of NPLB

Each promoter is characterized by one PA out of a finite set of PAs. Each PA is characterized by categorical distributions over nucleotides {A,C,G,T} at specific positions relative to the TSS. These positions and their distributions are expected to be unique to that PA. All other positions follow a background categorical distribution, common for all PAs. Parameters of models with various numbers of PAs are learned using Gibbs sampling and the best model is decided using cross validation. Key advantages of NPLB are that it Written in C and Python, NPLB requires a prior installation of gnuplot 4.6+. Weblogo 3.3 (Crooks *etal.*, 2004), and is modified to generate sequence logos.

1. identifies novel and possibly diverse architectures and elements, with the only input being the set of promoters,
2. is an organism and a cell-type independent,
3. can be applied to the full set, directly,
4. employs a likelihood-based approach, thus can be used to make new predictions of promoters, as well as classify between architectures,
5. uses multiprocessing, making it fast: takes about 2 h for bacteria and 10 h for fly on an Intel i7-3770 K desktop. (Supplementary Fig. S1 shows how runtime scales with number of promoters.)

### 2.2 NPLB input

NPLB can learn new PAs (promoterLearn) or categorize new promoters based on an input PA-model (promoterClassify). Both require a fasta file of promoters, aligned according to the TSS. A typical eukaryotic file would contain DNA sequences ∼50 bp up- and downstream of the TSS. promoterClassify also needs a previously learned model. Various other default settings such as number of PAs to be explored and the number of sampling iterations can be overridden by the user. This is especially useful when the user wants to choose between a quick, approximate solution and a slow, but more accurate characterization. A tab-separated text file with one line per promoter, containing additional characteristics of each TSS such as UTR length, TSS spread, etc. is an optional input. In such a situation, NPLB creates plots that can give insights into functional differences between PAs.

### 2.3 NPLB output

A successful run of promoterLearn produces the following outputs: A successful run of promoterClassify produces all the aforementioned files except *CVLikelihoods.txt*, *settings.txt* and the likelihood plots.

**Fig. 1.**
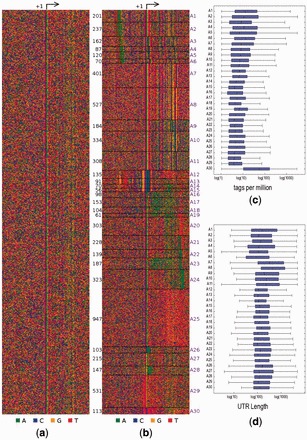
(**a**) Original set of promoter sequences. (**b**) 30 PAs learned by NPLB, ordered here based on presence of known PEs. (**c**) Tags per million at TSSs in each PA. (**d**) Length of 5′ UTRs in each PA
- If a characteristic file is supplied, box-plots (Fig. 1c and d) or piecharts are created for real or categorical characteristics, respectively.
- The model itself is saved in a binary file *bestmodel.p* and can be used by NPLB to classify a new promoter.
- The best model is determined by cross validation. Likelihoods of all models are recorded in *CVLikelihoods.txt.* The verbose option leads to likelihoods of all sampling iterations to be plotted in separate *png* files.
- The parameters of the execution are saved in *settings.txt.*

- PAs in two visual formats: image (*PAimage.png*; Fig. 1b) and logos (*PAlogo.html*; Supplementary Fig. S1). The input is stored as *rawImage.png* (Fig. 1a) for reference. An -eps option produces eps figures. More details about the PEs and PAs are reported in *modelOut.txt* and *architectureDetails.txt.*

## 3 Case study: *Drosophila*

promoterLearn was applied to 90-bp neighbourhoods centred on 6635 TSSs (Fig. 1a) reported in adult *Drosophila melanogaster* carcasses (Chen *etal.*, 2014). In the original study, four types of promoters were identified, based on known fly PEs (Ohler and Wassarman, 2010): TATA-box, INR, DPE, Dmv4 and Dmv5. These four types accounted for 2112 of the 6635 promoters (Supplementary Fig. S3a). Here, 12 PAs were identified (Supplementary Fig. S4a); promoterLearn was run again on each of them. Eight PAs were split further into a total of 23 PAs (Supplementary Fig. S4b), three of which were split to get a final set of 30 PAs (Fig. 1b).

A1–A6 contain the TATA-box, but differ in its distance from the TSS. Interestingly, the INR motif TCAGTY varies slightly with the TATA-box position in A3–A6. Standard analyses miss such variations, either because they rely on known PEs or look for elements overrepresented in the full set. For instance, in the sequences left out in the original study, NPLB finds PAs characterized by known as well as novel PEs (Supplementary Fig. S3b).

The characteristic file with the number of tags at each TSS and 5′ UTR length was used to construct two box-plots (Fig. 1c and d). A30 contains the ribosomal TCT motif (Parry *etal.*, 2010) in place of the INR, which explains the significantly higher number of tags at those promoters (*P* < 10^−^^21^). This PA was missed in the original analysis possibly since it contains <2% of all promoters. Interestingly, A7–A11, which contain variants of the DPE, but no obvious upstream element, create transcripts with longer 5′ UTRs than other PAs (*P* < 10^−^^62^). This has not been noted before. A more detailed description of the PAs is available in the Supplementary methods. PAs can be further analysed for function through conservation analysis (Karolchik *etal.*, 2014; Supplementary Fig. S5) and GO term enrichment studies (Huang *etal.*, 2007; Supplementary Table S1).

## 4 Conclusion

Data from new and advanced high-throughput technologies are increasingly making it clear that cells employ diverse mechanisms for transcriptional regulation. NPLB seeks to fulfil the need for an efficient and unbiased method that can identify these mechanisms directly from such data. Although NPLB has been designed for TSS maps, it can be applied to any DNA sequences aligned on the basis of a common genomic event such as splicing, eRNA synthesis or protein–DNA binding and expected to have distinct sequence architectures in the immediate neighbourhood.

## Supplementary Material

Supplementary Data
