# Supplementary material for: No Promoter Left Behind (NPLB): learn de novo promoter architectures from genome-wide transcription start sites
Source: Bioinformatics. 2015 Nov 2;32(5):779–81. doi: 10.1093/bioinformatics/btv645 (PMC4795619; doi:10.1093/bioinformatics/btv645)
Supplement: Supplementary Data [file btv645_supplementary_data.zip › TableS1.pdf]

Table S1: GO Term Analysis of all architectures

Table 1: Architecture 1

Category	Term	Count	%	PValue	List Total	Pop Hits	Pop Total	Fold Enrichment	Bonferroni	Benjamini	FDR
GOTERM_BP_FAT	GO:0006508 proteolysis	24	2.628697	3.922928e-05	103	731	7937	2.529956	0.024948	0.024948	0.058484
GOTERM_MF_FAT	GO:0008233 peptidase activity	25	2.738226	4.120117e-05	123	648	7918	2.483564	0.011511	0.011511	0.054494
GOTERM_MF_FAT	GO:0070011 peptidase activity, acting on L-amino acid peptides	24	2.628697	4.890471e-05	123	612	7918	2.524470	0.013649	0.006848	0.064680
GOTERM_BP_FAT	GO:0009072 aromatic amino acid family metabolic process	5	0.547645	8.591844e-05	103	19	7937	20.278487	0.053831	0.027288	0.128047
INTERPRO	IPR015421:Pyridoxal phosphate-dependent transferase, major region, subdomain 1	6	0.657174	1.029866e-04	149	33	10196	12.441733	0.029026	0.029026	0.136532
SMART	SM00020:Tryp_SPc	12	1.314348	1.927854e-04	61	248	4824	3.826547	0.008830	0.008830	0.182069
GOTERM_MF_FAT	GO:0070279 vitamin B6 binding	6	0.657174	3.028213e-04	123	39	7918	9.903690	0.081585	0.027970	0.399880
GOTERM_MF_FAT	GO:0030170 pyridoxal phosphate binding	6	0.657174	3.028213e-04	123	39	7918	9.903690	0.081585	0.027970	0.399880
INTERPRO	IPR001314:Peptidase S1A, chymotrypsin	11	1.204819	9.550531e-04	149	210	10196	3.584404	0.239117	0.127714	1.259541
INTERPRO	IPR001254:Peptidase S1 and S6, chymotrypsin/Hap	12	1.314348	9.596201e-04	149	248	10196	3.311106	0.240111	0.087464	1.265529
GOTERM_MF_FAT	GO:0008237 metallopeptidase activity	10	1.095290	0.001103	123	168	7918	3.831785	0.266641	0.074601	1.449455
GOTERM_MF_FAT	GO:0004175 endopeptidase activity	17	1.861993	0.001657	123	457	7918	2.394656	0.372485	0.088987	2.170005
GOTERM_BP_FAT	GO:0055114 oxidation reduction	18	1.971522	0.002175	103	620	7937	2.237175	0.753999	0.373417	3.195234
KEGG_PATHWAY	dme00350:Tyrosine metabolism	5	0.547645	0.003132	38	35	2054	7.721805	0.150516	0.150516	3.001856
GOTERM_MF_FAT	GO:0004252 serine-type endopeptidase activity	12	1.314348	0.003833	123	280	7918	2.758885	0.660146	0.164624	4.954186
SP_PIR_KEYWORDS	hydrolase	33	3.614458	0.004507	177	1470	12980	1.646259	0.423654	0.423654	5.067553
SP_PIR_KEYWORDS	pyridoxal phosphate	4	0.438116	0.005066	177	26	12980	11.282051	0.461854	0.266416	5.679979
INTERPRO	IPR011701:Major facilitator superfamily MFS-1	7	0.766703	0.005489	149	111	10196	4.315376	0.792814	0.325332	7.041192
SP_PIR_KEYWORDS	lyase	7	0.766703	0.006316	177	122	12980	4.207650	0.538385	0.227154	7.035611
GOTERM_BP_FAT	GO:0045087 innate immune response	6	0.657174	0.006461	103	92	7937	5.025538	0.984612	0.647797	9.213175
KEGG_PATHWAY	dme00360:Phenylalanine metabolism	4	0.438116	0.006548	38	22	2054	9.827751	0.289392	0.157024	6.183624
INTERPRO	IPR018114:Peptidase S1/S6, chymotrypsin/Hap, active site	9	0.985761	0.006636	149	191	10196	3.224428	0.851042	0.316698	8.453011
GOTERM_MF_FAT	GO:0008236 serine-type peptidase activity	12	1.314348	0.007758	123	308	7918	2.508077	0.887926	0.268499	9.790920
GOTERM_MF_FAT	GO:0017171 serine hydrolase activity	12	1.314348	0.008128	123	310	7918	2.491896	0.899071	0.249239	10.234699
GOTERM_BP_FAT	GO:0006582 melanin metabolic process	3	0.328587	0.008340	103	11	7937	21.015887	0.995455	0.659971	11.740849
INTERPRO	IPR005804:Fatty acid desaturase, type 1	3	0.328587	0.008725	149	10	10196	20.528859	0.918435	0.341457	10.974986
GOTERM_BP_FAT	GO:0046394 carboxylic acid biosynthetic process	5	0.547645	0.009058	103	64	7937	6.020176	0.997149	0.623438	12.688939
GOTERM_BP_FAT	GO:0016053 organic acid biosynthetic process	5	0.547645	0.009058	103	64	7937	6.020176	0.997149	0.623438	12.688939
GOTERM_MF_FAT	GO:0016717 oxidoreductase activity, acting on paired donors, with oxidation of a pair of donors resulting in the reduction of molecular oxygen to two molecules of water	3	0.328587	0.009773	123	10	7918	19.312195	0.936695	0.264087	12.184503
GOTERM_CC_FAT	GO:0043679 nerve terminal	3	0.328587	0.011944	55	15	4786	17.403636	0.730094	0.730094	12.674221
GOTERM_CC_FAT	GO:0033267 axon part	3	0.328587	0.011944	55	15	4786	17.403636	0.730094	0.730094	12.674221
INTERPRO	IPR015422:Pyridoxal phosphate-dependent transferase, major region, subdomain 2	3	0.328587	0.012556	149	12	10196	17.107383	0.973050	0.403248	15.432368
GOTERM_MF_FAT	GO:0019842 vitamin binding	6	0.657174	0.018124	123	99	7918	3.901454	0.994139	0.401867	21.491755
SMART	SM00680:CLIP	3	0.328587	0.018352	61	17	4824	13.955641	0.573458	0.346898	16.060424
GOTERM_BP_FAT	GO:0044270 nitrogen compound catabolic process	3	0.328587	0.019616	103	17	7937	13.598515	0.999997	0.838400	25.578820
SP_PIR_KEYWORDS	oxidoreductase	15	1.642935	0.020492	177	561	12980	1.960784	0.920021	0.468206	21.210410
GOTERM_CC_FAT	GO:0005811 lipid particle	8	0.876232	0.020703	55	249	4786	2.795765	0.897744	0.680225	21.018855
GOTERM_MF_FAT	GO:0020037 heme binding	7	0.766703	0.020836	123	140	7918	3.218699	0.997306	0.416015	24.312748
GOTERM_MF_FAT	GO:0046906 tetrapyrrole binding	7	0.766703	0.020836	123	140	7918	3.218699	0.997306	0.416015	24.312748
GOTERM_MF_FAT	GO:0008238 exopeptidase activity	6	0.657174	0.023605	123	106	7918	3.643810	0.998784	0.428438	27.096422
GOTERM_BP_FAT	GO:0006952 defense response	7	0.766703	0.023701	103	173	7937	3.117964	1.000000	0.854981	30.070895
GOTERM_MF_FAT	GO:0048037 cofactor binding	8	0.876232	0.023928	123	185	7918	2.783740	0.998892	0.407561	27.414940
GOTERM_MF_FAT	GO:0008483 transaminase activity	3	0.328587	0.024540	123	16	7918	12.070122	0.999071	0.392678	28.014388
INTERPRO	IPR001199:Cytochrome b5	3	0.328587	0.024678	149	17	10196	12.075799	0.999212	0.590695	28.213323
INTERPRO	IPR006604:Disulphide knot CLIP	3	0.328587	0.024678	149	17	10196	12.075799	0.999212	0.590695	28.213323
KEGG_PATHWAY	dme00980:Metabolism of xenobiotics by cytochrome P450	5	0.547645	0.026186	38	64	2054	4.222862	0.748369	0.368673	22.724995
INTERPRO	IPR005511:Senescence marker protein-30 (SMP-30)	2	0.219058	0.028822	149	2	10196	68.429530	0.999767	0.605189	32.155721
INTERPRO	IPR012171:Fatty acid/sphingolipid desaturase	2	0.219058	0.028822	149	2	10196	68.429530	0.999767	0.605189	32.155721

INTERPRO	IPR013658:SMP-30/Gluconolaconase/LRE-like region	2	0.219058	0.028822	149	2	10196	68.429530	0.999767	0.605189	32.155721
INTERPRO	IPR001176:1-aminocyclopropane-1-carboxylate synthase	2	0.219058	0.028822	149	2	10196	68.429530	0.999767	0.605189	32.155721
KEGG_PATHWAY	dme00982:Drug metabolism	5	0.547645	0.028952	38	66	2054	4.094896	0.782977	0.317462	24.831944
INTERPRO	IPR001930:Peptidase M1, membrane alanine aminopeptidase	3	0.328587	0.033516	149	20	10196	10.264430	0.999942	0.622802	36.379207
GOTERM_CC_FAT	GO:0030424 axon	3	0.328587	0.034164	55	26	4786	10.040559	0.977381	0.717193	32.434910
GOTERM_BP_FAT	GO:0009611 response to wounding	3	0.328587	0.034721	103	23	7937	10.051076	1.000000	0.920233	40.960937
GOTERM_CC_FAT	GO:0042995 cell projection	5	0.547645	0.035668	55	111	4786	3.919738	0.980915	0.628319	33.612432
SP_PIR_KEYWORDS	Serine protease	6	0.657174	0.036707	177	135	12980	3.259259	0.989564	0.598477	34.986738
GOTERM_BP_FAT	GO:0042381 hemolymph coagulation	2	0.219058	0.038065	103	3	7937	51.372168	1.000000	0.917854	43.938468
GOTERM_MF_FAT	GO:0016769 transferase activity, transferring nitrogenous groups	3	0.328587	0.040858	123	21	7918	9.196283	0.999992	0.542277	42.414265
PIR_SUPERFAMILY	PIRSF015921: FA_sphinglp_des	2	0.219058	0.041932	56	2	2596	46.357143	0.905202	0.905202	34.382841
PIR_SUPERFAMILY	PIRSF004716:senescence marker protein-30	2	0.219058	0.041932	56	2	2596	46.357143	0.905202	0.905202	34.382841
PIR_SUPERFAMILY	PIRSF015921:fatty acid desaturase/sphingolipid desaturase	2	0.219058	0.041932	56	2	2596	46.357143	0.905202	0.905202	34.382841
COG_ONTOLOGY	Defense mechanisms	3	0.328587	0.042242	19	23	1237	8.491991	0.350534	0.350534	22.830454
GOTERM_BP_FAT	GO:0006979 response to oxidative stress	4	0.438116	0.043376	103	61	7937	5.053000	1.000000	0.925444	48.381305
GOTERM_MF_FAT	GO:0016847 1-aminocyclopropane-1-carboxylate synthase activity	2	0.219058	0.045521	123	3	7918	42.915989	0.999998	0.558787	46.009507
GOTERM_MF_FAT	GO:0016744 transferase activity, transferring aldehyde or ketonic groups	2	0.219058	0.045521	123	3	7918	42.915989	0.999998	0.558787	46.009507
INTERPRO	IPR014782:Peptidase M1, membrane alanine aminopeptidase, N-terminal	3	0.328587	0.046894	149	24	10196	8.553691	0.999999	0.713139	47.119508
GOTERM_MF_FAT	GO:0005506 iron ion binding	9	0.985761	0.047168	123	260	7918	2.228330	0.999999	0.550063	47.229227
GOTERM_BP_FAT	GO:0050817 coagulation	2	0.219058	0.050432	103	4	7937	38.529126	1.000000	0.937783	53.776230
GOTERM_BP_FAT	GO:0007599 hemostasis	2	0.219058	0.050432	103	4	7937	38.529126	1.000000	0.937783	53.776230
GOTERM_BP_FAT	GO:0042440 pigment metabolic process	4	0.438116	0.052705	103	66	7937	4.670197	1.000000	0.931590	55.399102
GOTERM_CC_FAT	GO:0009288 flagellin-based flagellum	2	0.219058	0.055178	55	5	4786	34.807273	0.997943	0.709847	47.281193
GOTERM_MF_FAT	GO:0016831 carboxy-lyase activity	3	0.328587	0.056102	123	25	7918	7.724878	1.000000	0.593976	53.412715
GOTERM_CC_FAT	GO:0005576 extracellular region	11	1.204819	0.057328	55	512	4786	1.869531	0.998396	0.657848	48.618289
GOTERM_BP_FAT	GO:0006633 fatty acid biosynthetic process	3	0.328587	0.059748	103	31	7937	7.457250	1.000000	0.941220	60.096666
GOTERM_BP_FAT	GO:0035006 melanization defense response	2	0.219058	0.062641	103	5	7937	30.823301	1.000000	0.937794	61.888580
KEGG_PATHWAY	dme00030:Pentose phosphate pathway	3	0.328587	0.063023	38	23	2054	7.050343	0.966123	0.491863	46.871378
GOTERM_BP_FAT	GO:0019748 secondary metabolic process	4	0.438116	0.065064	103	72	7937	4.281014	1.000000	0.933323	63.331441
GOTERM_BP_FAT	GO:0006955 immune response	6	0.657174	0.066159	103	169	7937	2.735796	1.000000	0.925205	63.966652
SP_PIR_KEYWORDS	Protease	8	0.876232	0.068238	177	264	12980	2.222222	0.999820	0.762390	55.681379
GOTERM_BP_FAT	GO:0042417 dopamine metabolic process	2	0.219058	0.074695	103	6	7937	25.686084	1.000000	0.937805	68.577969
GOTERM_BP_FAT	GO:0001539 ciliary or flagellar motility	2	0.219058	0.074695	103	6	7937	25.686084	1.000000	0.937805	68.577969
GOTERM_BP_FAT	GO:0044271 nitrogen compound biosynthetic process	7	0.766703	0.079035	103	233	7937	2.315055	1.000000	0.938619	70.705452
UP_SEQ_FEATURE	signal peptide	9	0.985761	0.081998	34	419	2975	1.879475	0.999995	0.999995	63.650345
INTERPRO	IPR009081:Acyl carrier protein-like	2	0.219058	0.084013	149	6	10196	22.809843	1.000000	0.876491	68.779505
GOTERM_CC_FAT	GO:0043005 neuron projection	3	0.328587	0.087535	55	44	4786	5.933058	0.999954	0.759836	64.414807
SMART	SM00192:LDLa	3	0.328587	0.087838	61	40	4824	5.931148	0.985435	0.755787	58.062037
KEGG_PATHWAY	dme00250:Alanine, aspartate and glutamate metabolism	3	0.328587	0.089061	38	28	2054	5.791353	0.992176	0.554440	59.597186
SP_PIR_KEYWORDS	purine biosynthesis	2	0.219058	0.091161	177	7	12980	20.952381	0.999991	0.810988	66.731376
UP_SEQ_FEATURE	zinc finger region:C2H2-type 3	3	0.328587	0.091276	34	46	2975	5.706522	0.999999	0.998881	67.765657
KEGG_PATHWAY	dme00983:Drug metabolism	4	0.438116	0.091310	38	60	2054	3.603509	0.993120	0.508994	60.556103
SMART	SM00737:ML	2	0.219058	0.095343	61	8	4824	19.770492	0.990040	0.684089	61.212333
GOTERM_BP_FAT	GO:0006570 tyrosine metabolic process	2	0.219058	0.098344	103	8	7937	19.264563	1.000000	0.964329	78.641999
GOTERM_BP_FAT	GO:0043455 regulation of secondary metabolic process	2	0.219058	0.098344	103	8	7937	19.264563	1.000000	0.964329	78.641999

Table 2: Architecture 2

Category	Term	Count	%	PValue	List Total	Pop Hits	Pop Total	Fold Enrichment	Bonferroni	Benjamini	FDR
GOTERM_BP_FAT	GO:0006508 proteolysis	26	5.168986	1.336068e-05	110	731	7937	2.566372	0.008488	0.008488	0.019897
SP_PIR_KEYWORDS	hydrolase	44	8.747515	1.215305e-04	215	1470	12980	1.807056	0.015676	0.015676	0.141437
GOTERM_MF_FAT	GO:0008233 peptidase activity	26	5.168986	1.331205e-04	140	648	7918	2.269268	0.034536	0.034536	0.174278
GOTERM_MF_FAT	GO:0070011 peptidase activity, acting on L-amino acid peptides	24	4.771372	3.749853e-04	140	612	7918	2.217927	0.094271	0.048302	0.490202
SP_PIR_KEYWORDS	heme	9	1.789264	4.939774e-04	215	111	12980	4.895035	0.062213	0.031606	0.573751
GOTERM_CC_FAT	GO:0005624 membrane fraction	8	1.590457	6.842823e-04	69	106	4786	5.234892	0.080749	0.080749	0.786184
GOTERM_BP_FAT	GO:0055114 oxidation reduction	20	3.976143	6.994385e-04	110	620	7937	2.327566	0.360071	0.200044	1.036651
SP_PIR_KEYWORDS	pyridoxal phosphate	5	0.994036	8.077844e-04	215	26	12980	11.610018	0.099724	0.034412	0.936670
GOTERM_CC_FAT	GO:0005626 insoluble fraction	8	1.590457	8.543375e-04	69	110	4786	5.044532	0.099791	0.051207	0.980685
GOTERM_CC_FAT	GO:0000267 cell fraction	8	1.590457	0.001003	69	113	4786	4.910607	0.116071	0.040292	1.149950
GOTERM_CC_FAT	GO:0042598 vesicular fraction	7	1.391650	0.001515	69	89	4786	5.455463	0.170164	0.045561	1.733401
GOTERM_CC_FAT	GO:0005792 microsome	7	1.391650	0.001515	69	89	4786	5.455463	0.170164	0.045561	1.733401
SP_PIR_KEYWORDS	Serine protease	9	1.789264	0.001771	215	135	12980	4.024806	0.205849	0.055992	2.043504
SMART	SM00020:Tryp_SPc	11	2.186879	0.001992	68	248	4824	3.146584	0.083998	0.083998	1.848260
UP_SEQ_FEATURE	metal ion-binding site:Iron (heme axial ligand)	7	1.391650	0.002239	44	96	2975	4.930161	0.213266	0.213266	2.488360
GOTERM_BP_FAT	GO:0009408 response to heat	6	1.192843	0.002336	110	68	7937	6.366578	0.775168	0.391931	3.423843
INTERPRO	IPR015421:Pyridoxal phosphate-dependent transferase, major region, subdomain 1	5	0.994036	0.002474	179	33	10196	8.630438	0.534916	0.534916	3.270873
GOTERM_MF_FAT	GO:0008236 serine-type peptidase activity	14	2.783300	0.002878	140	308	7918	2.570779	0.532704	0.223996	3.705406
INTERPRO	IPR008597:Destabilase	3	0.596421	0.002928	179	5	10196	34.176536	0.595846	0.364269	3.859135
GOTERM_MF_FAT	GO:0017171 serine hydrolase activity	14	2.783300	0.003043	140	310	7918	2.554194	0.552740	0.182213	3.914608
GOTERM_MF_FAT	GO:0020037 heme binding	9	1.789264	0.003148	140	140	7918	3.635816	0.564992	0.153359	4.046969
GOTERM_MF_FAT	GO:0046906 tetrapyrrole binding	9	1.789264	0.003148	140	140	7918	3.635816	0.564992	0.153359	4.046969
GOTERM_BP_FAT	GO:0009266 response to temperature stimulus	6	1.192843	0.003189	110	73	7937	5.930511	0.869723	0.399218	4.646268
GOTERM_MF_FAT	GO:0030170 pyridoxal phosphate binding	5	0.994036	0.004646	140	39	7918	7.250916	0.707521	0.185266	5.918894
GOTERM_MF_FAT	GO:0070279 vitamin B6 binding	5	0.994036	0.004646	140	39	7918	7.250916	0.707521	0.185266	5.918894
GOTERM_MF_FAT	GO:0048037 cofactor binding	10	1.988072	0.005163	140	185	7918	3.057143	0.745041	0.177360	6.557764
SP_PIR_KEYWORDS	carboxypeptidase	5	0.994036	0.005823	215	44	12980	6.860465	0.531936	0.140866	6.574444
SMART	SM00700:JHBP	4	0.795229	0.005902	68	27	4824	10.509804	0.229318	0.122115	5.388005
GOTERM_MF_FAT	GO:0005506 iron ion binding	12	2.385686	0.005909	140	260	7918	2.610330	0.790846	0.177647	7.471649
GOTERM_MF_FAT	GO:0004180 carboxypeptidase activity	5	0.994036	0.007762	140	45	7918	6.284127	0.872187	0.204334	9.705861
SP_PIR_KEYWORDS	lipid transport	3	0.596421	0.009025	215	9	12980	20.124031	0.692264	0.178332	10.019040
GOTERM_MF_FAT	GO:0009055 electron carrier activity	10	1.988072	0.009027	140	202	7918	2.799859	0.908722	0.212888	11.201986
SP_PIR_KEYWORDS	iron	9	1.789264	0.009074	215	177	12980	3.069767	0.694263	0.155736	10.071541
GOTERM_MF_FAT	GO:0016831 carboxy-lyase activity	4	0.795229	0.009178	140	25	7918	9.049143	0.912339	0.198522	11.380021
SP_PIR_KEYWORDS	microsome	6	1.192843	0.009271	215	78	12980	4.644007	0.702054	0.140458	10.279246
GOTERM_BP_FAT	GO:0006979 response to oxidative stress	5	0.994036	0.009638	110	61	7937	5.914307	0.997927	0.709382	13.431573
GOTERM_MF_FAT	GO:0004252 serine-type endopeptidase activity	12	2.385686	0.010081	140	280	7918	2.423878	0.931085	0.199810	12.431915
GOTERM_MF_FAT	GO:0008238 exopeptidase activity	7	1.391650	0.010680	140	106	7918	3.734906	0.941258	0.195912	13.123357
GOTERM_BP_FAT	GO:0010817 regulation of hormone levels	4	0.795229	0.011067	110	34	7937	8.488770	0.999175	0.693764	15.274208
GOTERM_BP_FAT	GO:0042445 hormone metabolic process	4	0.795229	0.011067	110	34	7937	8.488770	0.999175	0.693764	15.274208
INTERPRO	IPR004272:Odorant binding protein	4	0.795229	0.011248	179	27	10196	8.438651	0.969656	0.688095	14.086862
INTERPRO	IPR001314:Peptidase S1A, chymotrypsin	10	1.988072	0.011343	179	210	10196	2.712424	0.970553	0.585751	14.198666
GOTERM_BP_FAT	GO:0016053 organic acid biosynthetic process	5	0.994036	0.011374	110	64	7937	5.637074	0.999323	0.647447	15.663896
GOTERM_BP_FAT	GO:0046394 carboxylic acid biosynthetic process	5	0.994036	0.011374	110	64	7937	5.637074	0.999323	0.647447	15.663896
GOTERM_CC_FAT	GO:0005576 extracellular region	15	2.982107	0.011377	69	512	4786	2.032099	0.755220	0.245333	12.360294
INTERPRO	IPR001254:Peptidase S1 and S6, chymotrypsin/Hap	11	2.186879	0.011625	179	248	10196	2.526491	0.973030	0.514516	14.525648
SP_PIR_KEYWORDS	Protease	11	2.186879	0.012258	215	264	12980	2.515504	0.798778	0.163183	13.379097
INTERPRO	IPR011701:Major facilitator superfamily MFS-1	7	1.391650	0.013027	179	111	10196	3.592128	0.982609	0.490997	16.139414
GOTERM_MF_FAT	GO:0004175 endopeptidase activity	16	3.180915	0.013929	140	457	7918	1.980119	0.975355	0.232421	16.788850
INTERPRO	IPR013053:Hormone binding	4	0.795229	0.015029	179	30	10196	7.594786	0.990713	0.487495	18.393819
INTERPRO	IPR017973:Cytochrome P450, C-terminal region	6	1.192843	0.016361	179	85	10196	4.020769	0.993886	0.471206	19.862714
INTERPRO	IPR001128:Cytochrome P450	6	1.192843	0.017132	179	86	10196	3.974016	0.995203	0.447503	20.702551
INTERPRO	IPR002129:Pyridoxal phosphate-dependent decarboxylase	3	0.596421	0.017830	179	12	10196	14.240223	0.996148	0.426461	21.455155
GOTERM_CC_FAT	GO:0005783 endoplasmic reticulum	10	1.988072	0.018046	69	284	4786	2.442335	0.893540	0.311563	18.940569
COG_ONTOLOGY	Secondary metabolites biosynthesis, transport, and catabolism	6	1.192843	0.018656	23	91	1237	3.546106	0.202276	0.202276	11.411090
INTERPRO	IPR018114:Peptidase S1/S6, chymotrypsin/Hap, active site	9	1.789264	0.018787	179	191	10196	2.684021	0.997150	0.413023	22.476077

GOTERM_CC_FAT	GO:0019898 extrinsic to membrane	7	1.391650	0.021334	69	154	4786	3.152833	0.929524	0.315404	22.015250
INTERPRO	IPR017972:Cytochrome P450, conserved site	6	1.192843	0.021353	179	91	10196	3.755663	0.998731	0.426386	25.153649
KEGG_PATHWAY	dme00903:Limonene and pinene degradation	6	1.192843	0.021440	40	86	2054	3.582558	0.614660	0.614660	18.353463
SP_PIR_KEYWORDS	Monooxygenase	6	1.192843	0.022952	215	98	12980	3.696251	0.951127	0.260554	23.692764
GOTERM_BP_FAT	GO:0009628 response to abiotic stimulus	8	1.590457	0.027599	110	214	7937	2.697366	1.000000	0.892683	34.086029
GOTERM_CC_FAT	GO:0005615 extracellular space	5	0.994036	0.028463	69	82	4786	4.229410	0.971325	0.358513	28.319861
GOTERM_MF_FAT	GO:0019842 vitamin binding	6	1.192843	0.029827	140	99	7918	3.427706	0.999663	0.413122	32.749385
SP_PIR_KEYWORDS	endoplasmic reticulum	7	1.391650	0.032026	215	144	12980	2.934755	0.985470	0.319336	31.549884
SP_PIR_KEYWORDS	thiol protease inhibitor	2	0.397614	0.032703	215	2	12980	60.372093	0.986733	0.302469	32.105271
GOTERM_MF_FAT	GO:0003796 lysozyme activity	3	0.596421	0.035026	140	17	7918	9.980672	0.999918	0.444728	37.321561
INTERPRO	IPR000618:Insect cuticle protein	6	1.192843	0.037875	179	106	10196	3.224202	0.999993	0.600585	40.445920
PIR_SUPERFAMILY	PIRSF001135:trypsin	5	0.994036	0.040814	57	61	2596	3.733103	0.890139	0.890139	33.406230
PIR_SUPERFAMILY	PIRSF000051:cytochrome P450 CYP3A5	4	0.795229	0.044037	57	37	2596	4.923661	0.908087	0.696828	35.557354
SP_PIR_KEYWORDS	oxidoreductase	16	3.180915	0.044867	215	561	12980	1.721842	0.997440	0.368113	41.408428
INTERPRO	IPR002401:Cytochrome P450, E-class, group I	5	0.994036	0.045442	179	77	10196	3.698759	0.999999	0.641735	46.435235
SMART	SM00280:KAZAL	3	0.596421	0.046434	68	25	4824	8.512941	0.876568	0.502100	35.907171
INTERPRO	IPR005829:Sugar transporter, conserved site	4	0.795229	0.051059	179	48	10196	4.746741	1.000000	0.660277	50.514859
GOTERM_BP_FAT	GO:0006869 lipid transport	3	0.596421	0.056037	110	28	7937	7.730844	1.000000	0.983228	57.635933
GOTERM_MF_FAT	GO:0015145 monosaccharide transmembrane transporter activity	3	0.596421	0.056217	140	22	7918	7.712338	1.000000	0.592827	53.143722
GOTERM_BP_FAT	GO:0008652 cellular amino acid biosynthetic process	3	0.596421	0.059662	110	29	7937	7.464263	1.000000	0.980252	59.995206
GOTERM_MF_FAT	GO:0042302 structural constituent of cuticle	6	1.192843	0.059670	140	120	7918	2.827857	1.000000	0.594390	55.340991
SMART	SM00708:PhBP	3	0.596421	0.060667	68	29	4824	7.338742	0.936311	0.497640	44.319024
GOTERM_CC_FAT	GO:0005811 lipid particle	8	1.590457	0.061673	69	249	4786	2.228508	0.999602	0.581039	52.001685
SP_PIR_KEYWORDS	Secreted	8	1.590457	0.061719	215	212	12980	2.278192	0.999747	0.446534	52.378510
GOTERM_BP_FAT	GO:0048878 chemical homeostasis	4	0.795229	0.061891	110	66	7937	4.373003	1.000000	0.975414	61.384679
PIR_SUPERFAMILY	PIRSF001127:serine carboxypeptidase	2	0.397614	0.063353	57	3	2596	30.362573	0.968845	0.685339	47.194227
SMART	SM00043:CY	2	0.397614	0.067569	68	5	4824	28.376471	0.953961	0.459711	48.031486
INTERPRO	IPR018073:Proteinase inhibitor I25, cystatin, conserved site	2	0.397614	0.068034	179	4	10196	28.480447	1.000000	0.743526	61.162408
INTERPRO	IPR002350:Proteinase inhibitor I1, Kazal	3	0.596421	0.069962	179	25	10196	6.835307	1.000000	0.732419	62.227325
GOTERM_BP_FAT	GO:0042592 homeostatic process	6	1.192843	0.073322	110	163	7937	2.655996	1.000000	0.982554	67.829520
UP_SEQ_FEATURE	disulfide bond	8	1.590457	0.073645	44	258	2975	2.096547	0.999721	0.983305	57.678886
GOTERM_BP_FAT	GO:0010876 lipid localization	3	0.596421	0.074920	110	33	7937	6.559504	1.000000	0.978113	68.645725
GOTERM_MF_FAT	GO:0008237 metallopeptidase activity	7	1.391650	0.074974	140	168	7918	2.356548	1.000000	0.661375	63.980199
GOTERM_BP_FAT	GO:0019748 secondary metabolic process	4	0.795229	0.076126	110	72	7937	4.008586	1.000000	0.972903	69.248911
GOTERM_MF_FAT	GO:0051119 sugar transmembrane transporter activity	3	0.596421	0.080751	140	27	7918	6.284127	1.000000	0.670907	66.818806
INTERPRO	IPR001563:Peptidase S10, serine carboxypeptidase	2	0.397614	0.084310	179	5	10196	22.784358	1.000000	0.779531	69.342272
INTERPRO	IPR000010:Proteinase inhibitor I25, cystatin	2	0.397614	0.084310	179	5	10196	22.784358	1.000000	0.779531	69.342272
INTERPRO	IPR000192:Aminotransferase, class V/Cysteine desulfurase	2	0.397614	0.084310	179	5	10196	22.784358	1.000000	0.779531	69.342272
INTERPRO	IPR006625:Insect pheromone/odorant binding protein PhBP	3	0.596421	0.090545	179	29	10196	5.892506	1.000000	0.786373	72.028657
INTERPRO	IPR017849:Alkaline phosphatase-like, alpha/beta/alpha	3	0.596421	0.090545	179	29	10196	5.892506	1.000000	0.786373	72.028657
UP_SEQ_FEATURE	sequence variant	7	1.391650	0.096819	44	222	2975	2.131962	0.999981	0.973537	68.166600

Table 3: Architecture 3

Category	Term	Count	%	PValue	List Total	Pop Hits	Pop Total	Fold Enrichment	Bonferroni	Benjamini	FDR
KEGG_PATHWAY	dme00910:Nitrogen metabolism	4	0.656814	0.002107	26	22	2054	14.363636	0.053365	0.053365	1.714909
GOTERM_BP_FAT	GO:0007369 gastrulation	5	0.821018	0.004229	74	72	7937	7.448386	0.947855	0.947855	6.187440
INTERPRO	IPR000618:Insect cuticle protein	6	0.985222	0.005077	108	106	10196	5.343816	0.678608	0.678608	6.287178
GOTERM_MF_FAT	GO:0005214 structural constituent of chitin-based cuticle	6	0.985222	0.005519	83	110	7918	5.203505	0.665691	0.665691	6.690301
GOTERM_MF_FAT	GO:0042302 structural constituent of cuticle	6	0.985222	0.007932	83	120	7918	4.769880	0.793363	0.545426	9.484681
INTERPRO	IPR001148:Carbonic anhydrase, alpha-class, catalytic domain	3	0.492611	0.010480	108	15	10196	18.881481	0.904563	0.691071	12.575579
GOTERM_CC_FAT	GO:0005811 lipid particle	8	1.313629	0.011200	49	249	4786	3.138103	0.752584	0.752584	12.196221
GOTERM_MF_FAT	GO:0004089 carbonate dehydratase activity	3	0.492611	0.014558	83	18	7918	15.899598	0.945181	0.620122	16.765723
GOTERM_MF_FAT	GO:0005198 structural molecule activity	11	1.806240	0.018766	83	456	7918	2.301258	0.976504	0.608485	21.105076
COG_ONTOLOGY	Translation, ribosomal structure and biogenesis	4	0.656814	0.020956	14	59	1237	5.990315	0.137782	0.137782	10.343181
GOTERM_BP_FAT	GO:0006952 defense response	6	0.985222	0.021185	74	173	7937	3.719888	1.000000	0.999426	27.583421
SP_PIR_KEYWORDS	stress response	3	0.492611	0.024206	138	23	12980	12.268431	0.941716	0.941716	24.388270
GOTERM_MF_FAT	GO:0016811 hydrolase activity, acting on carbon-nitrogen (but not peptide) bonds, in linear amides	4	0.656814	0.025056	83	61	7918	6.255580	0.993423	0.633899	27.205097
SMART	SM00382:AAA	5	0.821018	0.026240	45	125	4824	4.288000	0.672668	0.672668	21.814824
KEGG_PATHWAY	dme00790:Folate biosynthesis	3	0.492611	0.028314	26	22	2054	10.772727	0.526108	0.311602	20.985891
INTERPRO	IPR003593:ATPase, AAA+ type, core	5	0.821018	0.042330	108	125	10196	3.776296	0.999935	0.959848	42.407421
GOTERM_MF_FAT	GO:0051082 unfolded protein binding	4	0.656814	0.042442	83	75	7918	5.087871	0.999813	0.760971	41.881621
GOTERM_BP_FAT	GO:0007422 peripheral nervous system development	4	0.656814	0.047008	74	88	7937	4.875307	1.000000	0.999986	51.601222
GOTERM_BP_FAT	GO:0012501 programmed cell death	5	0.821018	0.051944	74	153	7937	3.505123	1.000000	0.999908	55.244622
GOTERM_BP_FAT	GO:0048598 embryonic morphogenesis	6	0.985222	0.056467	74	226	7937	2.847525	1.000000	0.999697	58.356923
INTERPRO	IPR000120:Amidase signature enzyme	2	0.328407	0.061351	108	6	10196	31.469136	0.999999	0.970688	55.412110
GOTERM_BP_FAT	GO:0008219 cell death	5	0.821018	0.061585	74	162	7937	3.310394	1.000000	0.999379	61.634733
GOTERM_BP_FAT	GO:0016265 death	5	0.821018	0.062711	74	163	7937	3.290085	1.000000	0.998417	62.322941
GOTERM_BP_FAT	GO:0009952 anterior/posterior pattern formation	5	0.821018	0.070900	74	170	7937	3.154610	1.000000	0.998350	66.990658
SMART	SM00587:CHK	3	0.492611	0.075620	45	50	4824	6.432000	0.963210	0.808194	51.699888
GOTERM_MF_FAT	GO:0004540 ribonuclease activity	3	0.492611	0.075799	83	44	7918	6.504381	1.000000	0.892435	62.707541
GOTERM_MF_FAT	GO:0016836 hydro-lyase activity	3	0.492611	0.078808	83	45	7918	6.359839	1.000000	0.868881	64.198566
INTERPRO	IPR018338:Carbonic anhydrase, alpha-class, conserved site	2	0.328407	0.080961	108	8	10196	23.601852	1.000000	0.976843	65.940122
SP_PIR_KEYWORDS	cell cycle control	2	0.328407	0.081404	138	8	12980	23.514493	0.999947	0.992735	62.043013
GOTERM_MF_FAT	GO:0005524 ATP binding	13	2.134647	0.082868	83	748	7918	1.657980	1.000000	0.850891	66.123489
GOTERM_MF_FAT	GO:0032559 adenyl ribonucleotide binding	13	2.134647	0.084162	83	750	7918	1.653558	1.000000	0.824608	66.716912
GOTERM_BP_FAT	GO:0006457 protein folding	4	0.656814	0.089014	74	115	7937	3.730670	1.000000	0.999268	75.466400
INTERPRO	IPR004119:Protein of unknown function DUF227	3	0.492611	0.096632	108	50	10196	5.664444	1.000000	0.977111	72.650013
INTERPRO	IPR015897:CHK kinase-like	3	0.492611	0.096632	108	50	10196	5.664444	1.000000	0.977111	72.650013

Table 4: Architecture 4

Category	Term	Count	%	PValue	List Total	Pop Hits	Pop Total	Fold Enrichment	Bonferroni	Benjamini	FDR
GOTERM_CC_FAT	GO:0005576 extracellular region	13	7.471264	1.856613e-05	30	512	4786	4.050651	0.001299	0.001299	0.019213
SP_PIR_KEYWORDS	Secreted	7	4.022989	0.001709	79	212	12980	5.425125	0.133836	0.133836	1.819268
SMART	SM00477:NUC	3	1.724138	0.002266	28	13	4824	39.758242	0.046516	0.046516	1.736115
INTERPRO	IPR001604:DNA/RNA non-specific endonuclease	3	1.724138	0.002465	60	13	10196	39.215385	0.210927	0.210927	2.681283
GOTERM_BP_FAT	GO:0007600 sensory perception	6	3.448276	0.010724	40	276	7937	4.313587	0.965026	0.965026	13.486438
UP_SEQ_FEATURE	signal peptide	8	4.597701	0.011429	20	419	2975	2.840095	0.515277	0.515277	10.987375
SP_PIR_KEYWORDS	signal	8	4.597701	0.013779	79	425	12980	3.092777	0.688241	0.441646	13.837512
INTERPRO	IPR006170: Pheromone/ general odorant binding protein, PBP/GOBP	3	1.724138	0.017330	60	35	10196	14.565714	0.813302	0.567915	17.514311
GOTERM_BP_FAT	GO:0006030 chitin metabolic process	4	2.298851	0.018122	40	114	7937	6.962281	0.996612	0.941796	21.786305
GOTERM_MF_FAT	GO:0008061 chitin binding	4	2.298851	0.018906	46	100	7918	6.885217	0.907992	0.907992	19.806244
GOTERM_BP_FAT	GO:0050890 cognition	6	3.448276	0.027226	40	350	7937	3.401571	0.999813	0.942819	30.987906
GOTERM_BP_FAT	GO:0006022 aminoglycan metabolic process	4	2.298851	0.032057	40	142	7937	5.589437	0.999960	0.920602	35.453898
GOTERM_MF_FAT	GO:0030247 polysaccharide binding	4	2.298851	0.033014	46	124	7918	5.552595	0.984950	0.877322	32.173693
GOTERM_MF_FAT	GO:0001871 pattern binding	4	2.298851	0.033014	46	124	7918	5.552595	0.984950	0.877322	32.173693
PIR_SUPERFAMILY	PIRSF036514:alpha-crystallin-related small heat shock protein	2	1.149425	0.037187	15	7	2596	49.447619	0.411719	0.411719	22.696215
GOTERM_BP_FAT	GO:0005976 polysaccharide metabolic process	4	2.298851	0.038714	40	153	7937	5.187582	0.999995	0.914209	41.169366
GOTERM_MF_FAT	GO:0005198 structural molecule activity	7	4.022989	0.042765	46	456	7918	2.642353	0.995760	0.838152	39.675070
GOTERM_BP_FAT	GO:0042742 defense response to bacterium	3	1.724138	0.044899	40	69	7937	8.627174	0.999999	0.907553	46.056888
INTERPRO	IPR013172:DIM, Drosophila melanogaster	2	1.149425	0.045381	60	8	10196	42.483333	0.988421	0.773765	40.041824
GOTERM_BP_FAT	GO:0019730 antimicrobial humoral response	3	1.724138	0.046079	40	70	7937	8.503929	1.000000	0.877037	46.945395
GOTERM_BP_FAT	GO:0006955 immune response	4	2.298851	0.049560	40	169	7937	4.696450	1.000000	0.861381	49.488832
INTERPRO	IPR001436:Alpha crystallin/Heat shock protein	2	1.149425	0.050909	60	9	10196	37.762963	0.993370	0.714646	43.757170
GOTERM_BP_FAT	GO:0006952 defense response	4	2.298851	0.052483	40	173	7937	4.587861	1.000000	0.844778	51.536464
GOTERM_BP_FAT	GO:0009617 response to bacterium	3	1.724138	0.058486	40	80	7937	7.440937	1.000000	0.846534	55.503442
GOTERM_BP_FAT	GO:0006959 humoral immune response	3	1.724138	0.062412	40	83	7937	7.171988	1.000000	0.838304	57.933050
INTERPRO	IPR002068:Heat shock protein Hsp20	2	1.149425	0.067306	60	12	10196	28.322222	0.998756	0.737584	53.579800
GOTERM_BP_FAT	GO:0007606 sensory perception of chemical stimulus	4	2.298851	0.069162	40	194	7937	4.091237	1.000000	0.843930	61.824909
SP_PIR_KEYWORDS	disulfide bond	5	2.873563	0.075196	79	265	12980	3.100072	0.998593	0.887956	56.790323
UP_SEQ_FEATURE	disulfide bond	5	2.873563	0.075892	20	258	2975	2.882752	0.993073	0.916773	55.029866
GOTERM_BP_FAT	GO:0051606 detection of stimulus	3	1.724138	0.086232	40	100	7937	5.952750	1.000000	0.884369	70.230127
GOTERM_BP_FAT	GO:0009628 response to abiotic stimulus	4	2.298851	0.087026	40	214	7937	3.708879	1.000000	0.867683	70.575733
SMART	SM00494:ChtBD2	3	1.724138	0.092867	28	92	4824	5.618012	0.870850	0.640626	52.884545
GOTERM_MF_FAT	GO:0030246 carbohydrate binding	4	2.298851	0.093905	46	191	7918	3.604826	0.999996	0.954115	68.029435
INTERPRO	IPR002557:Chitin binding protein, peritrophin-A	3	1.724138	0.099071	60	92	10196	5.541304	0.999955	0.811615	68.307130

Table 5: Architecture 5

Category	Term	Count	%	PValue	List Total	Pop Hits	Pop Total	Fold Enrichment	Bonferroni	Benjamini	FDR
GOTERM_CC_FAT	GO:0005576 extracellular region	14	2.713178	2.152775e-04	42	512	4786	3.115885	0.011984	0.011984	0.212370
SP_PIR_KEYWORDS	Lectin	4	0.775194	0.001980	103	32	12980	15.752427	0.170007	0.170007	2.151109
GOTERM_MF_FAT	GO:0005213 structural constituent of chorion	3	0.581395	0.002285	59	10	7918	40.261017	0.309709	0.309709	2.730522
GOTERM_CC_FAT	GO:0005615 extracellular space	5	0.968992	0.005008	42	82	4786	6.948316	0.245066	0.131131	4.836166
SP_PIR_KEYWORDS	Secreted	7	1.356589	0.006495	103	212	12980	4.161019	0.457995	0.263790	6.898236
GOTERM_CC_FAT	GO:0042600 chorion	3	0.581395	0.010045	42	18	4786	18.992063	0.431858	0.171767	9.488475
GOTERM_CC_FAT	GO:0030312 external encapsulating structure	3	0.581395	0.011167	42	19	4786	17.992481	0.466795	0.145478	10.495736
GOTERM_MF_FAT	GO:0030246 carbohydrate binding	6	1.162791	0.012634	59	191	7918	4.215813	0.872503	0.642933	14.259559
SP_PIR_KEYWORDS	signal	9	1.744186	0.018657	103	425	12980	2.668646	0.829723	0.445734	18.665449
UP_SEQ_FEATURE	signal peptide	9	1.744186	0.022239	27	419	2975	2.366746	0.778386	0.778386	20.599260
INTERPRO	IPR009392:Drosophila ACP53EA	2	0.387597	0.022487	78	3	10196	87.145299	0.967754	0.967754	23.808116
INTERPRO	IPR005649:Chorion 2	2	0.387597	0.022487	78	3	10196	87.145299	0.967754	0.967754	23.808116
SP_PIR_KEYWORDS	storage protein	2	0.387597	0.031068	103	4	12980	63.009709	0.948528	0.523686	29.264199
GOTERM_MF_FAT	GO:0030170 pyridoxal phosphate binding	3	0.581395	0.032861	59	39	7918	10.323338	0.995541	0.835408	33.256335
GOTERM_MF_FAT	GO:0070279 vitamin B6 binding	3	0.581395	0.032861	59	39	7918	10.323338	0.995541	0.835408	33.256335
GOTERM_CC_FAT	GO:0044421 extracellular region part	5	0.968992	0.039040	42	151	4786	3.773258	0.892477	0.359823	32.512064
PIR_SUPERFAMILY	PIRSF005266:arylphorin	2	0.387597	0.039486	27	4	2596	48.074074	0.713179	0.713179	29.255798
GOTERM_MF_FAT	GO:0045735 nutrient reservoir activity	2	0.387597	0.043167	59	6	7918	44.734463	0.999214	0.832557	41.371513
INTERPRO	IPR000896:Hemocyanin, copper-containing	2	0.387597	0.044476	78	6	10196	43.572650	0.998961	0.967771	41.952633
GOTERM_MF_FAT	GO:0005529 sugar binding	3	0.581395	0.046196	59	47	7918	8.566174	0.999530	0.783987	43.578247
GOTERM_CC_FAT	GO:0005616 larval serum protein complex	2	0.387597	0.050337	42	6	4786	37.984127	0.944552	0.382485	39.950210
GOTERM_BP_FAT	GO:0016337 cell-cell adhesion	3	0.581395	0.053005	51	59	7937	7.913260	1.000000	1.000000	53.854468
KEGG_PATHWAY	dme00680:Methane metabolism	2	0.387597	0.053333	15	8	2054	34.233333	0.745945	0.745945	35.896500
SP_PIR_KEYWORDS	heme	4	0.775194	0.056887	103	111	12980	4.541240	0.995936	0.667493	47.402097
INTERPRO	IPR008922:Di-copper centre-containing	2	0.387597	0.058863	78	8	10196	32.679487	0.999895	0.952808	51.581387
GOTERM_CC_FAT	GO:0005624 membrane fraction	4	0.775194	0.061357	42	106	4786	4.300090	0.971158	0.397436	46.487070
INTERPRO	IPR005204:Hemocyanin, N-terminal	2	0.387597	0.065976	78	9	10196	29.048433	0.999967	0.923963	55.779726
INTERPRO	IPR013788:Arthropod hemocyanin/insect LSP	2	0.387597	0.065976	78	9	10196	29.048433	0.999967	0.923963	55.779726
INTERPRO	IPR005203:Hemocyanin, C-terminal	2	0.387597	0.065976	78	9	10196	29.048433	0.999967	0.923963	55.779726
INTERPRO	IPR002403:Cytochrome P450, E-class, group IV	2	0.387597	0.065976	78	9	10196	29.048433	0.999967	0.923963	55.779726
GOTERM_CC_FAT	GO:0005626 insoluble fraction	4	0.775194	0.067065	42	110	4786	4.143723	0.979504	0.384881	49.615119
GOTERM_CC_FAT	GO:0000267 cell fraction	4	0.775194	0.071503	42	113	4786	4.033713	0.984306	0.369733	51.932144
INTERPRO	IPR007614:Retinin-like protein	2	0.387597	0.080043	78	11	10196	23.766900	0.999997	0.919503	63.116853
SMART	SM00494:ChtBD2	3	0.581395	0.081315	26	92	4824	6.050167	0.857822	0.857822	48.944148
GOTERM_MF_FAT	GO:0046906 tetrapyrrole binding	4	0.775194	0.082717	59	140	7918	3.834383	0.999999	0.902818	64.822163
GOTERM_MF_FAT	GO:0020037 heme binding	4	0.775194	0.082717	59	140	7918	3.834383	0.999999	0.902818	64.822163
GOTERM_MF_FAT	GO:0005344 oxygen transporter activity	2	0.387597	0.091217	59	13	7918	20.646675	1.000000	0.890693	68.569908
GOTERM_BP_FAT	GO:0007155 cell adhesion	4	0.775194	0.096189	51	174	7937	3.577643	1.000000	1.000000	76.216162
GOTERM_BP_FAT	GO:0003006 reproductive developmental process	7	1.356589	0.096508	51	506	7937	2.152949	1.000000	1.000000	76.335129

Table 6: Architecture 6

Category	Term	Count	%	PValue	List Total	Pop Hits	Pop Total	Fold Enrichment	Bonferroni	Benjamini	FDR
GOTERM_CC_FAT	GO:0005576 extracellular region	8	1.349073	0.003436	21	512	4786	3.561012	0.101212	0.101212	2.913855
GOTERM_BP_FAT	GO:0016042 lipid catabolic process	3	0.505902	0.013168	32	45	7937	16.535417	0.990336	0.990336	16.580500
SP_PIR_KEYWORDS	Acyltransferase	4	0.674536	0.015018	60	114	12980	7.590643	0.590483	0.590483	14.024127
GOTERM_MF_FAT	GO:0005198 structural molecule activity	7	1.180438	0.017911	38	456	7918	3.198638	0.897438	0.897438	18.885275
GOTERM_CC_FAT	GO:0005811 lipid particle	5	0.843170	0.017914	21	249	4786	4.576401	0.428999	0.244354	14.383367
SP_PIR_KEYWORDS	Serine protease	4	0.674536	0.023428	60	135	12980	6.409877	0.753082	0.503092	21.079688
INTERPRO	IPR016040:NAD(P)-binding domain	4	0.674536	0.025278	45	147	10196	6.165382	0.922720	0.922720	24.736796
UP_SEQ_FEATURE	signal peptide	6	1.011804	0.026371	14	419	2975	3.042959	0.787765	0.787765	23.348278
SP_PIR_KEYWORDS	Protease	5	0.843170	0.031820	60	264	12980	4.097222	0.851606	0.470574	27.596216
GOTERM_BP_FAT	GO:0006644 phospholipid metabolic process	3	0.505902	0.042213	32	84	7937	8.858259	1.000000	0.999473	44.560559
INTERPRO	IPR016038:Thiolase-like, subgroup	2	0.337268	0.042344	45	10	10196	45.315556	0.986789	0.885059	38.136023
SP_PIR_KEYWORDS	signal	6	1.011804	0.043436	60	425	12980	3.054118	0.927199	0.480561	35.817710
GOTERM_MF_FAT	GO:0005213 structural constituent of chorion	2	0.337268	0.045784	38	10	7918	41.673684	0.997274	0.947791	41.884026
GOTERM_BP_FAT	GO:0019637 organophosphate metabolic process	3	0.505902	0.054655	32	97	7937	7.671070	1.000000	0.998580	53.638857
SP_PIR_KEYWORDS	hydrolase	12	2.023609	0.064917	60	1470	12980	1.765986	0.980939	0.547070	48.841974
SP_PIR_KEYWORDS	Secreted	4	0.674536	0.071793	60	212	12980	4.081761	0.987668	0.519338	52.476799
GOTERM_BP_FAT	GO:0006030 chitin metabolic process	3	0.505902	0.072607	32	114	7937	6.527138	1.000000	0.998634	64.332248
GOTERM_CC_FAT	GO:0042600 chorion	2	0.337268	0.072731	21	18	4786	25.322751	0.903755	0.541725	47.727918
GOTERM_CC_FAT	GO:0030312 external encapsulating structure	2	0.337268	0.076620	21	19	4786	23.989975	0.915513	0.460866	49.581852
GOTERM_MF_FAT	GO:0005316 high affinity inorganic phosphate:sodium symporter activity	2	0.337268	0.080937	38	18	7918	23.152047	0.999976	0.971126	62.371514
INTERPRO	IPR011701:Major facilitator superfamily MFS-1	3	0.505902	0.082712	45	111	10196	6.123724	0.999822	0.943741	61.643527
GOTERM_BP_FAT	GO:0055114 oxidation reduction	6	1.011804	0.089887	32	620	7937	2.400302	1.000000	0.998630	72.422488
GOTERM_MF_FAT	GO:0005436 sodium:phosphate symporter activity	2	0.337268	0.093791	38	21	7918	19.844612	0.999996	0.955054	68.034956
GOTERM_BP_FAT	GO:0044242 cellular lipid catabolic process	2	0.337268	0.096889	32	26	7937	19.079327	1.000000	0.997380	75.186789

Table 7: Architecture 7

Category	Term	Count	%	PValue	List Total	Pop Hits	Pop Total	Fold Enrichment	Bonferroni	Benjamini	FDR
GOTERM_CC_FAT	GO:0005578 proteinaceous extracellular matrix	8	0.394477	3.670529e-04	132	50	4786	5.801212	0.067718	0.067718	0.455635
GOTERM_CC_FAT	GO:0031012 extracellular matrix	8	0.394477	5.290937e-04	132	53	4786	5.472842	0.096143	0.049286	0.656174
UP_SEQ_FEATURE	splice variant	30	1.479290	5.898765e-04	81	604	2975	1.824258	0.181774	0.181774	0.800302
GOTERM_BP_FAT	GO:0060538 skeletal muscle organ development	8	0.394477	6.864553e-04	210	57	7937	5.304595	0.577888	0.577888	1.110243
GOTERM_CC_FAT	GO:0005811 lipid particle	17	0.838264	0.001120	132	249	4786	2.475417	0.192660	0.068852	1.384137
GOTERM_BP_FAT	GO:0048732 gland development	13	0.641026	0.001200	210	163	7937	3.014344	0.778561	0.529427	1.932622
GOTERM_BP_FAT	GO:0044271 nitrogen compound biosynthetic process	16	0.788955	0.001212	210	233	7937	2.595381	0.782087	0.398234	1.952996
SP_PIR_KEYWORDS	DNA binding	11	0.542406	0.001304	336	123	12980	3.454801	0.234749	0.234749	1.629043
SP_PIR_KEYWORDS	alternative splicing	30	1.479290	0.001306	336	616	12980	1.881378	0.234990	0.125352	1.630945
GOTERM_BP_FAT	GO:0048707 instar larval or pupal morphogenesis	20	0.986193	0.001941	210	347	7937	2.178400	0.912823	0.456623	3.108889
GOTERM_BP_FAT	GO:0009161 ribonucleoside monophosphate metabolic process	5	0.246548	0.001966	210	21	7937	8.998866	0.915559	0.390026	3.148874
GOTERM_BP_FAT	GO:0009156 ribonucleoside monophosphate biosynthetic process	5	0.246548	0.001966	210	21	7937	8.998866	0.915559	0.390026	3.148874
GOTERM_BP_FAT	GO:0009886 post-embryonic morphogenesis	20	0.986193	0.002358	210	353	7937	2.141373	0.948441	0.389923	3.765384
SP_PIR_KEYWORDS	oxidoreductase	27	1.331361	0.002864	336	561	12980	1.859244	0.444507	0.177960	3.544671
GOTERM_BP_FAT	GO:0007552 metamorphosis	20	0.986193	0.003124	210	362	7937	2.088135	0.980358	0.429612	4.960084
GOTERM_BP_FAT	GO:0042692 muscle cell differentiation	8	0.394477	0.003207	210	74	7937	4.085972	0.982298	0.396047	5.087954
GOTERM_BP_FAT	GO:0055114 oxidation reduction	29	1.429980	0.003289	210	620	7937	1.767842	0.984046	0.368580	5.215608
GOTERM_BP_FAT	GO:0007431 salivary gland development	11	0.542406	0.003590	210	139	7937	2.990990	0.989081	0.363469	5.679701
GOTERM_BP_FAT	GO:0035272 exocrine system development	11	0.542406	0.003590	210	139	7937	2.990990	0.989081	0.363469	5.679701
INTERPRO	IPR016040:NAD(P)-binding domain	12	0.591716	0.004369	304	147	10196	2.737916	0.931395	0.931395	6.277898
GOTERM_BP_FAT	GO:0009791 post-embryonic development	22	1.084813	0.004982	210	434	7937	1.915888	0.998114	0.434646	7.799614
GOTERM_BP_FAT	GO:0055002 striated muscle cell development	6	0.295858	0.006264	210	45	7937	5.039365	0.999626	0.481940	9.711342
GOTERM_BP_FAT	GO:0055001 muscle cell development	6	0.295858	0.006264	210	45	7937	5.039365	0.999626	0.481940	9.711342
GOTERM_BP_FAT	GO:0046395 carboxylic acid catabolic process	6	0.295858	0.006264	210	45	7937	5.039365	0.999626	0.481940	9.711342
GOTERM_BP_FAT	GO:0016054 organic acid catabolic process	6	0.295858	0.006264	210	45	7937	5.039365	0.999626	0.481940	9.711342
GOTERM_BP_FAT	GO:0002165 instar larval or pupal development	21	1.035503	0.006882	210	418	7937	1.898804	0.999829	0.486861	10.620412
GOTERM_BP_FAT	GO:0051146 striated muscle cell differentiation	7	0.345168	0.007040	210	65	7937	4.070256	0.999860	0.469434	10.851107
GOTERM_BP_FAT	GO:0009260 ribonucleotide biosynthetic process	9	0.443787	0.007086	210	107	7937	3.179039	0.999868	0.448701	10.918945
GOTERM_BP_FAT	GO:0009309 amine biosynthetic process	6	0.295858	0.007538	210	47	7937	4.824924	0.999925	0.447893	11.576130
GOTERM_CC_FAT	GO:0031224 intrinsic to membrane	52	2.564103	0.007709	132	1382	4786	1.364250	0.771953	0.308956	9.178331
GOTERM_MF_FAT	GO:0015293 symporter activity	8	0.394477	0.008002	218	84	7918	3.459152	0.973310	0.973310	10.775890
GOTERM_MF_FAT	GO:0008083 growth factor activity	5	0.246548	0.008597	218	30	7918	6.053517	0.979632	0.857284	11.531620
GOTERM_CC_FAT	GO:0016021 integral to membrane	51	2.514793	0.009188	132	1360	4786	1.359659	0.828462	0.297135	10.847086
GOTERM_BP_FAT	GO:0009259 ribonucleotide metabolic process	9	0.443787	0.009251	210	112	7937	3.037117	0.999991	0.496732	14.023745
GOTERM_BP_FAT	GO:0007507 heart development	7	0.345168	0.009371	210	69	7937	3.834300	0.999993	0.481593	14.194041
GOTERM_MF_FAT	GO:0015171 amino acid transmembrane transporter activity	6	0.295858	0.009701	218	48	7918	4.540138	0.987683	0.769056	12.920674
GOTERM_BP_FAT	GO:0007435 salivary gland morphogenesis	9	0.443787	0.010242	210	114	7937	2.983835	0.999998	0.493652	15.411753
GOTERM_BP_FAT	GO:0022612 gland morphogenesis	9	0.443787	0.010242	210	114	7937	2.983835	0.999998	0.493652	15.411753
GOTERM_BP_FAT	GO:0009063 cellular amino acid catabolic process	5	0.246548	0.010510	210	33	7937	5.726551	0.999998	0.484981	15.784266
GOTERM_BP_FAT	GO:0048569 post-embryonic organ development	16	0.788955	0.011760	210	297	7937	2.036107	1.000000	0.507146	17.497037
GOTERM_BP_FAT	GO:0007444 imaginal disc development	20	0.986193	0.012113	210	412	7937	1.834720	1.000000	0.501314	17.974903
INTERPRO	IPR013781:Glycoside hydrolase, subgroup, catalytic core	6	0.295858	0.012396	304	47	10196	4.281635	0.999516	0.978005	16.866842
INTERPRO	IPR011993:Pleckstrin homology-type	9	0.443787	0.012810	304	105	10196	2.874812	0.999626	0.927923	17.380544
GOTERM_BP_FAT	GO:0006726 eye pigment biosynthetic process	5	0.246548	0.012912	210	35	7937	5.399320	1.000000	0.508210	19.046663
GOTERM_BP_FAT	GO:0007519 skeletal muscle tissue development	5	0.246548	0.012912	210	35	7937	5.399320	1.000000	0.508210	19.046663
INTERPRO	IPR013120:Male sterility, NAD-binding	4	0.197239	0.012970	304	17	10196	7.891641	0.999661	0.864317	17.579552
INTERPRO	IPR004262:Male sterility	4	0.197239	0.012970	304	17	10196	7.891641	0.999661	0.864317	17.579552
GOTERM_MF_FAT	GO:0005088 Ras guanyl-nucleotide exchange factor activity	5	0.246548	0.013363	218	34	7918	5.341338	0.997682	0.780589	17.379789
GOTERM_BP_FAT	GO:0042441 eye pigment metabolic process	5	0.246548	0.014234	210	36	7937	5.249339	1.000000	0.527750	20.790932
GOTERM_BP_FAT	GO:0009310 amine catabolic process	5	0.246548	0.014234	210	36	7937	5.249339	1.000000	0.527750	20.790932

GOTERM_BP_FAT	GO:0046148 pigment biosynthetic process	6	0.295858	0.015530	210	56	7937	4.049490	1.000000	0.544490	22.467328
GOTERM_CC_FAT	GO:0043292 contractile fiber	4	0.197239	0.016250	132	20	4786	7.251515	0.956253	0.406404	18.438010
INTERPRO	IPR000731:Sterol-sensing 5TM box	3	0.147929	0.016745	304	7	10196	14.374060	0.999968	0.873433	22.126618
GOTERM_BP_FAT	GO:0035296 regulation of tube diameter	3	0.147929	0.017404	210	8	7937	14.173214	1.000000	0.571785	24.832122
GOTERM_BP_FAT	GO:0048563 post-embryonic organ morphogenesis	15	0.739645	0.017586	210	284	7937	1.996227	1.000000	0.561919	25.058628
GOTERM_BP_FAT	GO:0007560 imaginal disc morphogenesis	15	0.739645	0.017586	210	284	7937	1.996227	1.000000	0.561919	25.058628
KEGG_PATHWAY	dme04142:Lysosome	8	0.394477	0.017700	65	88	2054	2.872727	0.645065	0.645065	16.279395
SP_PIR_KEYWORDS	phosphoprotein	32	1.577909	0.019736	336	815	12980	1.516798	0.983199	0.639976	22.186406
SP_PIR_KEYWORDS	disulfide bond	14	0.690335	0.020249	336	265	12980	2.040881	0.984908	0.567736	22.697043
GOTERM_BP_FAT	GO:0014706 striated muscle tissue development	5	0.246548	0.020362	210	40	7937	4.724405	1.000000	0.602595	28.428091
GOTERM_BP_FAT	GO:0006865 amino acid transport	5	0.246548	0.020362	210	40	7937	4.724405	1.000000	0.602595	28.428091
GOTERM_MF_FAT	GO:0005275 amine transmembrane transporter activity	6	0.295858	0.020855	218	58	7918	3.757355	0.999926	0.850587	25.851267
GOTERM_BP_FAT	GO:0007626 locomotory behavior	11	0.542406	0.021009	210	181	7937	2.296948	1.000000	0.601327	29.193374
SP_PIR_KEYWORDS	calcium	10	0.493097	0.021413	336	158	12980	2.444997	0.988173	0.522679	23.845397
GOTERM_BP_FAT	GO:0048066 pigmentation during development	6	0.295858	0.021780	210	61	7937	3.717564	1.000000	0.602246	30.093880
GOTERM_CC_FAT	GO:0044421 extracellular region part	10	0.493097	0.021830	132	151	4786	2.401164	0.985238	0.452419	24.009378
GOTERM_BP_FAT	GO:0006586 indolalkylamine metabolic process	3	0.147929	0.021992	210	9	7937	12.598413	1.000000	0.593825	30.339997
GOTERM_MF_FAT	GO:0015294 solute:cation symporter activity	7	0.345168	0.022098	218	80	7918	3.178096	0.999958	0.813556	27.175177
GOTERM_BP_FAT	GO:0048069 eye pigmentation	5	0.246548	0.022111	210	41	7937	4.609175	1.000000	0.584218	30.477759
GOTERM_BP_FAT	GO:0060537 muscle tissue development	5	0.246548	0.022111	210	41	7937	4.609175	1.000000	0.584218	30.477759
GOTERM_BP_FAT	GO:0015837 amine transport	5	0.246548	0.023949	210	42	7937	4.499433	1.000000	0.602522	32.572380
SP_PIR_KEYWORDS	dioxygenase	3	0.147929	0.026061	336	10	12980	11.589286	0.995543	0.538531	28.274165
GOTERM_BP_FAT	GO:0043473 pigmentation	6	0.295858	0.026216	210	64	7937	3.543304	1.000000	0.625210	35.074245
GOTERM_MF_FAT	GO:0015370 solute:sodium symporter activity	6	0.295858	0.026995	218	62	7918	3.514945	0.999996	0.828495	32.183334
GOTERM_BP_FAT	GO:0042398 cellular amino acid derivative biosynthetic process	4	0.197239	0.027023	210	25	7937	6.047238	1.000000	0.625846	35.943113
INTERPRO	IPR013090:Phospholipase A2, active site	3	0.147929	0.027602	304	9	10196	11.179825	1.000000	0.942445	33.934057
SMART	SM00325:RhoGEF	4	0.197239	0.029326	138	24	4824	5.826087	0.967383	0.967383	28.754759
GOTERM_BP_FAT	GO:0042440 pigment metabolic process	6	0.295858	0.029473	210	66	7937	3.435931	1.000000	0.647867	38.515944
GOTERM_BP_FAT	GO:0007517 muscle organ development	9	0.443787	0.031296	210	140	7937	2.429694	1.000000	0.660180	40.366314
SP_PIR_KEYWORDS	gpi-anchor	3	0.147929	0.031317	336	11	12980	10.535714	0.998530	0.557508	32.995872
GOTERM_BP_FAT	GO:0042430 indole and derivative metabolic process	3	0.147929	0.032459	210	11	7937	10.307792	1.000000	0.664000	41.519836
GOTERM_BP_FAT	GO:0042434 indole derivative metabolic process	3	0.147929	0.032459	210	11	7937	10.307792	1.000000	0.664000	41.519836
INTERPRO	IPR000219:Dbl homology (DH) domain	4	0.197239	0.033154	304	24	10196	5.589912	1.000000	0.947541	39.304842
GOTERM_MF_FAT	GO:0005089 Rho guanyl-nucleotide exchange factor activity	4	0.197239	0.033192	218	26	7918	5.587862	1.000000	0.850871	38.061855
UP_SEQ_FEATURE	domain:Ig-like C2-type 4	3	0.147929	0.033561	81	11	2975	10.016835	0.999991	0.996983	37.178755
INTERPRO	IPR006076:FAD dependent oxidoreductase	3	0.147929	0.033835	304	10	10196	10.061842	1.000000	0.928153	39.935463
GOTERM_BP_FAT	GO:0035220 wing disc development	14	0.690335	0.034209	210	281	7937	1.883037	1.000000	0.674042	43.216189
SP_PIR_KEYWORDS	hydrolase	50	2.465483	0.035977	336	1470	12980	1.313978	0.999453	0.565945	36.941309
GOTERM_BP_FAT	GO:0007409 axonogenesis	11	0.542406	0.036023	210	198	7937	2.099735	1.000000	0.683994	44.925543
GOTERM_BP_FAT	GO:0035023 regulation of Rho protein signal transduction	4	0.197239	0.036350	210	28	7937	5.399320	1.000000	0.678347	45.228309
GOTERM_BP_FAT	GO:0007167 enzyme linked receptor protein signaling pathway	9	0.443787	0.037398	210	145	7937	2.345911	1.000000	0.680129	46.189401
GOTERM_MF_FAT	GO:0003700 transcription factor activity	18	0.887574	0.038122	218	389	7918	1.680668	1.000000	0.857396	42.396393
GOTERM_BP_FAT	GO:0048812 neuron projection morphogenesis	14	0.690335	0.038501	210	286	7937	1.850117	1.000000	0.682352	47.182977
GOTERM_BP_FAT	GO:0035120 post-embryonic appendage morphogenesis	12	0.591716	0.039290	210	229	7937	1.980536	1.000000	0.681510	47.882741
GOTERM_BP_FAT	GO:0031175 neuron projection development	14	0.690335	0.039577	210	287	7937	1.843670	1.000000	0.676030	48.136041
GOTERM_BP_FAT	GO:0048666 neuron development	16	0.788955	0.040192	210	347	7937	1.742720	1.000000	0.673746	48.672978
GOTERM_BP_FAT	GO:0048667 cell morphogenesis involved in neuron differentiation	14	0.690335	0.040532	210	288	7937	1.837269	1.000000	0.669028	48.967972
UP_SEQ_FEATURE	disulfide bond	13	0.641026	0.040533	81	258	2975	1.850656	0.999999	0.990808	43.076842
UP_SEQ_FEATURE	compositionally biased region:Poly-Gln	7	0.345168	0.040708	81	95	2975	2.706303	0.999999	0.970771	43.218439
GOTERM_BP_FAT	GO:0030182 neuron differentiation	18	0.887574	0.041270	210	409	7937	1.663360	1.000000	0.668061	49.601967
GOTERM_BP_FAT	GO:0009124 nucleoside monophosphate biosynthetic process	5	0.246548	0.041947	210	50	7937	3.779524	1.000000	0.666601	50.177835
GOTERM_BP_FAT	GO:0007423 sensory organ development	18	0.887574	0.042075	210	410	7937	1.659303	1.000000	0.660339	50.286073
GOTERM_BP_FAT	GO:0000902 cell morphogenesis	19	0.936884	0.043002	210	442	7937	1.624682	1.000000	0.661243	51.062280

GOTERM_BP_FAT	GO:0009064 glutamine family amino acid metabolic process	4	0.197239	0.043368	210	30	7937	5.039365	1.000000	0.657303	51.366124
UP_SEQ_FEATURE	compositionally biased region:Poly-Thr	4	0.197239	0.045143	81	30	2975	4.897119	1.000000	0.956768	46.690695
GOTERM_BP_FAT	GO:0009165 nucleotide biosynthetic process	9	0.443787	0.045721	210	151	7937	2.252696	1.000000	0.670131	53.274789
KEGG_PATHWAY	dme00020:Citrate cycle (TCA cycle)	5	0.246548	0.045796	65	44	2054	3.590909	0.934055	0.743203	37.274827
UP_SEQ_FEATURE	domain:Ig-like C2-type 3	3	0.147929	0.045977	81	13	2975	8.475783	1.000000	0.930550	47.321154
GOTERM_BP_FAT	GO:0048747 muscle fiber development	4	0.197239	0.047113	210	31	7937	4.876805	1.000000	0.674525	54.370540
PIR_SUPERFAMILY	PIRSF017866:PIRSF017866	3	0.147929	0.047383	94	10	2596	8.285106	0.993580	0.993580	41.885811
GOTERM_MF_FAT	GO:0004623 phospholipase A2 activity	3	0.147929	0.047811	218	13	7918	8.381793	1.000000	0.890246	50.105514
GOTERM_BP_FAT	GO:0046942 carboxylic acid transport	5	0.246548	0.050227	210	53	7937	3.565588	1.000000	0.691739	56.735301
GOTERM_BP_FAT	GO:0015849 organic acid transport	5	0.246548	0.050227	210	53	7937	3.565588	1.000000	0.691739	56.735301
GOTERM_BP_FAT	GO:0035114 imaginal disc-derived appendage morphogenesis	12	0.591716	0.050716	210	239	7937	1.897669	1.000000	0.688807	57.095937
GOTERM_BP_FAT	GO:0007267 cell-cell signaling	11	0.542406	0.051321	210	211	7937	1.970368	1.000000	0.686798	57.538304
GOTERM_BP_FAT	GO:0009152 purine ribonucleotide biosynthetic process	7	0.345168	0.051987	210	102	7937	2.593791	1.000000	0.685293	58.020732
UP_SEQ_FEATURE	metal ion-binding site:Zinc 2	3	0.147929	0.052722	81	14	2975	7.870370	1.000000	0.927978	52.173232
GOTERM_BP_FAT	GO:0009123 nucleoside monophosphate metabolic process	5	0.246548	0.053172	210	54	7937	3.499559	1.000000	0.687498	58.865852
SP_PIR_KEYWORDS	ank repeat	6	0.295858	0.053273	336	79	12980	2.933996	0.999987	0.674456	49.789010
GOTERM_MF_FAT	GO:0004345 glucose-6-phosphate dehydrogenase activity	2	0.098619	0.054064	218	2	7918	36.321101	1.000000	0.897593	54.559597
GOTERM_BP_FAT	GO:0035107 appendage morphogenesis	12	0.591716	0.054391	210	242	7937	1.874144	1.000000	0.689859	59.718430
INTERPRO	IPR013816:ATP-grasp fold, subdomain 2	3	0.147929	0.055327	304	13	10196	7.739879	1.000000	0.979148	56.953317
GOTERM_BP_FAT	GO:0048737 imaginal disc-derived appendage development	12	0.591716	0.055878	210	243	7937	1.866432	1.000000	0.693924	60.735519
SP_PIR_KEYWORDS	calcium transport	3	0.147929	0.055888	336	15	12980	7.726190	0.999992	0.657603	51.506816
GOTERM_BP_FAT	GO:0000904 cell morphogenesis involved in differentiation	14	0.690335	0.056588	210	303	7937	1.746315	1.000000	0.692748	61.213465
SMART	SM00409:IG	7	0.345168	0.056623	138	97	4824	2.522636	0.998773	0.964973	48.519159
GOTERM_BP_FAT	GO:0034404 nucleobase, nucleoside and nucleotide biosynthetic process	9	0.443787	0.056868	210	158	7937	2.152893	1.000000	0.688785	61.400147
GOTERM_BP_FAT	GO:0034654 nucleobase, nucleoside, nucleotide and nucleic acid biosynthetic process	9	0.443787	0.056868	210	158	7937	2.152893	1.000000	0.688785	61.400147
GOTERM_BP_FAT	GO:0030030 cell projection organization	16	0.788955	0.057754	210	365	7937	1.656778	1.000000	0.688849	61.985533
INTERPRO	IPR001282:Glucose-6-phosphate dehydrogenase	2	0.098619	0.058555	304	2	10196	33.539474	1.000000	0.975097	59.081090
UP_SEQ_FEATURE	compositionally biased region:Poly-Ala	7	0.345168	0.058830	81	104	2975	2.472104	1.000000	0.923986	56.205890
GOTERM_BP_FAT	GO:0006928 cell motion	14	0.690335	0.059055	210	305	7937	1.734863	1.000000	0.691555	62.829898
INTERPRO	IPR013783:Immunoglobulin-like fold	8	0.394477	0.059554	304	117	10196	2.293297	1.000000	0.967161	59.719656
GOTERM_BP_FAT	GO:0048736 appendage development	12	0.591716	0.059788	210	246	7937	1.843670	1.000000	0.690628	63.297515
SMART	SM00233:PH	6	0.295858	0.060907	138	75	4824	2.796522	0.999273	0.910086	51.120322
GOTERM_BP_FAT	GO:0007268 synaptic transmission	10	0.493097	0.061496	210	189	7937	1.999748	1.000000	0.695717	64.366985
GOTERM_CC_FAT	GO:0030017 sarcomere	3	0.147929	0.061870	132	15	4786	7.251515	0.999995	0.782338	54.817517
GOTERM_BP_FAT	GO:0007476 imaginal disc-derived wing morphogenesis	11	0.542406	0.062073	210	218	7937	1.907099	1.000000	0.693843	64.721463
SP_PIR_KEYWORDS	transmembrane	36	1.775148	0.062703	336	1039	12980	1.338512	0.999998	0.669195	55.732232
GOTERM_BP_FAT	GO:0009150 purine ribonucleotide metabolic process	7	0.345168	0.062778	210	107	7937	2.472586	1.000000	0.692778	65.149976
GOTERM_CC_FAT	GO:0005576 extracellular region	21	1.035503	0.063006	132	512	4786	1.487127	0.999996	0.748699	55.493510
GOTERM_BP_FAT	GO:0006576 biogenic amine metabolic process	4	0.197239	0.063606	210	35	7937	4.319456	1.000000	0.692471	65.647243
GOTERM_BP_FAT	GO:0006084 acetyl-CoA metabolic process	4	0.197239	0.063606	210	35	7937	4.319456	1.000000	0.692471	65.647243
GOTERM_BP_FAT	GO:0007472 wing disc morphogenesis	11	0.542406	0.065075	210	220	7937	1.889762	1.000000	0.695887	66.513129
GOTERM_BP_FAT	GO:0007411 axon guidance	8	0.394477	0.067405	210	136	7937	2.223249	1.000000	0.703986	67.844399
SP_PIR_KEYWORDS	developmental protein	21	1.035503	0.067528	336	540	12980	1.502315	0.999999	0.667969	58.516355
GOTERM_BP_FAT	GO:0007178 transmembrane receptor protein serine/threonine kinase signaling pathway	4	0.197239	0.068094	210	36	7937	4.199471	1.000000	0.702814	68.228691
GOTERM_MF_FAT	GO:0005509 calcium ion binding	12	0.591716	0.068591	218	242	7918	1.801046	1.000000	0.930786	63.519877
INTERPRO	IPR003599:Immunoglobulin subtype	7	0.345168	0.068643	304	97	10196	2.420374	1.000000	0.973397	65.115476
GOTERM_CC_FAT	GO:0030016 myofibril	3	0.147929	0.069473	132	16	4786	6.798295	0.999999	0.747233	59.167594
GOTERM_BP_FAT	GO:0035239 tube morphogenesis	6	0.295858	0.070141	210	84	7937	2.699660	1.000000	0.708966	69.344336
PIR_SUPERFAMILY	PIRSF000110:G6PD	2	0.098619	0.070379	94	2	2596	27.617021	0.999494	0.977514	55.779667
PIR_SUPERFAMILY	PIRSF000110:glucose-6-phosphate dehydrogenase	2	0.098619	0.070379	94	2	2596	27.617021	0.999494	0.977514	55.779667
GOTERM_MF_FAT	GO:0008238 exopeptidase activity	7	0.345168	0.070568	218	106	7918	2.398563	1.000000	0.921043	64.603537
SP_PIR_KEYWORDS	glycosidase	6	0.295858	0.071415	336	86	12980	2.695183	1.000000	0.662075	60.640427

INTERPRO	IPR001849:Pleckstrin homology	6	0.295858	0.071958	304	75	10196	2.683158	1.000000	0.970271	66.909762
GOTERM_BP_FAT	GO:0009168 purine ribonucleoside monophosphate biosynthetic process	3	0.147929	0.072433	210	17	7937	6.669748	1.000000	0.716117	70.550184
GOTERM_BP_FAT	GO:0009126 purine nucleoside monophosphate metabolic process	3	0.147929	0.072433	210	17	7937	6.669748	1.000000	0.716117	70.550184
GOTERM_BP_FAT	GO:0009167 purine ribonucleoside monophosphate metabolic process	3	0.147929	0.072433	210	17	7937	6.669748	1.000000	0.716117	70.550184
GOTERM_BP_FAT	GO:0009127 purine nucleoside monophosphate biosynthetic process	3	0.147929	0.072433	210	17	7937	6.669748	1.000000	0.716117	70.550184
GOTERM_BP_FAT	GO:0019226 transmission of nerve impulse	10	0.493097	0.073484	210	196	7937	1.928328	1.000000	0.716731	71.087953
GOTERM_BP_FAT	GO:0048598 embryonic morphogenesis	11	0.542406	0.074927	210	226	7937	1.839591	1.000000	0.719280	71.811356
GOTERM_BP_FAT	GO:0006575 cellular amino acid derivative metabolic process	5	0.246548	0.076338	210	61	7937	3.097970	1.000000	0.721599	72.502320
GOTERM_BP_FAT	GO:0018130 heterocycle biosynthetic process	5	0.246548	0.076338	210	61	7937	3.097970	1.000000	0.721599	72.502320
GOTERM_BP_FAT	GO:0015800 acidic amino acid transport	2	0.098619	0.076945	210	3	7937	25.196825	1.000000	0.719995	72.794422
GOTERM_BP_FAT	GO:0015813 L-glutamate transport	2	0.098619	0.076945	210	3	7937	25.196825	1.000000	0.719995	72.794422
GOTERM_BP_FAT	GO:0009173 pyrimidine ribonucleoside monophosphate metabolic process	2	0.098619	0.076945	210	3	7937	25.196825	1.000000	0.719995	72.794422
GOTERM_BP_FAT	GO:0009174 pyrimidine ribonucleoside monophosphate biosynthetic process	2	0.098619	0.076945	210	3	7937	25.196825	1.000000	0.719995	72.794422
GOTERM_BP_FAT	GO:0015674 di-, tri-valent inorganic cation transport	4	0.197239	0.077488	210	38	7937	3.978446	1.000000	0.718124	73.053824
UP_SEQ_FEATURE	domain:HTH La-type RNA-binding	2	0.098619	0.078548	81	3	2975	24.485597	1.000000	0.954516	67.175754
GOTERM_BP_FAT	GO:0048858 cell projection morphogenesis	14	0.690335	0.079537	210	320	7937	1.653542	1.000000	0.723385	74.010407
GOTERM_MF_FAT	GO:0005313 L-glutamate transmembrane transporter activity	2	0.098619	0.079995	218	3	7918	24.214067	1.000000	0.931840	69.371174
GOTERM_MF_FAT	GO:0030291 protein serine/threonine kinase inhibitor activity	2	0.098619	0.079995	218	3	7918	24.214067	1.000000	0.931840	69.371174
GOTERM_MF_FAT	GO:0015172 acidic amino acid transmembrane transporter activity	2	0.098619	0.079995	218	3	7918	24.214067	1.000000	0.931840	69.371174
GOTERM_BP_FAT	GO:0042401 biogenic amine biosynthetic process	3	0.147929	0.080117	210	18	7937	6.299206	1.000000	0.721714	74.275246
GOTERM_BP_FAT	GO:0006032 chitin catabolic process	3	0.147929	0.080117	210	18	7937	6.299206	1.000000	0.721714	74.275246
GOTERM_MF_FAT	GO:0005085 guanyl-nucleotide exchange factor activity	5	0.246548	0.081586	218	60	7918	3.026758	1.000000	0.922608	70.114196
SMART	SM00680:CLIP	3	0.147929	0.082471	138	17	4824	6.168798	0.999950	0.915800	62.484854
SMART	SM00636:Glyco_18	3	0.147929	0.082471	138	17	4824	6.168798	0.999950	0.915800	62.484854
SP_PIR_KEYWORDS	carbohydrate metabolism	3	0.147929	0.085134	336	19	12980	6.099624	1.000000	0.703595	67.363908
GOTERM_BP_FAT	GO:0045165 cell fate commitment	11	0.542406	0.085494	210	232	7937	1.792016	1.000000	0.741385	76.614097
GOTERM_CC_FAT	GO:0044449 contractile fiber part	3	0.147929	0.085522	132	18	4786	6.042929	1.000000	0.788249	67.113521
GOTERM_CC_FAT	GO:0031225 anchored to membrane	3	0.147929	0.085522	132	18	4786	6.042929	1.000000	0.788249	67.113521
SMART	SM00248:ANK	6	0.295858	0.085928	138	83	4824	2.526977	0.999967	0.873366	64.063475
INTERPRO	IPR013625:Tensin phosphotyrosine-binding domain	2	0.098619	0.086538	304	3	10196	22.359649	1.000000	0.980874	73.826783
GOTERM_BP_FAT	GO:0046394 carboxylic acid biosynthetic process	5	0.246548	0.087589	210	64	7937	2.952753	1.000000	0.746049	77.470217
GOTERM_BP_FAT	GO:0016053 organic acid biosynthetic process	5	0.246548	0.087589	210	64	7937	2.952753	1.000000	0.746049	77.470217
UP_SEQ_FEATURE	glycosylation site:N-linked (GlcNAc...)	17	0.838264	0.088033	81	419	2975	1.490174	1.000000	0.956420	71.489643
GOTERM_BP_FAT	GO:0009072 aromatic amino acid family metabolic process	3	0.147929	0.088039	210	19	7937	5.967669	1.000000	0.743795	77.650147
GOTERM_BP_FAT	GO:0032989 cellular component morphogenesis	20	0.986193	0.088282	210	518	7937	1.459276	1.000000	0.740715	77.746606
SP_PIR_KEYWORDS	Aminopeptidase	4	0.197239	0.088590	336	41	12980	3.768873	1.000000	0.695327	68.881873
INTERPRO	IPR013098:Immunoglobulin I-set	5	0.246548	0.088769	304	57	10196	2.942059	1.000000	0.977466	74.757832
INTERPRO	IPR011583:Chitinase II	3	0.147929	0.089316	304	17	10196	5.918731	1.000000	0.972087	74.981332
INTERPRO	IPR001223:Glycoside hydrolase, family 18, catalytic domain	3	0.147929	0.089316	304	17	10196	5.918731	1.000000	0.972087	74.981332
INTERPRO	IPR006604:Disulphide knot CLIP	3	0.147929	0.089316	304	17	10196	5.918731	1.000000	0.972087	74.981332
UP_SEQ_FEATURE	domain:Ig-like C2-type 2	3	0.147929	0.090936	81	19	2975	5.799220	1.000000	0.947497	72.701238
UP_SEQ_FEATURE	domain:Ig-like C2-type 1	3	0.147929	0.090936	81	19	2975	5.799220	1.000000	0.947497	72.701238
GOTERM_BP_FAT	GO:0007498 mesoderm development	6	0.295858	0.091541	210	91	7937	2.491994	1.000000	0.749928	79.005194
SP_PIR_KEYWORDS	duplication	3	0.147929	0.093035	336	20	12980	5.794643	1.000000	0.691972	70.738868
GOTERM_MF_FAT	GO:0004568 chitinase activity	3	0.147929	0.094250	218	19	7918	5.734911	1.000000	0.938600	75.459563
SP_PIR_KEYWORDS	transport	18	0.887574	0.094536	336	465	12980	1.495392	1.000000	0.677292	71.342461
GOTERM_MF_FAT	GO:0003702 RNA polymerase II transcription factor activity	12	0.591716	0.095258	218	257	7918	1.695927	1.000000	0.929753	75.844117
GOTERM_BP_FAT	GO:0035051 cardiac cell differentiation	3	0.147929	0.096183	210	20	7937	5.669286	1.000000	0.763873	80.683217
GOTERM_BP_FAT	GO:0007390 germ-band shortening	3	0.147929	0.096183	210	20	7937	5.669286	1.000000	0.763873	80.683217
SP_PIR_KEYWORDS	glycoprotein	17	0.838264	0.096553	336	435	12980	1.509715	1.000000	0.665641	72.135521

GOTERM_BP_FAT	GO:0032990 cell part morphogenesis	14	0.690335	0.096811	210	331	7937	1.598590	1.000000	0.762355	80.900307
GOTERM_BP_FAT	GO:0007442 hindgut morphogenesis	4	0.197239	0.097851	210	42	7937	3.599546	1.000000	0.762379	81.254539
INTERPRO	IPR007110:Immunoglobulin-like	8	0.394477	0.098264	304	132	10196	2.032695	1.000000	0.975854	78.384927
INTERPRO	IPR011022:Arrestin-like, C-terminal	3	0.147929	0.098577	304	18	10196	5.589912	1.000000	0.970653	78.495741
INTERPRO	IPR011021:Arrestin-like, N-terminal	3	0.147929	0.098577	304	18	10196	5.589912	1.000000	0.970653	78.495741
GOTERM_BP_FAT	GO:0001654 eye development	14	0.690335	0.098751	210	332	7937	1.593775	1.000000	0.761899	81.556190
SP_PIR_KEYWORDS	glucose metabolism	2	0.098619	0.099318	336	4	12980	19.315476	1.000000	0.657741	73.190059

Table 8: Architecture 8

Category	Term	Count	%	PValue	List Total	Pop Hits	Pop Total	Fold Enrichment	Bonferroni	Benjamini	FDR
UP_SEQ_FEATURE	glycosylation site:N-linked (GlcNAc...)	38	1.305842	4.939275e-09	101	419	2975	2.671377	2.479513e-06	2.479513e-06	7.116753e-06
SP_PIR_KEYWORDS	glycoprotein	40	1.374570	2.179022e-08	437	435	12980	2.731266	4.074763e-06	4.074763e-06	2.701100e-05
GOTERM_CC_FAT	GO:0005578 proteinaceous extracellular matrix	13	0.446735	1.095742e-07	172	50	4786	7.234651	2.092846e-05	2.092846e-05	1.363039e-04
GOTERM_CC_FAT	GO:0031012 extracellular matrix	13	0.446735	2.192732e-07	172	53	4786	6.825143	4.188032e-05	2.094038e-05	2.727628e-04
GOTERM_BP_FAT	GO:0007155 cell adhesion	22	0.756014	3.263917e-07	268	174	7937	3.744510	4.516242e-04	4.516242e-04	5.370297e-04
GOTERM_CC_FAT	GO:0005886 plasma membrane	55	1.890034	3.601885e-07	172	781	4786	1.959548	6.879364e-05	2.293174e-05	4.480526e-04
GOTERM_BP_FAT	GO:0006928 cell motion	29	0.996564	1.133735e-06	268	305	7937	2.815916	0.001568	7.842373e-04	0.001865
GOTERM_BP_FAT	GO:0022610 biological adhesion	22	0.756014	1.195356e-06	268	188	7937	3.465664	0.001653	5.513060e-04	0.001967
GOTERM_CC_FAT	GO:0005604 basement membrane	7	0.240550	1.473982e-06	172	12	4786	16.231589	2.814912e-04	7.038022e-05	0.001834
SP_PIR_KEYWORDS	extracellular matrix	8	0.274914	1.632383e-06	437	19	12980	12.506323	3.052093e-04	1.526163e-04	0.002023
INTERPRO	IPR003598: Immunoglobulin subtype 2	16	0.549828	2.098090e-06	386	94	10196	4.496086	0.001300	0.001300	0.003113
GOTERM_CC_FAT	GO:0044420 extracellular matrix part	7	0.240550	2.656628e-06	172	13	4786	14.983005	5.072879e-04	1.014782e-04	0.003305
GOTERM_BP_FAT	GO:0007411 axon guidance	18	0.618557	2.748144e-06	268	136	7937	3.919721	0.003796	9.504073e-04	0.004522
GOTERM_BP_FAT	GO:0007409 axonogenesis	22	0.756014	2.786525e-06	268	198	7937	3.290630	0.003849	7.710138e-04	0.004585
SP_PIR_KEYWORDS	oxidoreductase	41	1.408935	5.635158e-06	437	561	12980	2.170772	0.001053	3.511975e-04	0.006985
SMART	SM00408:IGc2	16	0.549828	6.309797e-06	203	94	4824	4.044859	8.640714e-04	8.640714e-04	0.007416
INTERPRO	IPR013098: Immunoglobulin I-set	12	0.412371	7.442998e-06	386	57	10196	5.560949	0.004604	0.002305	0.011041
INTERPRO	IPR013783: Immunoglobulin-like fold	17	0.584192	7.680582e-06	386	117	10196	3.838005	0.004751	0.001586	0.011394
INTERPRO	IPR007110: Immunoglobulin-like	18	0.618557	9.106417e-06	386	132	10196	3.601978	0.005630	0.001411	0.013509
GOTERM_BP_FAT	GO:0030030 cell projection organization	30	1.030928	1.295800e-05	268	365	7937	2.434165	0.017774	0.002985	0.021318
SP_PIR_KEYWORDS	basement membrane	5	0.171821	1.785303e-05	437	6	12980	24.752098	0.003333	8.342884e-04	0.022128
INTERPRO	IPR013032:EGF-like region, conserved site	15	0.515464	1.930587e-05	386	99	10196	4.002198	0.011898	0.002391	0.028637
GOTERM_BP_FAT	GO:0055114 oxidation reduction	41	1.408935	4.199762e-05	268	620	7937	1.958456	0.056469	0.008269	0.069079
INTERPRO	IPR003599: Immunoglobulin subtype	14	0.481100	6.781059e-05	386	97	10196	3.812403	0.041172	0.006983	0.100553
GOTERM_CC_FAT	GO:0030054 cell junction	16	0.549828	7.131589e-05	172	134	4786	3.322457	0.013529	0.002268	0.088677
GOTERM_BP_FAT	GO:0000902 cell morphogenesis	32	1.099656	7.301335e-05	268	442	7937	2.144121	0.096116	0.012552	0.120065
GOTERM_CC_FAT	GO:0044459 plasma membrane part	31	1.065292	7.688764e-05	172	405	4786	2.129859	0.014579	0.002096	0.095602
GOTERM_BP_FAT	GO:0000904 cell morphogenesis involved in differentiation	25	0.859107	7.883977e-05	268	303	7937	2.443537	0.103376	0.012051	0.129641
GOTERM_CC_FAT	GO:0044421 extracellular region part	17	0.584192	7.959414e-05	172	151	4786	3.132681	0.015088	0.001899	0.098965
GOTERM_BP_FAT	GO:0048812 neuron projection morphogenesis	24	0.824742	8.730789e-05	268	286	7937	2.485231	0.113824	0.012011	0.143556
GOTERM_BP_FAT	GO:0031175 neuron projection development	24	0.824742	9.359465e-05	268	287	7937	2.476572	0.121501	0.011707	0.153885
GOTERM_BP_FAT	GO:0048667 cell morphogenesis involved in neuron differentiation	24	0.824742	9.840750e-05	268	288	7937	2.467973	0.127334	0.011286	0.161792
GOTERM_BP_FAT	GO:0007444 imaginal disc development	30	1.030928	1.182347e-04	268	412	7937	2.156481	0.150958	0.012509	0.194361
GOTERM_BP_FAT	GO:0032990 cell part morphogenesis	26	0.893471	1.199857e-04	268	331	7937	2.326307	0.153013	0.011792	0.197237
SP_PIR_KEYWORDS	cell binding	4	0.137457	1.468234e-04	437	4	12980	29.702517	0.027084	0.005477	0.181849
SMART	SM00409:IG	14	0.481100	1.720585e-04	203	97	4824	3.429790	0.023298	0.011718	0.202053
GOTERM_BP_FAT	GO:0048858 cell projection morphogenesis	25	0.859107	1.835440e-04	268	320	7937	2.313724	0.224346	0.016794	0.301568
GOTERM_BP_FAT	GO:0007424 open tracheal system development	16	0.549828	1.858947e-04	268	153	7937	3.097064	0.226865	0.015953	0.305424
GOTERM_BP_FAT	GO:0060541 respiratory system development	16	0.549828	1.858947e-04	268	153	7937	3.097064	0.226865	0.015953	0.305424
INTERPRO	IPR000034:Laminin B type IV	4	0.137457	2.077338e-04	386	4	10196	26.414508	0.120858	0.018233	0.307741
GOTERM_BP_FAT	GO:0007498 mesoderm development	12	0.412371	2.207422e-04	268	91	7937	3.905363	0.263275	0.017812	0.362581
GOTERM_BP_FAT	GO:0032989 cellular component morphogenesis	34	1.168385	2.666560e-04	268	518	7937	1.943886	0.308645	0.020297	0.437842
GOTERM_BP_FAT	GO:0048569 post-embryonic organ development	23	0.790378	4.068492e-04	268	297	7937	2.293470	0.430612	0.029207	0.667312
SP_PIR_KEYWORDS	FAD	7	0.240550	4.149456e-04	437	30	12980	6.930587	0.074676	0.012852	0.513149
GOTERM_BP_FAT	GO:0007398 ectoderm development	10	0.343643	4.172924e-04	268	68	7937	4.355246	0.438786	0.028470	0.684386
GOTERM_CC_FAT	GO:0005605 basal lamina	4	0.137457	4.251252e-04	172	5	4786	22.260465	0.078006	0.008983	0.527547
GOTERM_BP_FAT	GO:0035220 wing disc development	22	0.756014	4.884961e-04	268	281	7937	2.318665	0.491476	0.031689	0.800724
GOTERM_BP_FAT	GO:0048563 post-embryonic organ morphogenesis	22	0.756014	5.630161e-04	268	284	7937	2.294172	0.541334	0.034809	0.922344
GOTERM_BP_FAT	GO:0007560 imaginal disc morphogenesis	22	0.756014	5.630161e-04	268	284	7937	2.294172	0.541334	0.034809	0.922344
GOTERM_BP_FAT	GO:0048666 neuron development	25	0.859107	6.066794e-04	268	347	7937	2.133694	0.568246	0.035859	0.993539
SP_PIR_KEYWORDS	developmental protein	34	1.168385	6.656603e-04	437	540	12980	1.870158	0.117079	0.017631	0.822025

GOTERM_BP_FAT	GO:0046395 carboxylic acid catabolic process	8	0.274914	6.834376e-04	268	45	7937	5.265008	0.611787	0.038658	1.118581
GOTERM_BP_FAT	GO:0016054 organic acid catabolic process	8	0.274914	6.834376e-04	268	45	7937	5.265008	0.611787	0.038658	1.118581
GOTERM_BP_FAT	GO:0035120 post-embryonic appendage morphogenesis	19	0.652921	6.855811e-04	268	229	7937	2.457195	0.612938	0.037255	1.122070
UP_SEQ_FEATURE	disulfide bond	20	0.687285	7.217491e-04	101	258	2975	2.283368	0.304030	0.165752	1.034915
SP_PIR_KEYWORDS	Homeobox	12	0.412371	7.272694e-04	437	104	12980	3.427214	0.127200	0.016862	0.897791
GOTERM_BP_FAT	GO:0016337 cell-cell adhesion	9	0.309278	7.387804e-04	268	59	7937	4.517645	0.640431	0.038577	1.208644
GOTERM_CC_FAT	GO:0005911 cell-cell junction	9	0.309278	7.391912e-04	172	56	4786	4.471968	0.131717	0.014025	0.915633
KEGG_PATHWAY	dme00071:Fatty acid metabolism	8	0.274914	7.518036e-04	89	37	2054	4.989979	0.054134	0.054134	0.784095
INTERPRO	IPR002049:EGF-like, laminin	5	0.171821	7.783315e-04	386	12	10196	11.006045	0.382918	0.058560	1.148498
SP_PIR_KEYWORDS	cell adhesion	8	0.274914	7.917704e-04	437	46	12980	5.165655	0.137672	0.016323	0.977057
SP_PIR_KEYWORDS	Immunoglobulin domain	8	0.274914	9.038909e-04	437	47	12980	5.055748	0.155579	0.016768	1.114704
UP_SEQ_FEATURE	topological domain:Cytoplasmic	26	0.893471	9.088957e-04	101	394	2975	1.943760	0.366486	0.141147	1.301632
GOTERM_BP_FAT	GO:0060429 epithelium development	19	0.652921	9.286847e-04	268	235	7937	2.394459	0.723597	0.046509	1.517103
INTERPRO	IPR008211:Laminin, N-terminal	4	0.137457	9.812588e-04	386	6	10196	17.609672	0.455931	0.065395	1.445907
UP_SEQ_FEATURE	signal peptide	27	0.927835	9.831182e-04	101	419	2975	1.898084	0.389678	0.116127	1.407227
INTERPRO	IPR013151: Immunoglobulin	11	0.378007	9.908683e-04	386	82	10196	3.543410	0.459166	0.059613	1.459970
GOTERM_BP_FAT	GO:0007476 imaginal disc-derived wing morphogenesis	18	0.618557	0.001051	268	218	7937	2.445331	0.766719	0.050655	1.715488
UP_SEQ_FEATURE	topological domain:Extracellular	22	0.756014	0.001065	101	309	2975	2.097151	0.414226	0.101442	1.523331
GOTERM_BP_FAT	GO:0007552 metamorphosis	25	0.859107	0.001093	268	362	7937	2.045281	0.779790	0.050840	1.782841
GOTERM_BP_FAT	GO:0035114 imaginal disc-derived appendage morphogenesis	19	0.652921	0.001127	268	239	7937	2.354384	0.789886	0.050675	1.837627
SMART	SM00180:EGF_Lam	5	0.171821	0.001133	203	12	4824	9.901478	0.143894	0.050469	1.324100
SP_PIR_KEYWORDS	signal	28	0.962199	0.001154	437	425	12980	1.956872	0.194254	0.019444	1.421541
GOTERM_BP_FAT	GO:0007472 wing disc morphogenesis	18	0.618557	0.001163	268	220	7937	2.423100	0.800336	0.050644	1.897138
SP_PIR_KEYWORDS	laminin egf-like domain	4	0.137457	0.001191	437	7	12980	16.972867	0.199758	0.018399	1.466319
GOTERM_BP_FAT	GO:0035107 appendage morphogenesis	19	0.652921	0.001299	268	242	7937	2.325197	0.834538	0.054668	2.116034
SMART	SM00136:LamNT	4	0.137457	0.001318	203	6	4824	15.842365	0.165250	0.044151	1.537738
GOTERM_BP_FAT	GO:0048737 imaginal disc-derived appendage development	19	0.652921	0.001361	268	243	7937	2.315629	0.848253	0.055536	2.216671
GOTERM_BP_FAT	GO:0048707 instar larval or pupal morphogenesis	24	0.824742	0.001399	268	347	7937	2.048346	0.855890	0.055383	2.276687
GOTERM_BP_FAT	GO:0002009 morphogenesis of an epithelium	18	0.618557	0.001488	268	225	7937	2.369254	0.872689	0.057189	2.420572
GOTERM_MF_FAT	GO:0009055 electron carrier activity	18	0.618557	0.001547	299	202	7918	2.359747	0.543792	0.543792	2.208701
INTERPRO	IPR013106: Immunoglobulin V-set	8	0.274914	0.001553	386	46	10196	4.593827	0.618492	0.083875	2.279404
GOTERM_BP_FAT	GO:0048736 appendage development	19	0.652921	0.001564	268	246	7937	2.287389	0.885323	0.058383	2.541744
SP_PIR_KEYWORDS	disulfide bond	20	0.687285	0.001564	437	265	12980	2.241699	0.253702	0.022259	1.921109
INTERPRO	IPR001356:Homeobox	12	0.412371	0.001573	386	102	10196	3.107589	0.623165	0.078110	2.308217
GOTERM_MF_FAT	GO:0048037 cofactor binding	17	0.584192	0.001593	299	185	7918	2.433445	0.554378	0.332451	2.274017
GOTERM_BP_FAT	GO:0048729 tissue morphogenesis	19	0.652921	0.001636	268	247	7937	2.278129	0.896318	0.059416	2.658451
GOTERM_BP_FAT	GO:0009886 post-embryonic morphogenesis	24	0.824742	0.001753	268	353	7937	2.013530	0.911783	0.061895	2.845191
GOTERM_MF_FAT	GO:0050660 FAD binding	9	0.309278	0.001900	299	61	7918	3.907122	0.618727	0.274877	2.706795
GOTERM_CC_FAT	GO:0005576 extracellular region	32	1.099656	0.002018	172	512	4786	1.739099	0.320137	0.034470	2.481732
GOTERM_BP_FAT	GO:0007507 heart development	9	0.309278	0.002086	268	69	7937	3.862914	0.944415	0.071420	3.377218
GOTERM_MF_FAT	GO:0050662 coenzyme binding	14	0.481100	0.002157	299	139	7918	2.667212	0.665377	0.239430	3.067485
GOTERM_BP_FAT	GO:0030182 neuron differentiation	26	0.893471	0.002687	268	409	7937	1.882659	0.975869	0.088904	4.330974
GOTERM_BP_FAT	GO:0009791 post-embryonic development	27	0.927835	0.002893	268	434	7937	1.842450	0.981860	0.093167	4.655016
SP_PIR_KEYWORDS	Flavoprotein	7	0.240550	0.002949	437	43	12980	4.835293	0.424372	0.038682	3.594855
GOTERM_BP_FAT	GO:0009064 glutamine family amino acid metabolic process	6	0.206186	0.002965	268	30	7937	5.923134	0.983580	0.093205	4.767837
INTERPRO	IPR013525:ABC-2 type transporter	5	0.171821	0.003221	386	17	10196	7.768973	0.864728	0.142627	4.674004
UP_SEQ_FEATURE	domain:Laminin EGF-like 4	3	0.103093	0.003283	101	3	2975	29.455446	0.808105	0.240530	4.627673
UP_SEQ_FEATURE	domain:Laminin EGF-like 7	3	0.103093	0.003283	101	3	2975	29.455446	0.808105	0.240530	4.627673
UP_SEQ_FEATURE	domain:Laminin EGF-like 8	3	0.103093	0.003283	101	3	2975	29.455446	0.808105	0.240530	4.627673
UP_SEQ_FEATURE	domain:Laminin EGF-like 10	3	0.103093	0.003283	101	3	2975	29.455446	0.808105	0.240530	4.627673
UP_SEQ_FEATURE	domain:Laminin EGF-like 9	3	0.103093	0.003283	101	3	2975	29.455446	0.808105	0.240530	4.627673
UP_SEQ_FEATURE	domain:Laminin EGF-like 6	3	0.103093	0.003283	101	3	2975	29.455446	0.808105	0.240530	4.627673
SMART	SM00389:HOX	12	0.412371	0.003317	203	102	4824	2.795711	0.365661	0.087014	3.829957
KEGG_PATHWAY	dme00260:Glycine, serine and threonine metabolism	6	0.206186	0.003469	89	25	2054	5.538876	0.226758	0.120658	3.571998
INTERPRO	IPR017970:Homeobox, conserved site	11	0.378007	0.003534	386	97	10196	2.995460	0.888671	0.145129	5.117312

GOTERM_BP_FAT	GO:0002165 instar larval or pupal development	26	0.893471	0.003644	268	418	7937	1.842123	0.993609	0.110866	5.830131
GOTERM_BP_FAT	GO:0007445 determination of imaginal disc primordium	4	0.137457	0.003789	268	10	7937	11.846269	0.994776	0.112564	6.055563
GOTERM_BP_FAT	GO:0006536 glutamate metabolic process	4	0.137457	0.003789	268	10	7937	11.846269	0.994776	0.112564	6.055563
GOTERM_BP_FAT	GO:0035225 determination of genital disc primordium	4	0.137457	0.003789	268	10	7937	11.846269	0.994776	0.112564	6.055563
INTERPRO	IPR018031:Laminin B	3	0.103093	0.004160	386	3	10196	26.414508	0.924566	0.158273	5.996907
GOTERM_BP_FAT	GO:0009063 cellular amino acid catabolic process	6	0.206186	0.004549	268	33	7937	5.384668	0.998181	0.130829	7.226766
GOTERM_BP_FAT	GO:0007447 imaginal disc pattern formation	10	0.343643	0.004851	268	96	7937	3.084966	0.998806	0.136109	7.689416
GOTERM_BP_FAT	GO:0045165 cell fate commitment	17	0.584192	0.005033	268	232	7937	2.170114	0.999072	0.138061	7.966271
SMART	SM00281:LamB	3	0.103093	0.005091	203	3	4824	23.763547	0.503012	0.109998	5.822435
GOTERM_BP_FAT	GO:0007389 pattern specification process	28	0.962199	0.005727	268	480	7937	1.727581	0.999647	0.152615	9.017125
GOTERM_CC_FAT	GO:0005918 septate junction	5	0.171821	0.005847	172	21	4786	6.625138	0.673766	0.089121	7.035485
GOTERM_MF_FAT	GO:0043565 sequence-specific DNA binding	18	0.618557	0.005937	299	230	7918	2.072473	0.951153	0.453276	8.233090
SP_PIR_KEYWORDS	ionic channel	11	0.378007	0.005996	437	117	12980	2.792544	0.675225	0.072233	7.183839
GOTERM_MF_FAT	GO:0020037 heme binding	13	0.446735	0.006381	299	140	7918	2.459006	0.961049	0.417781	8.822447
GOTERM_MF_FAT	GO:0046906 tetrapyrrole binding	13	0.446735	0.006381	299	140	7918	2.459006	0.961049	0.417781	8.822447
UP_SEQ_FEATURE	DNA-binding region:Homeobox	7	0.240550	0.006559	101	51	2975	4.042904	0.963248	0.376207	9.046279
INTERPRO	IPR012287:Homeodomain-related	11	0.378007	0.006642	386	106	10196	2.741128	0.983950	0.227600	9.414201
GOTERM_BP_FAT	GO:0009310 amine catabolic process	6	0.206186	0.006658	268	36	7937	4.935945	0.999903	0.171940	10.408173
GOTERM_BP_FAT	GO:0009954 proximal/distal pattern formation	5	0.171821	0.006691	268	23	7937	6.438189	0.999908	0.169587	10.458030
GOTERM_BP_FAT	GO:0035218 leg disc development	7	0.240550	0.006927	268	51	7937	4.064896	0.999934	0.171909	10.807191
INTERPRO	IPR015590:Aldehyde dehydrogenase	4	0.137457	0.007029	386	11	10196	9.605276	0.987394	0.226842	9.936305
GOTERM_BP_FAT	GO:0035272 exocrine system development	12	0.412371	0.007053	268	139	7937	2.556749	0.999944	0.171693	10.992620
GOTERM_BP_FAT	GO:0007431 salivary gland development	12	0.412371	0.007053	268	139	7937	2.556749	0.999944	0.171693	10.992620
SP_PIR_KEYWORDS	dna-binding	25	0.859107	0.007330	437	417	12980	1.780726	0.747353	0.082392	8.716211
GOTERM_BP_FAT	GO:0007517 muscle organ development	12	0.412371	0.007432	268	140	7937	2.538486	0.999967	0.177008	11.551171
INTERPRO	IPR000572: Oxidoreductase, molybdopterin binding	3	0.103093	0.008112	386	4	10196	19.810881	0.993590	0.244634	11.382116
GOTERM_MF_FAT	GO:0003702 RNA polymerase II transcription factor activity	19	0.652921	0.008127	299	257	7918	1.957784	0.984035	0.446254	11.107601
GOTERM_CC_FAT	GO:0031224 intrinsic to membrane	65	2.233677	0.008647	172	1382	4786	1.308729	0.809620	0.119790	10.239919
INTERPRO	IPR011701:Major facilitator superfamily MFS-1	11	0.378007	0.009110	386	111	10196	2.617654	0.996567	0.258175	12.695995
INTERPRO	IPR016162:Aldehyde dehydrogenase, N-terminal	4	0.137457	0.009113	386	12	10196	8.804836	0.996573	0.247084	12.699780
GOTERM_MF_FAT	GO:0005506 iron ion binding	19	0.652921	0.009114	299	260	7918	1.935194	0.990363	0.440250	12.375442
SP_PIR_KEYWORDS	membrane	54	1.855670	0.009221	437	1139	12980	1.408197	0.823121	0.096879	10.848281
UP_SEQ_FEATURE	domain:EGF-like 1	4	0.137457	0.010243	101	14	2975	8.415842	0.994306	0.475880	13.786275
GOTERM_BP_FAT	GO:0044242 cellular lipid catabolic process	5	0.171821	0.010440	268	26	7937	5.695321	1.000000	0.235846	15.859507
GOTERM_BP_FAT	GO:0035088 establishment or maintenance of apical/basal cell polarity	5	0.171821	0.010440	268	26	7937	5.695321	1.000000	0.235846	15.859507
UP_SEQ_FEATURE	domain:Laminin N-terminal	3	0.103093	0.010470	101	5	2975	17.673267	0.994925	0.444036	14.070838
UP_SEQ_FEATURE	domain:Laminin EGF-like 1	3	0.103093	0.010470	101	5	2975	17.673267	0.994925	0.444036	14.070838
UP_SEQ_FEATURE	domain:Laminin EGF-like 2	3	0.103093	0.010470	101	5	2975	17.673267	0.994925	0.444036	14.070838
UP_SEQ_FEATURE	domain:Laminin EGF-like 3	3	0.103093	0.010470	101	5	2975	17.673267	0.994925	0.444036	14.070838
GOTERM_BP_FAT	GO:0006537 glutamate biosynthetic process	3	0.103093	0.010541	268	5	7937	17.769403	1.000000	0.234065	16.000344
GOTERM_CC_FAT	GO:0005887 integral to plasma membrane	15	0.515464	0.010654	172	198	4786	2.107999	0.870737	0.135960	12.474987
GOTERM_MF_FAT	GO:0003700 transcription factor activity	25	0.859107	0.011444	299	389	7918	1.701903	0.997078	0.477117	15.301577
SP_PIR_KEYWORDS	cell junction	8	0.274914	0.011948	437	74	12980	3.211083	0.894357	0.117390	13.842718
GOTERM_CC_FAT	GO:0031226 intrinsic to plasma membrane	15	0.515464	0.012068	172	201	4786	2.076536	0.901622	0.143239	14.017577
GOTERM_CC_FAT	GO:0070160 occluding junction	5	0.171821	0.012715	172	26	4786	5.351073	0.913200	0.141664	14.715912
GOTERM_BP_FAT	GO:0045596 negative regulation of cell differentiation	8	0.274914	0.012738	268	75	7937	3.159005	1.000000	0.271539	19.016453
GOTERM_BP_FAT	GO:0035215 genital disc development	5	0.171821	0.013569	268	28	7937	5.288513	1.000000	0.282306	20.130885
GOTERM_BP_FAT	GO:0007156 homophilic cell adhesion	5	0.171821	0.013569	268	28	7937	5.288513	1.000000	0.282306	20.130885
UP_SEQ_FEATURE	metal ion-binding site:Iron (heme axial ligand)	9	0.309278	0.014171	101	96	2975	2.761448	0.999227	0.511523	18.587435

INTERPRO	IPR006091:Acyl-CoA oxidase/dehydrogenase, central region	4	0.137457	0.014257	386	14	10196	7.547002	0.999864	0.345548	19.187279
INTERPRO	IPR013786:Acyl-CoA dehydrogenase/oxidase, N-terminal	4	0.137457	0.014257	386	14	10196	7.547002	0.999864	0.345548	19.187279
INTERPRO	IPR013764:Acyl-CoA oxidase/dehydrogenase, type1/2, C-terminal	4	0.137457	0.014257	386	14	10196	7.547002	0.999864	0.345548	19.187279
GOTERM_CC_FAT	GO:0016021 integral to membrane	63	2.164948	0.014272	172	1360	4786	1.288979	0.935787	0.149136	16.373681
GOTERM_CC_FAT	GO:0043296 apical junction complex	6	0.206186	0.014525	172	41	4786	4.072036	0.938861	0.143804	16.640445
SP_PIR_KEYWORDS	transmembrane	49	1.683849	0.014755	437	1039	12980	1.400792	0.937941	0.136100	16.828063
GOTERM_BP_FAT	GO:0009069 serine family amino acid metabolic process	4	0.137457	0.015232	268	16	7937	7.403918	1.000000	0.306684	22.318474
GOTERM_BP_FAT	GO:0007479 leg disc proximal/distal pattern formation	4	0.137457	0.015232	268	16	7937	7.403918	1.000000	0.306684	22.318474
GOTERM_BP_FAT	GO:0035223 leg disc pattern formation	4	0.137457	0.015232	268	16	7937	7.403918	1.000000	0.306684	22.318474
UP_SEQ_FEATURE	domain:EGF-like 5	3	0.103093	0.015362	101	6	2975	14.727723	0.999578	0.506635	19.993405
SP_PIR_KEYWORDS	heterotrimer	3	0.103093	0.015437	437	6	12980	14.851259	0.945478	0.135373	17.538835
GOTERM_CC_FAT	GO:0043190 ATP-binding cassette (ABC) transporter complex	6	0.206186	0.016024	172	42	4786	3.975083	0.954288	0.149890	18.204273
INTERPRO	IPR006210:EGF-like	8	0.274914	0.016103	386	70	10196	3.018801	0.999957	0.367132	21.402788
INTERPRO	IPR002172:Low density lipoprotein-receptor, class A, cysteine-rich	6	0.206186	0.016602	386	40	10196	3.962176	0.999969	0.363194	21.992670
INTERPRO	IPR006209:EGF	6	0.206186	0.016602	386	40	10196	3.962176	0.999969	0.363194	21.992670
COG_ONTOLOGY	Lipid metabolism	8	0.274914	0.016807	55	63	1237	2.855988	0.250355	0.250355	11.549670
GOTERM_MF_FAT	GO:0003995 acyl-CoA dehydrogenase activity	4	0.137457	0.017154	299	15	7918	7.061761	0.999845	0.584083	22.093390
GOTERM_BP_FAT	GO:0034329 cell junction assembly	5	0.171821	0.017236	268	30	7937	4.935945	1.000000	0.334918	24.878977
GOTERM_BP_FAT	GO:0003002 regionalization	25	0.859107	0.018141	268	454	7937	1.630819	1.000000	0.344453	26.008391
INTERPRO	IPR000742:EGF-like, type 3	7	0.240550	0.018433	386	56	10196	3.301813	0.999990	0.381611	24.120210
INTERPRO	IPR003439:ABC transporter-like	7	0.240550	0.018433	386	56	10196	3.301813	0.999990	0.381611	24.120210
GOTERM_BP_FAT	GO:0016331 morphogenesis of embryonic epithelium	10	0.343643	0.019557	268	120	7937	2.467973	1.000000	0.361167	27.744997
SP_PIR_KEYWORDS	ion transport	13	0.446735	0.019953	437	182	12980	2.121608	0.976922	0.164288	22.106958
GOTERM_CC_FAT	GO:0016327 apicolateral plasma membrane	6	0.206186	0.021130	172	45	4786	3.710078	0.983078	0.184503	23.330516
GOTERM_BP_FAT	GO:0048732 gland development	12	0.412371	0.021413	268	163	7937	2.180295	1.000000	0.383187	29.963236
GOTERM_BP_FAT	GO:0008544 epidermis development	6	0.206186	0.021846	268	48	7937	3.701959	1.000000	0.384456	30.471771
GOTERM_MF_FAT	GO:0022803 passive transmembrane transporter activity	14	0.481100	0.023990	299	187	7918	1.982580	0.999995	0.673460	29.556675
GOTERM_MF_FAT	GO:0015267 channel activity	14	0.481100	0.023990	299	187	7918	1.982580	0.999995	0.673460	29.556675
UP_SEQ_FEATURE	domain:Ig-like C2-type 2	4	0.137457	0.024147	101	19	2975	6.201146	0.999995	0.640319	29.685245
UP_SEQ_FEATURE	domain:Ig-like C2-type 1	4	0.137457	0.024147	101	19	2975	6.201146	0.999995	0.640319	29.685245
GOTERM_MF_FAT	GO:0016620 oxidoreductase activity, acting on the aldehyde or oxo group of donors, NAD or NADP as acceptor	4	0.137457	0.024257	299	17	7918	6.230966	0.999996	0.645657	29.834046
SMART	SM00192:LDLa	6	0.206186	0.024477	203	40	4824	3.564532	0.966460	0.384304	25.269431
INTERPRO	IPR001791:Laminin G	4	0.137457	0.024500	386	17	10196	6.215178	1.000000	0.459450	30.788071
INTERPRO	IPR012680:Laminin G, subdomain 2	4	0.137457	0.024500	386	17	10196	6.215178	1.000000	0.459450	30.788071
SMART	SM00181:EGF	8	0.274914	0.026137	203	70	4824	2.715834	0.973440	0.364626	26.750550
GOTERM_MF_FAT	GO:0016801 hydrolase activity, acting on ether bonds	3	0.103093	0.026154	299	7	7918	11.349259	0.999999	0.644275	31.777315
SP_PIR_KEYWORDS	alternative splicing	31	1.065292	0.026187	437	616	12980	1.494770	0.993003	0.201928	28.031317
GOTERM_BP_FAT	GO:0035316 non-sensory hair organization	5	0.171821	0.026283	268	34	7937	4.355246	1.000000	0.437845	35.482726
GOTERM_BP_FAT	GO:0035315 hair cell differentiation	5	0.171821	0.026283	268	34	7937	4.355246	1.000000	0.437845	35.482726
SP_PIR_KEYWORDS	Secreted	14	0.481100	0.026888	437	212	12980	1.961487	0.993884	0.198767	28.670795
GOTERM_BP_FAT	GO:0007561 imaginal disc eversion	3	0.103093	0.027608	268	8	7937	11.105877	1.000000	0.449045	36.911809
SP_PIR_KEYWORDS	egf-like domain	6	0.206186	0.027670	437	51	12980	3.494414	0.994738	0.196386	29.378389
KEGG_PATHWAY	dme00330:Arginine and proline metabolism	7	0.240550	0.027900	89	55	2054	2.937283	0.876801	0.502413	25.634080
SP_PIR_KEYWORDS	metal-binding	38	1.305842	0.027970	437	796	12980	1.417959	0.995033	0.191195	29.647785
GOTERM_BP_FAT	GO:0043062 extracellular structure organization	7	0.240550	0.028014	268	69	7937	3.004488	1.000000	0.448892	37.343628
GOTERM_BP_FAT	GO:0007448 anterior/posterior pattern formation, imaginal disc	4	0.137457	0.028097	268	20	7937	5.923134	1.000000	0.444951	37.431773
GOTERM_BP_FAT	GO:0035151 regulation of tube size, open tracheal system	4	0.137457	0.028097	268	20	7937	5.923134	1.000000	0.444951	37.431773

GOTERM_BP_FAT	GO:0045197 establishment or maintenance of epithelial cell apical/basal polarity	4	0.137457	0.028097	268	20	7937	5.923134	1.000000	0.444951	37.431773
KEGG_PATHWAY	dme00903:Limonene and pinene degradation	9	0.309278	0.028208	89	86	2054	2.415208	0.879653	0.411008	25.879999
KEGG_PATHWAY	dme00250:Alanine, aspartate and glutamate metabolism	5	0.171821	0.029316	89	28	2054	4.121188	0.889397	0.356197	26.759946
GOTERM_MF_FAT	GO:0004222 metalloendopeptidase activity	8	0.274914	0.030437	299	80	7918	2.648161	1.000000	0.673517	35.980591
INTERPRO	IPR008957:Fibronectin, type III-like fold	6	0.206186	0.031283	386	47	10196	3.372065	1.000000	0.531354	37.594707
GOTERM_BP_FAT	GO:0007610 behavior	23	0.790378	0.031313	268	429	7937	1.587787	1.000000	0.476652	40.752997
SP_PIR_KEYWORDS	cell membrane	18	0.618557	0.031634	437	308	12980	1.735861	0.997549	0.206416	32.865093
GOTERM_BP_FAT	GO:0016055 Wnt receptor signaling pathway	7	0.240550	0.031679	268	71	7937	2.919855	1.000000	0.475704	41.119958
GOTERM_BP_FAT	GO:0009913 epidermal cell differentiation	5	0.171821	0.031697	268	36	7937	4.113288	1.000000	0.471035	41.137460
GOTERM_BP_FAT	GO:0034330 cell junction organization	5	0.171821	0.031697	268	36	7937	4.113288	1.000000	0.471035	41.137460
SMART	SM00282:LamG	4	0.137457	0.031878	203	17	4824	5.591423	0.988186	0.389307	31.668835
GOTERM_BP_FAT	GO:0007449 proximal/distal pattern formation, imaginal disc	4	0.137457	0.031985	268	21	7937	5.641080	1.000000	0.469361	41.425454
GOTERM_MF_FAT	GO:0008028 monocarboxylic acid transmembrane transporter activity	4	0.137457	0.032713	299	19	7918	5.575075	1.000000	0.675079	38.114701
SP_PIR_KEYWORDS	heme	9	0.309278	0.033301	437	111	12980	2.408312	0.998224	0.209085	34.283605
GOTERM_BP_FAT	GO:0019748 secondary metabolic process	7	0.240550	0.033624	268	72	7937	2.879301	1.000000	0.481822	43.035552
SP_PIR_KEYWORDS	neurogenesis	7	0.240550	0.033686	437	72	12980	2.887745	0.998351	0.204551	34.607371
GOTERM_BP_FAT	GO:0016477 cell migration	12	0.412371	0.033795	268	175	7937	2.030789	1.000000	0.478888	43.201429
GOTERM_MF_FAT	GO:0004467 long-chain-fatty-acid-CoA ligase activity	3	0.103093	0.034017	299	8	7918	9.930602	1.000000	0.666018	39.307886
GOTERM_MF_FAT	GO:0015645 fatty-acid ligase activity	3	0.103093	0.034017	299	8	7918	9.930602	1.000000	0.666018	39.307886
UP_SEQ_FEATURE	domain:EGF-like 4	3	0.103093	0.034521	101	9	2975	9.818482	1.000000	0.742467	39.721136
UP_SEQ_FEATURE	transmembrane region	28	0.962199	0.036045	101	575	2975	1.434352	1.000000	0.731881	41.077058
GOTERM_BP_FAT	GO:0008045 motor axon guidance	4	0.137457	0.036139	268	22	7937	5.384668	1.000000	0.497630	45.427077
GOTERM_BP_FAT	GO:0045610 regulation of hemocyte differentiation	4	0.137457	0.036139	268	22	7937	5.384668	1.000000	0.497630	45.427077
SP_PIR_KEYWORDS	iron	12	0.412371	0.036617	437	177	12980	2.013730	0.999066	0.213804	37.024389
KEGG_PATHWAY	dme00310:Lysine degradation	5	0.171821	0.036779	89	30	2054	3.846442	0.937523	0.370078	32.443896
SP_PIR_KEYWORDS	metalloprotease	5	0.171821	0.037694	437	38	12980	3.908226	0.999242	0.212982	37.891688
GOTERM_BP_FAT	GO:0008354 germ cell migration	5	0.171821	0.037717	268	38	7937	3.896799	1.000000	0.508096	46.878460
GOTERM_BP_FAT	GO:0035152 regulation of tube architecture, open tracheal system	5	0.171821	0.037717	268	38	7937	3.896799	1.000000	0.508096	46.878460
INTERPRO	IPR016160:Aldehyde dehydrogenase, conserved site	4	0.137457	0.037802	386	20	10196	5.282902	1.000000	0.587235	43.542697
UP_SEQ_FEATURE	splice variant	29	0.996564	0.037932	101	604	2975	1.414252	1.000000	0.725872	42.717685
INTERPRO	IPR013320:Concanavalin A-like lectin/glucanase, subgroup	5	0.171821	0.038002	386	34	10196	3.884486	1.000000	0.575937	43.716405
GOTERM_CC_FAT	GO:0042598 vesicular fraction	8	0.274914	0.038844	172	89	4786	2.501176	0.999483	0.302561	38.910554
GOTERM_CC_FAT	GO:0005792 microsome	8	0.274914	0.038844	172	89	4786	2.501176	0.999483	0.302561	38.910554
SP_PIR_KEYWORDS	calcium	11	0.378007	0.040538	437	158	12980	2.067897	0.999564	0.220911	40.128754
GOTERM_BP_FAT	GO:0045471 response to ethanol	4	0.137457	0.040557	268	23	7937	5.150552	1.000000	0.529496	49.399567
GOTERM_BP_FAT	GO:0035150 regulation of tube size	4	0.137457	0.040557	268	23	7937	5.150552	1.000000	0.529496	49.399567
GOTERM_BP_FAT	GO:0007367 segment polarity determination	5	0.171821	0.040957	268	39	7937	3.796881	1.000000	0.528415	49.745648
GOTERM_BP_FAT	GO:0035110 leg morphogenesis	5	0.171821	0.040957	268	39	7937	3.796881	1.000000	0.528415	49.745648
INTERPRO	IPR017973:Cytochrome P450, C-terminal region	8	0.274914	0.041244	386	85	10196	2.486071	1.000000	0.593624	46.466249
GOTERM_BP_FAT	GO:0048598 embryonic morphogenesis	14	0.481100	0.041271	268	226	7937	1.834599	1.000000	0.526610	50.015895
UP_SEQ_FEATURE	domain:EGF-like 3	3	0.103093	0.042221	101	10	2975	8.836634	1.000000	0.741653	46.288970
GOTERM_BP_FAT	GO:0007242 intracellular signaling cascade	17	0.584192	0.042394	268	297	7937	1.695173	1.000000	0.531818	50.970397
GOTERM_BP_FAT	GO:0042493 response to drug	3	0.103093	0.042451	268	10	7937	8.884701	1.000000	0.527845	51.018707
GOTERM_MF_FAT	GO:0016769 transferase activity, transferring nitrogenous groups	4	0.137457	0.042502	299	21	7918	5.044115	1.000000	0.726178	46.562212
GOTERM_MF_FAT	GO:0008034 lipoprotein binding	3	0.103093	0.042666	299	9	7918	8.827202	1.000000	0.707168	46.694543
GOTERM_MF_FAT	GO:0030228 lipoprotein receptor activity	3	0.103093	0.042666	299	9	7918	8.827202	1.000000	0.707168	46.694543
INTERPRO	IPR002007:Haem peroxidase, animal	3	0.103093	0.042944	386	9	10196	8.804836	1.000000	0.596320	47.857121
INTERPRO	IPR000033:Low-density lipoprotein receptor, YWTD repeat	3	0.103093	0.042944	386	9	10196	8.804836	1.000000	0.596320	47.857121
KEGG_PATHWAY	dme00340:Histidine metabolism	3	0.103093	0.042948	89	8	2054	8.654494	0.961164	0.371272	36.837465
INTERPRO	IPR001128:Cytochrome P450	8	0.274914	0.043509	386	86	10196	2.457164	1.000000	0.589212	48.311814

GOTERM_BP_FAT	GO:0048645 organ formation	5	0.171821	0.044349	268	40	7937	3.701959	1.000000	0.539337	52.592126
GOTERM_BP_FAT	GO:0060173 limb development	5	0.171821	0.044349	268	40	7937	3.701959	1.000000	0.539337	52.592126
GOTERM_BP_FAT	GO:0035108 limb morphogenesis	5	0.171821	0.044349	268	40	7937	3.701959	1.000000	0.539337	52.592126
GOTERM_BP_FAT	GO:0051094 positive regulation of developmental process	4	0.137457	0.045233	268	24	7937	4.935945	1.000000	0.542168	53.308334
SP_PIR_KEYWORDS	Monooxygenase	8	0.274914	0.046744	437	98	12980	2.424695	0.999871	0.244026	44.755910
SP_PIR_KEYWORDS	microsome	7	0.240550	0.046963	437	78	12980	2.665611	0.999876	0.238585	44.913584
SP_PIR_KEYWORDS	lipoprotein	6	0.206186	0.047577	437	59	12980	3.020595	0.999890	0.235170	45.351547
KEGG_PATHWAY	dme00053:Ascorbate and aldarate metabolism	5	0.171821	0.049853	89	33	2054	3.496765	0.977274	0.376890	41.447807
GOTERM_BP_FAT	GO:0016318 ommatidial rotation	4	0.137457	0.050165	268	25	7937	4.738507	1.000000	0.576071	57.121909
GOTERM_BP_FAT	GO:0048870 cell motility	12	0.412371	0.050558	268	187	7937	1.900471	1.000000	0.574629	57.413007
GOTERM_BP_FAT	GO:0042430 indole and derivative metabolic process	3	0.103093	0.050755	268	11	7937	8.077001	1.000000	0.571784	57.558642
GOTERM_BP_FAT	GO:0042434 indole derivative metabolic process	3	0.103093	0.050755	268	11	7937	8.077001	1.000000	0.571784	57.558642
PIR_SUPERFAMILY	PIRSF000051:cytochrome P450 CYP3A5	5	0.171821	0.051717	101	37	2596	3.473374	0.996407	0.996407	44.891136
SMART	SM00135:LY	3	0.103093	0.051733	203	9	4824	7.921182	0.999309	0.517001	46.440537
GOTERM_MF_FAT	GO:0008237 metallopeptidase activity	12	0.412371	0.051950	299	168	7918	1.891543	1.000000	0.759143	53.686464
GOTERM_BP_FAT	GO:0048589 developmental growth	7	0.240550	0.051966	268	80	7937	2.591371	1.000000	0.576333	58.440730
INTERPRO	IPR019791:Haem peroxidase, animal, subgroup	3	0.103093	0.052366	386	10	10196	7.924352	1.000000	0.647292	54.974927
GOTERM_MF_FAT	GO:0016877 ligase activity, forming carbon-sulfur bonds	4	0.137457	0.053585	299	23	7918	4.605497	1.000000	0.752444	54.825328
GOTERM_MF_FAT	GO:0005044 scavenger receptor activity	4	0.137457	0.053585	299	23	7918	4.605497	1.000000	0.752444	54.825328
GOTERM_BP_FAT	GO:0048066 pigmentation during development	6	0.206186	0.053601	268	61	7937	2.913017	1.000000	0.583720	59.604452
SP_PIR_KEYWORDS	kinase	13	0.446735	0.054317	437	212	12980	1.821381	0.999971	0.257986	49.956971
SP_PIR_KEYWORDS	DNA binding	9	0.309278	0.055283	437	123	12980	2.173355	0.999976	0.255773	50.587195
GOTERM_BP_FAT	GO:0030718 germline stem cell maintenance	4	0.137457	0.055346	268	26	7937	4.556257	1.000000	0.591579	60.812433
GOTERM_BP_FAT	GO:0009968 negative regulation of signal transduction	8	0.274914	0.055708	268	102	7937	2.322798	1.000000	0.589899	61.058594
INTERPRO	IPR017972:Cytochrome P450, conserved site	8	0.274914	0.056026	386	91	10196	2.322155	1.000000	0.661506	57.487360
GOTERM_BP_FAT	GO:0001708 cell fate specification	6	0.206186	0.056775	268	62	7937	2.866033	1.000000	0.592956	61.776349
GOTERM_BP_FAT	GO:0010648 negative regulation of cell communication	8	0.274914	0.058137	268	103	7937	2.300246	1.000000	0.597854	62.674679
UP_SEQ_FEATURE	domain:EGF-like 2	3	0.103093	0.059291	101	12	2975	7.363861	1.000000	0.835507	58.549504
GOTERM_BP_FAT	GO:0042330 taxis	5	0.171821	0.059444	268	44	7937	3.365417	1.000000	0.602246	63.517535
GOTERM_BP_FAT	GO:0048859 formation of anatomical boundary	5	0.171821	0.059444	268	44	7937	3.365417	1.000000	0.602246	63.517535
GOTERM_BP_FAT	GO:0035309 wing and notum subfield formation	3	0.103093	0.059585	268	12	7937	7.403918	1.000000	0.599179	63.607391
SP_PIR_KEYWORDS	transferase	39	1.340206	0.059698	437	873	12980	1.326917	0.999990	0.267358	53.374365
GOTERM_BP_FAT	GO:0043297 apical junction assembly	4	0.137457	0.060772	268	27	7937	4.387507	1.000000	0.602719	64.355994
GOTERM_BP_FAT	GO:0043473 pigmentation	6	0.206186	0.063440	268	64	7937	2.776469	1.000000	0.615124	65.985675
GOTERM_BP_FAT	GO:0016042 lipid catabolic process	5	0.171821	0.063594	268	45	7937	3.290630	1.000000	0.612200	66.077942
KEGG_PATHWAY	dme00910:Nitrogen metabolism	4	0.137457	0.064973	89	22	2054	4.196118	0.993066	0.424417	50.497893
GOTERM_MF_FAT	GO:0005216 ion channel activity	12	0.412371	0.065763	299	175	7918	1.815882	1.000000	0.806472	62.525586
SP_PIR_KEYWORDS	nidogen binding	2	0.068729	0.066054	437	2	12980	29.702517	0.999997	0.285585	57.134494
SP_PIR_KEYWORDS	Molybdenum	2	0.068729	0.066054	437	2	12980	29.702517	0.999997	0.285585	57.134494
UP_SEQ_FEATURE	domain:Laminin EGF-like 12	2	0.068729	0.066108	101	2	2975	29.455446	1.000000	0.851541	62.673287
UP_SEQ_FEATURE	domain:Laminin EGF-like 11	2	0.068729	0.066108	101	2	2975	29.455446	1.000000	0.851541	62.673287
UP_SEQ_FEATURE	repeat:LDL-receptor class B 2	2	0.068729	0.066108	101	2	2975	29.455446	1.000000	0.851541	62.673287
UP_SEQ_FEATURE	domain:Laminin IV type A	2	0.068729	0.066108	101	2	2975	29.455446	1.000000	0.851541	62.673287
UP_SEQ_FEATURE	repeat:LDL-receptor class B 1	2	0.068729	0.066108	101	2	2975	29.455446	1.000000	0.851541	62.673287
UP_SEQ_FEATURE	domain:Laminin EGF-like 13	2	0.068729	0.066108	101	2	2975	29.455446	1.000000	0.851541	62.673287
UP_SEQ_FEATURE	region of interest:Domain II and I	2	0.068729	0.066108	101	2	2975	29.455446	1.000000	0.851541	62.673287
UP_SEQ_FEATURE	repeat:LDL-receptor class B 3	2	0.068729	0.066108	101	2	2975	29.455446	1.000000	0.851541	62.673287
GOTERM_BP_FAT	GO:0035001 dorsal trunk growth, open tracheal system	2	0.068729	0.066152	268	2	7937	29.615672	1.000000	0.623385	67.570901
GOTERM_BP_FAT	GO:0016142 O-glycoside catabolic process	2	0.068729	0.066152	268	2	7937	29.615672	1.000000	0.623385	67.570901
GOTERM_BP_FAT	GO:0016140 O-glycoside metabolic process	2	0.068729	0.066152	268	2	7937	29.615672	1.000000	0.623385	67.570901
GOTERM_BP_FAT	GO:0016139 glycoside catabolic process	2	0.068729	0.066152	268	2	7937	29.615672	1.000000	0.623385	67.570901
GOTERM_BP_FAT	GO:0014018 neuroblast fate specification	2	0.068729	0.066152	268	2	7937	29.615672	1.000000	0.623385	67.570901

GOTERM_BP_FAT	GO:0051674 localization of cell	12	0.412371	0.066371	268	196	7937	1.813204	1.000000	0.620871	67.695789
INTERPRO	IPR017871:ABC transporter, conserved site	6	0.206186	0.067125	386	58	10196	2.732535	1.000000	0.718341	64.328893
GOTERM_BP_FAT	GO:0042067 establishment of ommatidial polarity	5	0.171821	0.067893	268	46	7937	3.219095	1.000000	0.625770	68.551258
SP_PIR_KEYWORDS	Aminotransferase	3	0.103093	0.068768	437	13	12980	6.854427	0.999998	0.289382	58.653101
GOTERM_BP_FAT	GO:0009066 aspartate family amino acid metabolic process	3	0.103093	0.068895	268	13	7937	6.834386	1.000000	0.627656	69.102742
GOTERM_BP_FAT	GO:0009084 glutamine family amino acid biosynthetic process	3	0.103093	0.068895	268	13	7937	6.834386	1.000000	0.627656	69.102742
GOTERM_MF_FAT	GO:0003704 specific RNA polymerase II transcription factor activity	7	0.240550	0.069229	299	77	7918	2.407419	1.000000	0.808589	64.482228
GOTERM_CC_FAT	GO:0043256 laminin complex	2	0.068729	0.070189	172	2	4786	27.825581	0.999999	0.468372	59.556575
GOTERM_CC_FAT	GO:0005606 laminin-1 complex	2	0.068729	0.070189	172	2	4786	27.825581	0.999999	0.468372	59.556575
INTERPRO	IPR002401:Cytochrome P450, E-class, group I	7	0.240550	0.070320	386	77	10196	2.401319	1.000000	0.725179	66.099170
GOTERM_BP_FAT	GO:0007043 cell-cell junction assembly	4	0.137457	0.072333	268	29	7937	4.084920	1.000000	0.642582	70.927340
GOTERM_BP_FAT	GO:0035127 post-embryonic limb morphogenesis	4	0.137457	0.072333	268	29	7937	4.084920	1.000000	0.642582	70.927340
GOTERM_BP_FAT	GO:0008652 cellular amino acid biosynthetic process	4	0.137457	0.072333	268	29	7937	4.084920	1.000000	0.642582	70.927340
GOTERM_BP_FAT	GO:0007480 imaginal disc-derived leg morphogenesis	4	0.137457	0.072333	268	29	7937	4.084920	1.000000	0.642582	70.927340
GOTERM_BP_FAT	GO:0009309 amine biosynthetic process	5	0.171821	0.072338	268	47	7937	3.150603	1.000000	0.638988	70.930142
GOTERM_MF_FAT	GO:0008307 structural constituent of muscle	3	0.103093	0.072657	299	12	7918	6.620401	1.000000	0.810389	66.323592
INTERPRO	IPR001589:Actinin-type, actin-binding, conserved site	3	0.103093	0.073106	386	12	10196	6.603627	1.000000	0.729488	67.575318
GOTERM_BP_FAT	GO:0007163 establishment or maintenance of cell polarity	8	0.274914	0.074077	268	109	7937	2.173627	1.000000	0.644474	71.813707
INTERPRO	IPR009254:Laminin I	2	0.068729	0.074098	386	2	10196	26.414508	1.000000	0.724741	68.086234
INTERPRO	IPR000585: Hemopexin/ matrixin	2	0.068729	0.074098	386	2	10196	26.414508	1.000000	0.724741	68.086234
INTERPRO	IPR010307:Laminin II	2	0.068729	0.074098	386	2	10196	26.414508	1.000000	0.724741	68.086234
INTERPRO	IPR018487: Hemopexin/ matrixin, repeat	2	0.068729	0.074098	386	2	10196	26.414508	1.000000	0.724741	68.086234
INTERPRO	IPR001818:Peptidase M10A and M12B, matrixin and adamalysin	2	0.068729	0.074098	386	2	10196	26.414508	1.000000	0.724741	68.086234
GOTERM_BP_FAT	GO:0007422 peripheral nervous system development	7	0.240550	0.075422	268	88	7937	2.355792	1.000000	0.647800	72.479735
GOTERM_MF_FAT	GO:0022838 substrate specific channel activity	12	0.412371	0.076970	299	180	7918	1.765440	1.000000	0.815844	68.514170
KEGG_PATHWAY	dme00670:One carbon pool by folate	3	0.103093	0.077641	89	11	2054	6.294178	0.997473	0.450130	57.084017
GOTERM_BP_FAT	GO:0035222 wing disc pattern formation	6	0.206186	0.078031	268	68	7937	2.613147	1.000000	0.657291	73.730300
GOTERM_BP_FAT	GO:0048588 developmental cell growth	4	0.137457	0.078455	268	30	7937	3.948756	1.000000	0.655881	73.928099
GOTERM_BP_FAT	GO:0008406 gonad development	4	0.137457	0.078455	268	30	7937	3.948756	1.000000	0.655881	73.928099
GOTERM_BP_FAT	GO:0048608 reproductive structure development	4	0.137457	0.078455	268	30	7937	3.948756	1.000000	0.655881	73.928099
GOTERM_BP_FAT	GO:0035109 imaginal disc-derived limb morphogenesis	4	0.137457	0.078455	268	30	7937	3.948756	1.000000	0.655881	73.928099
GOTERM_MF_FAT	GO:0030528 transcription regulator activity	34	1.168385	0.078464	299	682	7918	1.320197	1.000000	0.809317	69.241536
SP_PIR_KEYWORDS	lipid metabolism	3	0.103093	0.078497	437	14	12980	6.364825	1.000000	0.317627	63.700247
GOTERM_BP_FAT	GO:0010623 developmental programmed cell death	3	0.103093	0.078643	268	14	7937	6.346215	1.000000	0.653351	74.015642
KEGG_PATHWAY	dme00640:Propanoate metabolism	4	0.137457	0.080361	89	24	2054	3.846442	0.997969	0.430828	58.390432
UP_SEQ_FEATURE	compositionally biased region:Gln-rich	10	0.343643	0.081052	101	158	2975	1.864269	1.000000	0.892823	70.414824
SMART	SM00120:HX	2	0.068729	0.082003	203	2	4824	23.763547	0.999992	0.655487	63.421063
GOTERM_MF_FAT	GO:0015370 solute:sodium symporter activity	6	0.206186	0.083062	299	62	7918	2.562736	1.000000	0.815656	71.383261
GOTERM_CC_FAT	GO:0005624 membrane fraction	8	0.274914	0.083657	172	106	4786	2.100044	1.000000	0.515918	66.269151
GOTERM_MF_FAT	GO:0016638 oxidoreductase activity, acting on the CH-NH2 group of donors	3	0.103093	0.083795	299	13	7918	6.111140	1.000000	0.806666	71.711534
GOTERM_BP_FAT	GO:0007478 leg disc morphogenesis	4	0.137457	0.084795	268	31	7937	3.821377	1.000000	0.678733	76.727598

GOTERM_BP_FAT	GO:0001738 morphogenesis of a polarized epithelium	6	0.206186	0.085949	268	70	7937	2.538486	1.000000	0.680528	77.205764
INTERPRO	IPR000873:AMP-dependent synthetase and ligase	4	0.137457	0.087291	386	28	10196	3.773501	1.000000	0.774686	74.206632
GOTERM_BP_FAT	GO:0030198 extracellular matrix organization	3	0.103093	0.088790	268	15	7937	5.923134	1.000000	0.689597	78.344022
GOTERM_BP_FAT	GO:0007432 salivary gland boundary specification	3	0.103093	0.088790	268	15	7937	5.923134	1.000000	0.689597	78.344022
SP_PIR_KEYWORDS	differentiation	10	0.343643	0.090394	437	160	12980	1.856407	1.000000	0.350872	69.100792
INTERPRO	IPR003961:Fibronectin, type III	6	0.206186	0.093432	386	64	10196	2.476360	1.000000	0.789730	76.664626
GOTERM_BP_FAT	GO:0044271 nitrogen compound biosynthetic process	13	0.446735	0.093832	268	233	7937	1.652377	1.000000	0.707277	80.233533
COG_ONTOLOGY	Secondary metabolites biosynthesis, transport, and catabolism	8	0.274914	0.095060	55	91	1237	1.977223	0.816963	0.572172	51.481670
SP_PIR_KEYWORDS	synapse	5	0.171821	0.096550	437	52	12980	2.856011	1.000000	0.363691	71.595309
GOTERM_CC_FAT	GO:0005626 insoluble fraction	8	0.274914	0.097288	172	110	4786	2.023679	1.000000	0.557162	72.006516
UP_SEQ_FEATURE	domain:Ig-like V-type	2	0.068729	0.097520	101	3	2975	19.636964	1.000000	0.923884	77.200790
UP_SEQ_FEATURE	domain:WIF	2	0.068729	0.097520	101	3	2975	19.636964	1.000000	0.923884	77.200790
GOTERM_BP_FAT	GO:0007421 stomatogastric nervous system development	2	0.068729	0.097575	268	3	7937	19.743781	1.000000	0.718803	81.534746
GOTERM_BP_FAT	GO:0034769 basement membrane disassembly	2	0.068729	0.097575	268	3	7937	19.743781	1.000000	0.718803	81.534746
GOTERM_BP_FAT	GO:0022617 extracellular matrix disassembly	2	0.068729	0.097575	268	3	7937	19.743781	1.000000	0.718803	81.534746
GOTERM_BP_FAT	GO:0051270 regulation of cell motion	2	0.068729	0.097575	268	3	7937	19.743781	1.000000	0.718803	81.534746
GOTERM_BP_FAT	GO:0030334 regulation of cell migration	2	0.068729	0.097575	268	3	7937	19.743781	1.000000	0.718803	81.534746
UP_SEQ_FEATURE	compositionally biased region:Poly-Gln	7	0.240550	0.097744	101	95	2975	2.170401	1.000000	0.914460	77.282000
GOTERM_BP_FAT	GO:0035317 imaginal disc-derived wing hair organization	4	0.137457	0.098100	268	33	7937	3.589778	1.000000	0.717650	81.710889
GOTERM_BP_FAT	GO:0019827 stem cell maintenance	4	0.137457	0.098100	268	33	7937	3.589778	1.000000	0.717650	81.710889
INTERPRO	IPR001478: PDZ/DHR/ GLGF	6	0.206186	0.098264	386	65	10196	2.438262	1.000000	0.798752	78.443379
GOTERM_CC_FAT	GO:0009986 cell surface	3	0.103093	0.098313	172	15	4786	5.565116	1.000000	0.546447	72.399157
SP_PIR_KEYWORDS	transmembrane protein	6	0.206186	0.098482	437	73	12980	2.441303	1.000000	0.362926	72.339306
GOTERM_BP_FAT	GO:0046545 development of primary female sexual characteristics	3	0.103093	0.099298	268	16	7937	5.552938	1.000000	0.719071	82.106592
GOTERM_BP_FAT	GO:0030155 regulation of cell adhesion	3	0.103093	0.099298	268	16	7937	5.552938	1.000000	0.719071	82.106592
GOTERM_MF_FAT	GO:0043169 cation binding	77	2.646048	0.099306	299	1764	7918	1.155943	1.000000	0.849503	77.888504

Table 9: Architecture 9

Category	Term	Count	%	PValue	List Total	Pop Hits	Pop Total	Fold Enrichment	Bonferroni	Benjamini	FDR
SP_PIR_KEYWORDS	membrane	27	5.046729	6.396257e-04	152	1139	12980	2.024283	0.072127	0.072127	0.728448
GOTERM_CC_FAT	GO:0031224 intrinsic to membrane	30	5.607477	7.435396e-04	60	1382	4786	1.731548	0.096883	0.096883	0.870483
GOTERM_CC_FAT	GO:0016021 integral to membrane	29	5.420561	0.001383	60	1360	4786	1.700907	0.172724	0.090453	1.613691
SP_PIR_KEYWORDS	transmembrane	24	4.485981	0.002029	152	1039	12980	1.972544	0.211496	0.112022	2.293949
GOTERM_BP_FAT	GO:0007155 cell adhesion	8	1.495327	0.003282	90	174	7937	4.054662	0.913622	0.913622	4.875875
GOTERM_BP_FAT	GO:0022610 biological adhesion	8	1.495327	0.005020	90	188	7937	3.752719	0.976468	0.846598	7.367455
GOTERM_BP_FAT	GO:0016311 dephosphorylation	6	1.121495	0.007417	90	109	7937	4.854434	0.996097	0.842559	10.703046
GOTERM_CC_FAT	GO:0033180 proton-transporting V-type ATPase, V1 domain	3	0.560748	0.018044	60	17	4786	14.076471	0.917467	0.564612	19.267176
GOTERM_MF_FAT	GO:0046933 hydrogen ion transporting ATP synthase activity, rotational mechanism	3	0.560748	0.022389	104	18	7918	12.689103	0.998460	0.998460	25.946317
GOTERM_BP_FAT	GO:0006812 cation transport	8	1.495327	0.022651	90	252	7937	2.799647	1.000000	0.985981	29.418736
GOTERM_BP_FAT	GO:0005996 monosaccharide metabolic process	5	0.934579	0.023233	90	97	7937	4.545819	1.000000	0.969879	30.054541
GOTERM_CC_FAT	GO:0008021 synaptic vesicle	4	0.747664	0.028307	60	54	4786	5.908642	0.980434	0.625995	28.646239
GOTERM_BP_FAT	GO:0034637 cellular carbohydrate biosynthetic process	3	0.560748	0.029245	90	24	7937	11.023611	1.000000	0.974912	36.322517
SMART	SM00724:TLC	2	0.373832	0.030170	50	3	4824	64.320000	0.820135	0.820135	26.103145
SP_PIR_KEYWORDS	oxidoreductase	13	2.429907	0.030669	152	561	12980	1.978844	0.973863	0.703230	29.947530
GOTERM_MF_FAT	GO:0016791 phosphatase activity	7	1.308411	0.031015	104	182	7918	2.928254	0.999878	0.988950	34.159920
GOTERM_BP_FAT	GO:0042335 cuticle development	4	0.747664	0.034767	90	64	7937	5.511806	1.000000	0.976856	41.613496
GOTERM_MF_FAT	GO:0046961 proton-transporting ATPase activity, rotational mechanism	3	0.560748	0.035502	104	23	7918	9.930602	0.999968	0.968128	38.091686
GOTERM_CC_FAT	GO:0030136 clathrin-coated vesicle	4	0.747664	0.035538	60	59	4786	5.407910	0.992968	0.628967	34.644073
GOTERM_BP_FAT	GO:0055085 transmembrane transport	5	0.934579	0.036743	90	112	7937	3.937004	1.000000	0.969380	43.404772
INTERPRO	IPR006634:TRAM, LAG1 and CLN8 homology	2	0.373832	0.038342	133	3	10196	51.107769	0.999988	0.999988	40.532607
GOTERM_BP_FAT	GO:0006811 ion transport	9	1.682243	0.039651	90	346	7937	2.293931	1.000000	0.964882	45.948006
GOTERM_BP_FAT	GO:0048812 neuron projection morphogenesis	8	1.495327	0.041002	90	286	7937	2.466822	1.000000	0.955800	47.092903
GOTERM_BP_FAT	GO:0031175 neuron projection development	8	1.495327	0.041656	90	287	7937	2.458227	1.000000	0.943958	47.638551
GOTERM_BP_FAT	GO:0048667 cell morphogenesis involved in neuron differentiation	8	1.495327	0.042316	90	288	7937	2.449691	1.000000	0.931732	48.184534
GOTERM_BP_FAT	GO:0055114 oxidation reduction	13	2.429907	0.043198	90	620	7937	1.849122	1.000000	0.920390	48.905284
GOTERM_BP_FAT	GO:0006793 phosphorus metabolic process	12	2.242991	0.043242	90	551	7937	1.920629	1.000000	0.904855	48.941616
GOTERM_BP_FAT	GO:0006796 phosphate metabolic process	12	2.242991	0.043242	90	551	7937	1.920629	1.000000	0.904855	48.941616
GOTERM_BP_FAT	GO:0015672 monovalent inorganic cation transport	6	1.121495	0.046364	90	175	7937	3.023619	1.000000	0.905377	51.417216
GOTERM_CC_FAT	GO:0033178 proton-transporting two-sector ATPase complex, catalytic domain	3	0.560748	0.049033	60	29	4786	8.251724	0.998980	0.682722	44.620304
GOTERM_BP_FAT	GO:0007224 smoothened signaling pathway	3	0.560748	0.052449	90	33	7937	8.017172	1.000000	0.918610	55.922787
GOTERM_BP_FAT	GO:0000904 cell morphogenesis involved in differentiation	8	1.495327	0.053050	90	303	7937	2.328420	1.000000	0.908259	56.346431
GOTERM_MF_FAT	GO:0015405 P-P-bond-hydrolysis-driven transmembrane transporter activity	6	1.121495	0.053512	104	157	7918	2.909603	1.000000	0.980401	51.788328
GOTERM_MF_FAT	GO:0015399 primary active transmembrane transporter activity	6	1.121495	0.053512	104	157	7918	2.909603	1.000000	0.980401	51.788328
GOTERM_MF_FAT	GO:0019829 cation-transporting ATPase activity	3	0.560748	0.054191	104	29	7918	7.875995	1.000000	0.958698	52.244772
GOTERM_BP_FAT	GO:0006754 ATP biosynthetic process	4	0.747664	0.056841	90	78	7937	4.522507	1.000000	0.911265	58.929627
GOTERM_CC_FAT	GO:0005783 endoplasmic reticulum	8	1.495327	0.058321	60	284	4786	2.246948	0.999734	0.691507	50.653971
SP_PIR_KEYWORDS	protein phosphatase	3	0.560748	0.059193	152	34	12980	7.534830	0.999206	0.832161	50.203428
GOTERM_BP_FAT	GO:0046034 ATP metabolic process	4	0.747664	0.060430	90	80	7937	4.409444	1.000000	0.913195	61.242694
SP_PIR_KEYWORDS	ion transport	6	1.121495	0.061373	152	182	12980	2.815211	0.999395	0.772839	51.506310
GOTERM_BP_FAT	GO:0009206 purine ribonucleoside triphosphate biosynthetic process	4	0.747664	0.064122	90	82	7937	4.301897	1.000000	0.915295	63.495462
GOTERM_BP_FAT	GO:0009145 purine nucleoside triphosphate biosynthetic process	4	0.747664	0.064122	90	82	7937	4.301897	1.000000	0.915295	63.495462
GOTERM_CC_FAT	GO:0033176 proton-transporting V-type ATPase complex	3	0.560748	0.065171	60	34	4786	7.038235	0.999902	0.684653	54.712176
GOTERM_BP_FAT	GO:0009142 nucleoside triphosphate biosynthetic process	4	0.747664	0.066007	90	83	7937	4.250067	1.000000	0.911305	64.597615
GOTERM_BP_FAT	GO:0009201 ribonucleoside triphosphate biosynthetic process	4	0.747664	0.066007	90	83	7937	4.250067	1.000000	0.911305	64.597615
GOTERM_CC_FAT	GO:0030135 coated vesicle	4	0.747664	0.066453	60	76	4786	4.198246	0.999919	0.648922	55.437075
GOTERM_BP_FAT	GO:0048858 cell projection morphogenesis	8	1.495327	0.067137	90	320	7937	2.204722	1.000000	0.904957	65.243333

KEGG_PATHWAY	dme00030:Pentose phosphate pathway	3	0.560748	0.069185	40	23	2054	6.697826	0.974175	0.974175	50.021072
GOTERM_BP_FAT	GO:0016192 vesicle-mediated transport	9	1.682243	0.071159	90	391	7937	2.029923	1.000000	0.908466	67.453307
GOTERM_MF_FAT	GO:0046872 metal ion binding	30	5.607477	0.072422	104	1718	7918	1.329475	1.000000	0.972222	63.112133
SP_PIR_KEYWORDS	zinc	12	2.242991	0.073547	152	577	12980	1.775974	0.999869	0.774548	58.226537
GOTERM_BP_FAT	GO:0009205 purine ribonucleoside triphosphate metabolic process	4	0.747664	0.073799	90	87	7937	4.054662	1.000000	0.907427	68.831825
GOTERM_BP_FAT	GO:0009144 purine nucleoside triphosphate metabolic process	4	0.747664	0.073799	90	87	7937	4.054662	1.000000	0.907427	68.831825
GOTERM_BP_FAT	GO:0009199 ribonucleoside triphosphate metabolic process	4	0.747664	0.075808	90	88	7937	4.008586	1.000000	0.904564	69.844444
GOTERM_BP_FAT	GO:0006732 coenzyme metabolic process	4	0.747664	0.075808	90	88	7937	4.008586	1.000000	0.904564	69.844444
GOTERM_BP_FAT	GO:0032990 cell part morphogenesis	8	1.495327	0.077360	90	331	7937	2.131454	1.000000	0.900450	70.605352
GOTERM_BP_FAT	GO:0009141 nucleoside triphosphate metabolic process	4	0.747664	0.077842	90	89	7937	3.963546	1.000000	0.893121	70.837903
SP_PIR_KEYWORDS	metal-binding	15	2.803738	0.079865	152	796	12980	1.609197	0.999941	0.751227	61.368408
GOTERM_BP_FAT	GO:0033365 protein localization in organelle	4	0.747664	0.081981	90	91	7937	3.876435	1.000000	0.897295	72.766093
GOTERM_MF_FAT	GO:0042625 ATPase activity, coupled to transmembrane movement of ions	4	0.747664	0.083317	104	79	7918	3.854917	1.000000	0.971399	68.463470
GOTERM_BP_FAT	GO:0009108 coenzyme biosynthetic process	3	0.560748	0.083541	90	43	7937	6.152713	1.000000	0.893659	73.461261
KEGG_PATHWAY	dme00565:Ether lipid metabolism	3	0.560748	0.085791	40	26	2054	5.925000	0.989689	0.898455	58.009062
GOTERM_CC_FAT	GO:0031300 intrinsic to organelle membrane	3	0.560748	0.086505	60	40	4786	5.982500	0.999996	0.710482	65.474903
INTERPRO	IPR000194:ATPase, F1/V1/A1 complex, alpha/beta subunit, nucleotide-binding domain	2	0.373832	0.087204	133	7	10196	21.903330	1.000000	0.999998	70.268735
INTERPRO	IPR000793:ATPase, F1/V1/A1 complex, alpha/beta subunit, C-terminal	2	0.373832	0.087204	133	7	10196	21.903330	1.000000	0.999998	70.268735
INTERPRO	IPR004100:ATPase, F1/V1/A1 complex, alpha/beta subunit, N-terminal	2	0.373832	0.087204	133	7	10196	21.903330	1.000000	0.999998	70.268735
UP_SEQ_FEATURE	active site:Phosphocysteine intermediate	2	0.373832	0.087953	35	8	2975	21.250000	0.999990	0.999990	65.514766
GOTERM_BP_FAT	GO:0009165 nucleotide biosynthetic process	5	0.934579	0.089191	90	151	7937	2.920162	1.000000	0.901726	75.843463
GOTERM_BP_FAT	GO:0016042 lipid catabolic process	3	0.560748	0.090303	90	45	7937	5.879259	1.000000	0.897151	76.287851
GOTERM_BP_FAT	GO:0048666 neuron development	8	1.495327	0.093787	90	347	7937	2.033173	1.000000	0.899013	77.632051
GOTERM_BP_FAT	GO:0019637 organophosphate metabolic process	4	0.747664	0.094952	90	97	7937	3.636655	1.000000	0.894847	78.065409
GOTERM_MF_FAT	GO:0043169 cation binding	30	5.607477	0.095087	104	1764	7918	1.294806	1.000000	0.971901	73.431652
SP_PIR_KEYWORDS	glycosyltransferase	5	0.934579	0.095438	152	149	12980	2.865595	0.999992	0.769374	68.214240
GOTERM_MF_FAT	GO:0043167 ion binding	30	5.607477	0.099477	104	1772	7918	1.288961	1.000000	0.964195	75.091536
SP_PIR_KEYWORDS	cell adhesion	3	0.560748	0.099912	152	46	12980	5.569222	0.999996	0.745490	69.964939

Table 10: Architecture 10

Category	Term	Count	%	PValue	List Total	Pop Hits	Pop Total	Fold Enrichment	Bonferroni	Benjamini	FDR
GOTERM_BP_FAT	GO:0007517 muscle organ development	10	0.451671	0.001017	146	140	7937	3.883072	0.692822	0.692822	1.624716
GOTERM_MF_FAT	GO:0015103 inorganic anion transmembrane transporter activity	6	0.271003	0.001384	176	38	7918	7.103469	0.392654	0.392654	1.884403
INTERPRO	IPR011701:Major facilitator superfamily MFS-1	10	0.451671	0.001424	247	111	10196	3.718861	0.501038	0.501038	2.023777
GOTERM_MF_FAT	GO:0050662 coenzyme binding	10	0.451671	0.003586	176	139	7918	3.236593	0.725618	0.476185	4.813975
SP_PIR_KEYWORDS	phosphoprotein	30	1.355014	0.005444	281	815	12980	1.700325	0.598142	0.598142	6.424249
GOTERM_BP_FAT	GO:0016358 dendrite development	8	0.361337	0.005474	146	117	7937	3.717129	0.998283	0.958560	8.456996
GOTERM_BP_FAT	GO:0048813 dendrite morphogenesis	8	0.361337	0.005474	146	117	7937	3.717129	0.998283	0.958560	8.456996
GOTERM_MF_FAT	GO:0003779 actin binding	9	0.406504	0.005504	176	122	7918	3.318834	0.862861	0.484312	7.299399
GOTERM_BP_FAT	GO:0048812 neuron projection morphogenesis	13	0.587173	0.005997	146	286	7937	2.471046	0.999067	0.902291	9.229028
GOTERM_BP_FAT	GO:0031175 neuron projection development	13	0.587173	0.006162	146	287	7937	2.462436	0.999231	0.833459	9.471532
GOTERM_BP_FAT	GO:0048667 cell morphogenesis involved in neuron differentiation	13	0.587173	0.006331	146	288	7937	2.453886	0.999368	0.770857	9.718688
INTERPRO	IPR001251:Cellular retinaldehyde-binding/triple function, C-terminal	5	0.225836	0.007809	247	33	10196	6.254447	0.978203	0.852362	10.641636
SMART	SM00516:SEC14	5	0.225836	0.008289	120	33	4824	6.090909	0.603053	0.603053	8.990138
GOTERM_MF_FAT	GO:0015294 solute:cation symporter activity	7	0.316170	0.008331	176	80	7918	3.936506	0.950790	0.529007	10.854026
GOTERM_MF_FAT	GO:0015296 anion:cation symporter activity	5	0.225836	0.008622	176	37	7918	6.079545	0.955735	0.463941	11.213481
GOTERM_MF_FAT	GO:0008092 cytoskeletal protein binding	12	0.542005	0.008843	176	218	7918	2.476439	0.959147	0.413137	11.484812
GOTERM_BP_FAT	GO:0000904 cell morphogenesis involved in differentiation	13	0.587173	0.009331	146	303	7937	2.332407	0.999981	0.836756	14.008700
GOTERM_MF_FAT	GO:0016564 transcription repressor activity	8	0.361337	0.010289	176	109	7918	3.301918	0.975844	0.412505	13.241462
GOTERM_MF_FAT	GO:0015293 symporter activity	7	0.316170	0.010486	176	84	7918	3.749053	0.977516	0.377723	13.478604
GOTERM_MF_FAT	GO:0005436 sodium:phosphate symporter activity	4	0.180668	0.010531	176	21	7918	8.569264	0.977881	0.345237	13.532633
GOTERM_MF_FAT	GO:0050660 FAD binding	6	0.271003	0.010921	176	61	7918	4.425112	0.980806	0.326533	13.999200
GOTERM_BP_FAT	GO:0048858 cell projection morphogenesis	13	0.587173	0.013966	146	320	7937	2.208497	1.000000	0.902763	20.260810
GOTERM_BP_FAT	GO:0002831 regulation of response to biotic stimulus	4	0.180668	0.014007	146	28	7937	7.766145	1.000000	0.870671	20.314953
GOTERM_BP_FAT	GO:0002759 regulation of antimicrobial humoral response	4	0.180668	0.014007	146	28	7937	7.766145	1.000000	0.870671	20.314953
GOTERM_BP_FAT	GO:0002920 regulation of humoral immune response	4	0.180668	0.014007	146	28	7937	7.766145	1.000000	0.870671	20.314953
GOTERM_BP_FAT	GO:0043900 regulation of multi-organism process	4	0.180668	0.014007	146	28	7937	7.766145	1.000000	0.870671	20.314953
GOTERM_BP_FAT	GO:0008358 maternal determination of anterior/posterior axis, embryo	6	0.271003	0.016032	146	81	7937	4.026890	1.000000	0.875448	22.908284
GOTERM_BP_FAT	GO:0030182 neuron differentiation	15	0.677507	0.017121	146	409	7937	1.993754	1.000000	0.865102	24.271025
GOTERM_BP_FAT	GO:0032990 cell part morphogenesis	13	0.587173	0.017792	146	331	7937	2.135103	1.000000	0.849406	25.099328
GOTERM_MF_FAT	GO:0015114 phosphate transmembrane transporter activity	4	0.180668	0.018996	176	26	7918	6.921329	0.998997	0.466171	23.157112
GOTERM_BP_FAT	GO:0002520 immune system development	5	0.225836	0.019982	146	57	7937	4.768685	1.000000	0.857888	27.742563
GOTERM_BP_FAT	GO:0060538 skeletal muscle organ development	5	0.225836	0.019982	146	57	7937	4.768685	1.000000	0.857888	27.742563
GOTERM_BP_FAT	GO:0048534 hemopoietic or lymphoid organ development	5	0.225836	0.019982	146	57	7937	4.768685	1.000000	0.857888	27.742563
GOTERM_MF_FAT	GO:0048037 cofactor binding	10	0.451671	0.021298	176	185	7918	2.431818	0.999569	0.475785	25.596946
GOTERM_BP_FAT	GO:0031328 positive regulation of cellular biosynthetic process	8	0.361337	0.021815	146	153	7937	2.842511	1.000000	0.860281	29.888013
GOTERM_BP_FAT	GO:0009891 positive regulation of biosynthetic process	8	0.361337	0.021815	146	153	7937	2.842511	1.000000	0.860281	29.888013
GOTERM_BP_FAT	GO:0032386 regulation of intracellular transport	4	0.180668	0.023638	146	34	7937	6.395649	1.000000	0.862220	31.962125
SP_PIR_KEYWORDS	zinc finger	5	0.225836	0.023963	281	51	12980	4.528644	0.982587	0.868042	25.547875
GOTERM_BP_FAT	GO:0048666 neuron development	13	0.587173	0.024707	146	347	7937	2.036655	1.000000	0.855529	33.151510
GOTERM_BP_FAT	GO:0007519 skeletal muscle tissue development	4	0.180668	0.025516	146	35	7937	6.212916	1.000000	0.846482	34.038822
GOTERM_BP_FAT	GO:0035186 syncytial blastoderm mitotic cell cycle	3	0.135501	0.026123	146	14	7937	11.649217	1.000000	0.835724	34.696964
KEGG_PATHWAY	dme00564:Glycerophospholipid metabolism	6	0.271003	0.026498	57	63	2054	3.431913	0.820710	0.820710	23.877435
GOTERM_BP_FAT	GO:0007613 memory	4	0.180668	0.027472	146	36	7937	6.040335	1.000000	0.833909	36.138497
INTERPRO	IPR000210:BTB/POZ-like	6	0.271003	0.029682	247	72	10196	3.439946	1.000000	0.992564	35.106592
GOTERM_BP_FAT	GO:0007632 visual behavior	3	0.135501	0.029786	146	15	7937	10.872603	1.000000	0.842157	38.540875
GOTERM_BP_FAT	GO:0033301 cell cycle comprising mitosis without cytokinesis	3	0.135501	0.029786	146	15	7937	10.872603	1.000000	0.842157	38.540875
UP_SEQ_FEATURE	compositionally biased region:Poly-Ser	7	0.316170	0.031161	69	105	2975	2.874396	0.999887	0.999887	34.306342
SMART	SM00225:BTB	6	0.271003	0.031627	120	72	4824	3.350000	0.971769	0.831980	30.490818
GOTERM_BP_FAT	GO:0030030 cell projection organization	13	0.587173	0.034658	146	365	7937	1.936217	1.000000	0.870728	43.325325
GOTERM_BP_FAT	GO:0060341 regulation of cellular localization	5	0.225836	0.035374	146	68	7937	3.997280	1.000000	0.863220	43.997863
GOTERM_BP_FAT	GO:0002164 larval development	5	0.225836	0.035374	146	68	7937	3.997280	1.000000	0.863220	43.997863

GOTERM_BP_FAT	GO:0014706 striated muscle tissue development	4	0.180668	0.036073	146	40	7937	5.436301	1.000000	0.855890	44.647686
SP_PIR_KEYWORDS	phosphotransferase	4	0.180668	0.036332	281	34	12980	5.434373	0.997931	0.872567	36.246413
GOTERM_BP_FAT	GO:0060537 muscle tissue development	4	0.180668	0.038415	146	41	7937	5.303709	1.000000	0.861329	46.773557
GOTERM_CC_FAT	GO:0001726 ruffle	2	0.090334	0.040128	98	2	4786	48.836735	0.999687	0.999687	40.074359
GOTERM_MF_FAT	GO:0017076 purine nucleotide binding	31	1.400181	0.042937	176	989	7918	1.410159	1.000000	0.703381	45.268698
GOTERM_BP_FAT	GO:0006007 glucose catabolic process	4	0.180668	0.043326	146	43	7937	5.057025	1.000000	0.882441	50.985094
GOTERM_BP_FAT	GO:0019320 hexose catabolic process	4	0.180668	0.043326	146	43	7937	5.057025	1.000000	0.882441	50.985094
INTERPRO	IPR000159:Ras-association	3	0.135501	0.043593	247	14	10196	8.845575	1.000000	0.995651	47.252458
SP_PIR_KEYWORDS	zinc	20	0.903342	0.044394	281	577	12980	1.601115	0.999491	0.849807	42.438689
UP_SEQ_FEATURE	region of interest:Interaction with NEDD4	2	0.090334	0.045199	69	2	2975	43.115942	0.999998	0.998689	45.876356
SMART	SM00314:RA	3	0.135501	0.045255	120	14	4824	8.614286	0.994145	0.819765	40.791375
SP_PIR_KEYWORDS	muscle protein	3	0.135501	0.045603	281	16	12980	8.661032	0.999588	0.789644	43.318266
INTERPRO	IPR011333:BTB/POZ fold	6	0.271003	0.045784	247	81	10196	3.057730	1.000000	0.989684	48.960502
GOTERM_BP_FAT	GO:0046365 monosaccharide catabolic process	4	0.180668	0.045894	146	44	7937	4.942092	1.000000	0.886946	53.060610
GOTERM_MF_FAT	GO:0015370 solute:sodium symporter activity	5	0.225836	0.047754	176	62	7918	3.628116	1.000000	0.715851	48.933318
GOTERM_MF_FAT	GO:0008017 microtubule binding	5	0.225836	0.050147	176	63	7918	3.570527	1.000000	0.709096	50.667911
UP_SEQ_FEATURE	zinc finger region:RING-type	3	0.135501	0.050265	69	16	2975	8.084239	1.000000	0.992800	49.565816
GOTERM_BP_FAT	GO:0007611 learning or memory	5	0.225836	0.051934	146	77	7937	3.530066	1.000000	0.907394	57.622624
GOTERM_MF_FAT	GO:0008509 anion transmembrane transporter activity	6	0.271003	0.052602	176	92	7918	2.934042	1.000000	0.703527	52.389982
GOTERM_BP_FAT	GO:0009791 post-embryonic development	14	0.632340	0.053491	146	434	7937	1.753646	1.000000	0.905756	58.728808
GOTERM_BP_FAT	GO:0007314 oocyte anterior/posterior axis specification	5	0.225836	0.053996	146	78	7937	3.484809	1.000000	0.899705	59.082092
GOTERM_BP_FAT	GO:0051235 maintenance of location	4	0.180668	0.054037	146	47	7937	4.626639	1.000000	0.891618	59.110904
GOTERM_MF_FAT	GO:0030554 adenyl nucleotide binding	26	1.174345	0.054168	176	812	7918	1.440523	1.000000	0.692515	53.459989
GOTERM_MF_FAT	GO:0001883 purine nucleoside binding	26	1.174345	0.057467	176	817	7918	1.431707	1.000000	0.693852	55.640403
GOTERM_MF_FAT	GO:0005316 high affinity inorganic phosphate:sodium symporter activity	3	0.135501	0.058951	176	18	7918	7.498106	1.000000	0.683756	56.590054
UP_SEQ_FEATURE	compositionally biased region:Ser-rich	7	0.316170	0.059850	69	123	2975	2.453753	1.000000	0.988063	55.919045
GOTERM_BP_FAT	GO:0000902 cell morphogenesis	14	0.632340	0.060144	146	442	7937	1.721905	1.000000	0.909142	63.159529
GOTERM_BP_FAT	GO:0008045 motor axon guidance	3	0.135501	0.060333	146	22	7937	7.413138	1.000000	0.902567	63.278273
UP_SEQ_FEATURE	domain:BTB	3	0.135501	0.062250	69	18	2975	7.185990	1.000000	0.975008	57.389287
GOTERM_MF_FAT	GO:0001882 nucleoside binding	26	1.174345	0.062325	176	824	7918	1.419544	1.000000	0.685993	58.679779
GOTERM_MF_FAT	GO:0015631 tubulin binding	5	0.225836	0.063082	176	68	7918	3.307988	1.000000	0.672745	59.135185
GOTERM_BP_FAT	GO:0016052 carbohydrate catabolic process	5	0.225836	0.064961	146	83	7937	3.274880	1.000000	0.912386	66.084259
GOTERM_BP_FAT	GO:0035150 regulation of tube size	3	0.135501	0.065308	146	23	7937	7.090828	1.000000	0.906900	66.286328
GOTERM_BP_FAT	GO:0046164 alcohol catabolic process	4	0.180668	0.065892	146	51	7937	4.263766	1.000000	0.902271	66.623434
GOTERM_BP_FAT	GO:0044275 cellular carbohydrate catabolic process	4	0.180668	0.065892	146	51	7937	4.263766	1.000000	0.902271	66.623434
INTERPRO	IPR001071:Cellular retinaldehyde binding/alpha-tocopherol transport	3	0.135501	0.068846	247	18	10196	6.879892	1.000000	0.996977	64.073016
GOTERM_BP_FAT	GO:0002697 regulation of immune effector process	3	0.135501	0.070416	146	24	7937	6.795377	1.000000	0.911080	69.132820
GOTERM_BP_FAT	GO:0048542 lymph gland development	3	0.135501	0.070416	146	24	7937	6.795377	1.000000	0.911080	69.132820
GOTERM_BP_FAT	GO:0002784 regulation of antimicrobial peptide production	3	0.135501	0.070416	146	24	7937	6.795377	1.000000	0.911080	69.132820
GOTERM_BP_FAT	GO:0002700 regulation of production of molecular mediator of immune response	3	0.135501	0.070416	146	24	7937	6.795377	1.000000	0.911080	69.132820
GOTERM_BP_FAT	GO:0002807 positive regulation of antimicrobial peptide biosynthetic process	3	0.135501	0.070416	146	24	7937	6.795377	1.000000	0.911080	69.132820
GOTERM_BP_FAT	GO:0002805 regulation of antimicrobial peptide biosynthetic process	3	0.135501	0.070416	146	24	7937	6.795377	1.000000	0.911080	69.132820
INTERPRO	IPR000412:ABC-2	2	0.090334	0.070656	247	3	10196	27.519568	1.000000	0.993954	65.062218
SP_PIR_KEYWORDS	alternative splicing	20	0.903342	0.074191	281	616	12980	1.499746	0.999997	0.883003	60.844474
INTERPRO	IPR008753:Peptidase M13	3	0.135501	0.075757	247	19	10196	6.517792	1.000000	0.991816	67.715887
SP_PIR_KEYWORDS	actin-binding	4	0.180668	0.076536	281	46	12980	4.016711	0.999998	0.850371	62.033920
GOTERM_BP_FAT	GO:0007422 peripheral nervous system development	5	0.225836	0.077001	146	88	7937	3.088808	1.000000	0.924369	72.471015
SP_PIR_KEYWORDS	zinc-finger	12	0.542005	0.078481	281	314	12980	1.765306	0.999999	0.818439	62.995257
GOTERM_BP_FAT	GO:0007612 learning	4	0.180668	0.078829	146	55	7937	3.953674	1.000000	0.923788	73.335327
GOTERM_BP_FAT	GO:0051050 positive regulation of transport	3	0.135501	0.081002	146	26	7937	6.272655	1.000000	0.924120	74.330285
GOTERM_BP_FAT	GO:0030001 metal ion transport	7	0.316170	0.081379	146	165	7937	2.306310	1.000000	0.919916	74.499265
GOTERM_BP_FAT	GO:0016051 carbohydrate biosynthetic process	4	0.180668	0.082224	146	56	7937	3.883072	1.000000	0.916946	74.874372
GOTERM_BP_FAT	GO:0007010 cytoskeleton organization	14	0.632340	0.082321	146	465	7937	1.636736	1.000000	0.912013	74.917095
SP_PIR_KEYWORDS	nucleotide-binding	23	1.038844	0.082367	281	743	12980	1.429906	0.999999	0.797089	64.848816
GOTERM_MF_FAT	GO:0008374 O-acyltransferase activity	3	0.135501	0.084086	176	22	7918	6.134814	1.000000	0.762425	70.069942
GOTERM_MF_FAT	GO:0004104 cholinesterase activity	2	0.090334	0.085534	176	4	7918	22.494318	1.000000	0.753289	70.713082
GOTERM_MF_FAT	GO:0004616 phosphogluconate dehydrogenase (decarboxylating) activity	2	0.090334	0.085534	176	4	7918	22.494318	1.000000	0.753289	70.713082
GOTERM_BP_FAT	GO:0045448 mitotic cell cycle, embryonic	3	0.135501	0.086468	146	27	7937	6.040335	1.000000	0.917733	76.680956

GOTERM_BP_FAT	GO:0045475 locomotor rhythm	3	0.135501	0.086468	146	27	7937	6.040335	1.000000	0.917733	76.680956
GOTERM_BP_FAT	GO:0016310 phosphorylation	13	0.587173	0.087855	146	425	7937	1.662869	1.000000	0.916315	77.244529
GOTERM_BP_FAT	GO:0006812 cation transport	9	0.406504	0.090076	146	252	7937	1.941536	1.000000	0.916972	78.120101
SP_PIR_KEYWORDS	cytoplasm	15	0.677507	0.090114	281	436	12980	1.589180	1.000000	0.793421	68.293029
GOTERM_BP_FAT	GO:0032989 cellular component morphogenesis	15	0.677507	0.090378	146	518	7937	1.574219	1.000000	0.912997	78.236549
GOTERM_BP_FAT	GO:0042752 regulation of circadian rhythm	3	0.135501	0.092042	146	28	7937	5.824609	1.000000	0.912396	78.868931
INTERPRO	IPR018121:Seven in absentia protein, TRAF-like domain	2	0.090334	0.093085	247	4	10196	20.639676	1.000000	0.994998	75.394613
GOTERM_BP_FAT	GO:0048569 post-embryonic organ development	10	0.451671	0.093472	146	297	7937	1.830405	1.000000	0.911258	79.398446
GOTERM_BP_FAT	GO:0010324 membrane invagination	9	0.406504	0.099754	146	258	7937	1.896384	1.000000	0.921104	81.580334
GOTERM_BP_FAT	GO:0006897 endocytosis	9	0.406504	0.099754	146	258	7937	1.896384	1.000000	0.921104	81.580334

Table 11: Architecture 11

Category	Term	Count	%	PValue	List Total	Pop Hits	Pop Total	Fold Enrichment	Bonferroni	Benjamini	FDR
GOTERM_MF_FAT	GO:0004222 metalloendopeptidase activity	8	1.476015	8.890791e-04	155	80	7918	5.108387	0.268820	0.268820	1.210164
INTERPRO	IPR018497:Peptidase M13, neprilysin, C-terminal	5	0.922509	0.001963	224	25	10196	9.103571	0.550634	0.550634	2.710851
INTERPRO	IPR000718:Peptidase M13, neprilysin	5	0.922509	0.002633	224	27	10196	8.429233	0.657967	0.415164	3.618873
GOTERM_BP_FAT	GO:0016052 carbohydrate catabolic process	7	1.291513	0.004266	148	83	7937	4.522875	0.987550	0.987550	6.551793
GOTERM_BP_FAT	GO:0055114 oxidation reduction	22	4.059041	0.004882	148	620	7937	1.902942	0.993406	0.918799	7.464992
INTERPRO	IPR008753:Peptidase M13	4	0.738007	0.007721	224	19	10196	9.582707	0.957355	0.650626	10.272283
GOTERM_BP_FAT	GO:0006873 cellular ion homeostasis	5	0.922509	0.008633	148	44	7937	6.094134	0.999863	0.948452	12.841017
GOTERM_MF_FAT	GO:0003869 4-nitrophenylphosphatase activity	3	0.553506	0.012370	155	9	7918	17.027957	0.987490	0.888152	15.665107
GOTERM_BP_FAT	GO:0050801 ion homeostasis	5	0.922509	0.013451	148	50	7937	5.362838	0.999999	0.968993	19.318488
GOTERM_BP_FAT	GO:0055082 cellular chemical homeostasis	5	0.922509	0.014390	148	51	7937	5.257684	1.000000	0.948921	20.528036
INTERPRO	IPR006357:HAD-superfamily hydrolase, subfamily IIA	3	0.553506	0.015493	224	9	10196	15.172619	0.998262	0.795809	19.614106
GOTERM_BP_FAT	GO:0008202 steroid metabolic process	4	0.738007	0.015991	148	29	7937	7.397018	1.000000	0.936495	22.549830
GOTERM_MF_FAT	GO:0008237 metallopeptidase activity	9	1.660517	0.016553	155	168	7918	2.736636	0.997192	0.858925	20.425674
GOTERM_BP_FAT	GO:0019725 cellular homeostasis	7	1.291513	0.016822	148	111	7937	3.381970	1.000000	0.916803	23.579151
PIR_SUPERFAMILY	PIRSF001552:4-coumarate-CoA ligase	3	0.553506	0.018586	52	11	2596	13.615385	0.669418	0.669418	17.084190
GOTERM_BP_FAT	GO:0006026 aminoglycan catabolic process	4	0.738007	0.020853	148	32	7937	6.703547	1.000000	0.932971	28.397445
GOTERM_BP_FAT	GO:0000272 polysaccharide catabolic process	4	0.738007	0.022634	148	33	7937	6.500410	1.000000	0.926456	30.434129
GOTERM_BP_FAT	GO:0008610 lipid biosynthetic process	7	1.291513	0.022885	148	119	7937	3.154610	1.000000	0.907015	30.717662
KEGG_PATHWAY	dme00052:Galactose metabolism	4	0.738007	0.024127	46	29	2054	6.158921	0.697811	0.697811	20.876663
KEGG_PATHWAY	dme00010:Glycolysis / Gluconeogenesis	5	0.922509	0.025302	46	52	2054	4.293478	0.715139	0.466276	21.785633
KEGG_PATHWAY	dme00520:Amino sugar and nucleotide sugar metabolism	5	0.922509	0.025302	46	52	2054	4.293478	0.715139	0.466276	21.785633
INTERPRO	IPR007110:Immunoglobulin-like	8	1.476015	0.025725	224	132	10196	2.758658	0.999975	0.880143	30.541018
GOTERM_BP_FAT	GO:0048878 chemical homeostasis	5	0.922509	0.033602	148	66	7937	4.062756	1.000000	0.958745	41.829346
INTERPRO	IPR018499:Tetraspanin	4	0.738007	0.034788	224	33	10196	5.517316	0.999999	0.909444	39.049559
SP_PIR_KEYWORDS	oxidoreductase	19	3.505535	0.040746	267	561	12980	1.646471	0.997391	0.997391	38.899628
GOTERM_CC_FAT	GO:0031224 intrinsic to membrane	36	6.642066	0.047785	95	1382	4786	1.312331	0.999790	0.999790	45.070317
GOTERM_MF_FAT	GO:0005539 glycosaminoglycan binding	3	0.553506	0.051772	155	19	7918	8.065874	1.000000	0.990704	51.697420
GOTERM_BP_FAT	GO:0030005 cellular di-, tri-valent inorganic cation homeostasis	3	0.553506	0.056864	148	21	7937	7.661197	1.000000	0.993300	60.466426
GOTERM_MF_FAT	GO:0016019 peptidoglycan receptor activity	2	0.369004	0.057228	155	3	7918	34.055914	1.000000	0.984215	55.365466
GOTERM_BP_FAT	GO:0016192 vesicle-mediated transport	13	2.398524	0.058414	148	391	7937	1.783041	1.000000	0.991351	61.483817
GOTERM_CC_FAT	GO:0016021 integral to membrane	35	6.457565	0.060775	95	1360	4786	1.296517	0.999981	0.995589	53.568657
SP_PIR_KEYWORDS	membrane	32	5.904059	0.062774	267	1139	12980	1.365808	0.999906	0.990298	53.595913
KEGG_PATHWAY	dme00561:Glycerolipid metabolism	4	0.738007	0.069727	46	44	2054	4.059289	0.971033	0.692886	49.992447
GOTERM_BP_FAT	GO:0055066 di-, tri-valent inorganic cation homeostasis	3	0.553506	0.072117	148	24	7937	6.703547	1.000000	0.995853	69.470309
GOTERM_BP_FAT	GO:0002700 regulation of production of molecular mediator of immune response	3	0.553506	0.072117	148	24	7937	6.703547	1.000000	0.995853	69.470309
GOTERM_BP_FAT	GO:0002784 regulation of antimicrobial peptide production	3	0.553506	0.072117	148	24	7937	6.703547	1.000000	0.995853	69.470309
GOTERM_BP_FAT	GO:0030855 epithelial cell differentiation	3	0.553506	0.072117	148	24	7937	6.703547	1.000000	0.995853	69.470309
GOTERM_BP_FAT	GO:0002807 positive regulation of antimicrobial peptide biosynthetic process	3	0.553506	0.072117	148	24	7937	6.703547	1.000000	0.995853	69.470309
GOTERM_BP_FAT	GO:0002805 regulation of antimicrobial peptide biosynthetic process	3	0.553506	0.072117	148	24	7937	6.703547	1.000000	0.995853	69.470309
GOTERM_BP_FAT	GO:0002697 regulation of immune effector process	3	0.553506	0.072117	148	24	7937	6.703547	1.000000	0.995853	69.470309
GOTERM_MF_FAT	GO:0016877 ligase activity, forming carbon-sulfur bonds	3	0.553506	0.072861	155	23	7918	6.663114	1.000000	0.988183	64.496937
GOTERM_BP_FAT	GO:0006909 phagocytosis	8	1.476015	0.072965	148	197	7937	2.177802	1.000000	0.994385	69.909770
GOTERM_BP_FAT	GO:0006631 fatty acid metabolic process	4	0.738007	0.074596	148	53	7937	4.047425	1.000000	0.993066	70.737785
GOTERM_MF_FAT	GO:0033764 steroid dehydrogenase activity, acting on the CH-OH group of donors, NAD or NADP as acceptor	2	0.369004	0.075571	155	4	7918	25.541935	1.000000	0.980772	65.891323
GOTERM_BP_FAT	GO:0042592 homeostatic process	7	1.291513	0.081838	148	163	7937	2.303059	1.000000	0.994218	74.164409
GOTERM_BP_FAT	GO:0030003 cellular cation homeostasis	3	0.553506	0.082933	148	26	7937	6.187890	1.000000	0.992808	74.648282
INTERPRO	IPR019402: Frag1/DRAM/ Sfk1	2	0.369004	0.084669	224	4	10196	22.758929	1.000000	0.994165	70.977263
INTERPRO	IPR001312:Hexokinase	2	0.369004	0.084669	224	4	10196	22.758929	1.000000	0.994165	70.977263

UP_SEQ_FEATURE	binding site:Substrate	4	0.738007	0.085916	66	48	2975	3.756313	1.000000	1.000000	67.383189
SP_PIR_KEYWORDS	glycolysis	3	0.553506	0.085980	267	24	12980	6.076779	0.999997	0.986232	65.517216
INTERPRO	IPR002401:Cytochrome P450, E-class, group I	5	0.922509	0.087917	224	77	10196	2.955705	1.000000	0.990737	72.384852
GOTERM_BP_FAT	GO:0007272 ensheathment of neurons	2	0.369004	0.089259	148	5	7937	21.451351	1.000000	0.993583	77.282792
GOTERM_BP_FAT	GO:0046504 glycerol ether biosynthetic process	2	0.369004	0.089259	148	5	7937	21.451351	1.000000	0.993583	77.282792
GOTERM_BP_FAT	GO:0019228 regulation of action potential in neuron	2	0.369004	0.089259	148	5	7937	21.451351	1.000000	0.993583	77.282792
GOTERM_BP_FAT	GO:0006637 acyl-CoA metabolic process	2	0.369004	0.089259	148	5	7937	21.451351	1.000000	0.993583	77.282792
GOTERM_BP_FAT	GO:0008366 axon ensheathment	2	0.369004	0.089259	148	5	7937	21.451351	1.000000	0.993583	77.282792
GOTERM_BP_FAT	GO:0016045 detection of bacterium	2	0.369004	0.089259	148	5	7937	21.451351	1.000000	0.993583	77.282792
GOTERM_BP_FAT	GO:0009595 detection of biotic stimulus	2	0.369004	0.089259	148	5	7937	21.451351	1.000000	0.993583	77.282792
SP_PIR_KEYWORDS	developmental protein	17	3.136531	0.089505	267	540	12980	1.530448	0.999998	0.964992	67.059610
GOTERM_MF_FAT	GO:0004396 hexokinase activity	2	0.369004	0.093560	155	5	7918	20.433548	1.000000	0.986728	73.935834
GOTERM_BP_FAT	GO:0002831 regulation of response to biotic stimulus	3	0.553506	0.094206	148	28	7937	5.745898	1.000000	0.993754	79.162117
GOTERM_BP_FAT	GO:0043900 regulation of multi-organism process	3	0.553506	0.094206	148	28	7937	5.745898	1.000000	0.993754	79.162117
GOTERM_BP_FAT	GO:0030203 glycosaminoglycan metabolic process	3	0.553506	0.094206	148	28	7937	5.745898	1.000000	0.993754	79.162117
GOTERM_BP_FAT	GO:0002759 regulation of antimicrobial humoral response	3	0.553506	0.094206	148	28	7937	5.745898	1.000000	0.993754	79.162117
GOTERM_BP_FAT	GO:0002920 regulation of humoral immune response	3	0.553506	0.094206	148	28	7937	5.745898	1.000000	0.993754	79.162117
GOTERM_BP_FAT	GO:0022411 cellular component disassembly	3	0.553506	0.094206	148	28	7937	5.745898	1.000000	0.993754	79.162117
GOTERM_CC_FAT	GO:0032156 septin cytoskeleton	2	0.369004	0.094459	95	5	4786	20.151579	1.000000	0.996726	70.301340
PIR_SUPERFAMILY	PIRSF006698:Septin	2	0.369004	0.094514	52	5	2596	19.969231	0.997142	0.946541	62.895161
KEGG_PATHWAY	dme00640:Propanoate metabolism	3	0.553506	0.095552	46	24	2054	5.581522	0.992709	0.707788	61.822574
GOTERM_CC_FAT	GO:0044459 plasma membrane part	13	2.398524	0.096923	95	405	4786	1.617102	1.000000	0.987836	71.275511
GOTERM_MF_FAT	GO:0009055 electron carrier activity	8	1.476015	0.098631	155	202	7918	2.023124	1.000000	0.982774	75.862631
GOTERM_BP_FAT	GO:0046486 glycerolipid metabolic process	4	0.738007	0.099458	148	60	7937	3.575225	1.000000	0.994013	80.996975

Table 12: Architecture 12

Category	Term	Count	%	PValue	List Total	Pop Hits	Pop Total	Fold Enrichment	Bonferroni	Benjamini	FDR
SP_PIR_KEYWORDS	cytoplasm	14	2.935010	1.049073e-04	114	436	12980	3.656044	0.014063	0.014063	0.122921
GOTERM_BP_FAT	GO:0007010 cytoskeleton organization	14	2.935010	2.748270e-04	74	465	7937	3.229236	0.198497	0.198497	0.421373
SP_PIR_KEYWORDS	nucleus	18	3.773585	0.001340	114	869	12980	2.358428	0.165637	0.086565	1.560294
GOTERM_BP_FAT	GO:0044257 cellular protein catabolic process	8	1.677149	0.001395	74	183	7937	4.688820	0.674818	0.429753	2.121023
GOTERM_BP_FAT	GO:0051603 proteolysis involved in cellular protein catabolic process	8	1.677149	0.001395	74	183	7937	4.688820	0.674818	0.429753	2.121023
SP_PIR_KEYWORDS	phosphoprotein	17	3.563941	0.001809	114	815	12980	2.374987	0.216862	0.078251	2.100452
GOTERM_BP_FAT	GO:0030163 protein catabolic process	8	1.677149	0.002318	74	200	7937	4.290270	0.845577	0.463499	3.502201
SP_PIR_KEYWORDS	zinc	13	2.725367	0.004381	114	577	12980	2.565295	0.447222	0.137741	5.017741
GOTERM_BP_FAT	GO:0044265 cellular macromolecule catabolic process	8	1.677149	0.004461	74	225	7937	3.813574	0.972648	0.593327	6.637733
SP_PIR_KEYWORDS	helicase	5	1.048218	0.004524	114	77	12980	7.393484	0.457789	0.115223	5.176802
GOTERM_CC_FAT	GO:0005819 spindle	5	1.048218	0.005253	51	68	4786	6.900231	0.585020	0.585020	6.205009
SMART	SM00487:DEXDc	5	1.048218	0.005369	47	75	4824	6.842553	0.260256	0.260256	5.176643
GOTERM_CC_FAT	GO:0019005 SCF ubiquitin ligase complex	3	0.628931	0.005540	51	11	4786	25.593583	0.604562	0.371161	6.533948
SMART	SM00490:HELICc	5	1.048218	0.005628	47	76	4824	6.752520	0.270973	0.146169	5.420322
GOTERM_BP_FAT	GO:0000226 microtubule cytoskeleton organization	9	1.886792	0.005799	74	298	7937	3.239298	0.990741	0.607974	8.547804
INTERPRO	IPR014021:Helicase, superfamily 1 and 2, ATP-binding	5	1.048218	0.005854	102	73	10196	6.846629	0.745391	0.745391	7.265447
INTERPRO	IPR014001:DEAD-like helicase, N-terminal	5	1.048218	0.006440	102	75	10196	6.664052	0.778063	0.528899	7.965012
INTERPRO	IPR001650:DNA/RNA helicase, C-terminal	5	1.048218	0.006747	102	76	10196	6.576367	0.793481	0.408911	8.329659
SP_PIR_KEYWORDS	alternative splicing	13	2.725367	0.007290	114	616	12980	2.402882	0.627609	0.151797	8.220727
GOTERM_MF_FAT	GO:0000166 nucleotide binding	21	4.402516	0.008386	77	1206	7918	1.790592	0.819044	0.819044	10.040285
INTERPRO	IPR011545:DNA/RNA helicase, DEAD/DEAH box type, N-terminal	4	0.838574	0.008739	102	43	10196	9.298678	0.870633	0.400270	10.663595
UP_SEQ_FEATURE	domain:Helicase ATP-binding	4	0.838574	0.009278	45	30	2975	8.814815	0.745935	0.745935	10.498737
UP_SEQ_FEATURE	domain:Helicase C-terminal	4	0.838574	0.009278	45	30	2975	8.814815	0.745935	0.745935	10.498737
SP_PIR_KEYWORDS	cell cycle	5	1.048218	0.009794	114	96	12980	5.930190	0.735185	0.172890	10.898084
GOTERM_BP_FAT	GO:0030036 actin cytoskeleton organization	6	1.257862	0.009969	74	143	7937	4.500284	0.999686	0.739272	14.266356
GOTERM_BP_FAT	GO:0030029 actin filament-based process	6	1.257862	0.010256	74	144	7937	4.469032	0.999751	0.694400	14.646203
COG_ONTOLOGY	Translation, ribosomal structure and biogenesis	5	1.048218	0.010574	20	59	1237	5.241525	0.100847	0.100847	6.183637
GOTERM_BP_FAT	GO:0034621 cellular macromolecular complex subunit organization	7	1.467505	0.010678	74	203	7937	3.698509	0.999823	0.660480	15.203792
GOTERM_MF_FAT	GO:0017076 purine nucleotide binding	18	3.773585	0.011157	77	989	7918	1.871548	0.897467	0.679792	13.148326
GOTERM_MF_FAT	GO:0032555 purine ribonucleotide binding	17	3.563941	0.012913	77	923	7918	1.893965	0.928529	0.585006	15.066869
GOTERM_MF_FAT	GO:0032553 ribonucleotide binding	17	3.563941	0.012913	77	923	7918	1.893965	0.928529	0.585006	15.066869
GOTERM_BP_FAT	GO:0009057 macromolecule catabolic process	8	1.677149	0.013350	74	277	7937	3.097668	0.999980	0.699432	18.654489
GOTERM_BP_FAT	GO:0048707 instar larval or pupal morphogenesis	9	1.886792	0.013892	74	347	7937	2.781876	0.999987	0.675727	19.339232
GOTERM_BP_FAT	GO:0002165 instar larval or pupal development	10	2.096436	0.013920	74	418	7937	2.565951	0.999987	0.641503	19.373838
GOTERM_BP_FAT	GO:0043933 macromolecular complex subunit organization	8	1.677149	0.014353	74	281	7937	3.053573	0.999991	0.620859	19.916691
GOTERM_BP_FAT	GO:0051276 chromosome organization	8	1.677149	0.014613	74	282	7937	3.042745	0.999993	0.598092	20.239647
UP_SEQ_FEATURE	short sequence motif:Q motif	3	0.628931	0.015038	45	13	2975	15.256410	0.892193	0.671660	16.499128
GOTERM_BP_FAT	GO:0048563 post-embryonic organ morphogenesis	8	1.677149	0.015141	74	284	7937	3.021317	0.999995	0.584084	20.894345
GOTERM_BP_FAT	GO:0007560 imaginal disc morphogenesis	8	1.677149	0.015141	74	284	7937	3.021317	0.999995	0.584084	20.894345
GOTERM_BP_FAT	GO:0009886 post-embryonic morphogenesis	9	1.886792	0.015273	74	353	7937	2.734592	0.999996	0.562202	21.057441
GOTERM_BP_FAT	GO:0007426 tracheal outgrowth, open tracheal system	3	0.628931	0.015651	74	21	7937	15.322394	0.999997	0.547817	21.521213
GOTERM_MF_FAT	GO:0008270 zinc ion binding	18	3.773585	0.015746	77	1026	7918	1.804056	0.960124	0.553133	18.079541
GOTERM_CC_FAT	GO:0031461 cullin-RING ubiquitin ligase complex	3	0.628931	0.016332	51	19	4786	14.817337	0.936067	0.600140	18.150315
SP_PIR_KEYWORDS	cell division	4	0.838574	0.016749	114	62	12980	7.345784	0.897749	0.248017	17.965380
GOTERM_BP_FAT	GO:0009791 post-embryonic development	10	2.096436	0.017415	74	434	7937	2.471354	0.999999	0.564784	23.654074
GOTERM_BP_FAT	GO:0007552 metamorphosis	9	1.886792	0.017530	74	362	7937	2.666604	0.999999	0.546577	23.791249
GOTERM_BP_FAT	GO:0034622 cellular macromolecular complex assembly	6	1.257862	0.017634	74	165	7937	3.900246	0.999999	0.529423	23.915315
GOTERM_BP_FAT	GO:0048569 post-embryonic organ development	8	1.677149	0.018916	74	297	7937	2.889071	1.000000	0.536361	25.425924
GOTERM_BP_FAT	GO:0019941 modification-dependent protein catabolic process	6	1.257862	0.019356	74	169	7937	3.807932	1.000000	0.527280	25.938368
GOTERM_BP_FAT	GO:0043632 modification-dependent macromolecule catabolic process	6	1.257862	0.019803	74	170	7937	3.785533	1.000000	0.518998	26.455494
GOTERM_MF_FAT	GO:0004386 helicase activity	5	1.048218	0.020436	77	109	7918	4.717026	0.984875	0.567545	22.850295

SP_PIR_KEYWORDS	metal-binding	14	2.935010	0.020825	114	796	12980	2.002557	0.941634	0.270700	21.864174
GOTERM_BP_FAT	GO:0007281 germ cell development	7	1.467505	0.023896	74	243	7937	3.089701	1.000000	0.571096	31.034618
GOTERM_BP_FAT	GO:0006396 RNA processing	8	1.677149	0.024046	74	312	7937	2.750173	1.000000	0.557983	31.197270
GOTERM_BP_FAT	GO:0007049 cell cycle	12	2.515723	0.024249	74	616	7937	2.089417	1.000000	0.546351	31.416227
GOTERM_BP_FAT	GO:0065003 macromolecular complex assembly	7	1.467505	0.025197	74	246	7937	3.052022	1.000000	0.546213	32.432804
GOTERM_CC_FAT	GO:0043228 non-membrane-bounded organelle	17	3.563941	0.026442	51	943	4786	1.691764	0.988612	0.673327	27.815322
GOTERM_CC_FAT	GO:0043232 intracellular non-membrane-bounded organelle	17	3.563941	0.026442	51	943	4786	1.691764	0.988612	0.673327	27.815322
GOTERM_BP_FAT	GO:0016192 vesicle-mediated transport	9	1.886792	0.026444	74	391	7937	2.468826	1.000000	0.550233	33.748475
GOTERM_BP_FAT	GO:0008104 protein localization	9	1.886792	0.026799	74	392	7937	2.462528	1.000000	0.542043	34.118820
GOTERM_BP_FAT	GO:0022402 cell cycle process	11	2.306080	0.027237	74	547	7937	2.156900	1.000000	0.535385	34.572926
INTERPRO	IPR011989:Armadillo-like helical	4	0.838574	0.027536	102	66	10196	6.058229	0.998505	0.727784	30.142330
GOTERM_BP_FAT	GO:0006403 RNA localization	5	1.048218	0.027722	74	125	7937	4.290270	1.000000	0.529697	35.072322
GOTERM_MF_FAT	GO:0005525 GTP binding	6	1.257862	0.027806	77	178	7918	3.466219	0.996735	0.614836	29.834529
SP_PIR_KEYWORDS	nucleotide-binding	13	2.725367	0.028064	114	743	12980	1.992161	0.978567	0.319059	28.374399
GOTERM_BP_FAT	GO:0030951 establishment or maintenance of microtubule cytoskeleton polarity	3	0.628931	0.028865	74	29	7937	11.095527	1.000000	0.532613	36.235411
GOTERM_BP_FAT	GO:0016325 oocyte microtubule cytoskeleton organization	3	0.628931	0.028865	74	29	7937	11.095527	1.000000	0.532613	36.235411
GOTERM_BP_FAT	GO:0030952 establishment or maintenance of cytoskeleton polarity	3	0.628931	0.028865	74	29	7937	11.095527	1.000000	0.532613	36.235411
GOTERM_MF_FAT	GO:0032561 guanyl ribonucleotide binding	6	1.257862	0.029601	77	181	7918	3.408768	0.997757	0.581635	31.445569
GOTERM_MF_FAT	GO:0019001 guanyl nucleotide binding	6	1.257862	0.030216	77	182	7918	3.390039	0.998027	0.540929	31.989062
GOTERM_BP_FAT	GO:0007017 microtubule-based process	9	1.886792	0.032129	74	406	7937	2.377613	1.000000	0.560231	39.448895
GOTERM_BP_FAT	GO:0007052 mitotic spindle organization	6	1.257862	0.032642	74	194	7937	3.317219	1.000000	0.554940	39.940204
GOTERM_BP_FAT	GO:0046605 regulation of centrosome cycle	2	0.419287	0.036292	74	4	7937	53.628378	1.000000	0.583241	43.329068
GOTERM_BP_FAT	GO:0032507 maintenance of protein location in cell	3	0.628931	0.036668	74	33	7937	9.750614	1.000000	0.576508	43.668001
GOTERM_BP_FAT	GO:0008293 torso signaling pathway	3	0.628931	0.036668	74	33	7937	9.750614	1.000000	0.576508	43.668001
GOTERM_MF_FAT	GO:0008026 ATP-dependent helicase activity	4	0.838574	0.038710	77	78	7918	5.273393	0.999669	0.589540	39.106122
GOTERM_MF_FAT	GO:0070035 purine NTP-dependent helicase activity	4	0.838574	0.038710	77	78	7918	5.273393	0.999669	0.589540	39.106122
GOTERM_BP_FAT	GO:0012502 induction of programmed cell death	3	0.628931	0.038732	74	34	7937	9.463831	1.000000	0.586592	45.494121
GOTERM_BP_FAT	GO:0008595 determination of anterior/posterior axis, embryo	5	1.048218	0.039610	74	140	7937	3.830598	1.000000	0.584931	46.253258
GOTERM_BP_FAT	GO:0007351 tripartite regional subdivision	5	1.048218	0.039610	74	140	7937	3.830598	1.000000	0.584931	46.253258
INTERPRO	IPR014014:RNA helicase, DEAD-box type, Q motif	3	0.628931	0.039745	102	32	10196	9.371324	0.999921	0.792982	40.609111
GOTERM_BP_FAT	GO:0043623 cellular protein complex assembly	4	0.838574	0.040646	74	83	7937	5.169000	1.000000	0.584817	47.137176
GOTERM_BP_FAT	GO:0051651 maintenance of location in cell	3	0.628931	0.040840	74	35	7937	9.193436	1.000000	0.577125	47.301462
GOTERM_BP_FAT	GO:0003006 reproductive developmental process	10	2.096436	0.041417	74	506	7937	2.119699	1.000000	0.573132	47.786668
GOTERM_BP_FAT	GO:0010564 regulation of cell cycle process	4	0.838574	0.041881	74	84	7937	5.107465	1.000000	0.568293	48.172946
GOTERM_CC_FAT	GO:0005938 cell cortex	4	0.838574	0.042841	51	75	4786	5.004967	0.999333	0.768329	41.290556
SP_PIR_KEYWORDS	gtp-binding	5	1.048218	0.043688	114	152	12980	3.745383	0.997596	0.422033	40.768268
GOTERM_BP_FAT	GO:0000578 embryonic axis specification	5	1.048218	0.044130	74	145	7937	3.698509	1.000000	0.578970	50.010760
GOTERM_BP_FAT	GO:0045185 maintenance of protein location	3	0.628931	0.045182	74	37	7937	8.696494	1.000000	0.579182	50.849495
GOTERM_BP_FAT	GO:0000278 mitotic cell cycle	8	1.677149	0.045606	74	358	7937	2.396799	1.000000	0.574298	51.184040
GOTERM_BP_FAT	GO:0009948 anterior/posterior axis specification	5	1.048218	0.046016	74	147	7937	3.648189	1.000000	0.569462	51.505048
GOTERM_BP_FAT	GO:0007350 blastoderm segmentation	6	1.257862	0.049767	74	218	7937	2.952021	1.000000	0.590715	54.353003
KEGG_PATHWAY	dme04350:TGF-beta signaling pathway	3	0.628931	0.049968	23	34	2054	7.879795	0.659195	0.659195	32.685263
GOTERM_MF_FAT	GO:0046872 metal ion binding	24	5.031447	0.050299	77	1718	7918	1.436524	0.999972	0.649237	47.713412
GOTERM_BP_FAT	GO:0007242 intracellular signaling cascade	7	1.467505	0.054893	74	297	7937	2.527937	1.000000	0.619772	57.992950
GOTERM_BP_FAT	GO:0007051 spindle organization	6	1.257862	0.055602	74	225	7937	2.860180	1.000000	0.616883	58.474180
GOTERM_MF_FAT	GO:0008186 RNA-dependent ATPase activity	3	0.628931	0.056101	77	40	7918	7.712338	0.999992	0.655443	51.588279
GOTERM_MF_FAT	GO:0004004 ATP-dependent RNA helicase activity	3	0.628931	0.056101	77	40	7918	7.712338	0.999992	0.655443	51.588279
SP_PIR_KEYWORDS	zinc-finger	7	1.467505	0.056367	114	314	12980	2.538272	0.999603	0.479364	49.347854
SP_PIR_KEYWORDS	protein transport	4	0.838574	0.057995	114	101	12980	4.509293	0.999686	0.462286	50.362917
GOTERM_BP_FAT	GO:0006511 ubiquitin-dependent protein catabolic process	4	0.838574	0.058145	74	96	7937	4.469032	1.000000	0.626235	60.159007
GOTERM_BP_FAT	GO:0051297 centrosome organization	3	0.628931	0.059154	74	43	7937	7.483030	1.000000	0.625332	60.810130

GOTERM_BP_FAT	GO:0009880 embryonic pattern specification	6	1.257862	0.060908	74	231	7937	2.785890	1.000000	0.629133	61.917354
GOTERM_BP_FAT	GO:0007276 gamete generation	12	2.515723	0.060997	74	715	7937	1.800113	1.000000	0.622543	61.972558
GOTERM_BP_FAT	GO:0007346 regulation of mitotic cell cycle	4	0.838574	0.061108	74	98	7937	4.377827	1.000000	0.616232	62.041742
GOTERM_BP_FAT	GO:0008360 regulation of cell shape	4	0.838574	0.061108	74	98	7937	4.377827	1.000000	0.616232	62.041742
GOTERM_BP_FAT	GO:0010627 regulation of protein kinase cascade	3	0.628931	0.061612	74	44	7937	7.312961	1.000000	0.612479	62.353561
GOTERM_MF_FAT	GO:0004518 nuclease activity	4	0.838574	0.062839	77	95	7918	4.329733	0.999998	0.666428	55.755570
GOTERM_BP_FAT	GO:0043068 positive regulation of programmed cell death	3	0.628931	0.064104	74	45	7937	7.150450	1.000000	0.620792	63.860506
GOTERM_BP_FAT	GO:0010942 positive regulation of cell death	3	0.628931	0.064104	74	45	7937	7.150450	1.000000	0.620792	63.860506
GOTERM_CC_FAT	GO:0000151 ubiquitin ligase complex	3	0.628931	0.064878	51	40	4786	7.038235	0.999986	0.845415	55.775111
SMART	SM00449:SPRY	2	0.419287	0.064909	47	7	4824	29.325228	0.976676	0.714283	48.453574
GOTERM_MF_FAT	GO:0043169 cation binding	24	5.031447	0.065021	77	1764	7918	1.399064	0.999999	0.650008	57.032664
GOTERM_BP_FAT	GO:0051726 regulation of cell cycle	5	1.048218	0.066156	74	166	7937	3.230625	1.000000	0.626152	65.058819
GOTERM_MF_FAT	GO:0004540 ribonuclease activity	3	0.628931	0.066407	77	44	7918	7.011216	0.999999	0.630782	57.826166
GOTERM_BP_FAT	GO:0009968 negative regulation of signal transduction	4	0.838574	0.067243	74	102	7937	4.206147	1.000000	0.625849	65.678462
GOTERM_BP_FAT	GO:0006461 protein complex assembly	5	1.048218	0.067326	74	167	7937	3.211280	1.000000	0.619922	65.725289
GOTERM_BP_FAT	GO:0070271 protein complex biogenesis	5	1.048218	0.067326	74	167	7937	3.211280	1.000000	0.619922	65.725289
INTERPRO	IPR018355:SPla/RYanodine receptor subgroup	2	0.419287	0.067333	102	7	10196	28.560224	1.000000	0.901752	59.160685
GOTERM_MF_FAT	GO:0043167 ion binding	24	5.031447	0.067870	77	1772	7918	1.392747	0.999999	0.613710	58.649061
GOTERM_BP_FAT	GO:0010648 negative regulation of cell communication	4	0.838574	0.068819	74	103	7937	4.165311	1.000000	0.621996	66.558899
GOTERM_BP_FAT	GO:0051235 maintenance of location	3	0.628931	0.069188	74	47	7937	6.846176	1.000000	0.617853	66.761821
GOTERM_BP_FAT	GO:0031023 microtubule organizing center organization	3	0.628931	0.069188	74	47	7937	6.846176	1.000000	0.617853	66.761821
GOTERM_BP_FAT	GO:0009952 anterior/posterior pattern formation	5	1.048218	0.070900	74	170	7937	3.154610	1.000000	0.621098	67.688727
GOTERM_BP_FAT	GO:0019953 sexual reproduction	12	2.515723	0.071461	74	735	7937	1.751131	1.000000	0.618127	67.987213
GOTERM_BP_FAT	GO:0002168 instar larval development	3	0.628931	0.071779	74	48	7937	6.703547	1.000000	0.613938	68.154992
SP_PIR_KEYWORDS	mitosis	3	0.628931	0.072708	114	51	12980	6.697626	0.999962	0.517079	58.727708
GOTERM_MF_FAT	GO:0042803 protein homodimerization activity	3	0.628931	0.074517	77	47	7918	6.563692	1.000000	0.625632	62.204985
GOTERM_BP_FAT	GO:0007264 small GTPase mediated signal transduction	4	0.838574	0.078628	74	109	7937	3.936028	1.000000	0.643007	71.579585
GOTERM_MF_FAT	GO:0046983 protein dimerization activity	4	0.838574	0.079617	77	105	7918	3.917378	1.000000	0.628683	64.739735
GOTERM_MF_FAT	GO:0003724 RNA helicase activity	3	0.628931	0.080091	77	49	7918	6.295786	1.000000	0.609950	64.967658
KEGG_PATHWAY	dme04120:Ubiquitin mediated proteolysis	4	0.838574	0.082052	23	97	2054	3.682654	0.834356	0.593006	48.369805
GOTERM_CC_FAT	GO:0044448 cell cortex part	3	0.628931	0.082788	51	46	4786	6.120205	0.999999	0.872755	65.044454
GOTERM_BP_FAT	GO:0007444 imaginal disc development	8	1.677149	0.083447	74	412	7937	2.082655	1.000000	0.660112	73.779416
GOTERM_BP_FAT	GO:0016568 chromatin modification	4	0.838574	0.083751	74	112	7937	3.830598	1.000000	0.655902	73.912565
SP_PIR_KEYWORDS	endosome	2	0.419287	0.083752	114	10	12980	22.771930	0.999993	0.544891	64.136734
SP_PIR_KEYWORDS	hydrolase	19	3.983229	0.084854	114	1470	12980	1.471655	0.999994	0.526768	64.639092
GOTERM_BP_FAT	GO:0048610 reproductive cellular process	9	1.886792	0.087158	74	500	7937	1.930622	1.000000	0.665685	75.363789
GOTERM_MF_FAT	GO:0046914 transition metal ion binding	19	3.983229	0.087696	77	1354	7918	1.442978	1.000000	0.624922	68.437467
GOTERM_BP_FAT	GO:0035282 segmentation	6	1.257862	0.090487	74	260	7937	2.475156	1.000000	0.674637	76.708600
SP_PIR_KEYWORDS	Fatty acid biosynthesis	2	0.419287	0.091735	114	11	12980	20.701754	0.999998	0.534244	67.633491
GOTERM_BP_FAT	GO:0007059 chromosome segregation	4	0.838574	0.092599	74	117	7937	3.666898	1.000000	0.678149	77.525846
GOTERM_CC_FAT	GO:0005694 chromosome	7	1.467505	0.094788	51	304	4786	2.160862	1.000000	0.874927	70.218361
GOTERM_BP_FAT	GO:0008610 lipid biosynthetic process	4	0.838574	0.096244	74	119	7937	3.605269	1.000000	0.687690	78.873429
GOTERM_BP_FAT	GO:0022604 regulation of cell morphogenesis	4	0.838574	0.096244	74	119	7937	3.605269	1.000000	0.687690	78.873429
GOTERM_BP_FAT	GO:0042127 regulation of cell proliferation	3	0.628931	0.096414	74	57	7937	5.645092	1.000000	0.683202	78.934163
GOTERM_BP_FAT	GO:0035081 induction of programmed cell death by hormones	2	0.419287	0.096703	74	11	7937	19.501229	1.000000	0.679255	79.037638
GOTERM_BP_FAT	GO:0048609 reproductive process in a multicellular organism	12	2.515723	0.097752	74	778	7937	1.654346	1.000000	0.678367	79.408418
GOTERM_BP_FAT	GO:0032504 multicellular organism reproduction	12	2.515723	0.097752	74	778	7937	1.654346	1.000000	0.678367	79.408418
UP_SEQ_FEATURE	domain:UBA	2	0.419287	0.099143	45	7	2975	18.888889	1.000000	0.994000	71.132542
GOTERM_CC_FAT	GO:0030496 midbody	2	0.419287	0.099784	51	10	4786	18.768627	1.000000	0.857806	72.157168
GOTERM_BP_FAT	GO:0007308 oocyte construction	4	0.838574	0.099948	74	121	7937	3.545678	1.000000	0.681942	80.164973

Table 13: Architecture 13

Category	Term	Count	%	PValue	List Total	Pop Hits	Pop Total	Fold Enrichment	Bonferroni	Benjamini	FDR
GOTERM_CC_FAT	GO:0005938 cell cortex	6	1.534527	7.315066e-05	30	75	4786	12.762667	0.008958	0.008958	0.084315
GOTERM_BP_FAT	GO:0034470 ncRNA processing	6	1.534527	2.167254e-04	49	92	7937	10.563886	0.121377	0.121377	0.319391
GOTERM_BP_FAT	GO:0034660 ncRNA metabolic process	6	1.534527	0.001177	49	133	7937	7.307350	0.504996	0.296435	1.723411
SP_PIR_KEYWORDS	phosphoprotein	13	3.324808	0.001434	72	815	12980	2.875596	0.109711	0.109711	1.517351
SP_PIR_KEYWORDS	cell cycle	5	1.278772	0.001869	72	96	12980	9.389468	0.140633	0.072980	1.974349
GOTERM_BP_FAT	GO:0006399 tRNA metabolic process	5	1.278772	0.002411	49	94	7937	8.615936	0.763263	0.381383	3.499291
GOTERM_BP_FAT	GO:0008033 tRNA processing	4	1.023018	0.002793	49	47	7937	13.785497	0.811715	0.341275	4.044046
SP_PIR_KEYWORDS	zinc-finger	7	1.790281	0.007325	72	314	12980	4.018931	0.448738	0.180053	7.536580
GOTERM_BP_FAT	GO:0051301 cell division	6	1.534527	0.007609	49	205	7937	4.740866	0.989535	0.598260	10.660528
INTERPRO	IPR017907:Zinc finger, RING-type, conserved site	4	1.023018	0.008067	62	69	10196	9.533427	0.698443	0.698443	9.199235
SP_PIR_KEYWORDS	cytoplasm	8	2.046036	0.009615	72	436	12980	3.307849	0.542763	0.177691	9.783891
GOTERM_BP_FAT	GO:0016310 phosphorylation	8	2.046036	0.013179	49	425	7937	3.049028	0.999637	0.732884	17.783176
GOTERM_BP_FAT	GO:0006796 phosphate metabolic process	9	2.301790	0.016408	49	551	7937	2.645765	0.999949	0.756093	21.664954
GOTERM_BP_FAT	GO:0006793 phosphorus metabolic process	9	2.301790	0.016408	49	551	7937	2.645765	0.999949	0.756093	21.664954
SMART	SM00252:SH2	3	0.767263	0.016419	30	33	4824	14.618182	0.458038	0.458038	13.813438
SP_PIR_KEYWORDS	ubl conjugation pathway	4	1.023018	0.016472	72	98	12980	7.358277	0.739552	0.235910	16.222902
INTERPRO	IPR000980:SH2 motif	3	0.767263	0.016503	62	33	10196	14.950147	0.914812	0.708130	17.984488
SP_PIR_KEYWORDS	nucleus	11	2.813299	0.019277	72	869	12980	2.281997	0.793346	0.231093	18.734656
GOTERM_BP_FAT	GO:0051276 chromosome organization	6	1.534527	0.026904	49	282	7937	3.446374	1.000000	0.869352	33.137068
GOTERM_BP_FAT	GO:0007049 cell cycle	9	2.301790	0.029872	49	616	7937	2.366585	1.000000	0.866242	36.084407
SP_PIR_KEYWORDS	coiled coil	5	1.278772	0.030046	72	215	12980	4.192506	0.915505	0.297430	27.756170
GOTERM_CC_FAT	GO:0044448 cell cortex part	3	0.767263	0.031125	30	46	4786	10.404348	0.979539	0.856959	30.552333
GOTERM_BP_FAT	GO:0000226 microtubule cytoskeleton organization	6	1.534527	0.033073	49	298	7937	3.261334	1.000000	0.865721	39.126939
GOTERM_CC_FAT	GO:0000775 chromosome, centromeric region	3	0.767263	0.036296	30	50	4786	9.572000	0.989406	0.780370	34.707864
GOTERM_BP_FAT	GO:0006396 RNA processing	6	1.534527	0.039148	49	312	7937	3.114992	1.000000	0.885523	44.534229
GOTERM_BP_FAT	GO:0010608 posttranscriptional regulation of gene expression	4	1.023018	0.040264	49	126	7937	5.142209	1.000000	0.870563	45.477162
GOTERM_BP_FAT	GO:0045995 regulation of embryonic development	3	0.767263	0.041992	49	54	7937	8.998866	1.000000	0.860552	46.908546
SMART	SM00184:RING	4	1.023018	0.044413	30	133	4824	4.836090	0.813792	0.568482	33.496687
GOTERM_BP_FAT	GO:0022402 cell cycle process	8	2.046036	0.044647	49	547	7937	2.368989	1.000000	0.857395	49.039003
INTERPRO	IPR001841:Zinc finger, RING-type	4	1.023018	0.045159	62	133	10196	4.945913	0.998929	0.897687	42.336713
SP_PIR_KEYWORDS	cell division	3	0.767263	0.045195	72	62	12980	8.723118	0.976390	0.373909	38.913480
GOTERM_BP_FAT	GO:0006119 oxidative phosphorylation	4	1.023018	0.045205	49	132	7937	4.908472	1.000000	0.841354	49.476510
GOTERM_BP_FAT	GO:0000902 cell morphogenesis	7	1.790281	0.048841	49	442	7937	2.565288	1.000000	0.845628	52.243201
GOTERM_BP_FAT	GO:0042775 mitochondrial ATP synthesis coupled electron transport	3	0.767263	0.061769	49	67	7937	7.252818	1.000000	0.893443	60.977400
GOTERM_BP_FAT	GO:0000278 mitotic cell cycle	6	1.534527	0.063725	49	358	7937	2.714742	1.000000	0.887397	62.160918
GOTERM_BP_FAT	GO:0042773 ATP synthesis coupled electron transport	3	0.767263	0.068386	49	71	7937	6.844208	1.000000	0.892014	64.847804
SP_PIR_KEYWORDS	SH2 domain	2	0.511509	0.068853	72	13	12980	27.735043	0.996906	0.473782	53.245926
KEGG_PATHWAY	dme00190:Oxidative phosphorylation	4	1.023018	0.070611	16	135	2054	3.803704	0.751254	0.751254	42.235706
GOTERM_BP_FAT	GO:0022904 respiratory electron transport chain	3	0.767263	0.073496	49	74	7937	6.566740	1.000000	0.897578	67.588786
GOTERM_MF_FAT	GO:0004715 non-membrane spanning protein tyrosine kinase activity	2	0.511509	0.074662	52	12	7918	25.378205	0.999998	0.999998	61.232952
GOTERM_BP_FAT	GO:0051656 establishment of organelle localization	3	0.767263	0.075226	49	75	7937	6.479184	1.000000	0.891748	68.470814
INTERPRO	IPR018957:Zinc finger, C3HC4 RING-type	3	0.767263	0.079186	62	78	10196	6.325062	0.999995	0.952755	62.576424
GOTERM_CC_FAT	GO:0031981 nuclear lumen	6	1.534527	0.079931	30	387	4786	2.473385	0.999965	0.922826	61.732934
GOTERM_BP_FAT	GO:0019941 modification-dependent protein catabolic process	4	1.023018	0.081840	49	169	7937	3.833836	1.000000	0.901431	71.639851
GOTERM_BP_FAT	GO:0043632 modification-dependent macromolecule catabolic process	4	1.023018	0.082969	49	170	7937	3.811285	1.000000	0.894409	72.150026
UP_SEQ_FEATURE	mutagenesis site	5	1.278772	0.083791	26	201	2975	2.846345	0.999842	0.999842	62.141306
GOTERM_BP_FAT	GO:0000910 cytokinesis	3	0.767263	0.084071	49	80	7937	6.074235	1.000000	0.887459	72.640123
INTERPRO	IPR006594:LisH dimerisation motif	2	0.511509	0.086135	62	15	10196	21.926882	0.999998	0.930481	65.805955
GOTERM_BP_FAT	GO:0008104 protein localization	6	1.534527	0.086491	49	392	7937	2.479279	1.000000	0.884702	73.687834
SMART	SM00667:LisH	2	0.511509	0.086597	30	15	4824	21.440000	0.964964	0.672783	55.661443
GOTERM_CC_FAT	GO:0005746 mitochondrial respiratory chain	3	0.767263	0.087443	30	82	4786	5.836585	0.999987	0.894707	65.184383
GOTERM_BP_FAT	GO:0051640 organelle localization	3	0.767263	0.087695	49	82	7937	5.926083	1.000000	0.878450	74.194989
SP_PIR_KEYWORDS	ligase	4	1.023018	0.088938	72	193	12980	3.736327	0.999471	0.529740	62.941434
GOTERM_BP_FAT	GO:0032989 cellular component morphogenesis	7	1.790281	0.090274	49	518	7937	2.188913	1.000000	0.876558	75.251166
GOTERM_BP_FAT	GO:0007062 sister chromatid cohesion	2	0.511509	0.092578	49	16	7937	20.247449	1.000000	0.873982	76.160267
GOTERM_CC_FAT	GO:0070469 respiratory chain	3	0.767263	0.092971	30	85	4786	5.630588	0.999994	0.864720	67.540282
INTERPRO	IPR017441:Protein kinase, ATP binding site	4	1.023018	0.095145	62	182	10196	3.614321	1.000000	0.915091	69.613067
SP_PIR_KEYWORDS	zinc	7	1.790281	0.095212	72	577	12980	2.187079	0.999698	0.521340	65.572472

GOTERM_BP_FAT	GO:0007017 microtubule-based process	6	1.534527	0.096981	49	406	7937	2.393787	1.000000	0.877549	77.811788
GOTERM_BP_FAT	GO:0007303 cytoplasmic transport, nurse cell to oocyte	2	0.511509	0.098077	49	17	7937	19.056423	1.000000	0.871805	78.205805
GOTERM_BP_FAT	GO:0016476 regulation of embryonic cell shape	2	0.511509	0.098077	49	17	7937	19.056423	1.000000	0.871805	78.205805
GOTERM_BP_FAT	GO:0044257 cellular protein catabolic process	4	1.023018	0.098246	49	183	7937	3.540538	1.000000	0.863516	78.266094
GOTERM_BP_FAT	GO:0051603 proteolysis involved in cellular protein catabolic process	4	1.023018	0.098246	49	183	7937	3.540538	1.000000	0.863516	78.266094

Table 14: Architecture 14

Category	Term	Count	%	PValue	List Total	Pop Hits	Pop Total	Fold Enrichment	Bonferroni	Benjamini	FDR
GOTERM_CC_FAT	GO:0031974 membrane-enclosed lumen	12	6.557377	2.897347e-04	30	571	4786	3.352715	0.033336	0.033336	0.330571
GOTERM_CC_FAT	GO:0070013 intracellular organelle lumen	11	6.010929	0.001056	30	556	4786	3.156235	0.116239	0.059914	1.199569
GOTERM_CC_FAT	GO:0043233 organelle lumen	11	6.010929	0.001056	30	556	4786	3.156235	0.116239	0.059914	1.199569
SP_PIR_KEYWORDS	transferase	12	6.557377	0.001267	58	873	12980	3.076194	0.111215	0.111215	1.378274
GOTERM_CC_FAT	GO:0031981 nuclear lumen	9	4.918033	0.001601	30	387	4786	3.710078	0.170979	0.060590	1.814626
GOTERM_MF_FAT	GO:0001882 nucleoside binding	12	6.557377	0.004444	45	824	7918	2.562460	0.528922	0.528922	5.283728
GOTERM_CC_FAT	GO:0043232 intracellular non-membrane-bounded organelle	13	7.103825	0.005982	30	943	4786	2.199293	0.504400	0.160960	6.626133
GOTERM_CC_FAT	GO:0043228 non-membrane-bounded organelle	13	7.103825	0.005982	30	943	4786	2.199293	0.504400	0.160960	6.626133
SP_PIR_KEYWORDS	Transcription	7	3.825137	0.007317	58	393	12980	3.986137	0.494894	0.289292	7.725064
GOTERM_MF_FAT	GO:0030554 adenyl nucleotide binding	11	6.010929	0.011910	45	812	7918	2.383634	0.867980	0.636654	13.586080
GOTERM_BP_FAT	GO:0033043 regulation of organelle organization	4	2.185792	0.011986	36	109	7937	8.090724	0.997787	0.997787	15.969085
GOTERM_BP_FAT	GO:0051301 cell division	5	2.732240	0.012094	36	205	7937	5.377371	0.997907	0.954250	16.101711
GOTERM_MF_FAT	GO:0001883 purine nucleoside binding	11	6.010929	0.012408	45	817	7918	2.369047	0.878770	0.505078	14.115803
SP_PIR_KEYWORDS	nucleus	10	5.464481	0.012807	58	869	12980	2.575295	0.698439	0.329408	13.161032
SP_PIR_KEYWORDS	activator	4	2.185792	0.013054	58	112	12980	7.992611	0.705371	0.263252	13.398403
SP_PIR_KEYWORDS	atp-binding	8	4.371585	0.013651	58	585	12980	3.060419	0.721480	0.225590	13.969734
SP_PIR_KEYWORDS	transit peptide	4	2.185792	0.013688	58	114	12980	7.852390	0.722465	0.192357	14.005598
GOTERM_BP_FAT	GO:0006350 transcription	7	3.825137	0.014039	36	459	7937	3.362321	0.999229	0.908312	18.453337
GOTERM_MF_FAT	GO:0017076 purine nucleotide binding	12	6.557377	0.016961	45	989	7918	2.134951	0.944481	0.514589	18.819187
GOTERM_MF_FAT	GO:0005524 ATP binding	10	5.464481	0.019820	45	748	7918	2.352347	0.966062	0.491680	21.650123
GOTERM_MF_FAT	GO:0032559 adenyl ribonucleotide binding	10	5.464481	0.020136	45	750	7918	2.346074	0.967863	0.436144	21.957567
GOTERM_BP_FAT	GO:0007411 axon guidance	4	2.185792	0.021596	36	136	7937	6.484477	0.999984	0.937170	27.022379
GOTERM_BP_FAT	GO:0016570 histone modification	3	1.639344	0.024240	36	55	7937	12.025758	0.999996	0.916946	29.816958
GOTERM_BP_FAT	GO:0016569 covalent chromatin modification	3	1.639344	0.024240	36	55	7937	12.025758	0.999996	0.916946	29.816958
GOTERM_MF_FAT	GO:0004674 protein serine/threonine kinase activity	5	2.732240	0.025601	45	203	7918	4.333881	0.987512	0.465348	27.100233
GOTERM_BP_FAT	GO:0006468 protein amino acid phosphorylation	5	2.732240	0.026225	36	259	7937	4.256221	0.999999	0.894135	31.848705
SP_PIR_KEYWORDS	transcription regulation	6	3.278689	0.027078	58	387	12980	3.469661	0.922150	0.305602	25.956842
GOTERM_MF_FAT	GO:0032553 ribonucleotide binding	11	6.010929	0.027217	45	923	7918	2.096978	0.990565	0.441733	28.559419
GOTERM_MF_FAT	GO:0032555 purine ribonucleotide binding	11	6.010929	0.027217	45	923	7918	2.096978	0.990565	0.441733	28.559419
GOTERM_MF_FAT	GO:0000166 nucleotide binding	13	7.103825	0.028045	45	1206	7918	1.896702	0.991831	0.413837	29.297788
GOTERM_BP_FAT	GO:0006793 phosphorus metabolic process	7	3.825137	0.031518	36	551	7937	2.800918	1.000000	0.901683	37.002931
GOTERM_BP_FAT	GO:0006796 phosphate metabolic process	7	3.825137	0.031518	36	551	7937	2.800918	1.000000	0.901683	37.002931
SP_PIR_KEYWORDS	chromatin regulator	3	1.639344	0.032104	58	64	12980	10.490302	0.951908	0.315681	30.038339
GOTERM_CC_FAT	GO:0005694 chromosome	6	3.278689	0.033724	30	304	4786	3.148684	0.981936	0.551912	32.430037
GOTERM_BP_FAT	GO:0016310 phosphorylation	6	3.278689	0.037071	36	425	7937	3.112549	1.000000	0.908738	42.018437
GOTERM_CC_FAT	GO:0005730 nucleolus	4	2.185792	0.039559	30	126	4786	5.064550	0.991106	0.544822	36.947878
SP_PIR_KEYWORDS	nucleotide-binding	8	4.371585	0.043046	58	743	12980	2.409616	0.983294	0.365341	38.226136
GOTERM_BP_FAT	GO:0006928 cell motion	5	2.732240	0.043904	36	305	7937	3.614299	1.000000	0.920276	47.680237
INTERPRO	IPR008271:Serine/threonine protein kinase, active site	4	2.185792	0.047207	54	156	10196	4.841406	0.998907	0.998907	43.520020
GOTERM_CC_FAT	GO:0005769 early endosome	2	1.092896	0.047493	30	8	4786	39.883333	0.996630	0.556599	42.650050
SP_PIR_KEYWORDS	mitochondrion	4	2.185792	0.047588	58	185	12980	4.838770	0.989267	0.364563	41.360955
GOTERM_BP_FAT	GO:0044087 regulation of cellular component biogenesis	3	1.639344	0.048181	36	80	7937	8.267708	1.000000	0.918208	50.957644
GOTERM_BP_FAT	GO:0000910 cytokinesis	3	1.639344	0.048181	36	80	7937	8.267708	1.000000	0.918208	50.957644
GOTERM_BP_FAT	GO:0007049 cell cycle	7	3.825137	0.050177	36	616	7937	2.505366	1.000000	0.906773	52.420573
GOTERM_CC_FAT	GO:0044451 nucleoplasm part	5	2.732240	0.054683	30	240	4786	3.323611	0.998611	0.560637	47.406336
GOTERM_BP_FAT	GO:0007409 axonogenesis	4	2.185792	0.055824	36	198	7937	4.453984	1.000000	0.911694	56.343217
INTERPRO	IPR017442:Serine/threonine protein kinase-related	4	2.185792	0.056564	54	168	10196	4.495591	0.999728	0.983509	49.735453
GOTERM_CC_FAT	GO:0044427 chromosomal part	5	2.732240	0.056774	30	243	4786	3.282579	0.998928	0.532259	48.720659
UP_SEQ_FEATURE	transit peptide:Mitochondrion	4	2.185792	0.061782	25	114	2975	4.175439	0.998069	0.998069	50.596494
GOTERM_BP_FAT	GO:0048666 neuron development	5	2.732240	0.064784	36	347	7937	3.176833	1.000000	0.926623	61.954963
GOTERM_CC_FAT	GO:0044454 nuclear chromosome part	3	1.639344	0.064794	30	69	4786	6.936232	0.999605	0.543319	53.488064
SP_PIR_KEYWORDS	kinase	4	2.185792	0.066136	58	212	12980	4.222511	0.998277	0.439258	52.719508
GOTERM_BP_FAT	GO:0030833 regulation of actin filament polymerization	2	1.092896	0.068332	36	16	7937	27.559028	1.000000	0.922942	63.985012
GOTERM_MF_FAT	GO:0004672 protein kinase activity	5	2.732240	0.071027	45	283	7918	3.108755	0.999996	0.712094	59.259076
GOTERM_CC_FAT	GO:0005654 nucleoplasm	5	2.732240	0.073440	30	265	4786	3.010063	0.999867	0.555720	58.171155
GOTERM_BP_FAT	GO:0032271 regulation of protein polymerization	2	1.092896	0.076548	36	18	7937	24.496914	1.000000	0.932234	68.305744
KEGG_PATHWAY	dme04310:Wnt signaling pathway	3	1.639344	0.077290	14	74	2054	5.947876	0.799872	0.799872	45.785525
GOTERM_CC_FAT	GO:0000228 nuclear chromosome	3	1.639344	0.078469	30	77	4786	6.215584	0.999930	0.549214	60.693490
GOTERM_BP_FAT	GO:0016568 chromatin modification	3	1.639344	0.086913	36	112	7937	5.905506	1.000000	0.943930	73.069267

GOTERM_BP_FAT	GO:0030832 regulation of actin filament length	2	1.092896	0.088739	36	21	7937	20.997354	1.000000	0.937425	73.836240
GOTERM_BP_FAT	GO:0008064 regulation of actin polymerization or depolymerization	2	1.092896	0.088739	36	21	7937	20.997354	1.000000	0.937425	73.836240
GOTERM_MF_FAT	GO:0016779 nucleotidyltransferase activity	3	1.639344	0.088934	45	90	7918	5.865185	1.000000	0.760924	67.863049
GOTERM_BP_FAT	GO:0048742 regulation of skeletal muscle fiber development	2	1.092896	0.092769	36	22	7937	20.042929	1.000000	0.935574	75.456698
GOTERM_BP_FAT	GO:0048641 regulation of skeletal muscle tissue development	2	1.092896	0.092769	36	22	7937	20.042929	1.000000	0.935574	75.456698
GOTERM_BP_FAT	GO:0008582 regulation of synaptic growth at neuromuscular junction	2	1.092896	0.092769	36	22	7937	20.042929	1.000000	0.935574	75.456698
GOTERM_BP_FAT	GO:0051153 regulation of striated muscle cell differentiation	2	1.092896	0.092769	36	22	7937	20.042929	1.000000	0.935574	75.456698
GOTERM_BP_FAT	GO:0051147 regulation of muscle cell differentiation	2	1.092896	0.092769	36	22	7937	20.042929	1.000000	0.935574	75.456698
GOTERM_BP_FAT	GO:0001745 compound eye morphogenesis	4	2.185792	0.096721	36	250	7937	3.527556	1.000000	0.933756	76.955061
GOTERM_BP_FAT	GO:0051963 regulation of synaptogenesis	2	1.092896	0.096780	36	23	7937	19.171498	1.000000	0.924253	76.976979

Table 15: Architecture 15

Category	Term	Count	%	PValue	List Total	Pop Hits	Pop Total	Fold Enrichment	Bonferroni	Benjamini	FDR
SP_PIR_KEYWORDS	motor protein	3	1.149425	0.017709	53	51	12980	14.406215	0.597978	0.597978	15.873672
GOTERM_MF_FAT	GO:0042623 ATPase activity, coupled	5	1.915709	0.019226	28	307	7918	4.605631	0.867208	0.867208	19.512151
GOTERM_BP_FAT	GO:0007297 ovarian follicle cell migration	3	1.149425	0.024716	26	78	7937	11.741124	0.999491	0.999491	28.461351
KEGG_PATHWAY	dme03040:Spliceosome	4	1.532567	0.029533	14	111	2054	5.287001	0.467158	0.467158	20.663178
GOTERM_MF_FAT	GO:0016887 ATPase activity	5	1.915709	0.030279	28	353	7918	4.005463	0.959142	0.797865	29.091906
GOTERM_MF_FAT	GO:0032553 ribonucleotide binding	8	3.065134	0.031307	28	923	7918	2.451014	0.963411	0.668017	29.928337
GOTERM_MF_FAT	GO:0032555 purine ribonucleotide binding	8	3.065134	0.031307	28	923	7918	2.451014	0.963411	0.668017	29.928337
GOTERM_CC_FAT	GO:0005856 cytoskeleton	5	1.915709	0.031524	18	351	4786	3.787591	0.893777	0.893777	28.215370
GOTERM_CC_FAT	GO:0000275 mitochondrial proton-transporting ATP synthase complex, catalytic core F(1)	2	0.766284	0.031544	18	9	4786	59.086420	0.893928	0.674313	28.230514
GOTERM_CC_FAT	GO:0045261 proton-transporting ATP synthase complex, catalytic core F(1)	2	0.766284	0.034990	18	10	4786	53.177778	0.917354	0.564415	30.830223
GOTERM_MF_FAT	GO:0003774 motor activity	3	1.149425	0.035810	28	88	7918	9.640422	0.977461	0.612535	33.484928
GOTERM_MF_FAT	GO:0005524 ATP binding	7	2.681992	0.036821	28	748	7918	2.646390	0.979792	0.541750	34.261153
GOTERM_MF_FAT	GO:0032559 adenyl ribonucleotide binding	7	2.681992	0.037242	28	750	7918	2.639333	0.980689	0.482035	34.581182
INTERPRO	IPR001509:NAD-dependent epimerase/dehydratase	2	0.766284	0.037336	44	9	10196	51.494949	0.982948	0.982948	34.800572
GOTERM_MF_FAT	GO:0017076 purine nucleotide binding	8	3.065134	0.043521	28	989	7918	2.287448	0.990222	0.483709	39.196523
GOTERM_MF_FAT	GO:0030554 adenyl nucleotide binding	7	2.681992	0.051878	28	812	7918	2.437808	0.996075	0.499694	44.879471
GOTERM_MF_FAT	GO:0001883 purine nucleoside binding	7	2.681992	0.053197	28	817	7918	2.422889	0.996604	0.468300	45.730909
GOTERM_MF_FAT	GO:0001882 nucleoside binding	7	2.681992	0.055079	28	824	7918	2.402306	0.997239	0.445231	46.925109
GOTERM_MF_FAT	GO:0046933 hydrogen ion transporting ATP synthase activity, rotational mechanism	2	0.766284	0.059694	28	18	7918	31.420635	0.998340	0.441181	49.752521
SP_PIR_KEYWORDS	myosin	2	0.766284	0.069751	53	18	12980	27.211740	0.974965	0.841774	50.314593
GOTERM_BP_FAT	GO:0046907 intracellular transport	4	1.532567	0.073670	26	313	7937	3.901204	1.000000	0.999991	64.089883
SP_PIR_KEYWORDS	nucleotide-binding	7	2.681992	0.075224	53	743	12980	2.307321	0.981471	0.735382	53.071409
GOTERM_MF_FAT	GO:0046961 proton-transporting ATPase activity, rotational mechanism	2	0.766284	0.075657	28	23	7918	24.590062	0.999720	0.494308	58.507519
GOTERM_MF_FAT	GO:0017022 myosin binding	2	0.766284	0.075657	28	23	7918	24.590062	0.999720	0.494308	58.507519
GOTERM_CC_FAT	GO:0005753 mitochondrial proton-transporting ATP synthase complex	2	0.766284	0.078757	18	23	4786	23.120773	0.996792	0.762014	57.213675
SP_PIR_KEYWORDS	atp-binding	6	2.298851	0.083880	53	585	12980	2.511853	0.988530	0.672741	57.152326
GOTERM_CC_FAT	GO:0016459 myosin complex	2	0.766284	0.088588	18	26	4786	20.452991	0.998486	0.727097	61.710217
GOTERM_MF_FAT	GO:0019829 cation-transporting ATPase activity	2	0.766284	0.094469	28	29	7918	19.502463	0.999967	0.547907	67.029472
GOTERM_CC_FAT	GO:0045259 proton-transporting ATP synthase complex	2	0.766284	0.095087	18	28	4786	18.992063	0.999083	0.688293	64.443588
GOTERM_CC_FAT	GO:0033178 proton-transporting two-sector ATPase complex, catalytic domain	2	0.766284	0.098320	18	29	4786	18.337165	0.999286	0.644759	65.736607

Table 16: Architecture 16

Category	Term	Count	%	PValue	List Total	Pop Hits	Pop Total	Fold Enrichment	Bonferroni	Benjamini	FDR
GOTERM_CC_FAT	GO:0031090 organelle membrane	9	3.125000	0.002768	29	438	4786	3.391120	0.207691	0.207691	2.931043
GOTERM_MF_FAT	GO:0030554 adenyl nucleotide binding	13	4.513889	0.003212	50	812	7918	2.535320	0.392653	0.392653	3.789884
GOTERM_MF_FAT	GO:0001883 purine nucleoside binding	13	4.513889	0.003380	50	817	7918	2.519804	0.408327	0.230797	3.984586
GOTERM_MF_FAT	GO:0001882 nucleoside binding	13	4.513889	0.003628	50	824	7918	2.498398	0.430666	0.171189	4.270482
GOTERM_MF_FAT	GO:0032559 adenyl ribonucleotide binding	12	4.166667	0.005211	50	750	7918	2.533760	0.555043	0.183268	6.081377
GOTERM_MF_FAT	GO:0017076 purine nucleotide binding	14	4.861111	0.005823	50	989	7918	2.241699	0.595521	0.165591	6.772860
GOTERM_CC_FAT	GO:0044429 mitochondrial part	8	2.777778	0.007906	29	411	4786	3.212350	0.486638	0.283507	8.167407
GOTERM_MF_FAT	GO:0032553 ribonucleotide binding	13	4.513889	0.009012	50	923	7918	2.230423	0.754183	0.208529	10.301605
GOTERM_MF_FAT	GO:0032555 purine ribonucleotide binding	13	4.513889	0.009012	50	923	7918	2.230423	0.754183	0.208529	10.301605
GOTERM_CC_FAT	GO:0031966 mitochondrial membrane	6	2.083333	0.011782	29	241	4786	4.108742	0.630481	0.282406	11.945625
GOTERM_MF_FAT	GO:0000166 nucleotide binding	15	5.208333	0.012230	50	1206	7918	1.969652	0.851516	0.238502	13.737525
GOTERM_CC_FAT	GO:0005739 mitochondrion	9	3.125000	0.013805	29	571	4786	2.601244	0.688922	0.253177	13.861580
GOTERM_MF_FAT	GO:0005524 ATP binding	11	3.819444	0.014663	50	748	7918	2.328824	0.898688	0.248883	16.255017
GOTERM_CC_FAT	GO:0005740 mitochondrial envelope	6	2.083333	0.015504	29	258	4786	3.838011	0.730867	0.230881	15.441170
GOTERM_BP_FAT	GO:0007242 intracellular signaling cascade	6	2.083333	0.023355	45	297	7937	3.563187	0.999993	0.999993	28.858440
SP_PIR_KEYWORDS	cell cycle	4	1.388889	0.025803	87	96	12980	6.216475	0.907351	0.907351	24.798597
GOTERM_BP_FAT	GO:0051181 cofactor transport	2	0.694444	0.027419	45	5	7937	70.551111	0.999999	0.999068	33.007610
INTERPRO	IPR012934:Zinc finger, AD-type	4	1.388889	0.027637	72	94	10196	6.026005	0.986267	0.986267	28.524779
GOTERM_BP_FAT	GO:0006839 mitochondrial transport	3	1.041667	0.034619	45	53	7937	9.983648	1.000000	0.997248	39.809067
GOTERM_MF_FAT	GO:0008889 glycerophosphodiester phosphodiesterase activity	2	0.694444	0.036572	50	6	7918	52.786667	0.996896	0.473583	36.073884
INTERPRO	IPR004129:Glycerophosphoryl diester phosphodiesterase	2	0.694444	0.041070	72	6	10196	47.203704	0.998366	0.959572	39.499544
SP_PIR_KEYWORDS	mitosis	3	1.041667	0.044872	87	51	12980	8.776200	0.984669	0.876180	39.377969
GOTERM_MF_FAT	GO:0008270 zinc ion binding	12	4.166667	0.045592	50	1026	7918	1.852164	0.999278	0.514851	42.902602
GOTERM_CC_FAT	GO:0031967 organelle envelope	6	2.083333	0.046516	29	344	4786	2.878508	0.981706	0.486684	40.027628
GOTERM_CC_FAT	GO:0031975 envelope	6	2.083333	0.047009	29	345	4786	2.870165	0.982483	0.438867	40.359351
GOTERM_MF_FAT	GO:0004559 alpha-mannosidase activity	2	0.694444	0.054364	50	9	7918	35.191111	0.999827	0.545084	48.895159
SP_PIR_KEYWORDS	nucleotide-binding	10	3.472222	0.056942	87	743	12980	2.008013	0.995180	0.831084	47.225769
SMART	SM00355:ZnF_C2H2	6	2.083333	0.059793	33	324	4824	2.707071	0.877088	0.877088	41.853400
SP_PIR_KEYWORDS	cell division	3	1.041667	0.063542	87	62	12980	7.219132	0.997457	0.775427	51.115982
GOTERM_BP_FAT	GO:0016192 vesicle-mediated transport	6	2.083333	0.063610	45	391	7937	2.706564	1.000000	0.999738	61.208847
INTERPRO	IPR007087:Zinc finger, C2H2-type	6	2.083333	0.071773	72	320	10196	2.655208	0.999989	0.977594	59.034969
INTERPRO	IPR015880:Zinc finger, C2H2-like	6	2.083333	0.074823	72	324	10196	2.622428	0.999993	0.948939	60.619216
INTERPRO	IPR017946:PLC-like phosphodiesterase, TIM beta/alpha-barrel domain	2	0.694444	0.080477	72	12	10196	23.601852	0.999997	0.923260	63.407817
GOTERM_MF_FAT	GO:0043021 ribonucleoprotein binding	2	0.694444	0.083304	50	14	7918	22.622857	0.999999	0.674853	64.815394
GOTERM_BP_FAT	GO:0006911 phagocytosis, engulfment	4	1.388889	0.084438	45	187	7937	3.772787	1.000000	0.999858	71.946977
GOTERM_MF_FAT	GO:0004693 cyclin-dependent protein kinase activity	2	0.694444	0.088987	50	15	7918	21.114667	0.999999	0.670837	67.347347
GOTERM_MF_FAT	GO:0015923 mannosidase activity	2	0.694444	0.088987	50	15	7918	21.114667	0.999999	0.670837	67.347347
SP_PIR_KEYWORDS	atp-binding	8	2.777778	0.092654	87	585	12980	2.040279	0.999856	0.829601	65.354715
GOTERM_BP_FAT	GO:0006909 phagocytosis	4	1.388889	0.095173	45	197	7937	3.581275	1.000000	0.999768	76.331119
GOTERM_BP_FAT	GO:0019400 alditol metabolic process	2	0.694444	0.095317	45	18	7937	19.597531	1.000000	0.999241	76.385620
GOTERM_BP_FAT	GO:0006071 glycerol metabolic process	2	0.694444	0.095317	45	18	7937	19.597531	1.000000	0.999241	76.385620
GOTERM_MF_FAT	GO:0004672 protein kinase activity	5	1.736111	0.096833	50	283	7918	2.797880	1.000000	0.676189	70.569033
UP_SEQ_FEATURE	nucleotide phosphate-binding region:ATP	5	1.736111	0.097682	28	196	2975	2.710459	0.999981	0.999981	68.443477
GOTERM_BP_FAT	GO:0016044 membrane organization	5	1.736111	0.099115	45	319	7937	2.764542	1.000000	0.998569	77.774410

Table 17: Architecture 17

Category	Term	Count	%	PValue	List Total	Pop Hits	Pop Total	Fold Enrichment	Bonferroni	Benjamini	FDR
GOTERM_BP_FAT	GO:0015031 protein transport	11	0.965759	2.616018e-04	76	278	7937	4.132289	0.186730	0.186730	0.400146
GOTERM_BP_FAT	GO:0008104 protein localization	13	1.141352	2.751145e-04	76	392	7937	3.463379	0.195368	0.102987	0.420774
GOTERM_BP_FAT	GO:0045184 establishment of protein localization	11	0.965759	3.194580e-04	76	285	7937	4.030794	0.223076	0.080695	0.488440
GOTERM_BP_FAT	GO:0016192 vesicle-mediated transport	12	1.053556	0.001024	76	391	7937	3.205142	0.554764	0.183140	1.557364
GOTERM_BP_FAT	GO:0006897 endocytosis	9	0.790167	0.002869	76	258	7937	3.643054	0.896642	0.364862	4.307060
GOTERM_BP_FAT	GO:0010324 membrane invagination	9	0.790167	0.002869	76	258	7937	3.643054	0.896642	0.364862	4.307060
GOTERM_BP_FAT	GO:0016044 membrane organization	10	0.877963	0.002966	76	319	7937	3.273800	0.904331	0.323717	4.450444
GOTERM_BP_FAT	GO:0048858 cell projection morphogenesis	10	0.877963	0.003029	76	320	7937	3.263569	0.908979	0.289922	4.542704
GOTERM_BP_FAT	GO:0032989 cellular component morphogenesis	13	1.141352	0.003136	76	518	7937	2.620936	0.916393	0.266702	4.699905
GOTERM_BP_FAT	GO:0032990 cell part morphogenesis	10	0.877963	0.003793	76	331	7937	3.155112	0.950311	0.283625	5.656994
GOTERM_BP_FAT	GO:0016358 dendrite development	6	0.526778	0.004853	76	117	7937	5.355601	0.978568	0.319063	7.183415
GOTERM_BP_FAT	GO:0048813 dendrite morphogenesis	6	0.526778	0.004853	76	117	7937	5.355601	0.978568	0.319063	7.183415
GOTERM_BP_FAT	GO:0030182 neuron differentiation	11	0.965759	0.004860	76	409	7937	2.808744	0.978686	0.295208	7.193353
GOTERM_CC_FAT	GO:0044451 nucleoplasm part	9	0.790167	0.004991	55	240	4786	3.263182	0.603722	0.603722	6.003431
GOTERM_BP_FAT	GO:0048812 neuron projection morphogenesis	9	0.790167	0.005355	76	286	7937	3.286391	0.985623	0.297780	7.899490
GOTERM_BP_FAT	GO:0031175 neuron projection development	9	0.790167	0.005468	76	287	7937	3.274940	0.986849	0.283355	8.058644
GOTERM_BP_FAT	GO:0048667 cell morphogenesis involved in neuron differentiation	9	0.790167	0.005582	76	288	7937	3.263569	0.987988	0.270829	8.220085
GOTERM_BP_FAT	GO:0030030 cell projection organization	10	0.877963	0.007145	76	365	7937	2.861211	0.996534	0.314525	10.406265
GOTERM_CC_FAT	GO:0043233 organelle lumen	14	1.229148	0.007443	55	556	4786	2.191105	0.748930	0.498931	8.829363
GOTERM_CC_FAT	GO:0070013 intracellular organelle lumen	14	1.229148	0.007443	55	556	4786	2.191105	0.748930	0.498931	8.829363
GOTERM_BP_FAT	GO:0000904 cell morphogenesis involved in differentiation	9	0.790167	0.007521	76	303	7937	3.102006	0.997430	0.311163	10.925041
GOTERM_BP_FAT	GO:0000902 cell morphogenesis	11	0.965759	0.008291	76	442	7937	2.599041	0.998608	0.320833	11.978120
GOTERM_CC_FAT	GO:0005654 nucleoplasm	9	0.790167	0.008971	55	265	4786	2.955334	0.811226	0.426349	10.551914
GOTERM_CC_FAT	GO:0031974 membrane-enclosed lumen	14	1.229148	0.009276	55	571	4786	2.133546	0.821668	0.350159	10.891721
GOTERM_CC_FAT	GO:0031981 nuclear lumen	11	0.965759	0.010111	55	387	4786	2.473385	0.847411	0.313398	11.816042
GOTERM_BP_FAT	GO:0007517 muscle organ development	6	0.526778	0.010218	76	140	7937	4.475752	0.999701	0.362872	14.563815
GOTERM_BP_FAT	GO:0065004 protein-DNA complex assembly	4	0.351185	0.010337	76	48	7937	8.702851	0.999728	0.350803	14.719855
GOTERM_MF_FAT	GO:0008134 transcription factor binding	5	0.438982	0.012331	81	89	7918	5.491746	0.928840	0.928840	14.539776
GOTERM_BP_FAT	GO:0048666 neuron development	9	0.790167	0.016178	76	347	7937	2.708668	0.999997	0.474956	22.116512
GOTERM_BP_FAT	GO:0034622 cellular macromolecular complex assembly	6	0.526778	0.019608	76	165	7937	3.797608	1.000000	0.525248	26.174877
SP_PIR_KEYWORDS	phosphoprotein	15	1.316945	0.020674	123	815	12980	1.942242	0.921817	0.921817	21.379055
INTERPRO	IPR018783:Transcription factor, enhancer of yellow 2	2	0.175593	0.020685	107	2	10196	95.289720	0.995257	0.995257	23.856793
INTERPRO	IPR001026:Epsin, N-terminal	2	0.175593	0.020685	107	2	10196	95.289720	0.995257	0.995257	23.856793
GOTERM_BP_FAT	GO:0008593 regulation of Notch signaling pathway	3	0.263389	0.022970	76	25	7937	12.532105	1.000000	0.565895	29.961200
GOTERM_BP_FAT	GO:0006886 intracellular protein transport	6	0.526778	0.024042	76	174	7937	3.601180	1.000000	0.566500	31.128629
GOTERM_MF_FAT	GO:0003712 transcription cofactor activity	4	0.351185	0.025558	81	63	7918	6.206545	0.995973	0.936540	27.953287
GOTERM_BP_FAT	GO:0034613 cellular protein localization	6	0.526778	0.026764	76	179	7937	3.500588	1.000000	0.590571	34.014837
GOTERM_BP_FAT	GO:0046907 intracellular transport	8	0.702371	0.027847	76	313	7937	2.669245	1.000000	0.590350	35.131113
GOTERM_BP_FAT	GO:0042796 snRNA transcription from RNA polymerase III promoter	2	0.175593	0.028085	76	3	7937	69.622807	1.000000	0.579182	35.373667
GOTERM_BP_FAT	GO:0009301 snRNA transcription	2	0.175593	0.028085	76	3	7937	69.622807	1.000000	0.579182	35.373667
GOTERM_BP_FAT	GO:0065003 macromolecular complex assembly	7	0.614574	0.028335	76	246	7937	2.971705	1.000000	0.568735	35.628166
SP_PIR_KEYWORDS	nucleus	15	1.316945	0.033377	123	869	12980	1.821550	0.984102	0.873912	32.352284
GOTERM_BP_FAT	GO:0045449 regulation of transcription	14	1.229148	0.035223	76	799	7937	1.829886	1.000000	0.636402	42.276787
GOTERM_CC_FAT	GO:0000123 histone acetyltransferase complex	3	0.263389	0.036635	55	27	4786	9.668687	0.998997	0.683610	36.986623
INTERPRO	IPR013809:Epsin-like, N-terminal	2	0.175593	0.040947	107	4	10196	47.644860	0.999978	0.995259	42.025237
GOTERM_BP_FAT	GO:0034621 cellular macromolecular complex subunit organization	6	0.526778	0.042544	76	203	7937	3.086725	1.000000	0.694049	48.636723
SMART	SM00273:ENTH	2	0.175593	0.045646	57	4	4824	42.315789	0.952014	0.952014	37.884619
GOTERM_BP_FAT	GO:0043933 macromolecular complex subunit organization	7	0.614574	0.049107	76	281	7937	2.601564	1.000000	0.734461	53.775495
GOTERM_BP_FAT	GO:0032880 regulation of protein localization	3	0.263389	0.049771	76	38	7937	8.244806	1.000000	0.727740	54.267112
SP_PIR_KEYWORDS	coiled coil	6	0.526778	0.052173	123	215	12980	2.944980	0.998551	0.886852	46.040749
GOTERM_BP_FAT	GO:0022613 ribonucleoprotein complex biogenesis	4	0.351185	0.058997	76	94	7937	4.444009	1.000000	0.777140	60.618358

GOTERM_MF_FAT	GO:0010843 promoter binding	2	0.175593	0.059129	81	6	7918	32.584362	0.999998	0.986798	53.782228
GOTERM_CC_FAT	GO:0005667 transcription factor complex	4	0.351185	0.060720	55	80	4786	4.350909	0.999991	0.809011	53.935260
GOTERM_CC_FAT	GO:0044431 Golgi apparatus part	4	0.351185	0.064442	55	82	4786	4.244789	0.999996	0.785705	56.143379
SP_PIR_KEYWORDS	alternative splicing	11	0.965759	0.064510	123	616	12980	1.884437	0.999707	0.869174	53.596090
GOTERM_CC_FAT	GO:0016585 chromatin remodeling complex	3	0.263389	0.064745	55	37	4786	7.055528	0.999996	0.747391	56.319025
GOTERM_BP_FAT	GO:0006350 transcription	9	0.790167	0.066930	76	459	7937	2.047730	1.000000	0.809558	65.410516
GOTERM_MF_FAT	GO:0016563 transcription activator activity	4	0.351185	0.067519	81	93	7918	4.204434	1.000000	0.975827	58.738103
GOTERM_MF_FAT	GO:0008318 protein prenyltransferase activity	2	0.175593	0.068642	81	7	7918	27.929453	1.000000	0.951653	59.363246
SP_PIR_KEYWORDS	protein transport	4	0.351185	0.069629	123	101	12980	4.179345	0.999850	0.828127	56.437135
KEGG_PATHWAY	dme04512:ECM-receptor interaction	2	0.175593	0.072712	23	7	2054	25.515528	0.795116	0.795116	44.171923
SP_PIR_KEYWORDS	zinc-finger	7	0.614574	0.075695	123	314	12980	2.352545	0.999932	0.798207	59.597689
GOTERM_BP_FAT	GO:0006325 chromatin organization	5	0.438982	0.076675	76	170	7937	3.071594	1.000000	0.843322	70.550800
GOTERM_BP_FAT	GO:0070727 cellular macromolecule localization	6	0.526778	0.080345	76	244	7937	2.568054	1.000000	0.849005	72.294576
SP_PIR_KEYWORDS	alternative initiation	2	0.175593	0.081504	123	9	12980	23.450768	0.999969	0.772759	62.426443
GOTERM_BP_FAT	GO:0007264 small GTPase mediated signal transduction	4	0.351185	0.083739	76	109	7937	3.832448	1.000000	0.853265	73.820701
UP_SEQ_FEATURE	compositionally biased region:Ser-rich	5	0.438982	0.086935	42	123	2975	2.879404	0.999998	0.999998	66.074155
GOTERM_BP_FAT	GO:0016568 chromatin modification	4	0.351185	0.089149	76	112	7937	3.729793	1.000000	0.863809	76.091897
INTERPRO	IPR008942:ENTH/VHS	2	0.175593	0.089802	107	9	10196	21.175493	1.000000	0.999674	70.680515
SP_PIR_KEYWORDS	transcription initiation	2	0.175593	0.090143	123	10	12980	21.105691	0.999990	0.763224	66.300092
SP_PIR_KEYWORDS	zinc	10	0.877963	0.093434	123	577	12980	1.828916	0.999994	0.735440	67.677169
GOTERM_CC_FAT	GO:0005783 endoplasmic reticulum	7	0.614574	0.098378	55	284	4786	2.144814	1.000000	0.852783	72.235894
GOTERM_BP_FAT	GO:0006383 transcription from RNA polymerase III promoter	2	0.175593	0.099229	76	11	7937	18.988038	1.000000	0.886117	79.840370

Table 18: Architecture 18

Category	Term	Count	%	PValue	List Total	Pop Hits	Pop Total	Fold Enrichment	Bonferroni	Benjamini	FDR
GOTERM_BP_FAT	GO:0008103 oocyte microtubule cytoskeleton polarization	3	0.837989	0.004411	48	17	7937	29.180147	0.920582	0.920582	6.281840
GOTERM_BP_FAT	GO:0051276 chromosome organization	7	1.955307	0.006015	48	282	7937	4.104536	0.968473	0.822442	8.473495
SMART	SM00384:AT_hook	3	0.837989	0.011917	31	27	4824	17.290323	0.342686	0.342686	10.074490
GOTERM_BP_FAT	GO:0030951 establishment or maintenance of microtubule cytoskeleton polarity	3	0.837989	0.012587	48	29	7937	17.105603	0.999296	0.911027	16.964707
GOTERM_BP_FAT	GO:0016325 oocyte microtubule cytoskeleton organization	3	0.837989	0.012587	48	29	7937	17.105603	0.999296	0.911027	16.964707
GOTERM_BP_FAT	GO:0030952 establishment or maintenance of cytoskeleton polarity	3	0.837989	0.012587	48	29	7937	17.105603	0.999296	0.911027	16.964707
INTERPRO	IPR017956:AT hook, DNA-binding, conserved site	3	0.837989	0.017489	78	27	10196	14.524217	0.927842	0.927842	18.978069
SP_PIR_KEYWORDS	transferase	13	3.631285	0.018526	92	873	12980	2.100951	0.784201	0.784201	18.108563
GOTERM_BP_FAT	GO:0007010 cytoskeleton organization	8	2.234637	0.018578	48	465	7937	2.844803	0.999978	0.931872	24.059673
INTERPRO	IPR007829:TM2	2	0.558659	0.022487	78	3	10196	87.145299	0.966254	0.816298	23.760368
INTERPRO	IPR014014:RNA helicase, DEAD-box type, Q motif	3	0.837989	0.024122	78	32	10196	12.254808	0.973703	0.702626	25.267470
GOTERM_MF_FAT	GO:0008186 RNA-dependent ATPase activity	3	0.837989	0.027149	52	40	7918	11.420192	0.994495	0.994495	28.950721
GOTERM_MF_FAT	GO:0004004 ATP-dependent RNA helicase activity	3	0.837989	0.027149	52	40	7918	11.420192	0.994495	0.994495	28.950721
GOTERM_CC_FAT	GO:0043232 intracellular non-membrane-bounded organelle	12	3.351955	0.029132	32	943	4786	1.903234	0.975167	0.975167	28.957195
GOTERM_CC_FAT	GO:0043228 non-membrane-bounded organelle	12	3.351955	0.029132	32	943	4786	1.903234	0.975167	0.975167	28.957195
GOTERM_BP_FAT	GO:0000226 microtubule cytoskeleton organization	6	1.675978	0.030531	48	298	7937	3.329279	1.000000	0.971371	36.558597
GOTERM_BP_FAT	GO:0007017 microtubule-based process	7	1.955307	0.031419	48	406	7937	2.850934	1.000000	0.952579	37.406862
GOTERM_BP_FAT	GO:0002168 instar larval development	3	0.837989	0.032576	48	48	7937	10.334635	1.000000	0.933528	38.494851
GOTERM_BP_FAT	GO:0070085 glycosylation	3	0.837989	0.033836	48	49	7937	10.123724	1.000000	0.915030	39.660201
GOTERM_BP_FAT	GO:0043413 biopolymer glycosylation	3	0.837989	0.033836	48	49	7937	10.123724	1.000000	0.915030	39.660201
GOTERM_BP_FAT	GO:0006486 protein amino acid glycosylation	3	0.837989	0.033836	48	49	7937	10.123724	1.000000	0.915030	39.660201
GOTERM_MF_FAT	GO:0003724 RNA helicase activity	3	0.837989	0.039465	52	49	7918	9.322606	0.999505	0.977740	39.346581
INTERPRO	IPR011545:DNA/RNA helicase, DEAD/DEAH box type, N-terminal	3	0.837989	0.041648	78	43	10196	9.119857	0.998233	0.794975	39.794890
GOTERM_MF_FAT	GO:0008417 fucosyltransferase activity	2	0.558659	0.044242	52	7	7918	43.505495	0.999807	0.942200	42.987934
GOTERM_BP_FAT	GO:0009994 oocyte differentiation	4	1.117318	0.047872	48	138	7937	4.792874	1.000000	0.955984	51.322200
UP_SEQ_FEATURE	short sequence motif:SH3-binding	2	0.558659	0.049446	31	5	2975	38.387097	0.985138	0.985138	41.902081
GOTERM_BP_FAT	GO:0009101 glycoprotein biosynthetic process	3	0.837989	0.053349	48	63	7937	7.874008	1.000000	0.956782	55.274436
GOTERM_BP_FAT	GO:0007293 germarium-derived egg chamber formation	3	0.837989	0.053349	48	63	7937	7.874008	1.000000	0.956782	55.274436
SMART	SM00166:UBX	2	0.558659	0.054642	31	9	4824	34.580645	0.860083	0.625946	39.208760
GOTERM_BP_FAT	GO:0009100 glycoprotein metabolic process	3	0.837989	0.057948	48	66	7937	7.516098	1.000000	0.955380	58.359522
SP_PIR_KEYWORDS	phosphoprotein	11	3.072626	0.058723	92	815	12980	1.904241	0.993004	0.916359	47.613386
UP_SEQ_FEATURE	domain:N-acetyltransferase	2	0.558659	0.059048	31	6	2975	31.989247	0.993601	0.920006	47.887151
GOTERM_BP_FAT	GO:0002164 larval development	3	0.837989	0.061089	48	68	7937	7.295037	1.000000	0.950703	60.351013
SP_PIR_KEYWORDS	coiled coil	5	1.396648	0.064320	92	215	12980	3.281092	0.995710	0.837515	50.847150
INTERPRO	IPR001012:UBX	2	0.558659	0.065976	78	9	10196	29.048433	0.999962	0.869180	55.696513
GOTERM_CC_FAT	GO:0016272 prefoldin complex	2	0.558659	0.069056	32	11	4786	27.193182	0.999870	0.988579	56.285257
GOTERM_BP_FAT	GO:0051656 establishment of organelle localization	3	0.837989	0.072523	48	75	7937	6.614167	1.000000	0.963790	66.876368
GOTERM_BP_FAT	GO:0006730 one-carbon metabolic process	3	0.837989	0.074209	48	76	7937	6.527138	1.000000	0.957399	67.749521
GOTERM_BP_FAT	GO:0051726 regulation of cell cycle	4	1.117318	0.074688	48	166	7937	3.984438	1.000000	0.948452	67.993380
GOTERM_BP_FAT	GO:0070271 protein complex biogenesis	4	1.117318	0.075749	48	167	7937	3.960579	1.000000	0.940454	68.527648
GOTERM_BP_FAT	GO:0006461 protein complex assembly	4	1.117318	0.075749	48	167	7937	3.960579	1.000000	0.940454	68.527648
SMART	SM00487:DEXDc	3	0.837989	0.078456	31	75	4824	6.224516	0.942713	0.614505	51.505227
SMART	SM00490:HELICc	3	0.837989	0.080273	31	76	4824	6.142615	0.946536	0.519144	52.345453
GOTERM_BP_FAT	GO:0044087 regulation of cellular component biogenesis	3	0.837989	0.081081	48	80	7937	6.200781	1.000000	0.942161	71.089907
GOTERM_BP_FAT	GO:0051640 organelle localization	3	0.837989	0.084589	48	82	7937	6.049543	1.000000	0.940006	72.668099
GOTERM_MF_FAT	GO:0008026 ATP-dependent helicase activity	3	0.837989	0.089677	52	78	7918	5.856509	1.000000	0.988197	68.861651
GOTERM_MF_FAT	GO:0070035 purine NTP-dependent helicase activity	3	0.837989	0.089677	52	78	7918	5.856509	1.000000	0.988197	68.861651
GOTERM_BP_FAT	GO:0007062 sister chromatid cohesion	2	0.558659	0.090734	48	16	7937	20.669271	1.000000	0.943219	75.240626

Table 19: Architecture 19

Category	Term	Count	%	PValue	List Total	Pop Hits	Pop Total	Fold Enrichment	Bonferroni	Benjamini	FDR
SP_PIR_KEYWORDS	nucleus	11	3.741497	7.673181e-04	47	869	12980	3.495825	0.042811	0.042811	0.757986
SP_PIR_KEYWORDS	Transcription	7	2.380952	0.002494	47	393	12980	4.919062	0.132645	0.068681	2.444329
GOTERM_BP_FAT	GO:0006357 regulation of transcription from RNA polymerase II promoter	5	1.700680	0.006263	32	192	7937	6.459147	0.921492	0.921492	8.404865
GOTERM_MF_FAT	GO:0008134 transcription factor binding	4	1.360544	0.006387	35	89	7918	10.167576	0.565249	0.565249	7.190307
GOTERM_BP_FAT	GO:0016044 membrane organization	6	2.040816	0.007302	32	319	7937	4.665165	0.948602	0.773288	9.733780
GOTERM_BP_FAT	GO:0006350 transcription	7	2.380952	0.007737	32	459	7937	3.782612	0.956965	0.649565	10.285166
SP_PIR_KEYWORDS	transcription regulation	6	2.040816	0.011540	47	387	12980	4.281709	0.483982	0.197913	10.868164
GOTERM_BP_FAT	GO:0006897 endocytosis	5	1.700680	0.017210	32	258	7937	4.806807	0.999116	0.827565	21.539937
GOTERM_BP_FAT	GO:0010324 membrane invagination	5	1.700680	0.017210	32	258	7937	4.806807	0.999116	0.827565	21.539937
GOTERM_CC_FAT	GO:0070013 intracellular organelle lumen	7	2.380952	0.028174	22	556	4786	2.738882	0.961535	0.961535	27.748593
GOTERM_CC_FAT	GO:0043233 organelle lumen	7	2.380952	0.028174	22	556	4786	2.738882	0.961535	0.961535	27.748593
GOTERM_MF_FAT	GO:0003712 transcription cofactor activity	3	1.020408	0.029687	35	63	7918	10.772789	0.980114	0.858981	29.598292
GOTERM_BP_FAT	GO:0045449 regulation of transcription	8	2.721088	0.031273	32	799	7937	2.483417	0.999997	0.923735	35.850844
GOTERM_CC_FAT	GO:0031974 membrane-enclosed lumen	7	2.380952	0.031684	22	571	4786	2.666932	0.974534	0.840420	30.660840
GOTERM_BP_FAT	GO:0051252 regulation of RNA metabolic process	7	2.380952	0.044319	32	678	7937	2.560795	1.000000	0.953106	46.923129
GOTERM_BP_FAT	GO:0006366 transcription from RNA polymerase II promoter	3	1.020408	0.056673	32	99	7937	7.516098	1.000000	0.965799	55.746228
SP_PIR_KEYWORDS	activator	3	1.020408	0.059736	47	112	12980	7.397416	0.970130	0.584273	45.694297
GOTERM_MF_FAT	GO:0016563 transcription activator activity	3	1.020408	0.060065	35	93	7918	7.297696	0.999682	0.931728	51.391526
SP_PIR_KEYWORDS	metal-binding	7	2.380952	0.060499	47	796	12980	2.428633	0.971481	0.509060	46.129423
GOTERM_BP_FAT	GO:0016192 vesicle-mediated transport	5	1.700680	0.063885	32	391	7937	3.171755	1.000000	0.964638	60.246664
GOTERM_MF_FAT	GO:0030528 transcription regulator activity	7	2.380952	0.067458	35	682	7918	2.321994	0.999886	0.896670	55.662149
GOTERM_BP_FAT	GO:0032583 regulation of gene-specific transcription	2	0.680272	0.068088	32	18	7937	27.559028	1.000000	0.958133	62.669262
GOTERM_BP_FAT	GO:0045941 positive regulation of transcription	3	1.020408	0.074832	32	116	7937	6.414601	1.000000	0.957151	66.271694
GOTERM_BP_FAT	GO:0010628 positive regulation of gene expression	3	1.020408	0.075952	32	117	7937	6.359776	1.000000	0.945433	66.838133
GOTERM_BP_FAT	GO:0051173 positive regulation of nitrogen compound metabolic process	3	1.020408	0.080489	32	121	7937	6.149535	1.000000	0.941110	69.042201
GOTERM_BP_FAT	GO:0045935 positive regulation of nucleobase, nucleoside, nucleotide and nucleic acid metabolic process	3	1.020408	0.080489	32	121	7937	6.149535	1.000000	0.941110	69.042201
GOTERM_CC_FAT	GO:0031981 nuclear lumen	5	1.700680	0.084071	22	387	4786	2.810665	0.999955	0.964457	63.164216
GOTERM_CC_FAT	GO:0044451 nucleoplasm part	4	1.360544	0.085082	22	240	4786	3.625758	0.999960	0.920679	63.624299
GOTERM_BP_FAT	GO:0006355 regulation of transcription, DNA-dependent	6	2.040816	0.086087	32	612	7937	2.431679	1.000000	0.939460	71.574007
GOTERM_BP_FAT	GO:0010608 posttranscriptional regulation of gene expression	3	1.020408	0.086278	32	126	7937	5.905506	1.000000	0.926480	71.657058
GOTERM_BP_FAT	GO:0010557 positive regulation of macromolecule biosynthetic process	3	1.020408	0.089811	32	129	7937	5.768169	1.000000	0.921196	73.150736
GOTERM_BP_FAT	GO:0006351 transcription, DNA-dependent	3	1.020408	0.094590	32	133	7937	5.594690	1.000000	0.919155	75.054628
GOTERM_MF_FAT	GO:0003702 RNA polymerase II transcription factor activity	4	1.360544	0.096868	35	257	7918	3.521067	0.999998	0.929281	69.471744
GOTERM_BP_FAT	GO:0032774 RNA biosynthetic process	3	1.020408	0.098222	32	136	7937	5.471278	1.000000	0.914824	76.417176
SP_PIR_KEYWORDS	zinc-finger	4	1.360544	0.099609	47	314	12980	3.518092	0.997473	0.630940	64.656232

Table 20: Architecture 20

Category	Term	Count	%	PValue	List Total	Pop Hits	Pop Total	Fold Enrichment	Bonferroni	Benjamini	FDR
GOTERM_BP_FAT	GO:0015031 protein transport	16	1.413428	1.591004e-04	146	278	7937	3.128807	0.118120	0.118120	0.243538
SP_PIR_KEYWORDS	protein transport	10	0.883392	1.687039e-04	257	101	12980	5.000578	0.022355	0.022355	0.197345
GOTERM_BP_FAT	GO:0045184 establishment of protein localization	16	1.413428	2.090449e-04	146	285	7937	3.051959	0.152244	0.079263	0.319875
GOTERM_BP_FAT	GO:0046907 intracellular transport	16	1.413428	5.689533e-04	146	313	7937	2.778940	0.362117	0.139178	0.868358
GOTERM_BP_FAT	GO:0008104 protein localization	17	1.501767	0.002053	146	392	7937	2.357580	0.802766	0.333584	3.099985
GOTERM_CC_FAT	GO:0005762 mitochondrial large ribosomal subunit	6	0.530035	0.003005	103	47	4786	5.931832	0.465230	0.465230	3.724816
GOTERM_CC_FAT	GO:0000315 organellar large ribosomal subunit	6	0.530035	0.003005	103	47	4786	5.931832	0.465230	0.465230	3.724816
GOTERM_CC_FAT	GO:0043232 intracellular non-membrane-bounded organelle	33	2.915194	0.003147	103	943	4786	1.626064	0.480913	0.279523	3.898453
GOTERM_CC_FAT	GO:0043228 non-membrane-bounded organelle	33	2.915194	0.003147	103	943	4786	1.626064	0.480913	0.279523	3.898453
SP_PIR_KEYWORDS	mitochondrion	11	0.971731	0.003695	257	185	12980	3.003050	0.391032	0.219636	4.241167
GOTERM_CC_FAT	GO:0031974 membrane-enclosed lumen	23	2.031802	0.003712	103	571	4786	1.871661	0.538665	0.227310	4.583423
COG_ONTOLOGY	RNA processing and modification	4	0.353357	0.004330	25	18	1237	10.995556	0.058939	0.058939	2.904472
GOTERM_CC_FAT	GO:0000313 organellar ribosome	7	0.618375	0.004643	103	74	4786	4.395434	0.620149	0.214939	5.701434
GOTERM_CC_FAT	GO:0005761 mitochondrial ribosome	7	0.618375	0.004643	103	74	4786	4.395434	0.620149	0.214939	5.701434
GOTERM_CC_FAT	GO:0043233 organelle lumen	22	1.943463	0.005854	103	556	4786	1.838584	0.705140	0.216709	7.138835
GOTERM_CC_FAT	GO:0070013 intracellular organelle lumen	22	1.943463	0.005854	103	556	4786	1.838584	0.705140	0.216709	7.138835
SMART	SM00320:WD40	11	0.971731	0.007562	107	187	4824	2.652007	0.498804	0.498804	7.942205
INTERPRO	IPR001680:WD40 repeat	11	0.971731	0.008740	227	187	10196	2.642135	0.974951	0.974951	11.601129
INTERPRO	IPR012677:Nucleotide-binding, alpha-beta plait	9	0.795053	0.009841	227	134	10196	3.016766	0.984296	0.874683	12.970765
GOTERM_BP_FAT	GO:0016192 vesicle-mediated transport	15	1.325088	0.011946	146	391	7937	2.085538	0.999925	0.850259	16.820725
INTERPRO	IPR019782:WD40 repeat 2	9	0.795053	0.014746	227	144	10196	2.807269	0.998049	0.875038	18.834004
INTERPRO	IPR013087:Zinc finger, C2H2-type/integrase, DNA-binding	9	0.795053	0.015319	227	145	10196	2.787908	0.998472	0.802282	19.494791
SP_PIR_KEYWORDS	wd repeat	9	0.795053	0.017175	257	166	12980	2.738268	0.901863	0.538742	18.358531
KEGG_PATHWAY	dme03040:Spliceosome	8	0.706714	0.017880	52	111	2054	2.846847	0.629281	0.629281	16.260386
INTERPRO	IPR017986:WD40 repeat, region	9	0.795053	0.018430	227	150	10196	2.694978	0.999595	0.790394	22.994706
GOTERM_BP_FAT	GO:0016071 mRNA metabolic process	10	0.883392	0.018718	146	219	7937	2.482329	1.000000	0.916920	25.141645
SP_PIR_KEYWORDS	rna-binding	8	0.706714	0.019543	257	138	12980	2.927875	0.928970	0.483749	20.632194
GOTERM_BP_FAT	GO:0000381 regulation of alternative nuclear mRNA splicing, via spliceosome	5	0.441696	0.019982	146	57	7937	4.768685	1.000000	0.897504	26.605141
INTERPRO	IPR019781:WD40 repeat, subgroup	9	0.795053	0.021226	227	154	10196	2.624979	0.999878	0.777271	26.019586
GOTERM_BP_FAT	GO:0044265 cellular macromolecule catabolic process	10	0.883392	0.021889	146	225	7937	2.416134	1.000000	0.887582	28.763474
GOTERM_BP_FAT	GO:0000082 G1/S transition of mitotic cell cycle	3	0.265018	0.022659	146	13	7937	12.545311	1.000000	0.866258	29.618340
GOTERM_CC_FAT	GO:0005794 Golgi apparatus	9	0.795053	0.022891	103	164	4786	2.549964	0.991906	0.551918	25.331589
GOTERM_CC_FAT	GO:0005654 nucleoplasm	12	1.060071	0.024486	103	265	4786	2.104122	0.994239	0.521286	26.854992
GOTERM_BP_FAT	GO:0000377 RNA splicing, via transesterification reactions with bulged adenosine as nucleophile	7	0.618375	0.027615	146	126	7937	3.020167	1.000000	0.890551	34.893709
GOTERM_BP_FAT	GO:0000398 nuclear mRNA splicing, via spliceosome	7	0.618375	0.027615	146	126	7937	3.020167	1.000000	0.890551	34.893709
GOTERM_BP_FAT	GO:0050684 regulation of mRNA processing	5	0.441696	0.027725	146	63	7937	4.314525	1.000000	0.867252	35.006378
GOTERM_BP_FAT	GO:0048024 regulation of nuclear mRNA splicing, via spliceosome	5	0.441696	0.027725	146	63	7937	4.314525	1.000000	0.867252	35.006378
GOTERM_BP_FAT	GO:0000375 RNA splicing, via transesterification reactions	7	0.618375	0.028564	146	127	7937	2.996387	1.000000	0.851598	35.860182
INTERPRO	IPR015943:WD40/YVTN repeat-like	10	0.883392	0.029610	227	195	10196	2.303400	0.999997	0.835270	34.440950
GOTERM_MF_FAT	GO:0000166 nucleotide binding	33	2.915194	0.029744	152	1206	7918	1.425406	0.999906	0.999906	33.297385
GOTERM_BP_FAT	GO:0009057 macromolecule catabolic process	11	0.971731	0.030138	146	277	7937	2.158820	1.000000	0.844267	37.434334
GOTERM_CC_FAT	GO:0044451 nucleoplasm part	11	0.971731	0.030752	103	240	4786	2.129693	0.998492	0.556082	32.565528
GOTERM_BP_FAT	GO:0043484 regulation of RNA splicing	5	0.441696	0.032182	146	66	7937	4.118410	1.000000	0.842110	39.425191
SP_PIR_KEYWORDS	phosphoprotein	25	2.208481	0.032428	257	815	12980	1.549259	0.987934	0.586655	32.020812
GOTERM_CC_FAT	GO:0031981 nuclear lumen	15	1.325088	0.033827	103	387	4786	1.801009	0.999221	0.548557	35.214697
GOTERM_BP_FAT	GO:0019941 modification-dependent protein catabolic process	8	0.706714	0.034967	146	169	7937	2.573397	1.000000	0.846578	42.041879
GOTERM_BP_FAT	GO:0043632 modification-dependent macromolecule catabolic process	8	0.706714	0.035932	146	170	7937	2.558259	1.000000	0.835821	42.923751
GOTERM_BP_FAT	GO:0001737 establishment of imaginal disc-derived wing hair orientation	3	0.265018	0.037677	146	17	7937	9.593473	1.000000	0.832151	44.486297
GOTERM_BP_FAT	GO:0006886 intracellular protein transport	8	0.706714	0.039969	146	174	7937	2.499449	1.000000	0.833072	46.478192
GOTERM_BP_FAT	GO:0018993 somatic sex determination	3	0.265018	0.041888	146	18	7937	9.060502	1.000000	0.831226	48.094922
INTERPRO	IPR007275:YT521-B-like protein	2	0.176678	0.043842	227	2	10196	44.916300	1.000000	0.904981	46.727950

SP_PIR_KEYWORDS	ubl conjugation pathway	6	0.530035	0.044414	257	98	12980	3.092194	0.997729	0.637461	41.252351
GOTERM_BP_FAT	GO:0034613 cellular protein localization	8	0.706714	0.045418	146	179	7937	2.429632	1.000000	0.840550	50.949359
GOTERM_BP_FAT	GO:0044257 cellular protein catabolic process	8	0.706714	0.050107	146	183	7937	2.376525	1.000000	0.855408	54.514601
GOTERM_BP_FAT	GO:0051603 proteolysis involved in cellular protein catabolic process	8	0.706714	0.050107	146	183	7937	2.376525	1.000000	0.855408	54.514601
GOTERM_MF_FAT	GO:0003735 structural constituent of ribosome	8	0.706714	0.053592	152	178	7918	2.341218	1.000000	0.999787	52.223600
GOTERM_BP_FAT	GO:0008380 RNA splicing	7	0.618375	0.053607	146	148	7937	2.571224	1.000000	0.861723	57.016304
GOTERM_CC_FAT	GO:0005739 mitochondrion	19	1.678445	0.055383	103	571	4786	1.546155	0.999993	0.694285	51.262485
INTERPRO	IPR012934:Zinc finger, AD-type	6	0.530035	0.057544	227	94	10196	2.866998	1.000000	0.937072	56.504843
GOTERM_CC_FAT	GO:0030529 ribonucleoprotein complex	14	1.236749	0.057579	103	379	4786	1.716423	0.999996	0.674166	52.672307
GOTERM_BP_FAT	GO:0006397 mRNA processing	8	0.706714	0.064545	146	194	7937	2.241774	1.000000	0.898913	64.030489
GOTERM_MF_FAT	GO:0003729 mRNA binding	8	0.706714	0.064791	152	186	7918	2.240521	1.000000	0.998946	59.273037
INTERPRO	IPR002130:Peptidyl-prolyl cis-trans isomerase, cyclophilin-type	3	0.265018	0.065329	227	19	10196	7.092047	1.000000	0.941432	61.288593
GOTERM_CC_FAT	GO:0015934 large ribosomal subunit	6	0.530035	0.065534	103	102	4786	2.733295	0.999999	0.691137	57.472033
GOTERM_BP_FAT	GO:0016044 membrane organization	11	0.971731	0.066685	146	319	7937	1.874587	1.000000	0.896860	65.271033
UP_SEQ_FEATURE	nucleotide phosphate-binding region:GTP	5	0.441696	0.068694	77	61	2975	3.166915	1.000000	1.000000	59.335701
GOTERM_MF_FAT	GO:0003677 DNA binding	23	2.031802	0.068807	152	830	7918	1.443516	1.000000	0.995795	61.556436
GOTERM_BP_FAT	GO:0016197 endosome transport	3	0.265018	0.070416	146	24	7937	6.795377	1.000000	0.900477	67.338563
GOTERM_BP_FAT	GO:0007021 tubulin complex assembly	2	0.176678	0.071110	146	4	7937	27.181507	1.000000	0.893683	67.710513
INTERPRO	IPR019775:WD40 repeat, conserved site	7	0.618375	0.073106	227	132	10196	2.381925	1.000000	0.944899	65.575125
GOTERM_BP_FAT	GO:0030163 protein catabolic process	8	0.706714	0.073388	146	200	7937	2.174521	1.000000	0.892488	68.902755
GOTERM_BP_FAT	GO:0007530 sex determination	3	0.265018	0.075649	146	25	7937	6.523562	1.000000	0.891330	70.045554
GOTERM_BP_FAT	GO:0051329 interphase of mitotic cell cycle	3	0.265018	0.075649	146	25	7937	6.523562	1.000000	0.891330	70.045554
GOTERM_BP_FAT	GO:0051325 interphase	3	0.265018	0.075649	146	25	7937	6.523562	1.000000	0.891330	70.045554
GOTERM_BP_FAT	GO:0070727 cellular macromolecule localization	9	0.795053	0.078064	146	244	7937	2.005193	1.000000	0.890756	71.222697
GOTERM_BP_FAT	GO:0006413 translational initiation	4	0.353357	0.078829	146	55	7937	3.953674	1.000000	0.884929	71.586170
SP_PIR_KEYWORDS	gtp-binding	7	0.618375	0.080501	257	152	12980	2.325927	0.999987	0.799430	62.567681
GOTERM_BP_FAT	GO:0065003 macromolecular complex assembly	9	0.795053	0.080971	146	246	7937	1.988891	1.000000	0.883725	72.582260
INTERPRO	IPR018503:Tetraspanin, conserved site	2	0.176678	0.085770	227	4	10196	22.458150	1.000000	0.956655	71.624432
INTERPRO	IPR006903:Protein of unknown function DUF618	2	0.176678	0.085770	227	4	10196	22.458150	1.000000	0.956655	71.624432
SP_PIR_KEYWORDS	Rotamase	3	0.265018	0.086409	257	25	12980	6.060700	0.999994	0.779915	65.288912
SMART	SM00360:RRM	7	0.618375	0.088824	107	141	4824	2.238218	0.999789	0.985482	63.726810
KEGG_PATHWAY	dme04120:Ubiquitin mediated proteolysis	6	0.530035	0.089470	52	97	2054	2.443299	0.994230	0.924039	60.223740
GOTERM_MF_FAT	GO:0019787 small conjugating protein ligase activity	5	0.441696	0.089789	152	89	7918	2.926523	1.000000	0.996900	71.680038
GOTERM_MF_FAT	GO:0016251 general RNA polymerase II transcription factor activity	5	0.441696	0.089789	152	89	7918	2.926523	1.000000	0.996900	71.680038
GOTERM_CC_FAT	GO:0005667 transcription factor complex	5	0.441696	0.090168	103	80	4786	2.904126	1.000000	0.779514	69.638695
SP_PIR_KEYWORDS	ribosomal protein	7	0.618375	0.090667	257	157	12980	2.251853	0.999997	0.757100	67.136510
INTERPRO	IPR000504:RNA recognition motif, RNP-1	7	0.618375	0.093346	227	141	10196	2.229887	1.000000	0.957826	74.754616
GOTERM_MF_FAT	GO:0003743 translation initiation factor activity	4	0.353357	0.098377	152	58	7918	3.592559	1.000000	0.995002	75.060812
GOTERM_BP_FAT	GO:0006511 ubiquitin-dependent protein catabolic process	5	0.441696	0.098409	146	96	7937	2.831407	1.000000	0.922502	79.557543
GOTERM_MF_FAT	GO:0003755 peptidyl-prolyl cis-trans isomerase activity	3	0.265018	0.098978	152	28	7918	5.581297	1.000000	0.989653	75.282838

Table 21: Architecture 21

Category	Term	Count	%	PValue	List Total	Pop Hits	Pop Total	Fold Enrichment	Bonferroni	Benjamini	FDR
GOTERM_CC_FAT	GO:0005739 mitochondrion	22	2.131783	3.102864e-04	81	571	4786	2.276535	0.059596	0.059596	0.387579
GOTERM_CC_FAT	GO:0031966 mitochondrial membrane	13	1.259690	5.727274e-04	81	241	4786	3.187234	0.107236	0.055138	0.714315
GOTERM_CC_FAT	GO:0005740 mitochondrial envelope	13	1.259690	0.001051	81	258	4786	2.977223	0.187885	0.067020	1.306638
GOTERM_BP_FAT	GO:0043933 macromolecular complex subunit organization	13	1.259690	0.001066	121	281	7937	3.034646	0.627449	0.627449	1.654352
GOTERM_CC_FAT	GO:0031967 organelle envelope	15	1.453488	0.001451	81	344	4786	2.576443	0.249798	0.069332	1.800010
GOTERM_CC_FAT	GO:0031975 envelope	15	1.453488	0.001492	81	345	4786	2.568975	0.255883	0.057398	1.850547
GOTERM_CC_FAT	GO:0044429 mitochondrial part	16	1.550388	0.002864	81	411	4786	2.300201	0.433238	0.090296	3.524891
GOTERM_BP_FAT	GO:0042775 mitochondrial ATP synthesis coupled electron transport	6	0.581395	0.003325	121	67	7937	5.874183	0.954208	0.786010	5.076467
GOTERM_BP_FAT	GO:0042773 ATP synthesis coupled electron transport	6	0.581395	0.004273	121	71	7937	5.543243	0.981035	0.733326	6.479748
GOTERM_BP_FAT	GO:0033554 cellular response to stress	9	0.872093	0.005084	121	176	7937	3.354292	0.991087	0.692741	7.665202
GOTERM_BP_FAT	GO:0022904 respiratory electron transport chain	6	0.581395	0.005101	121	74	7937	5.318517	0.991221	0.612129	7.688840
SP_PIR_KEYWORDS	mitochondrion	9	0.872093	0.007935	201	185	12980	3.141589	0.647827	0.647827	8.871734
GOTERM_MF_FAT	GO:0008026 ATP-dependent helicase activity	6	0.581395	0.008683	130	78	7918	4.685207	0.920260	0.920260	10.946367
GOTERM_MF_FAT	GO:0070035 purine NTP-dependent helicase activity	6	0.581395	0.008683	130	78	7918	4.685207	0.920260	0.920260	10.946367
GOTERM_CC_FAT	GO:0019866 organelle inner membrane	10	0.968992	0.009647	81	217	4786	2.722876	0.853291	0.239810	11.422958
UP_SEQ_FEATURE	topological domain:Mitochondrial intermembrane	3	0.290698	0.009975	53	9	2975	18.710692	0.819893	0.819893	11.522284
GOTERM_BP_FAT	GO:0051276 chromosome organization	11	1.065891	0.010154	121	282	7937	2.558672	0.999921	0.793031	14.758124
GOTERM_BP_FAT	GO:0022900 electron transport chain	6	0.581395	0.011505	121	90	7937	4.373003	0.999978	0.783642	16.560104
GOTERM_BP_FAT	GO:0034621 cellular macromolecular complex subunit organization	9	0.872093	0.011635	121	203	7937	2.908155	0.999980	0.741963	16.731180
KEGG_PATHWAY	dme00190:Oxidative phosphorylation	8	0.775194	0.013556	41	135	2054	2.968744	0.466258	0.466258	12.103081
GOTERM_BP_FAT	GO:0007242 intracellular signaling cascade	11	1.065891	0.014236	121	297	7937	2.429446	0.999998	0.771269	20.093594
GOTERM_BP_FAT	GO:0006325 chromatin organization	8	0.775194	0.014365	121	170	7937	3.086825	0.999998	0.738113	20.257500
GOTERM_BP_FAT	GO:0006119 oxidative phosphorylation	7	0.678295	0.014733	121	132	7937	3.478525	0.999999	0.713353	20.722448
GOTERM_BP_FAT	GO:0006122 mitochondrial electron transport, ubiquinol to cytochrome c	3	0.290698	0.015858	121	13	7937	15.137317	1.000000	0.708732	22.126397
GOTERM_CC_FAT	GO:0005743 mitochondrial inner membrane	9	0.872093	0.017743	81	200	4786	2.658889	0.971122	0.357947	20.069966
GOTERM_BP_FAT	GO:0006281 DNA repair	6	0.581395	0.018918	121	102	7937	3.858532	1.000000	0.743461	25.829938
GOTERM_MF_FAT	GO:0003924 GTPase activity	7	0.678295	0.019224	130	130	7918	3.279645	0.996409	0.940071	22.744230
GOTERM_BP_FAT	GO:0006997 nucleus organization	4	0.387597	0.020743	121	39	7937	6.727697	1.000000	0.750035	27.959011
SP_PIR_KEYWORDS	nucleotide-binding	20	1.937984	0.020763	201	743	12980	1.738280	0.935984	0.746987	21.704298
GOTERM_BP_FAT	GO:0006944 membrane fusion	3	0.290698	0.020932	121	15	7937	13.119008	1.000000	0.729071	28.175836
GOTERM_BP_FAT	GO:0045333 cellular respiration	6	0.581395	0.021169	121	105	7937	3.748288	1.000000	0.710130	28.447977
INTERPRO	IPR007502:Helicase-associated region	3	0.290698	0.022784	174	14	10196	12.556650	0.999788	0.999788	27.198766
GOTERM_CC_FAT	GO:0044455 mitochondrial membrane part	7	0.678295	0.023809	81	134	4786	3.086604	0.991531	0.411479	26.032115
GOTERM_BP_FAT	GO:0007286 spermatid development	5	0.484496	0.026535	121	75	7937	4.373003	1.000000	0.768898	34.344090
GOTERM_BP_FAT	GO:0016568 chromatin modification	6	0.581395	0.027083	121	112	7937	3.514020	1.000000	0.756463	34.920075
GOTERM_BP_FAT	GO:0048515 spermatid differentiation	5	0.484496	0.027692	121	76	7937	4.315463	1.000000	0.745549	35.554174
GOTERM_CC_FAT	GO:0031090 organelle membrane	14	1.356589	0.028278	81	438	4786	1.888607	0.996586	0.433327	30.159173
GOTERM_BP_FAT	GO:0034728 nucleosome organization	4	0.387597	0.028476	121	44	7937	5.963186	1.000000	0.737516	36.362396
GOTERM_BP_FAT	GO:0015980 energy derivation by oxidation of organic compounds	6	0.581395	0.028948	121	114	7937	3.452371	1.000000	0.726185	36.844470
GOTERM_BP_FAT	GO:0006974 response to DNA damage stimulus	6	0.581395	0.029910	121	115	7937	3.422350	1.000000	0.721449	37.816467
SP_PIR_KEYWORDS	gtp-binding	7	0.678295	0.030270	201	152	12980	2.973946	0.982166	0.738735	30.123831
SP_PIR_KEYWORDS	helicase	5	0.484496	0.030960	201	77	12980	4.193319	0.983753	0.642978	30.701006
GOTERM_BP_FAT	GO:0006338 chromatin remodeling	4	0.387597	0.031941	121	46	7937	5.703917	1.000000	0.729356	39.822075
GOTERM_MF_FAT	GO:0001671 ATPase activator activity	2	0.193798	0.032321	130	2	7918	60.907692	0.999927	0.958245	35.387618
GOTERM_MF_FAT	GO:0004386 helicase activity	6	0.581395	0.032348	130	109	7918	3.352717	0.999928	0.907816	35.411561
GOTERM_BP_FAT	GO:0065003 macromolecular complex assembly	9	0.872093	0.032589	121	246	7937	2.399819	1.000000	0.721495	40.448879
GOTERM_BP_FAT	GO:0048589 developmental growth	5	0.484496	0.032618	121	80	7937	4.099690	1.000000	0.707212	40.477091
GOTERM_BP_FAT	GO:0006289 nucleotide-excision repair	3	0.290698	0.032779	121	19	7937	10.357112	1.000000	0.694868	40.632389
GOTERM_BP_FAT	GO:0070646 protein modification by small protein removal	3	0.290698	0.032779	121	19	7937	10.357112	1.000000	0.694868	40.632389
INTERPRO	IPR006884:Fzo-like conserved region	2	0.193798	0.033649	174	2	10196	58.597701	0.999996	0.998128	37.588812
GOTERM_BP_FAT	GO:0016049 cell growth	4	0.387597	0.035617	121	48	7937	5.466253	1.000000	0.711714	43.299209
GOTERM_MF_FAT	GO:0042624 ATPase activity, uncoupled	8	0.775194	0.037078	130	192	7918	2.537821	0.999983	0.888244	39.485621

GOTERM_BP_FAT	GO:0006793 phosphorus metabolic process	15	1.453488	0.037915	121	551	7937	1.785709	1.000000	0.721490	45.377105
GOTERM_BP_FAT	GO:0006796 phosphate metabolic process	15	1.453488	0.037915	121	551	7937	1.785709	1.000000	0.721490	45.377105
GOTERM_BP_FAT	GO:0006302 double-strand break repair	3	0.290698	0.039477	121	21	7937	9.370720	1.000000	0.723657	46.748320
SP_PIR_KEYWORDS	sh3 domain	4	0.387597	0.039561	201	49	12980	5.271601	0.994947	0.652701	37.544368
GOTERM_MF_FAT	GO:0032555 purine ribonucleotide binding	23	2.228682	0.042112	130	923	7918	1.517743	0.999996	0.875010	43.558545
GOTERM_MF_FAT	GO:0032553 ribonucleotide binding	23	2.228682	0.042112	130	923	7918	1.517743	0.999996	0.875010	43.558545
SP_PIR_KEYWORDS	hydrolase	32	3.100775	0.043161	201	1470	12980	1.405760	0.996910	0.618361	40.219828
GOTERM_BP_FAT	GO:0032434 regulation of proteasomal ubiquitin-dependent protein catabolic process	2	0.193798	0.044680	121	3	7937	43.730028	1.000000	0.756075	51.086479
GOTERM_BP_FAT	GO:0007256 activation of JNKK activity	2	0.193798	0.044680	121	3	7937	43.730028	1.000000	0.756075	51.086479
GOTERM_MF_FAT	GO:0003724 RNA helicase activity	4	0.387597	0.045225	130	49	7918	4.972057	0.999999	0.852999	45.949046
GOTERM_MF_FAT	GO:0008289 lipid binding	6	0.581395	0.047289	130	121	7918	3.020216	0.999999	0.827282	47.481861
GOTERM_CC_FAT	GO:0005746 mitochondrial respiratory chain	5	0.484496	0.047450	81	82	4786	3.602830	0.999934	0.583153	45.573129
GOTERM_MF_FAT	GO:0004519 endonuclease activity	4	0.387597	0.047558	130	50	7918	4.872615	0.999999	0.791967	47.678294
GOTERM_MF_FAT	GO:0060590 ATPase regulator activity	2	0.193798	0.048090	130	3	7918	40.605128	0.999999	0.760514	48.065554
GOTERM_BP_FAT	GO:0032989 cellular component morphogenesis	14	1.356589	0.048588	121	518	7937	1.772839	1.000000	0.774135	54.124651
GOTERM_BP_FAT	GO:0048284 organelle fusion	3	0.290698	0.050393	121	24	7937	8.199380	1.000000	0.776034	55.467189
GOTERM_BP_FAT	GO:0034637 cellular carbohydrate biosynthetic process	3	0.290698	0.050393	121	24	7937	8.199380	1.000000	0.776034	55.467189
GOTERM_CC_FAT	GO:0070469 respiratory chain	5	0.484496	0.052933	81	85	4786	3.475672	0.999979	0.592357	49.365925
GOTERM_BP_FAT	GO:0016310 phosphorylation	12	1.162791	0.056135	121	425	7937	1.852095	1.000000	0.802324	59.498870
UP_SEQ_FEATURE	mutagenesis site	8	0.775194	0.057909	53	201	2975	2.234112	0.999963	0.993905	51.735630
GOTERM_BP_FAT	GO:0000186 activation of MAPKK activity	2	0.193798	0.059129	121	4	7937	32.797521	1.000000	0.809858	61.462908
INTERPRO	IPR005225:Small GTP-binding protein	5	0.484496	0.060287	174	87	10196	3.367684	1.000000	0.999503	57.532237
SP_PIR_KEYWORDS	iron-sulfur	3	0.290698	0.060288	201	26	12980	7.451206	0.999710	0.687668	51.573673
GOTERM_MF_FAT	GO:0016887 ATPase activity	11	1.065891	0.061831	130	353	7918	1.897973	1.000000	0.814119	57.193479
GOTERM_BP_FAT	GO:0007243 protein kinase cascade	4	0.387597	0.061957	121	60	7937	4.373003	1.000000	0.815881	63.235212
GOTERM_BP_FAT	GO:0032535 regulation of cellular component size	5	0.484496	0.062623	121	99	7937	3.312881	1.000000	0.810517	63.641841
GOTERM_MF_FAT	GO:0042623 ATPase activity, coupled	10	0.968992	0.062757	130	307	7918	1.983964	1.000000	0.791186	57.752170
GOTERM_MF_FAT	GO:0004518 nuclease activity	5	0.484496	0.069048	130	95	7918	3.205668	1.000000	0.797309	61.370470
GOTERM_MF_FAT	GO:0005525 GTP binding	7	0.678295	0.070306	130	178	7918	2.395246	1.000000	0.779106	62.058656
GOTERM_BP_FAT	GO:0007281 germ cell development	8	0.775194	0.074864	121	243	7937	2.159508	1.000000	0.857364	70.399894
GOTERM_MF_FAT	GO:0032561 guanyl ribonucleotide binding	7	0.678295	0.074898	130	181	7918	2.355546	1.000000	0.778009	64.475344
SP_PIR_KEYWORDS	chromatin regulator	4	0.387597	0.075873	201	64	12980	4.036070	0.999968	0.725303	60.154248
GOTERM_MF_FAT	GO:0019001 guanyl nucleotide binding	7	0.678295	0.076465	130	182	7918	2.342604	1.000000	0.763496	65.267425
GOTERM_MF_FAT	GO:0000166 nucleotide binding	27	2.616279	0.077729	130	1206	7918	1.363605	1.000000	0.748505	65.893813
GOTERM_MF_FAT	GO:0017076 purine nucleotide binding	23	2.228682	0.078171	130	989	7918	1.416458	1.000000	0.730548	66.110317
SP_PIR_KEYWORDS	phosphoprotein	19	1.841085	0.078466	201	815	12980	1.505479	0.999978	0.695600	61.438466
GOTERM_BP_FAT	GO:0070647 protein modification by small protein conjugation or removal	4	0.387597	0.080506	121	67	7937	3.916122	1.000000	0.870656	73.101441
GOTERM_CC_FAT	GO:0001673 male germ cell nucleus	2	0.193798	0.080862	81	5	4786	23.634568	1.000000	0.723140	65.184671
INTERPRO	IPR016561:Dynein light chain, roadblock-type	2	0.193798	0.082022	174	5	10196	23.439080	1.000000	0.999611	69.233354
INTERPRO	IPR017923:TFIIS N-terminal	2	0.193798	0.082022	174	5	10196	23.439080	1.000000	0.999611	69.233354
GOTERM_BP_FAT	GO:0007264 small GTPase mediated signal transduction	5	0.484496	0.082728	121	109	7937	3.008947	1.000000	0.871303	74.100700
GOTERM_BP_FAT	GO:0040007 growth	5	0.484496	0.084895	121	110	7937	2.981593	1.000000	0.871753	75.041647
GOTERM_BP_FAT	GO:0048232 male gamete generation	6	0.581395	0.085404	121	155	7937	2.539163	1.000000	0.866848	75.257829
GOTERM_BP_FAT	GO:0007283 spermatogenesis	6	0.581395	0.085404	121	155	7937	2.539163	1.000000	0.866848	75.257829
GOTERM_CC_FAT	GO:0043073 germ cell nucleus	2	0.193798	0.096242	81	6	4786	19.695473	1.000000	0.760970	71.811847
INTERPRO	IPR001401:Dynamin, GTPase region	2	0.193798	0.097606	174	6	10196	19.532567	1.000000	0.999468	75.696669
INTERPRO	IPR017900:4Fe-4S ferredoxin, iron-sulphur binding, conserved site	2	0.193798	0.097606	174	6	10196	19.532567	1.000000	0.999468	75.696669
PIR_SUPERFAMILY	PIRSF009998:DLC7	2	0.193798	0.098069	54	5	2596	19.229630	0.997734	0.997734	64.324505
GOTERM_BP_FAT	GO:0007005 mitochondrion organization	4	0.387597	0.098089	121	73	7937	3.594249	1.000000	0.897324	80.114189
SP_PIR_KEYWORDS	transit peptide	5	0.484496	0.099452	201	114	12980	2.832330	0.999999	0.746465	70.522798

Table 22: Architecture 22

Category	Term	Count	%	PValue	List Total	Pop Hits	Pop Total	Fold Enrichment	Bonferroni	Benjamini	FDR
GOTERM_BP_FAT	GO:0008104 protein localization	9	1.477833	0.005220	56	392	7937	3.254054	0.949364	0.949364	7.388055
GOTERM_CC_FAT	GO:0044429 mitochondrial part	10	1.642036	0.006809	42	411	4786	2.772564	0.643616	0.643616	7.844089
GOTERM_CC_FAT	GO:0005739 mitochondrion	12	1.970443	0.006953	42	571	4786	2.394796	0.651313	0.409503	8.003263
GOTERM_CC_FAT	GO:0043232 intracellular non-membrane-bounded organelle	15	2.463054	0.020932	42	943	4786	1.812604	0.959004	0.655189	22.345930
GOTERM_CC_FAT	GO:0043228 non-membrane-bounded organelle	15	2.463054	0.020932	42	943	4786	1.812604	0.959004	0.655189	22.345930
GOTERM_CC_FAT	GO:0005743 mitochondrial inner membrane	6	0.985222	0.026746	42	200	4786	3.418571	0.983321	0.640629	27.682938
GOTERM_CC_FAT	GO:0019866 organelle inner membrane	6	0.985222	0.036311	42	217	4786	3.150757	0.996246	0.672739	35.737201
GOTERM_MF_FAT	GO:0032553 ribonucleotide binding	12	1.970443	0.039263	54	923	7918	1.906344	0.997988	0.997988	38.185444
GOTERM_MF_FAT	GO:0032555 purine ribonucleotide binding	12	1.970443	0.039263	54	923	7918	1.906344	0.997988	0.997988	38.185444
GOTERM_BP_FAT	GO:0015031 protein transport	6	0.985222	0.042589	56	278	7937	3.058967	1.000000	0.999996	47.179995
GOTERM_BP_FAT	GO:0045184 establishment of protein localization	6	0.985222	0.046561	56	285	7937	2.983835	1.000000	0.999884	50.303882
KEGG_PATHWAY	dme04914:Progesterone-mediated oocyte maturation	3	0.492611	0.048617	19	41	2054	7.910141	0.612072	0.612072	31.168800
GOTERM_CC_FAT	GO:0031974 membrane-enclosed lumen	10	1.642036	0.048641	42	571	4786	1.995663	0.999463	0.714899	44.906839
GOTERM_CC_FAT	GO:0030125 clathrin vesicle coat	2	0.328407	0.050337	42	6	4786	37.984127	0.999590	0.671800	46.069665
GOTERM_MF_FAT	GO:0000166 nucleotide binding	14	2.298851	0.050993	54	1206	7918	1.702168	0.999700	0.982686	46.663801
GOTERM_CC_FAT	GO:0031966 mitochondrial membrane	6	0.985222	0.053076	42	241	4786	2.836989	0.999735	0.642767	47.899636
GOTERM_BP_FAT	GO:0048610 reproductive cellular process	8	1.313629	0.055788	56	500	7937	2.267714	1.000000	0.999720	56.909735
GOTERM_CC_FAT	GO:0005905 coated pit	2	0.328407	0.058483	42	7	4786	32.557823	0.999888	0.636173	51.347431
GOTERM_MF_FAT	GO:0017076 purine nucleotide binding	12	1.970443	0.059882	54	989	7918	1.779126	0.999930	0.958845	52.363845
KEGG_PATHWAY	dme03018:RNA degradation	3	0.492611	0.064514	19	48	2054	6.756579	0.718351	0.469294	39.334678
GOTERM_BP_FAT	GO:0051188 cofactor biosynthetic process	3	0.492611	0.064654	56	60	7937	7.086607	1.000000	0.999509	62.477270
GOTERM_CC_FAT	GO:0005740 mitochondrial envelope	6	0.985222	0.067329	42	258	4786	2.650055	0.999973	0.650938	56.539506
GOTERM_CC_FAT	GO:0005813 centrosome	3	0.492611	0.072574	42	52	4786	6.574176	0.999989	0.644506	59.373502
INTERPRO	IPR011990:Tetratricopeptide-like helical	4	0.656814	0.073193	99	101	10196	4.078808	1.000000	1.000000	62.131815
INTERPRO	IPR001440:Tetratricopeptide TPR-1	3	0.492611	0.074803	99	47	10196	6.573823	1.000000	0.999841	62.963510
GOTERM_CC_FAT	GO:0031090 organelle membrane	8	1.313629	0.075740	42	438	4786	2.081322	0.999993	0.628827	61.000786
GOTERM_CC_FAT	GO:0030665 clathrin coated vesicle membrane	2	0.328407	0.082513	42	10	4786	22.790476	0.999998	0.632224	64.283628
GOTERM_BP_FAT	GO:0007281 germ cell development	5	0.821018	0.087061	56	243	7937	2.916299	1.000000	0.999825	73.705911
GOTERM_BP_FAT	GO:0070727 cellular macromolecule localization	5	0.821018	0.088073	56	244	7937	2.904347	1.000000	0.999451	74.130371
GOTERM_CC_FAT	GO:0005815 microtubule organizing center	3	0.492611	0.092864	42	60	4786	5.697619	1.000000	0.650483	68.814005
GOTERM_BP_FAT	GO:0007286 spermatid development	3	0.492611	0.095003	56	75	7937	5.669286	1.000000	0.999185	76.868170
GOTERM_CC_FAT	GO:0070013 intracellular organelle lumen	9	1.477833	0.095696	42	556	4786	1.844553	1.000000	0.636728	69.958476
GOTERM_CC_FAT	GO:0043233 organelle lumen	9	1.477833	0.095696	42	556	4786	1.844553	1.000000	0.636728	69.958476
GOTERM_BP_FAT	GO:0048515 spermatid differentiation	3	0.492611	0.097151	56	76	7937	5.594690	1.000000	0.998455	77.660691
SMART	SM00028:TPR	3	0.492611	0.098173	43	61	4824	5.517347	0.992987	0.992987	62.699390

Table 23: Architecture 23

Category	Term	Count	%	PValue	List Total	Pop Hits	Pop Total	Fold Enrichment	Bonferroni	Benjamini	FDR
GOTERM_CC_FAT	GO:0030135 coated vesicle	7	0.923483	2.271744e-04	57	76	4786	7.733610	0.036356	0.036356	0.274833
SP_PIR_KEYWORDS	cytoplasm	15	1.978892	6.854895e-04	155	436	12980	2.881030	0.077728	0.077728	0.781698
GOTERM_MF_FAT	GO:0008047 enzyme activator activity	7	0.923483	8.339100e-04	98	91	7918	6.215071	0.179400	0.179400	1.068918
GOTERM_BP_FAT	GO:0015031 protein transport	12	1.583113	8.384265e-04	103	278	7937	3.326256	0.532694	0.532694	1.300226
GOTERM_CC_FAT	GO:0016023 cytoplasmic membrane-bounded vesicle	7	0.923483	8.975535e-04	57	98	4786	5.997494	0.136159	0.070570	1.081817
GOTERM_BP_FAT	GO:0045184 establishment of protein localization	12	1.583113	0.001028	103	285	7937	3.244558	0.606656	0.372829	1.592345
GOTERM_CC_FAT	GO:0031988 membrane-bounded vesicle	7	0.923483	0.001108	57	102	4786	5.762298	0.165271	0.058439	1.333501
GOTERM_CC_FAT	GO:0031410 cytoplasmic vesicle	7	0.923483	0.001289	57	105	4786	5.597661	0.189565	0.051189	1.549823
GOTERM_BP_FAT	GO:0048477 oogenesis	17	2.242744	0.001316	103	539	7937	2.430409	0.697067	0.328393	2.033493
GOTERM_BP_FAT	GO:0007292 female gamete generation	17	2.242744	0.001506	103	546	7937	2.399250	0.745081	0.289440	2.323887
GOTERM_BP_FAT	GO:0007267 cell-cell signaling	10	1.319261	0.001507	103	211	7937	3.652050	0.745406	0.239377	2.326032
GOTERM_CC_FAT	GO:0031982 vesicle	7	0.923483	0.001718	57	111	4786	5.295085	0.244416	0.054511	2.061220
GOTERM_MF_FAT	GO:0046982 protein heterodimerization activity	5	0.659631	0.002522	98	47	7918	8.595310	0.450398	0.258648	3.201028
GOTERM_BP_FAT	GO:0007268 synaptic transmission	9	1.187335	0.002897	103	189	7937	3.669441	0.928024	0.355042	4.425895
GOTERM_CC_FAT	GO:0008021 synaptic vesicle	5	0.659631	0.003445	57	54	4786	7.774529	0.430187	0.089482	4.093623
GOTERM_BP_FAT	GO:0019226 transmission of nerve impulse	9	1.187335	0.003620	103	196	7937	3.538389	0.962734	0.374969	5.502067
GOTERM_BP_FAT	GO:0008104 protein localization	13	1.715040	0.004287	103	392	7937	2.555503	0.979684	0.385558	6.483127
GOTERM_CC_FAT	GO:0030136 clathrin-coated vesicle	5	0.659631	0.004748	57	59	4786	7.115671	0.539630	0.104898	5.601707
INTERPRO	IPR019781:WD40 repeat, subgroup	8	1.055409	0.004874	139	154	10196	3.810520	0.731348	0.731348	6.218730
GOTERM_CC_FAT	GO:0030117 membrane coat	4	0.527704	0.004926	57	30	4786	11.195322	0.552868	0.095717	5.806162
GOTERM_CC_FAT	GO:0048475 coated membrane	4	0.527704	0.004926	57	30	4786	11.195322	0.552868	0.095717	5.806162
GOTERM_BP_FAT	GO:0050807 regulation of synapse organization	4	0.527704	0.005908	103	29	7937	10.628724	0.995366	0.449626	8.831056
GOTERM_BP_FAT	GO:0031396 regulation of protein ubiquitination	3	0.395778	0.006881	103	10	7937	23.117476	0.998094	0.465423	10.213774
GOTERM_CC_FAT	GO:0044456 synapse part	6	0.791557	0.006980	57	104	4786	4.844130	0.680724	0.119139	8.134457
GOTERM_MF_FAT	GO:0030695 GTPase regulator activity	7	0.923483	0.007777	98	142	7918	3.982897	0.842821	0.460326	9.568168
GOTERM_MF_FAT	GO:0060589 nucleoside-triphosphatase regulator activity	7	0.923483	0.008582	98	145	7918	3.900493	0.870328	0.399917	10.508847
SMART	SM00320:WD40	8	1.055409	0.008721	62	187	4824	3.328618	0.424099	0.424099	8.487073
GOTERM_BP_FAT	GO:0007298 border follicle cell migration	5	0.659631	0.010077	103	66	7937	5.837746	0.999898	0.566172	14.617528
GOTERM_BP_FAT	GO:0040008 regulation of growth	6	0.791557	0.010299	103	103	7937	4.488830	0.999916	0.542731	14.916102
GOTERM_MF_FAT	GO:0005096 GTPase activator activity	5	0.659631	0.012093	98	73	7918	5.533967	0.944065	0.438259	14.506678
INTERPRO	IPR019782:WD40 repeat 2	7	0.923483	0.013411	139	144	10196	3.565747	0.973539	0.837331	16.257679
INTERPRO	IPR001680:WD40 repeat	8	1.055409	0.013497	139	187	10196	3.138076	0.974153	0.704331	16.353638
GOTERM_BP_FAT	GO:0019748 secondary metabolic process	5	0.659631	0.013570	103	72	7937	5.351268	0.999996	0.614525	19.199702
GOTERM_BP_FAT	GO:0007242 intracellular signaling cascade	10	1.319261	0.013883	103	297	7937	2.594554	0.999997	0.595763	19.598960
SP_PIR_KEYWORDS	wd repeat	7	0.923483	0.014221	155	166	12980	3.531286	0.815502	0.570468	15.118869
GOTERM_BP_FAT	GO:0050803 regulation of synapse structure and activity	4	0.527704	0.014431	103	40	7937	7.705825	0.999998	0.584771	20.292111
GOTERM_BP_FAT	GO:0009798 axis specification	8	1.055409	0.014650	103	201	7937	3.066995	0.999998	0.566828	20.568662
GOTERM_CC_FAT	GO:0045202 synapse	6	0.791557	0.014792	57	125	4786	4.030316	0.911883	0.215657	16.516001
INTERPRO	IPR017986:WD40 repeat, region	7	0.923483	0.016121	139	150	10196	3.423118	0.987374	0.664789	19.230460
INTERPRO	IPR015943:WD40/YVTN repeat-like	8	1.055409	0.016647	139	195	10196	3.009334	0.989066	0.594716	19.796060
GOTERM_BP_FAT	GO:0007413 axonal fasciculation	3	0.395778	0.017453	103	16	7937	14.448422	1.000000	0.609136	24.022005
GOTERM_BP_FAT	GO:0007297 ovarian follicle cell migration	5	0.659631	0.017753	103	78	7937	4.939632	1.000000	0.594486	24.383293
GOTERM_BP_FAT	GO:0007276 gamete generation	17	2.242744	0.019546	103	715	7937	1.832154	1.000000	0.610275	26.508553
GOTERM_BP_FAT	GO:0006900 membrane budding	3	0.395778	0.021886	103	18	7937	12.843042	1.000000	0.633419	29.197453
GOTERM_CC_FAT	GO:0070013 intracellular organelle lumen	13	1.715040	0.024646	57	556	4786	1.963208	0.982882	0.309117	26.087030
GOTERM_CC_FAT	GO:0043233 organelle lumen	13	1.715040	0.024646	57	556	4786	1.963208	0.982882	0.309117	26.087030
GOTERM_BP_FAT	GO:0019953 sexual reproduction	17	2.242744	0.024652	103	735	7937	1.782300	1.000000	0.659746	32.257955
GOTERM_BP_FAT	GO:0009057 macromolecule catabolic process	9	1.187335	0.025793	103	277	7937	2.503698	1.000000	0.659490	33.483808
GOTERM_BP_FAT	GO:0000902 cell morphogenesis	12	1.583113	0.025975	103	442	7937	2.092079	1.000000	0.645783	33.677598
GOTERM_CC_FAT	GO:0031974 membrane-enclosed lumen	13	1.715040	0.029684	57	571	4786	1.911635	0.992641	0.335895	30.581301
GOTERM_CC_FAT	GO:0030529 ribonucleoprotein complex	10	1.319261	0.030183	57	379	4786	2.215433	0.993232	0.319054	31.012052
GOTERM_BP_FAT	GO:0008582 regulation of synaptic growth at neuromuscular junction	3	0.395778	0.031965	103	22	7937	10.507944	1.000000	0.707047	39.763948
GOTERM_BP_FAT	GO:0051147 regulation of muscle cell differentiation	3	0.395778	0.031965	103	22	7937	10.507944	1.000000	0.707047	39.763948
GOTERM_BP_FAT	GO:0048742 regulation of skeletal muscle fiber development	3	0.395778	0.031965	103	22	7937	10.507944	1.000000	0.707047	39.763948

GOTERM_BP_FAT	GO:0048641 regulation of skeletal muscle tissue development	3	0.395778	0.031965	103	22	7937	10.507944	1.000000	0.707047	39.763948
GOTERM_BP_FAT	GO:0051153 regulation of striated muscle cell differentiation	3	0.395778	0.031965	103	22	7937	10.507944	1.000000	0.707047	39.763948
GOTERM_BP_FAT	GO:0051963 regulation of synaptogenesis	3	0.395778	0.034721	103	23	7937	10.051076	1.000000	0.722537	42.384807
PIR_SUPERFAMILY	PIRSF001710:Ras-related protein Rab	3	0.395778	0.035242	40	20	2596	9.735000	0.855925	0.855925	29.635589
GOTERM_BP_FAT	GO:0048534 hemopoietic or lymphoid organ development	4	0.527704	0.036573	103	57	7937	5.407597	1.000000	0.727397	44.085297
GOTERM_BP_FAT	GO:0002520 immune system development	4	0.527704	0.036573	103	57	7937	5.407597	1.000000	0.727397	44.085297
GOTERM_BP_FAT	GO:0030182 neuron differentiation	11	1.451187	0.036800	103	409	7937	2.072471	1.000000	0.716210	44.290625
GOTERM_BP_FAT	GO:0000578 embryonic axis specification	6	0.791557	0.038581	103	145	7937	3.188617	1.000000	0.720425	45.876292
KEGG_PATHWAY	dme04320:Dorso-ventral axis formation	3	0.395778	0.038673	26	26	2054	9.115385	0.668569	0.668569	28.103683
GOTERM_BP_FAT	GO:0048609 reproductive process in a multicellular organism	17	2.242744	0.038998	103	778	7937	1.683792	1.000000	0.711803	46.241574
GOTERM_BP_FAT	GO:0032504 multicellular organism reproduction	17	2.242744	0.038998	103	778	7937	1.683792	1.000000	0.711803	46.241574
GOTERM_MF_FAT	GO:0046983 protein dimerization activity	5	0.659631	0.039596	98	105	7918	3.847425	0.999931	0.797262	40.573896
SMART	SM00028:TPR	4	0.527704	0.041242	62	61	4824	5.102062	0.929586	0.734644	34.718183
KEGG_PATHWAY	dme04150:mTOR signaling pathway	3	0.395778	0.041459	26	27	2054	8.777778	0.694439	0.447225	29.828446
GOTERM_BP_FAT	GO:0007243 protein kinase cascade	4	0.527704	0.041620	103	60	7937	5.137217	1.000000	0.723416	48.485041
GOTERM_BP_FAT	GO:0007269 neurotransmitter secretion	5	0.659631	0.041981	103	102	7937	3.777365	1.000000	0.714869	48.787066
GOTERM_MF_FAT	GO:0005083 small GTPase regulator activity	5	0.659631	0.043193	98	108	7918	3.740552	0.999971	0.775736	43.378571
GOTERM_BP_FAT	GO:0003001 generation of a signal involved in cell-cell signaling	5	0.659631	0.043261	103	103	7937	3.740692	1.000000	0.714494	49.844412
GOTERM_BP_FAT	GO:0030707 ovarian follicle cell development	7	0.923483	0.047195	103	204	7937	2.644156	1.000000	0.735194	52.967527
INTERPRO	IPR011990: Tetratricopeptide-like helical	5	0.659631	0.047989	139	101	10196	3.631313	0.999998	0.889734	47.598773
INTERPRO	IPR019734: Tetratricopeptide repeat	4	0.527704	0.049452	139	61	10196	4.810001	0.999999	0.857580	48.647079
GOTERM_BP_FAT	GO:0016333 morphogenesis of follicular epithelium	3	0.395778	0.049781	103	28	7937	8.256241	1.000000	0.743895	54.920081
GOTERM_MF_FAT	GO:0008092 cytoskeletal protein binding	7	0.923483	0.050751	98	218	7918	2.594364	0.999996	0.786260	48.877089
GOTERM_BP_FAT	GO:0007264 small GTPase mediated signal transduction	5	0.659631	0.051401	103	109	7937	3.534782	1.000000	0.745250	56.104573
GOTERM_BP_FAT	GO:0007169 transmembrane receptor protein tyrosine kinase signaling pathway	5	0.659631	0.051401	103	109	7937	3.534782	1.000000	0.745250	56.104573
GOTERM_BP_FAT	GO:0001505 regulation of neurotransmitter levels	5	0.659631	0.052834	103	110	7937	3.502648	1.000000	0.745277	57.127923
GOTERM_BP_FAT	GO:0048610 reproductive cellular process	12	1.583113	0.055412	103	500	7937	1.849398	1.000000	0.752768	58.912873
GOTERM_BP_FAT	GO:0016202 regulation of striated muscle tissue development	3	0.395778	0.056354	103	30	7937	7.705825	1.000000	0.749543	59.547561
GOTERM_BP_FAT	GO:0007040 lysosome organization	3	0.395778	0.056354	103	30	7937	7.705825	1.000000	0.749543	59.547561
GOTERM_BP_FAT	GO:0002164 larval development	4	0.527704	0.056686	103	68	7937	4.532838	1.000000	0.742608	59.769108
SMART	SM00175:RAB	3	0.395778	0.057856	62	31	4824	7.529657	0.976591	0.713938	45.308232
GOTERM_BP_FAT	GO:0009950 dorsal/ventral axis specification	4	0.527704	0.058729	103	69	7937	4.467145	1.000000	0.746499	61.107326
GOTERM_BP_FAT	GO:0003006 reproductive developmental process	12	1.583113	0.059384	103	506	7937	1.827468	1.000000	0.741877	61.527669
SMART	SM00745:MIT	2	0.263852	0.061672	62	5	4824	31.122581	0.981872	0.633065	47.509828
GOTERM_BP_FAT	GO:0016071 mRNA metabolic process	7	0.923483	0.062441	103	219	7937	2.463049	1.000000	0.751511	63.432576
GOTERM_BP_FAT	GO:0007033 vacuole organization	3	0.395778	0.063212	103	32	7937	7.224211	1.000000	0.747749	63.898962
GOTERM_BP_FAT	GO:0016192 vesicle-mediated transport	10	1.319261	0.063515	103	391	7937	1.970799	1.000000	0.741460	64.081018
GOTERM_BP_FAT	GO:0032940 secretion by cell	5	0.659631	0.065076	103	118	7937	3.265180	1.000000	0.742381	65.004037
INTERPRO	IPR003579:Ras small GTPase, Rab type	3	0.395778	0.065525	139	31	10196	7.098631	1.000000	0.897592	58.956864
INTERPRO	IPR007330:MIT	2	0.263852	0.065879	139	5	10196	29.341007	1.000000	0.869568	59.160466
GOTERM_BP_FAT	GO:0016050 vesicle organization	3	0.395778	0.066741	103	33	7937	7.005296	1.000000	0.743836	65.963973
GOTERM_BP_FAT	GO:0008293 torso signaling pathway	3	0.395778	0.066741	103	33	7937	7.005296	1.000000	0.743836	65.963973
GOTERM_BP_FAT	GO:0048634 regulation of muscle development	3	0.395778	0.066741	103	33	7937	7.005296	1.000000	0.743836	65.963973
GOTERM_BP_FAT	GO:0032989 cellular component morphogenesis	12	1.583113	0.067890	103	518	7937	1.785133	1.000000	0.742499	66.611904
GOTERM_CC_FAT	GO:0005732 small nucleolar ribonucleoprotein complex	2	0.263852	0.068217	57	6	4786	27.988304	0.999990	0.560728	57.508561
GOTERM_BP_FAT	GO:0044265 cellular macromolecule catabolic process	7	0.923483	0.069272	103	225	7937	2.397368	1.000000	0.742441	67.376087
GOTERM_BP_FAT	GO:0006886 intracellular protein transport	6	0.791557	0.073006	103	174	7937	2.657181	1.000000	0.754197	69.359265

GOTERM_BP_FAT	GO:0048812 neuron projection morphogenesis	8	1.055409	0.074659	103	286	7937	2.155476	1.000000	0.755254	70.200764
GOTERM_BP_FAT	GO:0031175 neuron projection development	8	1.055409	0.075751	103	287	7937	2.147965	1.000000	0.753633	70.744710
GOTERM_BP_FAT	GO:0009880 embryonic pattern specification	7	0.923483	0.076524	103	231	7937	2.335099	1.000000	0.750575	71.124429
GOTERM_BP_FAT	GO:0048667 cell morphogenesis involved in neuron differentiation	8	1.055409	0.076852	103	288	7937	2.140507	1.000000	0.745505	71.283946
GOTERM_BP_FAT	GO:0046903 secretion	5	0.659631	0.076903	103	125	7937	3.082330	1.000000	0.739216	71.308840
GOTERM_BP_FAT	GO:0045165 cell fate commitment	7	0.923483	0.077774	103	232	7937	2.325033	1.000000	0.736889	71.728001
GOTERM_BP_FAT	GO:0006836 neurotransmitter transport	5	0.659631	0.078676	103	126	7937	3.057867	1.000000	0.734780	72.156574
GOTERM_BP_FAT	GO:0034613 cellular protein localization	6	0.791557	0.080229	103	179	7937	2.582958	1.000000	0.735721	72.879776
GOTERM_BP_FAT	GO:0008283 cell proliferation	5	0.659631	0.080469	103	127	7937	3.033789	1.000000	0.730690	72.990195
GOTERM_MF_FAT	GO:0008171 O-methyltransferase activity	2	0.263852	0.082696	98	7	7918	23.084548	1.000000	0.896997	67.106952
GOTERM_BP_FAT	GO:0044087 regulation of cellular component biogenesis	4	0.527704	0.083405	103	80	7937	3.852913	1.000000	0.737845	74.304858
GOTERM_BP_FAT	GO:0032268 regulation of cellular protein metabolic process	5	0.659631	0.085970	103	130	7937	2.963779	1.000000	0.743049	75.404159
GOTERM_BP_FAT	GO:0000278 mitotic cell cycle	9	1.187335	0.088933	103	358	7937	1.937219	1.000000	0.749644	76.619250
GOTERM_BP_FAT	GO:0007300 ovarian nurse cell to oocyte transport	3	0.395778	0.089194	103	39	7937	5.927558	1.000000	0.745058	76.723337
GOTERM_BP_FAT	GO:0050877 neurological system process	12	1.583113	0.090721	103	546	7937	1.693588	1.000000	0.745687	77.325022
INTERPRO	IPR002553: Clathrin/ coatomer adaptor, adaptin-like, N-terminal	2	0.263852	0.091007	139	7	10196	20.957862	1.000000	0.923216	71.459650
GOTERM_BP_FAT	GO:0043087 regulation of GTPase activity	3	0.395778	0.093129	103	40	7937	5.779369	1.000000	0.749768	78.243857
GOTERM_BP_FAT	GO:0006364 rRNA processing	3	0.395778	0.093129	103	40	7937	5.779369	1.000000	0.749768	78.243857
GOTERM_BP_FAT	GO:0048638 regulation of developmental growth	3	0.395778	0.093129	103	40	7937	5.779369	1.000000	0.749768	78.243857
GOTERM_CC_FAT	GO:0043005 neuron projection	3	0.395778	0.093139	57	44	4786	5.724880	1.000000	0.654370	69.402555
GOTERM_BP_FAT	GO:0070727 cellular macromolecule localization	7	0.923483	0.093667	103	244	7937	2.210688	1.000000	0.746487	78.444472
GOTERM_BP_FAT	GO:0000904 cell morphogenesis involved in differentiation	8	1.055409	0.094505	103	303	7937	2.034541	1.000000	0.744429	78.753326
GOTERM_MF_FAT	GO:0003702 RNA polymerase II transcription factor activity	7	0.923483	0.094942	98	257	7918	2.200667	1.000000	0.905978	72.335950
GOTERM_BP_FAT	GO:0007049 cell cycle	13	1.715040	0.095847	103	616	7937	1.626229	1.000000	0.744358	79.239452
SMART	SM00246:WH2	2	0.263852	0.096862	62	8	4824	19.451613	0.998369	0.722987	64.356161
GOTERM_BP_FAT	GO:0016072 rRNA metabolic process	3	0.395778	0.097113	103	41	7937	5.638409	1.000000	0.744008	79.688508
GOTERM_BP_FAT	GO:0051964 negative regulation of synaptogenesis	2	0.263852	0.098344	103	8	7937	19.264563	1.000000	0.743541	80.116120
GOTERM_BP_FAT	GO:0051961 negative regulation of nervous system development	2	0.263852	0.098344	103	8	7937	19.264563	1.000000	0.743541	80.116120

Table 24: Architecture 24

Category	Term	Count	%	PValue	List Total	Pop Hits	Pop Total	Fold Enrichment	Bonferroni	Benjamini	FDR
GOTERM_BP_FAT	GO:0048489 synaptic vesicle transport	11	0.651659	2.369121e-05	174	90	7937	5.575160	0.025793	0.025793	0.037892
GOTERM_CC_FAT	GO:0012505 endomembrane system	17	1.007109	1.214124e-04	124	218	4786	3.009840	0.033309	0.033309	0.160328
SP_PIR_KEYWORDS	phosphoprotein	35	2.073460	1.426416e-04	279	815	12980	1.997933	0.024794	0.024794	0.174898
GOTERM_BP_FAT	GO:0051169 nuclear transport	9	0.533175	1.580330e-04	174	72	7937	5.701868	0.159975	0.083471	0.252507
GOTERM_BP_FAT	GO:0006913 nucleocytoplasmic transport	9	0.533175	1.580330e-04	174	72	7937	5.701868	0.159975	0.083471	0.252507
SP_PIR_KEYWORDS	cytoplasm	23	1.362559	1.767023e-04	279	436	12980	2.454211	0.030624	0.015431	0.216620
GOTERM_BP_FAT	GO:0007269 neurotransmitter secretion	10	0.592417	3.630125e-04	174	102	7937	4.472053	0.329999	0.124965	0.579136
GOTERM_BP_FAT	GO:0003001 generation of a signal involved in cell-cell signaling	10	0.592417	3.904393e-04	174	103	7937	4.428635	0.349971	0.102089	0.622763
GOTERM_BP_FAT	GO:0001505 regulation of neurotransmitter levels	10	0.592417	6.342375e-04	174	110	7937	4.146813	0.503308	0.130605	1.009783
GOTERM_CC_FAT	GO:0005643 nuclear pore	7	0.414692	9.140225e-04	124	45	4786	6.003943	0.225183	0.119763	1.201166
GOTERM_BP_FAT	GO:0034622 cellular macromolecular complex assembly	12	0.710900	9.165683e-04	174	165	7937	3.317450	0.636305	0.155130	1.456207
GOTERM_BP_FAT	GO:0032940 secretion by cell	10	0.592417	0.001053	174	118	7937	3.865673	0.687052	0.152920	1.670752
GOTERM_BP_FAT	GO:0015031 protein transport	16	0.947867	0.001058	174	278	7937	2.625320	0.688890	0.135800	1.679153
GOTERM_CC_FAT	GO:0031967 organelle envelope	20	1.184834	0.001089	124	344	4786	2.243998	0.262150	0.096373	1.429675
GOTERM_CC_FAT	GO:0031975 envelope	20	1.184834	0.001128	124	345	4786	2.237494	0.270071	0.075685	1.480050
GOTERM_CC_FAT	GO:0046930 pore complex	7	0.414692	0.001293	124	48	4786	5.628696	0.303054	0.069664	1.695591
GOTERM_BP_FAT	GO:0045184 establishment of protein localization	16	0.947867	0.001359	174	285	7937	2.560839	0.776770	0.153476	2.151359
GOTERM_BP_FAT	GO:0034621 cellular macromolecular complex subunit organization	13	0.770142	0.001566	174	203	7937	2.921154	0.822450	0.158736	2.475728
GOTERM_BP_FAT	GO:0046903 secretion	10	0.592417	0.001581	174	125	7937	3.649195	0.825415	0.146720	2.499548
GOTERM_BP_FAT	GO:0006836 neurotransmitter transport	10	0.592417	0.001671	174	126	7937	3.620234	0.842003	0.142526	2.640616
GOTERM_BP_FAT	GO:0006796 phosphate metabolic process	24	1.421801	0.001878	174	551	7937	1.986858	0.874238	0.147421	2.962283
GOTERM_BP_FAT	GO:0006793 phosphorus metabolic process	24	1.421801	0.001878	174	551	7937	1.986858	0.874238	0.147421	2.962283
GOTERM_BP_FAT	GO:0051640 organelle localization	8	0.473934	0.001991	174	82	7937	4.450238	0.889033	0.145328	3.138277
SP_PIR_KEYWORDS	exocytosis	4	0.236967	0.002370	279	13	12980	14.314861	0.341430	0.129971	2.870228
GOTERM_BP_FAT	GO:0046907 intracellular transport	16	0.947867	0.003375	174	313	7937	2.331754	0.975970	0.220080	5.263877
GOTERM_BP_FAT	GO:0051028 mRNA transport	5	0.296209	0.004868	174	32	7937	7.127335	0.995404	0.285666	7.509402
GOTERM_BP_FAT	GO:0016192 vesicle-mediated transport	18	1.066351	0.004975	174	391	7937	2.099921	0.995918	0.276458	7.668461
GOTERM_BP_FAT	GO:0008104 protein localization	18	1.066351	0.005103	174	392	7937	2.094564	0.996457	0.269111	7.858060
SP_PIR_KEYWORDS	mRNA transport	4	0.236967	0.005290	279	17	12980	10.946658	0.606839	0.208150	6.301582
GOTERM_BP_FAT	GO:0016079 synaptic vesicle exocytosis	6	0.355450	0.006198	174	54	7937	5.068327	0.998949	0.302992	9.467961
GOTERM_CC_FAT	GO:0005635 nuclear envelope	8	0.473934	0.006245	124	86	4786	3.590398	0.825846	0.252710	7.945239
GOTERM_BP_FAT	GO:0048488 synaptic vesicle endocytosis	5	0.296209	0.006739	174	35	7937	6.516420	0.999423	0.311256	10.251710
GOTERM_BP_FAT	GO:0006606 protein import into nucleus	5	0.296209	0.007454	174	36	7937	6.335409	0.999739	0.324967	11.280758
GOTERM_BP_FAT	GO:0007268 synaptic transmission	11	0.651659	0.008167	174	189	7937	2.654838	0.999882	0.337100	12.294011
GOTERM_BP_FAT	GO:0051170 nuclear import	5	0.296209	0.008218	174	37	7937	6.164181	0.999889	0.326829	12.367130
GOTERM_BP_FAT	GO:0034504 protein localization in nucleus	5	0.296209	0.009032	174	38	7937	6.001966	0.999955	0.340973	13.510347
GOTERM_BP_FAT	GO:0043623 cellular protein complex assembly	7	0.414692	0.009294	174	83	7937	3.847043	0.999966	0.337653	13.875056
GOTERM_BP_FAT	GO:0044265 cellular macromolecule catabolic process	12	0.710900	0.010028	174	225	7937	2.432797	0.999985	0.347909	14.890381
GOTERM_BP_FAT	GO:0051726 regulation of cell cycle	10	0.592417	0.010152	174	166	7937	2.747888	0.999987	0.340873	15.060329
GOTERM_BP_FAT	GO:0019226 transmission of nerve impulse	11	0.651659	0.010451	174	196	7937	2.560022	0.999991	0.338904	15.469933
GOTERM_BP_FAT	GO:0010324 membrane invagination	13	0.770142	0.010614	174	258	7937	2.298427	0.999992	0.333594	15.692612
GOTERM_BP_FAT	GO:0006897 endocytosis	13	0.770142	0.010614	174	258	7937	2.298427	0.999992	0.333594	15.692612
GOTERM_BP_FAT	GO:0016310 phosphorylation	18	1.066351	0.011091	174	425	7937	1.931927	0.999995	0.336389	16.340525
SP_PIR_KEYWORDS	nucleus	30	1.777251	0.011603	279	869	12980	1.606098	0.871794	0.336896	13.343610
GOTERM_MF_FAT	GO:0016790 thiolester hydrolase activity	5	0.296209	0.011784	178	40	7918	5.560393	0.987840	0.987840	15.090947
GOTERM_CC_FAT	GO:0005905 coated pit	3	0.177725	0.012643	124	7	4786	16.541475	0.971272	0.397777	15.476755
GOTERM_BP_FAT	GO:0016081 synaptic vesicle docking during exocytosis	4	0.236967	0.013081	174	23	7937	7.933033	1.000000	0.374057	18.993106
GOTERM_BP_FAT	GO:0006323 DNA packaging	6	0.355450	0.013376	174	65	7937	4.210610	1.000000	0.371331	19.379231
GOTERM_BP_FAT	GO:0006886 intracellular protein transport	10	0.592417	0.013672	174	174	7937	2.621548	1.000000	0.368795	19.765673
GOTERM_MF_FAT	GO:0032183 SUMO binding	6	0.355450	0.014785	178	65	7918	4.106137	0.996078	0.937371	18.581307
SP_PIR_KEYWORDS	nuclear pore complex	3	0.177725	0.014898	279	9	12980	15.507766	0.928767	0.356156	16.822527
GOTERM_BP_FAT	GO:0006887 exocytosis	6	0.355450	0.015107	174	67	7937	4.084920	1.000000	0.389721	21.613418
GOTERM_CC_FAT	GO:0031090 organelle membrane	20	1.184834	0.015361	124	438	4786	1.762410	0.986687	0.417178	18.500439
GOTERM_MF_FAT	GO:0032182 small conjugating protein binding	6	0.355450	0.015716	178	66	7918	4.043922	0.997240	0.859732	19.636280
SP_PIR_KEYWORDS	kinase	11	0.651659	0.015858	279	212	12980	2.413945	0.939993	0.330951	17.811167

GOTERM_BP_FAT	GO:0015931 nucleobase, nucleoside, nucleotide and nucleic acid transport	6	0.355450	0.016027	174	68	7937	4.024848	1.000000	0.399006	22.776149
GOTERM_BP_FAT	GO:0034613 cellular protein localization	10	0.592417	0.016202	174	179	7937	2.548321	1.000000	0.393758	22.995514
GOTERM_CC_FAT	GO:0000145 exocyst	3	0.177725	0.016576	124	8	4786	14.473790	0.990566	0.404386	19.819385
GOTERM_BP_FAT	GO:0007267 cell-cell signaling	11	0.651659	0.016861	174	211	7937	2.378030	1.000000	0.397647	23.816028
GOTERM_CC_FAT	GO:0030136 clathrin-coated vesicle	6	0.355450	0.017108	124	59	4786	3.925096	0.991887	0.382102	20.390257
GOTERM_BP_FAT	GO:0070727 cellular macromolecule localization	12	0.710900	0.017523	174	244	7937	2.243358	1.000000	0.401379	24.632640
INTERPRO	IPR008942:ENTH/VHS	3	0.177725	0.017531	239	9	10196	14.220363	0.999779	0.999779	22.347355
GOTERM_MF_FAT	GO:0004722 protein serine/threonine phosphatase activity	5	0.296209	0.017631	178	45	7918	4.942572	0.998663	0.808771	21.767624
GOTERM_BP_FAT	GO:0017038 protein import	6	0.355450	0.017977	174	70	7937	3.909852	1.000000	0.401329	25.187933
GOTERM_BP_FAT	GO:0009891 positive regulation of biosynthetic process	9	0.533175	0.018497	174	153	7937	2.683232	1.000000	0.402399	25.819518
GOTERM_BP_FAT	GO:0031328 positive regulation of cellular biosynthetic process	9	0.533175	0.018497	174	153	7937	2.683232	1.000000	0.402399	25.819518
GOTERM_BP_FAT	GO:0065003 macromolecular complex assembly	12	0.710900	0.018505	174	246	7937	2.225119	1.000000	0.394983	25.829419
GOTERM_BP_FAT	GO:0043933 macromolecular complex subunit organization	13	0.770142	0.019668	174	281	7937	2.110300	1.000000	0.406477	27.223258
GOTERM_BP_FAT	GO:0019220 regulation of phosphate metabolic process	6	0.355450	0.020077	174	72	7937	3.801245	1.000000	0.405625	27.707153
GOTERM_BP_FAT	GO:0051174 regulation of phosphorus metabolic process	6	0.355450	0.020077	174	72	7937	3.801245	1.000000	0.405625	27.707153
SP_PIR_KEYWORDS	protein transport	7	0.414692	0.021336	279	101	12980	3.224387	0.977533	0.377781	23.252388
GOTERM_BP_FAT	GO:0051053 negative regulation of DNA metabolic process	3	0.177725	0.022830	174	11	7937	12.440439	1.000000	0.439515	30.888841
GOTERM_BP_FAT	GO:0016080 synaptic vesicle targeting	3	0.177725	0.022830	174	11	7937	12.440439	1.000000	0.439515	30.888841
GOTERM_BP_FAT	GO:0051656 establishment of organelle localization	6	0.355450	0.023520	174	75	7937	3.649195	1.000000	0.441990	31.664378
GOTERM_MF_FAT	GO:0003729 mRNA binding	10	0.592417	0.023581	178	186	7918	2.391567	0.999860	0.830586	28.059199
PIR_SUPERFAMILY	PIRSF000908: serine/ threonine-protein phosphatase, conventional type	3	0.177725	0.024077	73	9	2596	11.853881	0.888466	0.888466	23.289430
GOTERM_BP_FAT	GO:0048278 vesicle docking	4	0.236967	0.026846	174	30	7937	6.081992	1.000000	0.479266	35.294596
GOTERM_BP_FAT	GO:0022406 membrane docking	4	0.236967	0.026846	174	30	7937	6.081992	1.000000	0.479266	35.294596
GOTERM_BP_FAT	GO:0006904 vesicle docking during exocytosis	4	0.236967	0.026846	174	30	7937	6.081992	1.000000	0.479266	35.294596
SP_PIR_KEYWORDS	rna-binding	8	0.473934	0.028985	279	138	12980	2.697003	0.994354	0.437410	30.298243
GOTERM_BP_FAT	GO:0051650 establishment of vesicle localization	3	0.177725	0.031469	174	13	7937	10.526525	1.000000	0.527820	40.040989
GOTERM_BP_FAT	GO:0051648 vesicle localization	3	0.177725	0.031469	174	13	7937	10.526525	1.000000	0.527820	40.040989
GOTERM_BP_FAT	GO:0006903 vesicle targeting	3	0.177725	0.031469	174	13	7937	10.526525	1.000000	0.527820	40.040989
GOTERM_BP_FAT	GO:0006605 protein targeting	7	0.414692	0.031519	174	109	7937	2.929400	1.000000	0.520943	40.089954
GOTERM_BP_FAT	GO:0006325 chromatin organization	9	0.533175	0.031747	174	170	7937	2.414909	1.000000	0.516269	40.315544
GOTERM_BP_FAT	GO:0006470 protein amino acid dephosphorylation	6	0.355450	0.032972	174	82	7937	3.337679	1.000000	0.522703	41.511579
KEGG_PATHWAY	dme04150:mTOR signaling pathway	4	0.236967	0.033738	56	27	2054	5.433862	0.786565	0.786565	27.585338
GOTERM_BP_FAT	GO:0016050 vesicle organization	4	0.236967	0.034415	174	33	7937	5.529084	1.000000	0.531121	42.892235
GOTERM_BP_FAT	GO:0016568 chromatin modification	7	0.414692	0.035330	174	112	7937	2.850934	1.000000	0.533716	43.752087
SP_PIR_KEYWORDS	protein phosphatase	4	0.236967	0.035682	279	34	12980	5.473329	0.998330	0.472434	35.972990
GOTERM_MF_FAT	GO:0030554 adenyl nucleotide binding	27	1.599526	0.037633	178	812	7918	1.479119	0.999999	0.907288	41.103093
GOTERM_MF_FAT	GO:0000166 nucleotide binding	37	2.191943	0.039062	178	1206	7918	1.364740	1.000000	0.879670	42.299185
GOTERM_BP_FAT	GO:0009057 macromolecule catabolic process	12	0.710900	0.039470	174	277	7937	1.976099	1.000000	0.567460	47.492203
GOTERM_MF_FAT	GO:0001883 purine nucleoside binding	27	1.599526	0.040127	178	817	7918	1.470067	1.000000	0.851085	43.175249
SP_PIR_KEYWORDS	thiol protease	4	0.236967	0.041278	279	36	12980	5.169255	0.999400	0.490570	40.386071
SMART	SM00438:ZnF_NFX	2	0.118483	0.043063	106	2	4824	45.509434	0.990589	0.990589	38.977256
GOTERM_BP_FAT	GO:0006979 response to oxidative stress	5	0.296209	0.043537	174	61	7937	3.738930	1.000000	0.597166	50.938088
GOTERM_MF_FAT	GO:0001882 nucleoside binding	27	1.599526	0.043826	178	824	7918	1.457579	1.000000	0.843132	46.123548
GOTERM_BP_FAT	GO:0051276 chromosome organization	12	0.710900	0.043979	174	282	7937	1.941061	1.000000	0.594228	51.299328
GOTERM_CC_FAT	GO:0005768 endosome	4	0.236967	0.044138	124	31	4786	4.980229	0.999997	0.681766	44.929467
GOTERM_MF_FAT	GO:0005524 ATP binding	25	1.481043	0.044189	178	748	7918	1.486736	1.000000	0.813861	46.405544
GOTERM_CC_FAT	GO:0030135 coated vesicle	6	0.355450	0.044857	124	76	4786	3.047114	0.999997	0.655973	45.473921
GOTERM_MF_FAT	GO:0032559 adenyl ribonucleotide binding	25	1.481043	0.045352	178	750	7918	1.482772	1.000000	0.791868	47.298371
GOTERM_BP_FAT	GO:0016044 membrane organization	13	0.770142	0.045759	174	319	7937	1.858916	1.000000	0.602498	52.729411
INTERPRO	IPR005839:Uncharacterised protein family UPF0004	2	0.118483	0.046142	239	2	10196	42.661088	1.000000	0.999987	49.113939
INTERPRO	IPR013848:Uncharacterised protein family UPF0004, N-terminal	2	0.118483	0.046142	239	2	10196	42.661088	1.000000	0.999987	49.113939
INTERPRO	IPR002792: Deoxyribonuclease/ rho motif-related TRAM	2	0.118483	0.046142	239	2	10196	42.661088	1.000000	0.999987	49.113939
INTERPRO	IPR000967:Zinc finger, NF-X1-type	2	0.118483	0.046142	239	2	10196	42.661088	1.000000	0.999987	49.113939
SP_PIR_KEYWORDS	atp-binding	20	1.184834	0.046373	279	585	12980	1.590540	0.999765	0.501634	44.159877
GOTERM_BP_FAT	GO:0070142 synaptic vesicle budding	3	0.177725	0.046400	174	16	7937	8.552802	1.000000	0.601237	53.235400

GOTERM_BP_FAT	GO:0016185 synaptic vesicle budding from presynaptic membrane	3	0.177725	0.046400	174	16	7937	8.552802	1.000000	0.601237	53.235400
GOTERM_BP_FAT	GO:0044257 cellular protein catabolic process	9	0.533175	0.046553	174	183	7937	2.243358	1.000000	0.596094	53.354679
GOTERM_BP_FAT	GO:0051603 proteolysis involved in cellular protein catabolic process	9	0.533175	0.046553	174	183	7937	2.243358	1.000000	0.596094	53.354679
SP_PIR_KEYWORDS	dna replication	4	0.236967	0.047288	279	38	12980	4.897189	0.999802	0.480991	44.813228
GOTERM_BP_FAT	GO:0050658 RNA transport	5	0.296209	0.048084	174	63	7937	3.620234	1.000000	0.601978	54.538484
GOTERM_BP_FAT	GO:0050657 nucleic acid transport	5	0.296209	0.048084	174	63	7937	3.620234	1.000000	0.601978	54.538484
SP_PIR_KEYWORDS	chromatin regulator	5	0.296209	0.048119	279	64	12980	3.634633	0.999830	0.462035	45.401165
GOTERM_BP_FAT	GO:0033365 protein localization in organelle	6	0.355450	0.048187	174	91	7937	3.007579	1.000000	0.596628	54.617637
SP_PIR_KEYWORDS	nucleotide-binding	24	1.421801	0.048428	279	743	12980	1.502771	0.999839	0.441470	45.618628
GOTERM_MF_FAT	GO:0017076 purine nucleotide binding	31	1.836493	0.048870	178	989	7918	1.394315	1.000000	0.788438	49.916354
GOTERM_CC_FAT	GO:0008021 synaptic vesicle	5	0.296209	0.049013	124	54	4786	3.573775	0.999999	0.659912	48.527592
GOTERM_BP_FAT	GO:0009451 RNA modification	4	0.236967	0.049207	174	38	7937	4.801573	1.000000	0.598439	55.389228
GOTERM_BP_FAT	GO:0051236 establishment of RNA localization	5	0.296209	0.050452	174	64	7937	3.563667	1.000000	0.601873	56.314311
GOTERM_CC_FAT	GO:0005943 1-phosphatidylinositol-4-phosphate 3-kinase, class IA complex	2	0.118483	0.050745	124	2	4786	38.596774	1.000000	0.645777	49.752359
GOTERM_BP_FAT	GO:0000059 protein import into nucleus, docking	3	0.177725	0.051850	174	17	7937	8.049696	1.000000	0.606303	57.332410
GOTERM_CC_FAT	GO:0031410 cytoplasmic vesicle	7	0.414692	0.052191	124	105	4786	2.573118	1.000000	0.631015	50.754643
GOTERM_BP_FAT	GO:0042325 regulation of phosphorylation	5	0.296209	0.052883	174	65	7937	3.508842	1.000000	0.607958	58.069678
GOTERM_MF_FAT	GO:0032555 purine ribonucleotide binding	29	1.718009	0.056278	178	923	7918	1.397629	1.000000	0.809388	55.039636
GOTERM_MF_FAT	GO:0032553 ribonucleotide binding	29	1.718009	0.056278	178	923	7918	1.397629	1.000000	0.809388	55.039636
GOTERM_BP_FAT	GO:0006468 protein amino acid phosphorylation	11	0.651659	0.056471	174	259	7937	1.937314	1.000000	0.627082	60.540110
GOTERM_MF_FAT	GO:0008234 cysteine-type peptidase activity	5	0.296209	0.057029	178	65	7918	3.421780	1.000000	0.789917	55.530471
SMART	SM00156:PP2Ac	3	0.177725	0.057252	106	18	4824	7.584906	0.998068	0.956049	48.394983
GOTERM_BP_FAT	GO:0002786 regulation of antibacterial peptide production	3	0.177725	0.057516	174	18	7937	7.602490	1.000000	0.628409	61.233160
GOTERM_BP_FAT	GO:0051052 regulation of DNA metabolic process	3	0.177725	0.057516	174	18	7937	7.602490	1.000000	0.628409	61.233160
GOTERM_BP_FAT	GO:0006900 membrane budding	3	0.177725	0.057516	174	18	7937	7.602490	1.000000	0.628409	61.233160
GOTERM_BP_FAT	GO:0002808 regulation of antibacterial peptide biosynthetic process	3	0.177725	0.057516	174	18	7937	7.602490	1.000000	0.628409	61.233160
GOTERM_BP_FAT	GO:0006963 positive regulation of antibacterial peptide biosynthetic process	3	0.177725	0.057516	174	18	7937	7.602490	1.000000	0.628409	61.233160
GOTERM_BP_FAT	GO:0006260 DNA replication	6	0.355450	0.058173	174	96	7937	2.850934	1.000000	0.627181	61.663083
SP_PIR_KEYWORDS	ubl conjugation pathway	6	0.355450	0.059267	279	98	12980	2.848365	0.999979	0.489343	52.750353
GOTERM_CC_FAT	GO:0044429 mitochondrial part	17	1.007109	0.059807	124	411	4786	1.596460	1.000000	0.658830	55.734968
GOTERM_BP_FAT	GO:0035222 wing disc pattern formation	5	0.296209	0.060553	174	68	7937	3.354040	1.000000	0.636945	63.184041
GOTERM_BP_FAT	GO:0060341 regulation of cellular localization	5	0.296209	0.060553	174	68	7937	3.354040	1.000000	0.636945	63.184041
GOTERM_CC_FAT	GO:0008023 transcription elongation factor complex	3	0.177725	0.062168	124	16	4786	7.236895	1.000000	0.651243	57.181546
GOTERM_BP_FAT	GO:0007346 regulation of mitotic cell cycle	6	0.355450	0.062475	174	98	7937	2.792752	1.000000	0.643441	64.370736
GOTERM_MF_FAT	GO:0004721 phosphoprotein phosphatase activity	6	0.355450	0.063452	178	96	7918	2.780197	1.000000	0.803235	59.533383
SMART	SM00288:VHS	2	0.118483	0.063900	106	3	4824	30.339623	0.999088	0.903013	52.334532
UP_SEQ_FEATURE	domain:TRAM	2	0.118483	0.064808	99	2	2975	30.050505	1.000000	1.000000	59.597948
UP_SEQ_FEATURE	domain:MTTase N-terminal	2	0.118483	0.064808	99	2	2975	30.050505	1.000000	1.000000	59.597948
INTERPRO	IPR006186:Serine/threonine-specific protein phosphatase and bis(5-nucleosyl)-tetraphosphatase	3	0.177725	0.064966	239	18	10196	7.110181	1.000000	0.999976	61.734163
GOTERM_CC_FAT	GO:0031982 vesicle	7	0.414692	0.064986	124	111	4786	2.434031	1.000000	0.647078	58.851163
GOTERM_CC_FAT	GO:0005740 mitochondrial envelope	12	0.710900	0.066268	124	258	4786	1.795199	1.000000	0.634625	59.590243
INTERPRO	IPR018205:VHS subgroup	2	0.118483	0.068412	239	3	10196	28.440725	1.000000	0.999782	63.702526
GOTERM_BP_FAT	GO:0030163 protein catabolic process	9	0.533175	0.068987	174	200	7937	2.052672	1.000000	0.675785	68.129851
GOTERM_BP_FAT	GO:0045793 positive regulation of cell size	3	0.177725	0.069447	174	20	7937	6.842241	1.000000	0.673119	68.380669
GOTERM_CC_FAT	GO:0015629 actin cytoskeleton	6	0.355450	0.072069	124	87	4786	2.661846	1.000000	0.647754	62.784778
GOTERM_MF_FAT	GO:0000287 magnesium ion binding	6	0.355450	0.072908	178	100	7918	2.668989	1.000000	0.827967	64.821665
GOTERM_BP_FAT	GO:0019941 modification-dependent protein catabolic process	8	0.473934	0.074580	174	169	7937	2.159287	1.000000	0.694978	71.058481
GOTERM_CC_FAT	GO:0032982 myosin filament	2	0.118483	0.075151	124	3	4786	25.731183	1.000000	0.645816	64.385566
GOTERM_CC_FAT	GO:0031933 telomeric heterochromatin	2	0.118483	0.075151	124	3	4786	25.731183	1.000000	0.645816	64.385566
GOTERM_CC_FAT	GO:0005863 striated muscle thick filament	2	0.118483	0.075151	124	3	4786	25.731183	1.000000	0.645816	64.385566
GOTERM_BP_FAT	GO:0006406 mRNA export from nucleus	3	0.177725	0.075689	174	21	7937	6.516420	1.000000	0.695544	71.608521

GOTERM_BP_FAT	GO:0043632 modification-dependent macromolecule catabolic process	8	0.473934	0.076651	174	170	7937	2.146586	1.000000	0.695374	72.077275
GOTERM_BP_FAT	GO:0045333 cellular respiration	6	0.355450	0.078911	174	105	7937	2.606568	1.000000	0.701466	73.150968
SP_PIR_KEYWORDS	coated pit	2	0.118483	0.082966	279	4	12980	23.261649	1.000000	0.592078	65.452198
SP_PIR_KEYWORDS	gastrulation	2	0.118483	0.082966	279	4	12980	23.261649	1.000000	0.592078	65.452198
GOTERM_BP_FAT	GO:0046146 tetrahydrobiopterin metabolic process	2	0.118483	0.084393	174	4	7937	22.807471	1.000000	0.721849	75.596040
GOTERM_BP_FAT	GO:0006729 tetrahydrobiopterin biosynthetic process	2	0.118483	0.084393	174	4	7937	22.807471	1.000000	0.721849	75.596040
GOTERM_BP_FAT	GO:0030241 muscle thick filament assembly	2	0.118483	0.084393	174	4	7937	22.807471	1.000000	0.721849	75.596040
UP_SEQ_FEATURE	metal ion-binding site:Iron	3	0.177725	0.085388	99	15	2975	6.010101	1.000000	0.999999	70.098802
GOTERM_BP_FAT	GO:0006915 apoptosis	5	0.296209	0.086865	174	77	7937	2.962009	1.000000	0.727932	76.629067
GOTERM_CC_FAT	GO:0005700 polytene chromosome	6	0.355450	0.086879	124	92	4786	2.517181	1.000000	0.684191	69.913166
SP_PIR_KEYWORDS	sh3 domain	4	0.236967	0.087266	279	49	12980	3.797820	1.000000	0.590497	67.388044
SP_PIR_KEYWORDS	metal-binding	24	1.421801	0.088312	279	796	12980	1.402712	1.000000	0.575332	67.843867
GOTERM_BP_FAT	GO:0006405 RNA export from nucleus	3	0.177725	0.088676	174	23	7937	5.949775	1.000000	0.731012	77.359662
GOTERM_BP_FAT	GO:0007163 establishment or maintenance of cell polarity	6	0.355450	0.089250	174	109	7937	2.510914	1.000000	0.728900	77.586747
GOTERM_BP_FAT	GO:0016311 dephosphorylation	6	0.355450	0.089250	174	109	7937	2.510914	1.000000	0.728900	77.586747
GOTERM_CC_FAT	GO:0031966 mitochondrial membrane	11	0.651659	0.089664	124	241	4786	1.761678	1.000000	0.680038	71.103377
INTERPRO	IPR002014:VHS	2	0.118483	0.090164	239	4	10196	21.330544	1.000000	0.999876	74.109561
SP_PIR_KEYWORDS	coiled coil	9	0.533175	0.091488	279	215	12980	1.947487	1.000000	0.570157	69.191873
GOTERM_CC_FAT	GO:0000792 heterochromatin	3	0.177725	0.092160	124	20	4786	5.789516	1.000000	0.675020	72.133131
UP_SEQ_FEATURE	active site:Proton donor	4	0.236967	0.092854	99	33	2975	3.642485	1.000000	0.999974	73.236526
GOTERM_BP_FAT	GO:0002807 positive regulation of antimicrobial peptide biosynthetic process	3	0.177725	0.095400	174	24	7937	5.701868	1.000000	0.749016	79.888971
GOTERM_BP_FAT	GO:0002784 regulation of antimicrobial peptide production	3	0.177725	0.095400	174	24	7937	5.701868	1.000000	0.749016	79.888971
GOTERM_BP_FAT	GO:0016197 endosome transport	3	0.177725	0.095400	174	24	7937	5.701868	1.000000	0.749016	79.888971
GOTERM_BP_FAT	GO:0002700 regulation of production of molecular mediator of immune response	3	0.177725	0.095400	174	24	7937	5.701868	1.000000	0.749016	79.888971
GOTERM_BP_FAT	GO:0002805 regulation of antimicrobial peptide biosynthetic process	3	0.177725	0.095400	174	24	7937	5.701868	1.000000	0.749016	79.888971
GOTERM_BP_FAT	GO:0002697 regulation of immune effector process	3	0.177725	0.095400	174	24	7937	5.701868	1.000000	0.749016	79.888971
UP_SEQ_FEATURE	metal ion-binding site:Manganese	3	0.177725	0.095556	99	16	2975	5.634470	1.000000	0.999714	74.295137
UP_SEQ_FEATURE	metal ion-binding site:Iron-sulfur (4Fe-4S-S-AdoMet)	2	0.118483	0.095635	99	3	2975	20.033670	1.000000	0.998547	74.325372
SP_PIR_KEYWORDS	transit peptide	6	0.355450	0.098080	279	114	12980	2.448595	1.000000	0.579017	71.825618
GOTERM_CC_FAT	GO:0005942 phosphoinositide 3-kinase complex	2	0.118483	0.098934	124	4	4786	19.298387	1.000000	0.687333	74.759105
GOTERM_BP_FAT	GO:0051338 regulation of transferase activity	4	0.236967	0.099331	174	51	7937	3.577643	1.000000	0.759398	81.242576
GOTERM_BP_FAT	GO:0043549 regulation of kinase activity	4	0.236967	0.099331	174	51	7937	3.577643	1.000000	0.759398	81.242576
GOTERM_BP_FAT	GO:0045859 regulation of protein kinase activity	4	0.236967	0.099331	174	51	7937	3.577643	1.000000	0.759398	81.242576

Table 25: Architecture 25

Category	Term	Count	%	PValue	List Total	Pop Hits	Pop Total	Fold Enrichment	Bonferroni	Benjamini	FDR
GOTERM_CC_FAT	GO:0031974 membrane-enclosed lumen	85	1.810822	3.548092e-11	350	571	4786	2.035577	1.355372e-08	1.355372e-08	4.915734e-08
GOTERM_CC_FAT	GO:0070013 intracellular organelle lumen	82	1.746911	1.476813e-10	350	556	4786	2.016711	5.641426e-08	2.820713e-08	2.046061e-07
GOTERM_CC_FAT	GO:0043233 organelle lumen	82	1.746911	1.476813e-10	350	556	4786	2.016711	5.641426e-08	2.820713e-08	2.046061e-07
GOTERM_BP_FAT	GO:0006396 RNA processing	50	1.065190	3.839030e-09	514	312	7937	2.474621	6.430355e-06	6.430355e-06	6.463808e-06
SP_PIR_KEYWORDS	atp-binding	67	1.427354	1.416262e-06	806	585	12980	1.844415	3.171927e-04	3.171927e-04	0.001808
GOTERM_BP_FAT	GO:0046907 intracellular transport	44	0.937367	1.660678e-06	514	313	7937	2.170709	0.002778	0.001390	0.002796
GOTERM_CC_FAT	GO:0005654 nucleoplasm	42	0.894759	2.071509e-06	350	265	4786	2.167245	7.910043e-04	2.637376e-04	0.002870
GOTERM_CC_FAT	GO:0044451 nucleoplasm part	39	0.830848	2.854126e-06	350	240	4786	2.222071	0.001090	2.725323e-04	0.003954
GOTERM_CC_FAT	GO:0031981 nuclear lumen	54	1.150405	2.945103e-06	350	387	4786	1.908040	0.001124	2.249809e-04	0.004080
GOTERM_CC_FAT	GO:0005739 mitochondrion	71	1.512569	4.057092e-06	350	571	4786	1.700305	0.001549	2.582687e-04	0.005621
SP_PIR_KEYWORDS	phosphoprotein	84	1.789519	4.062942e-06	806	815	12980	1.659821	9.096869e-04	4.549469e-04	0.005187
GOTERM_MF_FAT	GO:0000166 nucleotide binding	120	2.556455	4.416212e-06	534	1206	7918	1.475395	0.002774	0.002774	0.006564
GOTERM_MF_FAT	GO:0005524 ATP binding	82	1.746911	7.289815e-06	534	748	7918	1.625498	0.004575	0.002290	0.010835
GOTERM_MF_FAT	GO:0032559 adenyl ribonucleotide binding	82	1.746911	8.048172e-06	534	750	7918	1.621164	0.005050	0.001686	0.011963
GOTERM_CC_FAT	GO:0044429 mitochondrial part	55	1.171709	8.424064e-06	350	411	4786	1.829892	0.003213	4.596095e-04	0.011671
GOTERM_MF_FAT	GO:0001882 nucleoside binding	88	1.874734	8.880391e-06	534	824	7918	1.583542	0.005570	0.001395	0.013200
GOTERM_MF_FAT	GO:0001883 purine nucleoside binding	87	1.853430	1.129198e-05	534	817	7918	1.578961	0.007078	0.001420	0.016784
GOTERM_BP_FAT	GO:0006351 transcription, DNA-dependent	24	0.511291	1.191420e-05	514	133	7937	2.786460	0.019759	0.006630	0.020058
GOTERM_BP_FAT	GO:0034660 ncRNA metabolic process	24	0.511291	1.191420e-05	514	133	7937	2.786460	0.019759	0.006630	0.020058
GOTERM_BP_FAT	GO:0006259 DNA metabolic process	33	0.703025	1.242781e-05	514	221	7937	2.305764	0.020602	0.005191	0.020923
GOTERM_CC_FAT	GO:0012505 endomembrane system	35	0.745633	1.378502e-05	350	218	4786	2.195413	0.005252	6.580229e-04	0.019097
GOTERM_CC_FAT	GO:0005761 mitochondrial ribosome	18	0.383468	1.544154e-05	350	74	4786	3.326178	0.005881	6.551980e-04	0.021391
GOTERM_CC_FAT	GO:0000313 organellar ribosome	18	0.383468	1.544154e-05	350	74	4786	3.326178	0.005881	6.551980e-04	0.021391
GOTERM_MF_FAT	GO:0030554 adenyl nucleotide binding	86	1.832126	1.592387e-05	534	812	7918	1.570423	0.009966	0.001668	0.023668
GOTERM_BP_FAT	GO:0032774 RNA biosynthetic process	24	0.511291	1.737785e-05	514	136	7937	2.724994	0.028689	0.005805	0.029255
GOTERM_CC_FAT	GO:0031980 mitochondrial lumen	28	0.596506	1.863587e-05	350	157	4786	2.438726	0.007094	7.116435e-04	0.025816
GOTERM_CC_FAT	GO:0005759 mitochondrial matrix	28	0.596506	1.863587e-05	350	157	4786	2.438726	0.007094	7.116435e-04	0.025816
GOTERM_BP_FAT	GO:0016071 mRNA metabolic process	32	0.681721	2.682084e-05	514	219	7937	2.256312	0.043931	0.007460	0.045149
GOTERM_MF_FAT	GO:0032555 purine ribonucleotide binding	94	2.002556	2.749288e-05	534	923	7918	1.510082	0.017145	0.002467	0.040859
GOTERM_MF_FAT	GO:0032553 ribonucleotide binding	94	2.002556	2.749288e-05	534	923	7918	1.510082	0.017145	0.002467	0.040859
GOTERM_CC_FAT	GO:0016591 DNA-directed RNA polymerase II, holoenzyme	15	0.319557	3.201081e-05	350	56	4786	3.662755	0.012154	0.001111	0.044340
GOTERM_MF_FAT	GO:0003729 mRNA binding	29	0.617810	4.394393e-05	534	186	7918	2.311848	0.027263	0.003449	0.065301
GOTERM_BP_FAT	GO:0006397 mRNA processing	29	0.617810	4.611164e-05	514	194	7937	2.308286	0.074331	0.010973	0.077610
GOTERM_BP_FAT	GO:0006366 transcription from RNA polymerase II promoter	19	0.404772	5.328383e-05	514	99	7937	2.963546	0.085386	0.011095	0.089677
GOTERM_MF_FAT	GO:0017076 purine nucleotide binding	98	2.087772	5.351704e-05	534	989	7918	1.469278	0.033103	0.003733	0.079522
GOTERM_MF_FAT	GO:0016251 general RNA polymerase II transcription factor activity	18	0.383468	7.432890e-05	534	89	7918	2.998864	0.045678	0.004665	0.110430
GOTERM_CC_FAT	GO:0030529 ribonucleoprotein complex	49	1.043886	7.480933e-05	350	379	4786	1.767916	0.028174	0.002379	0.103595
SP_PIR_KEYWORDS	nucleotide-binding	73	1.555177	9.228431e-05	806	743	12980	1.582245	0.020460	0.006867	0.117749
GOTERM_BP_FAT	GO:0043933 macromolecular complex subunit organization	36	0.766937	1.241447e-04	514	281	7937	1.978288	0.187757	0.022841	0.208818
SP_PIR_KEYWORDS	cytoplasm	48	1.022582	1.451397e-04	806	436	12980	1.772941	0.031991	0.008095	0.185132
GOTERM_CC_FAT	GO:0017053 transcriptional repressor complex	6	0.127823	1.976544e-04	350	9	4786	9.116190	0.072731	0.005792	0.273494
GOTERM_CC_FAT	GO:0031967 organelle envelope	44	0.937367	2.508660e-04	350	344	4786	1.749037	0.091393	0.006823	0.347004
GOTERM_CC_FAT	GO:0031975 envelope	44	0.937367	2.624492e-04	350	345	4786	1.743967	0.095406	0.006662	0.362999
SMART	SM00490:HELICc	15	0.319557	2.668254e-04	310	76	4824	3.071307	0.050200	0.050200	0.331978
GOTERM_MF_FAT	GO:0008312 7S RNA binding	5	0.106519	2.730783e-04	534	6	7918	12.356429	0.157844	0.015496	0.405153
GOTERM_BP_FAT	GO:0006886 intracellular protein transport	25	0.532595	3.212913e-04	514	174	7937	2.218626	0.416231	0.052402	0.539587
SP_PIR_KEYWORDS	nucleus	80	1.704303	3.364595e-04	806	869	12980	1.482552	0.072609	0.014963	0.428685
GOTERM_BP_FAT	GO:0006612 protein targeting to membrane	9	0.191734	3.683998e-04	514	29	7937	4.792231	0.460538	0.054563	0.618472
SP_PIR_KEYWORDS	ligase	26	0.553899	3.705367e-04	806	193	12980	2.169480	0.079663	0.013741	0.472008
GOTERM_CC_FAT	GO:0048500 signal recognition particle	6	0.127823	3.717657e-04	350	10	4786	8.204571	0.132414	0.008838	0.513837

GOTERM_CC_FAT	GO:0005786 signal recognition particle, endoplasmic reticulum targeting	6	0.127823	3.717657e-04	350	10	4786	8.204571	0.132414	0.008838	0.513837
GOTERM_CC_FAT	GO:0031090 organelle membrane	52	1.107797	3.795923e-04	350	438	4786	1.623431	0.135005	0.008495	0.524628
GOTERM_BP_FAT	GO:0000226 microtubule cytoskeleton organization	36	0.766937	3.930782e-04	514	298	7937	1.865432	0.482391	0.053399	0.659773
GOTERM_BP_FAT	GO:0000278 mitotic cell cycle	41	0.873455	4.349106e-04	514	358	7937	1.768455	0.517432	0.054507	0.729746
GOTERM_BP_FAT	GO:0070727 cellular macromolecule localization	31	0.660418	4.685780e-04	514	244	7937	1.961847	0.543904	0.054532	0.786028
GOTERM_BP_FAT	GO:0015031 protein transport	34	0.724329	4.739438e-04	514	278	7937	1.888545	0.547987	0.051560	0.794995
GOTERM_CC_FAT	GO:0005681 spliceosome	15	0.319557	4.852083e-04	350	71	4786	2.888934	0.169224	0.010247	0.670143
GOTERM_BP_FAT	GO:0034613 cellular protein localization	25	0.532595	4.912097e-04	514	179	7937	2.156653	0.560878	0.050136	0.823845
GOTERM_BP_FAT	GO:0006613 cotranslational protein targeting to membrane	7	0.149127	6.725629e-04	514	18	7937	6.005080	0.675971	0.064140	1.126389
GOTERM_BP_FAT	GO:0031023 microtubule organizing center organization	11	0.234342	6.857911e-04	514	47	7937	3.614000	0.683076	0.061844	1.148423
SP_PIR_KEYWORDS	ribosomal protein	22	0.468683	6.954831e-04	806	157	12980	2.256642	0.144306	0.022017	0.884251
GOTERM_BP_FAT	GO:0006352 transcription initiation	14	0.298253	7.055179e-04	514	73	7937	2.961409	0.693384	0.060323	1.181273
GOTERM_BP_FAT	GO:0045184 establishment of protein localization	34	0.724329	7.384291e-04	514	285	7937	1.842160	0.709841	0.059991	1.236056
GOTERM_BP_FAT	GO:0034470 ncRNA processing	16	0.340861	7.451618e-04	514	92	7937	2.685502	0.713097	0.057725	1.247259
INTERPRO	IPR001650:DNA/RNA helicase, C-terminal	15	0.319557	7.877660e-04	723	76	10196	2.783359	0.583040	0.583040	1.253769
GOTERM_BP_FAT	GO:0006367 transcription initiation from RNA polymerase II promoter	13	0.276949	8.043576e-04	514	65	7937	3.088327	0.740199	0.059427	1.345712
SMART	SM00487:DEXDc	14	0.298253	8.093765e-04	310	75	4824	2.904774	0.144674	0.075162	1.003885
SP_PIR_KEYWORDS	coiled coil	27	0.575202	8.248880e-04	806	215	12980	2.022390	0.168772	0.022841	1.047981
SP_PIR_KEYWORDS	helicase	14	0.298253	8.312223e-04	806	77	12980	2.928040	0.169952	0.020484	1.055989
GOTERM_MF_FAT	GO:0003712 transcription cofactor activity	13	0.276949	8.606172e-04	534	63	7918	3.059687	0.418161	0.044127	1.271672
SP_PIR_KEYWORDS	rna-binding	20	0.426076	8.619870e-04	806	138	12980	2.333945	0.175657	0.019131	1.094875
GOTERM_BP_FAT	GO:0022613 ribonucleoprotein complex biogenesis	16	0.340861	9.381479e-04	514	94	7937	2.628363	0.792399	0.066070	1.567888
GOTERM_BP_FAT	GO:0008380 RNA splicing	21	0.447380	0.001284	514	148	7937	2.191043	0.883744	0.085763	2.139923
INTERPRO	IPR015413:Aminoacyl-tRNA synthetase, class I (M)	4	0.085215	0.001340	723	4	10196	14.102351	0.774260	0.524879	2.123838
GOTERM_BP_FAT	GO:0051297 centrosome organization	10	0.213038	0.001429	514	43	7937	3.591078	0.908889	0.091379	2.379364
GOTERM_CC_FAT	GO:0005675 holo TFIIH complex	5	0.106519	0.001537	350	8	4786	8.546429	0.444331	0.030452	2.108531
GOTERM_CC_FAT	GO:0043232 intracellular non-membrane-bounded organelle	92	1.959949	0.001577	350	943	4786	1.334077	0.452771	0.029695	2.162850
GOTERM_CC_FAT	GO:0043228 non-membrane-bounded organelle	92	1.959949	0.001577	350	943	4786	1.334077	0.452771	0.029695	2.162850
GOTERM_BP_FAT	GO:0006605 protein targeting	17	0.362164	0.001609	514	109	7937	2.408328	0.932572	0.098522	2.674310
INTERPRO	IPR014021:Helicase, superfamily 1 and 2, ATP-binding	14	0.298253	0.001657	723	73	10196	2.704561	0.841315	0.458607	2.620141
GOTERM_CC_FAT	GO:0000315 organellar large ribosomal subunit	11	0.234342	0.001689	350	47	4786	3.200365	0.475733	0.030282	2.314841
GOTERM_CC_FAT	GO:0005762 mitochondrial large ribosomal subunit	11	0.234342	0.001689	350	47	4786	3.200365	0.475733	0.030282	2.314841
GOTERM_BP_FAT	GO:0007098 centrosome cycle	9	0.191734	0.001740	514	36	7937	3.860409	0.945907	0.102407	2.889645
UP_SEQ_FEATURE	compositionally biased region:Poly-Glu	11	0.234342	0.001909	233	45	2975	3.121125	0.676710	0.676710	2.776798
GOTERM_MF_FAT	GO:0008026 ATP-dependent helicase activity	14	0.298253	0.001934	534	78	7918	2.661385	0.704084	0.089415	2.836629
GOTERM_MF_FAT	GO:0070035 purine NTP-dependent helicase activity	14	0.298253	0.001934	534	78	7918	2.661385	0.704084	0.089415	2.836629
GOTERM_BP_FAT	GO:0033365 protein localization in organelle	15	0.319557	0.001969	514	91	7937	2.545324	0.963143	0.111200	3.263435
INTERPRO	IPR014001:DEAD-like helicase, N-terminal	14	0.298253	0.002134	723	75	10196	2.632439	0.906642	0.447239	3.362384
GOTERM_BP_FAT	GO:0007010 cytoskeleton organization	47	1.001278	0.002268	514	465	7937	1.560767	0.977694	0.122901	3.750549
GOTERM_BP_FAT	GO:0042254 ribosome biogenesis	12	0.255646	0.002399	514	64	7937	2.895306	0.982112	0.125516	3.963841
SP_PIR_KEYWORDS	mitochondrion	23	0.489987	0.002484	806	185	12980	2.002146	0.427119	0.049382	3.125045
GOTERM_BP_FAT	GO:0006399 tRNA metabolic process	15	0.319557	0.002688	514	94	7937	2.464091	0.988991	0.135371	4.431343
GOTERM_MF_FAT	GO:0008270 zinc ion binding	92	1.959949	0.002793	534	1026	7918	1.329581	0.827838	0.118090	4.072402
GOTERM_BP_FAT	GO:0006974 response to DNA damage stimulus	17	0.362164	0.002823	514	115	7937	2.282676	0.991220	0.137550	4.648411
GOTERM_BP_FAT	GO:0006350 transcription	46	0.979974	0.003023	514	459	7937	1.547528	0.993723	0.142438	4.969459
KEGG_PATHWAY	dme03040:Spliceosome	20	0.426076	0.003097	183	111	2054	2.022350	0.229404	0.229404	3.275109
KEGG_PATHWAY	dme03060:Protein export	5	0.106519	0.003143	183	8	2054	7.015027	0.232338	0.123837	3.322248

GOTERM_BP_FAT	GO:0000375 RNA splicing, via transesterification reactions	18	0.383468	0.003199	514	127	7937	2.188578	0.995334	0.146035	5.252371
GOTERM_BP_FAT	GO:0006260 DNA replication	15	0.319557	0.003279	514	96	7937	2.412755	0.995918	0.145448	5.379698
GOTERM_BP_FAT	GO:0007052 mitotic spindle organization	24	0.511291	0.003348	514	194	7937	1.910305	0.996367	0.144481	5.490428
GOTERM_CC_FAT	GO:0005635 nuclear envelope	15	0.319557	0.003361	350	86	4786	2.385050	0.723610	0.056776	4.556831
GOTERM_BP_FAT	GO:0006364 rRNA processing	9	0.191734	0.003521	514	40	7937	3.474368	0.997284	0.147597	5.766511
GOTERM_BP_FAT	GO:0032984 macromolecular complex disassembly	6	0.127823	0.003569	514	17	7937	5.449989	0.997495	0.145826	5.843071
GOTERM_BP_FAT	GO:0034623 cellular macromolecular complex disassembly	6	0.127823	0.003569	514	17	7937	5.449989	0.997495	0.145826	5.843071
GOTERM_BP_FAT	GO:0006614 SRP-dependent cotranslational protein targeting to membrane	6	0.127823	0.003569	514	17	7937	5.449989	0.997495	0.145826	5.843071
GOTERM_BP_FAT	GO:0045047 protein targeting to ER	6	0.127823	0.003569	514	17	7937	5.449989	0.997495	0.145826	5.843071
GOTERM_MF_FAT	GO:0032183 SUMO binding	12	0.255646	0.003729	534	65	7918	2.737424	0.904631	0.145011	5.402156
GOTERM_BP_FAT	GO:0016072 rRNA metabolic process	9	0.191734	0.004133	514	41	7937	3.389627	0.999030	0.162969	6.736338
GOTERM_MF_FAT	GO:0032182 small conjugating protein binding	12	0.255646	0.004211	534	66	7918	2.695948	0.929649	0.152861	6.079896
GOTERM_BP_FAT	GO:0033554 cellular response to stress	22	0.468683	0.004587	514	176	7937	1.930204	0.999548	0.175140	7.449601
GOTERM_BP_FAT	GO:0007017 microtubule-based process	41	0.873455	0.004656	514	406	7937	1.559377	0.999597	0.173590	7.557054
GOTERM_CC_FAT	GO:0005667 transcription factor complex	14	0.298253	0.004721	350	80	4786	2.393000	0.835956	0.075583	6.345683
GOTERM_CC_FAT	GO:0005763 mitochondrial small ribosomal subunit	8	0.170430	0.004848	350	30	4786	3.646476	0.843756	0.074432	6.511024
GOTERM_CC_FAT	GO:0000314 organellar small ribosomal subunit	8	0.170430	0.004848	350	30	4786	3.646476	0.843756	0.074432	6.511024
SP_PIR_KEYWORDS	transferase	74	1.576481	0.005052	806	873	12980	1.365077	0.678449	0.090218	6.261720
GOTERM_BP_FAT	GO:0007051 spindle organization	26	0.553899	0.005374	514	225	7937	1.784367	0.999880	0.193365	8.672697
GOTERM_BP_FAT	GO:0016568 chromatin modification	16	0.340861	0.005429	514	112	7937	2.205948	0.999890	0.191074	8.757999
GOTERM_MF_FAT	GO:0003713 transcription coactivator activity	8	0.170430	0.005533	534	33	7918	3.594598	0.969495	0.185586	7.916390
GOTERM_BP_FAT	GO:0006281 DNA repair	15	0.319557	0.005714	514	102	7937	2.270829	0.999932	0.195981	9.196941
INTERPRO	IPR008011:Complex 1 LYR protein	4	0.085215	0.006013	723	6	10196	9.401568	0.998763	0.737895	9.204857
GOTERM_BP_FAT	GO:0000398 nuclear mRNA splicing, via spliceosome	17	0.362164	0.006988	514	126	7937	2.083395	0.999992	0.229747	11.137247
GOTERM_BP_FAT	GO:0000377 RNA splicing, via transesterification reactions with bulged adenosine as nucleophile	17	0.362164	0.006988	514	126	7937	2.083395	0.999992	0.229747	11.137247
SP_PIR_KEYWORDS	ubl conjugation pathway	14	0.298253	0.007364	806	98	12980	2.300603	0.809023	0.119578	9.004193
GOTERM_BP_FAT	GO:0008104 protein localization	39	0.830848	0.007482	514	392	7937	1.536285	0.999997	0.239257	11.877776
GOTERM_CC_FAT	GO:0019866 organelle inner membrane	27	0.575202	0.007519	350	217	4786	1.701409	0.944030	0.108917	9.927947
SMART	SM00233:PH	12	0.255646	0.007614	310	75	4824	2.489806	0.771252	0.388421	9.084552
GOTERM_BP_FAT	GO:0022411 cellular component disassembly	7	0.149127	0.007860	514	28	7937	3.860409	0.999998	0.245130	12.440862
GOTERM_MF_FAT	GO:0031202 RNA splicing factor activity, transesterification mechanism	7	0.149127	0.007930	534	27	7918	3.844222	0.993316	0.242871	11.161465
GOTERM_BP_FAT	GO:0065003 macromolecular complex assembly	27	0.575202	0.008799	514	246	7937	1.694814	1.000000	0.265374	13.825718
GOTERM_MF_FAT	GO:0016563 transcription activator activity	14	0.298253	0.009058	534	93	7918	2.232129	0.996733	0.260107	12.651525
KEGG_PATHWAY	dme03022:Basal transcription factors	8	0.170430	0.009070	183	28	2054	3.206870	0.534830	0.225175	9.316952
SP_PIR_KEYWORDS	Aminoacyl-tRNA synthetase	7	0.149127	0.009201	806	30	12980	3.757651	0.873877	0.137476	11.130642
SP_PIR_KEYWORDS	repressor	13	0.276949	0.009435	806	90	12980	2.326165	0.880385	0.132001	11.398563
GOTERM_BP_FAT	GO:0051298 centrosome duplication	6	0.127823	0.009473	514	21	7937	4.411895	1.000000	0.277726	14.807010
GOTERM_BP_FAT	GO:0043039 tRNA aminoacylation	9	0.191734	0.009686	514	47	7937	2.956909	1.000000	0.278240	15.115544
GOTERM_BP_FAT	GO:0006418 tRNA aminoacylation for protein translation	9	0.191734	0.009686	514	47	7937	2.956909	1.000000	0.278240	15.115544
GOTERM_CC_FAT	GO:0005743 mitochondrial inner membrane	25	0.532595	0.009864	350	200	4786	1.709286	0.977335	0.135541	12.833170
SMART	SM00292:BRCT	5	0.106519	0.009893	310	14	4824	5.557604	0.853217	0.381031	11.651879
INTERPRO	IPR016135:Ubiquitin-conjugating enzyme/RWD-like	8	0.170430	0.010174	723	35	10196	3.223395	0.999988	0.849200	15.101641
GOTERM_BP_FAT	GO:0000381 regulation of alternative nuclear mRNA splicing, via spliceosome	10	0.213038	0.010214	514	57	7937	2.709059	1.000000	0.286221	15.874207
GOTERM_BP_FAT	GO:0043624 cellular protein complex disassembly	5	0.106519	0.010257	514	14	7937	5.514869	1.000000	0.282583	15.935733

GOTERM_BP_FAT	GO:0043241 protein complex disassembly	5	0.106519	0.010257	514	14	7937	5.514869	1.000000	0.282583	15.935733
GOTERM_BP_FAT	GO:0043038 amino acid activation	9	0.191734	0.010986	514	48	7937	2.895306	1.000000	0.294686	16.971946
SP_PIR_KEYWORDS	signal recognition particle	3	0.063911	0.011050	806	3	12980	16.104218	0.917006	0.144065	13.225121
GOTERM_BP_FAT	GO:0070271 protein complex biogenesis	20	0.426076	0.011192	514	167	7937	1.849298	1.000000	0.294686	17.262792
GOTERM_BP_FAT	GO:0006461 protein complex assembly	20	0.426076	0.011192	514	167	7937	1.849298	1.000000	0.294686	17.262792
GOTERM_BP_FAT	GO:0000002 mitochondrial genome maintenance	4	0.085215	0.011766	514	8	7937	7.720817	1.000000	0.302638	18.067823
GOTERM_MF_FAT	GO:0019899 enzyme binding	11	0.234342	0.012169	534	66	7918	2.471286	0.999548	0.319596	16.639864
GOTERM_CC_FAT	GO:0005694 chromosome	34	0.724329	0.012178	350	304	4786	1.529361	0.990725	0.159157	15.612686
GOTERM_BP_FAT	GO:0006796 phosphate metabolic process	50	1.065190	0.012849	514	551	7937	1.401237	1.000000	0.320791	19.567243
GOTERM_BP_FAT	GO:0006793 phosphorus metabolic process	50	1.065190	0.012849	514	551	7937	1.401237	1.000000	0.320791	19.567243
GOTERM_BP_FAT	GO:0033044 regulation of chromosome organization	5	0.106519	0.013285	514	15	7937	5.147211	1.000000	0.324980	20.162882
GOTERM_BP_FAT	GO:0006325 chromatin organization	20	0.426076	0.013381	514	170	7937	1.816663	1.000000	0.322286	20.292806
GOTERM_MF_FAT	GO:0016876 ligase activity, forming aminoacyl-tRNA and related compounds	9	0.191734	0.013822	534	48	7918	2.780197	0.999842	0.340914	18.689731
GOTERM_MF_FAT	GO:0004812 aminoacyl-tRNA ligase activity	9	0.191734	0.013822	534	48	7918	2.780197	0.999842	0.340914	18.689731
GOTERM_MF_FAT	GO:0016875 ligase activity, forming carbon-oxygen bonds	9	0.191734	0.013822	534	48	7918	2.780197	0.999842	0.340914	18.689731
INTERPRO	IPR001357:BRCT	5	0.106519	0.014076	723	14	10196	5.036554	1.000000	0.894385	20.304700
GOTERM_MF_FAT	GO:0004386 helicase activity	15	0.319557	0.014210	534	109	7918	2.040511	0.999877	0.335812	19.163440
GOTERM_BP_FAT	GO:0051276 chromosome organization	29	0.617810	0.014994	514	282	7937	1.587969	1.000000	0.348770	22.458914
GOTERM_CC_FAT	GO:0033279 ribosomal subunit	21	0.447380	0.015022	350	164	4786	1.750976	0.996917	0.186569	18.917096
GOTERM_CC_FAT	GO:0016581 NuRD complex	3	0.063911	0.015141	350	3	4786	13.674286	0.997056	0.182059	19.052725
GOTERM_BP_FAT	GO:0007033 vacuole organization	7	0.149127	0.015194	514	32	7937	3.377857	1.000000	0.347810	22.723870
GOTERM_MF_FAT	GO:0008134 transcription factor binding	13	0.276949	0.015626	534	89	7918	2.165846	0.999950	0.349958	20.872752
GOTERM_CC_FAT	GO:0016585 chromatin remodeling complex	8	0.170430	0.015792	350	37	4786	2.956602	0.997714	0.183471	19.791554
INTERPRO	IPR011993:Pleckstrin homology-type	15	0.319557	0.015830	723	105	10196	2.014622	1.000000	0.890743	22.544819
INTERPRO	IPR001849:Pleckstrin homology	12	0.255646	0.015922	723	75	10196	2.256376	1.000000	0.861867	22.660293
GOTERM_MF_FAT	GO:0004004 ATP-dependent RNA helicase activity	8	0.170430	0.016080	534	40	7918	2.965543	0.999963	0.346129	21.412729
GOTERM_MF_FAT	GO:0008186 RNA-dependent ATPase activity	8	0.170430	0.016080	534	40	7918	2.965543	0.999963	0.346129	21.412729
GOTERM_MF_FAT	GO:0003735 structural constituent of ribosome	21	0.447380	0.016177	534	178	7918	1.749337	0.999965	0.336572	21.527960
SP_PIR_KEYWORDS	chromatin regulator	10	0.213038	0.016632	806	64	12980	2.516284	0.976645	0.198281	19.274659
GOTERM_BP_FAT	GO:0006913 nucleocytoplasmic transport	11	0.234342	0.016730	514	72	7937	2.359139	1.000000	0.370787	24.728855
GOTERM_BP_FAT	GO:0051169 nuclear transport	11	0.234342	0.016730	514	72	7937	2.359139	1.000000	0.370787	24.728855
GOTERM_BP_FAT	GO:0006890 retrograde vesicle-mediated transport, Golgi to ER	4	0.085215	0.016812	514	9	7937	6.862949	1.000000	0.367493	24.834400
GOTERM_MF_FAT	GO:0016779 nucleotidyltransferase activity	13	0.276949	0.016970	534	90	7918	2.141781	0.999979	0.339043	22.463226
GOTERM_BP_FAT	GO:0042592 homeostatic process	19	0.404772	0.017687	514	163	7937	1.799945	1.000000	0.377779	25.952527
GOTERM_BP_FAT	GO:0007049 cell cycle	54	1.150405	0.017981	514	616	7937	1.353650	1.000000	0.378047	26.325245
KEGG_PATHWAY	dme00970:Aminoacyl-tRNA biosynthesis	9	0.191734	0.018245	183	39	2054	2.590164	0.787059	0.320695	17.934188
GOTERM_CC_FAT	GO:0005789 endoplasmic reticulum membrane	12	0.255646	0.018953	350	75	4786	2.187886	0.999331	0.210052	23.287295
GOTERM_BP_FAT	GO:0050684 regulation of mRNA processing	10	0.213038	0.019103	514	63	7937	2.451053	1.000000	0.391674	27.729840
GOTERM_BP_FAT	GO:0048024 regulation of nuclear mRNA splicing, via spliceosome	10	0.213038	0.019103	514	63	7937	2.451053	1.000000	0.391674	27.729840
GOTERM_BP_FAT	GO:0043414 biopolymer methylation	8	0.170430	0.019136	514	43	7937	2.872862	1.000000	0.387590	27.770092
GOTERM_MF_FAT	GO:0016791 phosphatase activity	21	0.447380	0.020166	534	182	7918	1.710890	0.999997	0.377862	26.127016
GOTERM_BP_FAT	GO:0006120 mitochondrial electron transport, NADH to ubiquinone	7	0.149127	0.020226	514	34	7937	3.179160	1.000000	0.400004	29.109700
GOTERM_CC_FAT	GO:0005740 mitochondrial envelope	29	0.617810	0.020316	350	258	4786	1.537032	0.999607	0.217311	24.751035
GOTERM_BP_FAT	GO:0009057 macromolecule catabolic process	28	0.596506	0.020819	514	277	7937	1.560887	1.000000	0.404431	29.828922
GOTERM_MF_FAT	GO:0046914 transition metal ion binding	110	2.343417	0.021191	534	1354	7918	1.204615	0.999999	0.381935	27.267697

GOTERM_BP_FAT	GO:0045664 regulation of neuron differentiation	9	0.191734	0.021622	514	54	7937	2.573606	1.000000	0.411769	30.791175
GOTERM_CC_FAT	GO:0042175 nuclear envelope-endoplasmic reticulum network	12	0.255646	0.022707	350	77	4786	2.131058	0.999845	0.233467	27.255605
SP_PIR_KEYWORDS	kinase	22	0.468683	0.022726	806	212	12980	1.671192	0.994197	0.248793	25.433085
SP_PIR_KEYWORDS	zinc	49	1.043886	0.022736	806	577	12980	1.367603	0.994211	0.237494	25.443375
GOTERM_BP_FAT	GO:0010507 negative regulation of autophagy	4	0.085215	0.022883	514	10	7937	6.176654	1.000000	0.425309	32.278163
GOTERM_BP_FAT	GO:0006448 regulation of translational elongation	3	0.063911	0.022923	514	4	7937	11.581226	1.000000	0.421367	32.324821
GOTERM_CC_FAT	GO:0070603 SWI/SNF-type complex	4	0.085215	0.023196	350	9	4786	6.077460	0.999872	0.231780	27.758154
GOTERM_CC_FAT	GO:0035060 brahma complex	4	0.085215	0.023196	350	9	4786	6.077460	0.999872	0.231780	27.758154
GOTERM_MF_FAT	GO:0003954 NADH dehydrogenase activity	8	0.170430	0.023376	534	43	7918	2.758645	1.000000	0.401320	29.643455
GOTERM_BP_FAT	GO:0006730 one-carbon metabolic process	11	0.234342	0.023693	514	76	7937	2.234973	1.000000	0.427546	33.216966
SP_PIR_KEYWORDS	transit peptide	14	0.298253	0.024122	806	114	12980	1.977711	0.995787	0.239270	26.781485
GOTERM_MF_FAT	GO:0016651 oxidoreductase activity, acting on NADH or NADPH	9	0.191734	0.024229	534	53	7918	2.517914	1.000000	0.402058	30.552079
SP_PIR_KEYWORDS	mrna processing	8	0.170430	0.024716	806	47	12980	2.741144	0.996324	0.234287	27.348389
SP_PIR_KEYWORDS	DNA damage	7	0.149127	0.024975	806	37	12980	3.046744	0.996537	0.227036	27.594733
GOTERM_BP_FAT	GO:0043484 regulation of RNA splicing	10	0.213038	0.025209	514	66	7937	2.339642	1.000000	0.443367	34.942136
GOTERM_BP_FAT	GO:0007099 centriole replication	5	0.106519	0.025541	514	18	7937	4.289343	1.000000	0.443254	35.314367
GOTERM_BP_FAT	GO:0007067 mitosis	17	0.362164	0.026034	514	146	7937	1.797999	1.000000	0.445188	35.862573
GOTERM_BP_FAT	GO:0019725 cellular homeostasis	14	0.298253	0.026326	514	111	7937	1.947594	1.000000	0.444553	36.185453
GOTERM_CC_FAT	GO:0031966 mitochondrial membrane	27	0.575202	0.026567	350	241	4786	1.531974	0.999966	0.254633	31.136789
GOTERM_BP_FAT	GO:0050767 regulation of neurogenesis	10	0.213038	0.027521	514	67	7937	2.304722	1.000000	0.455055	37.491931
GOTERM_CC_FAT	GO:0005815 microtubule organizing center	10	0.213038	0.028300	350	60	4786	2.279048	0.999983	0.262598	32.815956
GOTERM_CC_FAT	GO:0005840 ribosome	22	0.468683	0.028353	350	186	4786	1.617389	0.999983	0.256922	32.866952
GOTERM_BP_FAT	GO:0000280 nuclear division	17	0.362164	0.029116	514	148	7937	1.773701	1.000000	0.469816	39.195572
GOTERM_BP_FAT	GO:0000087 M phase of mitotic cell cycle	17	0.362164	0.029116	514	148	7937	1.773701	1.000000	0.469816	39.195572
INTERPRO	IPR011545:DNA/RNA helicase, DEAD/DEAH box type, N-terminal	8	0.170430	0.029931	723	43	10196	2.623693	1.000000	0.965717	38.523307
GOTERM_BP_FAT	GO:0010506 regulation of autophagy	4	0.085215	0.029983	514	11	7937	5.615140	1.000000	0.475573	40.103745
KEGG_PATHWAY	dme03420:Nucleotide excision repair	8	0.170430	0.030348	183	35	2054	2.565496	0.924883	0.404135	28.164740
GOTERM_BP_FAT	GO:0008213 protein amino acid alkylation	6	0.127823	0.031560	514	28	7937	3.308922	1.000000	0.489023	41.721510
GOTERM_BP_FAT	GO:0006479 protein amino acid methylation	6	0.127823	0.031560	514	28	7937	3.308922	1.000000	0.489023	41.721510
SP_PIR_KEYWORDS	ribonucleoprotein	15	0.319557	0.032416	806	131	12980	1.843994	0.999377	0.274532	34.340894
SP_PIR_KEYWORDS	hydrolase	109	2.322113	0.032555	806	1470	12980	1.194122	0.999397	0.265746	34.460627
UP_SEQ_FEATURE	domain:Chromo 2	3	0.063911	0.032703	233	4	2975	9.576180	1.000000	0.999946	38.740584
UP_SEQ_FEATURE	domain:Chromo 1	3	0.063911	0.032703	233	4	2975	9.576180	1.000000	0.999946	38.740584
GOTERM_MF_FAT	GO:0016887 ATPase activity	34	0.724329	0.033151	534	353	7918	1.428165	1.000000	0.495431	39.415661
SP_PIR_KEYWORDS	nucleotidyltransferase	10	0.213038	0.033263	806	72	12980	2.236697	0.999488	0.261479	35.070473
INTERPRO	IPR000629:RNA helicase, ATP-dependent, DEAD-box, conserved site	6	0.127823	0.033302	723	26	10196	3.254389	1.000000	0.967212	41.855485
GOTERM_MF_FAT	GO:0042624 ATPase activity, uncoupled	21	0.447380	0.033463	534	192	7918	1.621781	1.000000	0.487786	39.705342
GOTERM_BP_FAT	GO:0030163 protein catabolic process	21	0.447380	0.033658	514	200	7937	1.621372	1.000000	0.507367	43.811289
GOTERM_CC_FAT	GO:0005700 polytene chromosome	13	0.276949	0.033829	350	92	4786	1.932236	0.999998	0.292455	37.923089
UP_SEQ_FEATURE	compositionally biased region:Poly-Asp	7	0.149127	0.034234	233	32	2975	2.793053	1.000000	0.998953	40.154107
SMART	SM00391:MBD	3	0.063911	0.035941	310	5	4824	9.336774	0.999145	0.756555	36.624899
GOTERM_MF_FAT	GO:0008092 cytoskeletal protein binding	23	0.489987	0.036288	534	218	7918	1.564392	1.000000	0.505658	42.272315
GOTERM_BP_FAT	GO:0051261 protein depolymerization	3	0.063911	0.036583	514	5	7937	9.264981	1.000000	0.532936	46.607628
GOTERM_BP_FAT	GO:0016575 histone deacetylation	3	0.063911	0.036583	514	5	7937	9.264981	1.000000	0.532936	46.607628
GOTERM_MF_FAT	GO:0015631 tubulin binding	10	0.213038	0.037522	534	68	7918	2.180546	1.000000	0.507135	43.361865
GOTERM_BP_FAT	GO:0048285 organelle fission	17	0.362164	0.037996	514	153	7937	1.715737	1.000000	0.542390	47.910995
GOTERM_CC_FAT	GO:0005747 mitochondrial respiratory chain complex I	8	0.170430	0.038028	350	44	4786	2.486234	1.000000	0.315966	41.558362
GOTERM_CC_FAT	GO:0030964 NADH dehydrogenase complex	8	0.170430	0.038028	350	44	4786	2.486234	1.000000	0.315966	41.558362
GOTERM_CC_FAT	GO:0045271 respiratory chain complex I	8	0.170430	0.038028	350	44	4786	2.486234	1.000000	0.315966	41.558362
GOTERM_BP_FAT	GO:0031056 regulation of histone modification	4	0.085215	0.038103	514	12	7937	5.147211	1.000000	0.539134	48.008402
GOTERM_BP_FAT	GO:0031330 negative regulation of cellular catabolic process	4	0.085215	0.038103	514	12	7937	5.147211	1.000000	0.539134	48.008402

GOTERM_BP_FAT	GO:0034621 cellular macromolecular complex subunit organization	21	0.447380	0.038503	514	203	7937	1.597410	1.000000	0.538706	48.370679
SMART	SM00212:UBCc	6	0.127823	0.039657	310	30	4824	3.112258	0.999594	0.727911	39.602849
GOTERM_BP_FAT	GO:0032268 regulation of cellular protein metabolic process	15	0.319557	0.040678	514	130	7937	1.781727	1.000000	0.554622	50.302340
GOTERM_BP_FAT	GO:0007040 lysosome organization	6	0.127823	0.041195	514	30	7937	3.088327	1.000000	0.555109	50.751918
SP_PIR_KEYWORDS	serine/threonine-protein kinase	16	0.340861	0.041220	806	148	12980	1.740997	0.999920	0.304173	41.572396
GOTERM_BP_FAT	GO:0051174 regulation of phosphorus metabolic process	10	0.213038	0.041337	514	72	7937	2.144671	1.000000	0.552255	50.874148
GOTERM_BP_FAT	GO:0019220 regulation of phosphate metabolic process	10	0.213038	0.041337	514	72	7937	2.144671	1.000000	0.552255	50.874148
GOTERM_MF_FAT	GO:0003723 RNA binding	53	1.129101	0.042247	534	610	7918	1.288310	1.000000	0.539640	47.357110
GOTERM_MF_FAT	GO:0019213 deacetylase activity	4	0.085215	0.042266	534	12	7918	4.942572	1.000000	0.529771	47.372300
GOTERM_BP_FAT	GO:0007030 Golgi organization	5	0.106519	0.042895	514	21	7937	3.676580	1.000000	0.561821	52.201759
GOTERM_BP_FAT	GO:0060284 regulation of cell development	15	0.319557	0.042972	514	131	7937	1.768126	1.000000	0.558442	52.266012
INTERPRO	IPR001739:Methyl-CpG DNA binding	3	0.063911	0.043372	723	5	10196	8.461411	1.000000	0.983452	50.830008
GOTERM_MF_FAT	GO:0003724 RNA helicase activity	8	0.170430	0.044179	534	49	7918	2.420851	1.000000	0.536122	48.913385
GOTERM_BP_FAT	GO:0043549 regulation of kinase activity	8	0.170430	0.044276	514	51	7937	2.422217	1.000000	0.565503	53.349591
GOTERM_BP_FAT	GO:0045859 regulation of protein kinase activity	8	0.170430	0.044276	514	51	7937	2.422217	1.000000	0.565503	53.349591
GOTERM_BP_FAT	GO:0051338 regulation of transferase activity	8	0.170430	0.044276	514	51	7937	2.422217	1.000000	0.565503	53.349591
GOTERM_BP_FAT	GO:0007005 mitochondrion organization	10	0.213038	0.044575	514	73	7937	2.115292	1.000000	0.564040	53.594852
GOTERM_BP_FAT	GO:0008363 larval chitin-based cuticle development	4	0.085215	0.047220	514	13	7937	4.751272	1.000000	0.581550	55.710715
GOTERM_BP_FAT	GO:0009895 negative regulation of catabolic process	4	0.085215	0.047220	514	13	7937	4.751272	1.000000	0.581550	55.710715
GOTERM_CC_FAT	GO:0044427 chromosomal part	26	0.553899	0.047948	350	243	4786	1.463092	1.000000	0.374526	49.376428
GOTERM_MF_FAT	GO:0016879 ligase activity, forming carbon-nitrogen bonds	16	0.340861	0.048974	534	140	7918	1.694596	1.000000	0.564462	52.593598
GOTERM_MF_FAT	GO:0004842 ubiquitin-protein ligase activity	11	0.234342	0.051043	534	83	7918	1.965119	1.000000	0.570435	54.103555
INTERPRO	IPR012677:Nucleotide-binding, alpha-beta plait	16	0.340861	0.051551	723	134	10196	1.683863	1.000000	0.989102	57.145284
GOTERM_CC_FAT	GO:0005849 mRNA cleavage factor complex	4	0.085215	0.051626	350	12	4786	4.558095	1.000000	0.389734	52.019450
GOTERM_CC_FAT	GO:0031523 Myb complex	4	0.085215	0.051626	350	12	4786	4.558095	1.000000	0.389734	52.019450
GOTERM_BP_FAT	GO:0032259 methylation	8	0.170430	0.052849	514	53	7937	2.330813	1.000000	0.619980	59.916594
GOTERM_BP_FAT	GO:0043067 regulation of programmed cell death	13	0.276949	0.054052	514	111	7937	1.808480	1.000000	0.624591	60.764613
GOTERM_BP_FAT	GO:0016310 phosphorylation	37	0.788240	0.056127	514	425	7937	1.344331	1.000000	0.635000	62.189149
GOTERM_BP_FAT	GO:0044265 cellular macromolecule catabolic process	22	0.468683	0.056229	514	225	7937	1.509849	1.000000	0.631877	62.258048
KEGG_PATHWAY	dme03018:RNA degradation	9	0.191734	0.056451	183	48	2054	2.104508	0.992411	0.556696	46.404920
GOTERM_BP_FAT	GO:0048193 Golgi vesicle transport	7	0.149127	0.056452	514	43	7937	2.513754	1.000000	0.629598	62.407435
GOTERM_BP_FAT	GO:0045893 positive regulation of transcription, DNA-dependent	11	0.234342	0.056687	514	88	7937	1.930204	1.000000	0.627439	62.564916
GOTERM_BP_FAT	GO:0042325 regulation of phosphorylation	9	0.191734	0.056877	514	65	7937	2.138072	1.000000	0.625007	62.691439
GOTERM_CC_FAT	GO:0046930 pore complex	8	0.170430	0.057090	350	48	4786	2.279048	1.000000	0.414133	55.711208
INTERPRO	IPR000608:Ubiquitin-conjugating enzyme, E2	6	0.127823	0.057280	723	30	10196	2.820470	1.000000	0.990691	61.107150
GOTERM_BP_FAT	GO:0042336 protein-based cuticle development during molting	4	0.085215	0.057301	514	14	7937	4.411895	1.000000	0.624161	62.972874
GOTERM_BP_FAT	GO:0042337 chitin-based cuticle development during molting	4	0.085215	0.057301	514	14	7937	4.411895	1.000000	0.624161	62.972874
GOTERM_BP_FAT	GO:0007592 protein-based cuticle development	4	0.085215	0.057301	514	14	7937	4.411895	1.000000	0.624161	62.972874
GOTERM_BP_FAT	GO:0006379 mRNA cleavage	4	0.085215	0.057301	514	14	7937	4.411895	1.000000	0.624161	62.972874
GOTERM_BP_FAT	GO:0031329 regulation of cellular catabolic process	4	0.085215	0.057301	514	14	7937	4.411895	1.000000	0.624161	62.972874
GOTERM_MF_FAT	GO:0008017 microtubule binding	9	0.191734	0.059285	534	63	7918	2.118245	1.000000	0.617502	59.685177
INTERPRO	IPR009091:Regulator of chromosome condensation/beta-lactamase-inhibitor protein II	4	0.085215	0.059301	723	13	10196	4.339185	1.000000	0.989152	62.420560
GOTERM_MF_FAT	GO:0008276 protein methyltransferase activity	6	0.127823	0.060511	534	32	7918	2.780197	1.000000	0.616188	60.459301

GOTERM_MF_FAT	GO:0042623 ATPase activity, coupled	29	0.617810	0.060747	534	307	7918	1.400664	1.000000	0.608813	60.606890
GOTERM_CC_FAT	GO:0031227 intrinsic to endoplasmic reticulum membrane	5	0.106519	0.061925	350	21	4786	3.255782	1.000000	0.433285	58.756006
GOTERM_BP_FAT	GO:0031399 regulation of protein modification process	7	0.149127	0.061965	514	44	7937	2.456624	1.000000	0.650223	65.939726
INTERPRO	IPR001431:Peptidase M16, zinc-binding site	3	0.063911	0.062051	723	6	10196	7.051176	1.000000	0.988253	64.141512
SP_PIR_KEYWORDS	dna repair	6	0.127823	0.062886	806	35	12980	2.760723	1.000000	0.416582	56.359329
GOTERM_BP_FAT	GO:0051960 regulation of nervous system development	11	0.234342	0.064183	514	90	7937	1.887311	1.000000	0.659979	67.270252
GOTERM_BP_FAT	GO:0051254 positive regulation of RNA metabolic process	11	0.234342	0.064183	514	90	7937	1.887311	1.000000	0.659979	67.270252
GOTERM_BP_FAT	GO:0022900 electron transport chain	11	0.234342	0.064183	514	90	7937	1.887311	1.000000	0.659979	67.270252
UP_SEQ_FEATURE	compositionally biased region:Poly-Lys	5	0.106519	0.064809	233	20	2975	3.192060	1.000000	0.999950	62.751541
GOTERM_BP_FAT	GO:0017148 negative regulation of translation	6	0.127823	0.065275	514	34	7937	2.724994	1.000000	0.662835	67.907507
GOTERM_CC_FAT	GO:0035062 omega speckle	3	0.063911	0.065368	350	6	4786	6.837143	1.000000	0.443959	60.804363
GOTERM_BP_FAT	GO:0042775 mitochondrial ATP synthesis coupled electron transport	9	0.191734	0.065817	514	67	7937	2.074249	1.000000	0.662465	68.219556
GOTERM_MF_FAT	GO:0030528 transcription regulator activity	57	1.214316	0.065883	534	682	7918	1.239267	1.000000	0.630996	63.690206
GOTERM_CC_FAT	GO:0015934 large ribosomal subunit	13	0.276949	0.065935	350	102	4786	1.742801	1.000000	0.439551	61.132216
SP_PIR_KEYWORDS	metal-binding	61	1.299531	0.066523	806	796	12980	1.234117	1.000000	0.423461	58.472746
GOTERM_BP_FAT	GO:0010941 regulation of cell death	13	0.276949	0.067221	514	115	7937	1.745576	1.000000	0.666998	69.014311
GOTERM_CC_FAT	GO:0031300 intrinsic to organelle membrane	7	0.149127	0.067407	350	40	4786	2.393000	1.000000	0.439837	61.972344
SP_PIR_KEYWORDS	Transcription	33	0.703025	0.069371	806	393	12980	1.352263	1.000000	0.426115	60.061638
GOTERM_BP_FAT	GO:0032042 mitochondrial DNA metabolic process	3	0.063911	0.070493	514	7	7937	6.617843	1.000000	0.681565	70.794299
GOTERM_BP_FAT	GO:0006264 mitochondrial DNA replication	3	0.063911	0.070493	514	7	7937	6.617843	1.000000	0.681565	70.794299
GOTERM_CC_FAT	GO:0044450 microtubule organizing center part	5	0.106519	0.071517	350	22	4786	3.107792	1.000000	0.452884	64.229505
SMART	SM00717:SANT	5	0.106519	0.072012	310	25	4824	3.112258	0.999999	0.872622	60.595033
INTERPRO	IPR014014:RNA helicase, DEAD-box type, Q motif	6	0.127823	0.072185	723	32	10196	2.644191	1.000000	0.992494	69.865536
GOTERM_BP_FAT	GO:0045216 cell-cell junction organization	6	0.127823	0.072300	514	35	7937	2.647137	1.000000	0.687742	71.735847
SP_PIR_KEYWORDS	kelch repeat	4	0.085215	0.072732	806	16	12980	4.026055	1.000000	0.430972	61.864088
GOTERM_BP_FAT	GO:0006261 DNA-dependent DNA replication	8	0.170430	0.072931	514	57	7937	2.167247	1.000000	0.687669	72.057427
GOTERM_CC_FAT	GO:0044431 Golgi apparatus part	11	0.234342	0.073001	350	82	4786	1.834355	1.000000	0.452975	65.013518
GOTERM_MF_FAT	GO:0017016 Ras GTPase binding	5	0.106519	0.073826	534	24	7918	3.089107	1.000000	0.665914	68.018341
GOTERM_MF_FAT	GO:0031267 small GTPase binding	5	0.106519	0.073826	534	24	7918	3.089107	1.000000	0.665914	68.018341
SP_PIR_KEYWORDS	peroxidase	5	0.106519	0.074065	806	26	12980	3.096965	1.000000	0.426520	62.558056
GOTERM_CC_FAT	GO:0000139 Golgi membrane	7	0.149127	0.074355	350	41	4786	2.334634	1.000000	0.452477	65.715311
GOTERM_BP_FAT	GO:0010604 positive regulation of macromolecule metabolic process	15	0.319557	0.074425	514	142	7937	1.631159	1.000000	0.692005	72.806000
GOTERM_BP_FAT	GO:0022402 cell cycle process	45	0.958671	0.074450	514	547	7937	1.270336	1.000000	0.688847	72.818379
GOTERM_MF_FAT	GO:0019787 small conjugating protein ligase activity	11	0.234342	0.074985	534	89	7918	1.832639	1.000000	0.663618	68.608567
GOTERM_MF_FAT	GO:0019894 kinesin binding	3	0.063911	0.075774	534	7	7918	6.354735	1.000000	0.659547	69.003807
GOTERM_BP_FAT	GO:0006606 protein import into nucleus	6	0.127823	0.079722	514	36	7937	2.573606	1.000000	0.711335	75.311277
GOTERM_BP_FAT	GO:0034330 cell junction organization	6	0.127823	0.079722	514	36	7937	2.573606	1.000000	0.711335	75.311277
INTERPRO	IPR001412:Aminoacyl-tRNA synthetase, class I, conserved site	6	0.127823	0.080358	723	33	10196	2.564064	1.000000	0.994292	73.845765
GOTERM_BP_FAT	GO:0017038 protein import	9	0.191734	0.080772	514	70	7937	1.985353	1.000000	0.713040	75.781183
GOTERM_CC_FAT	GO:0005813 centrosome	8	0.170430	0.081184	350	52	4786	2.103736	1.000000	0.476319	69.058016
GOTERM_MF_FAT	GO:0003677 DNA binding	67	1.427354	0.081586	534	830	7918	1.196936	1.000000	0.679856	71.778462
SMART	SM00184:RING	14	0.298253	0.082154	310	133	4824	1.638031	1.000000	0.873579	65.637834
GOTERM_BP_FAT	GO:0022604 regulation of cell morphogenesis	13	0.276949	0.082329	514	119	7937	1.686901	1.000000	0.717017	76.462682
INTERPRO	IPR011237:Peptidase M16, core	3	0.063911	0.082883	723	7	10196	6.043865	1.000000	0.993620	74.971821
INTERPRO	IPR007863:Peptidase M16, C-terminal	3	0.063911	0.082883	723	7	10196	6.043865	1.000000	0.993620	74.971821
INTERPRO	IPR019775:WD40 repeat, conserved site	15	0.319557	0.083538	723	132	10196	1.602540	1.000000	0.992105	75.256386
SP_PIR_KEYWORDS	one-carbon metabolism	3	0.063911	0.083837	806	8	12980	6.039082	1.000000	0.458237	67.301133
GOTERM_BP_FAT	GO:0042773 ATP synthesis coupled electron transport	9	0.191734	0.086170	514	71	7937	1.957390	1.000000	0.730851	78.067683
GOTERM_BP_FAT	GO:0051170 nuclear import	6	0.127823	0.087535	514	37	7937	2.504049	1.000000	0.733601	78.612811
SMART	SM00320:WD40	18	0.383468	0.088709	310	187	4824	1.497878	1.000000	0.863584	68.573742

GOTERM_MF_FAT	GO:0004674 protein serine/threonine kinase activity	20	0.426076	0.089331	534	203	7918	1.460859	1.000000	0.706606	75.116797
GOTERM_BP_FAT	GO:0035293 chitin-based larval cuticle pattern formation	3	0.063911	0.090069	514	8	7937	5.790613	1.000000	0.741085	79.590859
GOTERM_BP_FAT	GO:0051188 cofactor biosynthetic process	8	0.170430	0.090583	514	60	7937	2.058885	1.000000	0.740196	79.784164
GOTERM_MF_FAT	GO:0004527 exonuclease activity	6	0.127823	0.091253	534	36	7918	2.471286	1.000000	0.707217	75.885785
GOTERM_BP_FAT	GO:0019991 septate junction assembly	4	0.085215	0.092886	514	17	7937	3.633326	1.000000	0.746450	80.629176
GOTERM_BP_FAT	GO:0045860 positive regulation of protein kinase activity	5	0.106519	0.093096	514	27	7937	2.859562	1.000000	0.744361	80.704506
GOTERM_BP_FAT	GO:0043297 apical junction assembly	5	0.106519	0.093096	514	27	7937	2.859562	1.000000	0.744361	80.704506
GOTERM_BP_FAT	GO:0051347 positive regulation of transferase activity	5	0.106519	0.093096	514	27	7937	2.859562	1.000000	0.744361	80.704506
GOTERM_BP_FAT	GO:0033674 positive regulation of kinase activity	5	0.106519	0.093096	514	27	7937	2.859562	1.000000	0.744361	80.704506
GOTERM_BP_FAT	GO:0016311 dephosphorylation	12	0.255646	0.093115	514	109	7937	1.699996	1.000000	0.741538	80.711276
GOTERM_BP_FAT	GO:0033043 regulation of organelle organization	12	0.255646	0.093115	514	109	7937	1.699996	1.000000	0.741538	80.711276
GOTERM_BP_FAT	GO:0042981 regulation of apoptosis	11	0.234342	0.095258	514	97	7937	1.751113	1.000000	0.747007	81.464392
SP_PIR_KEYWORDS	protein transport	11	0.234342	0.095601	806	101	12980	1.753925	1.000000	0.494433	72.274386
GOTERM_BP_FAT	GO:0031344 regulation of cell projection organization	6	0.127823	0.095732	514	38	7937	2.438153	1.000000	0.745985	81.627412
GOTERM_BP_FAT	GO:0034504 protein localization in nucleus	6	0.127823	0.095732	514	38	7937	2.438153	1.000000	0.745985	81.627412
GOTERM_BP_FAT	GO:0009451 RNA modification	6	0.127823	0.095732	514	38	7937	2.438153	1.000000	0.745985	81.627412
INTERPRO	IPR001005:SANT, DNA-binding	5	0.106519	0.096462	723	25	10196	2.820470	1.000000	0.995307	80.288847
GOTERM_MF_FAT	GO:0004407 histone deacetylase activity	3	0.063911	0.096646	534	8	7918	5.560393	1.000000	0.721584	77.927771
GOTERM_MF_FAT	GO:0033558 protein deacetylase activity	3	0.063911	0.096646	534	8	7918	5.560393	1.000000	0.721584	77.927771
GOTERM_MF_FAT	GO:0008536 Ran GTPase binding	3	0.063911	0.096646	534	8	7918	5.560393	1.000000	0.721584	77.927771
GOTERM_BP_FAT	GO:0006979 response to oxidative stress	8	0.170430	0.096956	514	61	7937	2.025132	1.000000	0.747820	82.041729
UP_SEQ_FEATURE	mutagenesis site	22	0.468683	0.097642	233	201	2975	1.397519	1.000000	0.999995	78.004201
SP_PIR_KEYWORDS	cytoskeleton	10	0.213038	0.099742	806	89	12980	1.809463	1.000000	0.499553	73.852253

Table 26: Architecture 26

Category	Term	Count	%	PValue	List Total	Pop Hits	Pop Total	Fold Enrichment	Bonferroni	Benjamini	FDR
GOTERM_BP_FAT	GO:0000278 mitotic cell cycle	9	2.941176	0.003375	57	358	7937	3.500588	0.868480	0.868480	4.870847
SP_PIR_KEYWORDS	phosphoprotein	13	4.248366	0.004939	83	815	12980	2.494493	0.356379	0.356379	5.232654
SP_PIR_KEYWORDS	alternative splicing	11	3.594771	0.005292	83	616	12980	2.792599	0.376391	0.210311	5.597059
GOTERM_BP_FAT	GO:0000279 M phase	10	3.267974	0.005806	57	478	7937	2.913088	0.969619	0.825700	8.241043
GOTERM_BP_FAT	GO:0022403 cell cycle phase	10	3.267974	0.007360	57	496	7937	2.807371	0.988115	0.771791	10.336573
GOTERM_BP_FAT	GO:0007052 mitotic spindle organization	6	1.960784	0.011543	57	194	7937	4.306565	0.999057	0.824750	15.757862
GOTERM_BP_FAT	GO:0022402 cell cycle process	10	3.267974	0.013532	57	547	7937	2.545624	0.999718	0.805038	18.227160
GOTERM_BP_FAT	GO:0051276 chromosome organization	7	2.287582	0.013957	57	282	7937	3.456451	0.999783	0.754771	18.746090
GOTERM_MF_FAT	GO:0001882 nucleoside binding	11	3.594771	0.017853	47	824	7918	2.248967	0.923923	0.923923	19.211712
GOTERM_BP_FAT	GO:0000226 microtubule cytoskeleton organization	7	2.287582	0.017886	57	298	7937	3.270870	0.999980	0.787110	23.398728
GOTERM_BP_FAT	GO:0007067 mitosis	5	1.633987	0.019271	57	146	7937	4.768685	0.999991	0.767631	24.978498
GOTERM_BP_FAT	GO:0000087 M phase of mitotic cell cycle	5	1.633987	0.020152	57	148	7937	4.704244	0.999995	0.742609	25.967262
GOTERM_BP_FAT	GO:0000280 nuclear division	5	1.633987	0.020152	57	148	7937	4.704244	0.999995	0.742609	25.967262
GOTERM_BP_FAT	GO:0007051 spindle organization	6	1.960784	0.020706	57	225	7937	3.713216	0.999996	0.715033	26.583211
SP_PIR_KEYWORDS	transferase	12	3.921569	0.020982	83	873	12980	2.149629	0.848515	0.466924	20.561730
GOTERM_BP_FAT	GO:0048285 organelle fission	5	1.633987	0.022461	57	153	7937	4.550510	0.999999	0.710361	28.503058
INTERPRO	IPR008271:Serine/threonine protein kinase, active site	5	1.633987	0.024295	73	156	10196	4.476642	0.986150	0.986150	26.009039
GOTERM_MF_FAT	GO:0005524 ATP binding	10	3.267974	0.026021	47	748	7918	2.252247	0.976956	0.848196	26.820073
GOTERM_MF_FAT	GO:0032559 adenyl ribonucleotide binding	10	3.267974	0.026425	47	750	7918	2.246241	0.978281	0.720993	27.178084
GOTERM_BP_FAT	GO:0007049 cell cycle	10	3.267974	0.027230	57	616	7937	2.260481	1.000000	0.748521	33.485323
GOTERM_MF_FAT	GO:0004674 protein serine/threonine kinase activity	5	1.633987	0.029591	47	203	7918	4.149460	0.986368	0.658302	29.933576
INTERPRO	IPR017442:Serine/threonine protein kinase-related	5	1.633987	0.030783	73	168	10196	4.156882	0.995663	0.934141	31.815346
SP_PIR_KEYWORDS	atp-binding	9	2.941176	0.031102	83	585	12980	2.405931	0.939916	0.504903	29.034730
GOTERM_BP_FAT	GO:0016572 histone phosphorylation	2	0.653595	0.034792	57	5	7937	55.698246	1.000000	0.804929	40.726369
GOTERM_BP_FAT	GO:0006468 protein amino acid phosphorylation	6	1.960784	0.035184	57	259	7937	3.225767	1.000000	0.784559	41.081077
SMART	SM00220:S_TKc	4	1.307190	0.037086	33	112	4824	5.220779	0.752974	0.752974	28.775254
GOTERM_MF_FAT	GO:0000166 nucleotide binding	13	4.248366	0.038825	47	1206	7918	1.815991	0.996527	0.677779	37.434784
GOTERM_MF_FAT	GO:0030554 adenyl nucleotide binding	10	3.267974	0.041278	47	812	7918	2.074730	0.997590	0.633832	39.299660
GOTERM_BP_FAT	GO:0000070 mitotic sister chromatid segregation	3	0.980392	0.041519	57	46	7937	9.081236	1.000000	0.816628	46.543586
GOTERM_MF_FAT	GO:0001883 purine nucleoside binding	10	3.267974	0.042685	47	817	7918	2.062033	0.998046	0.589817	40.346594
GOTERM_BP_FAT	GO:0000819 sister chromatid segregation	3	0.980392	0.043172	57	47	7937	8.888018	1.000000	0.808897	47.888987
GOTERM_BP_FAT	GO:0007010 cytoskeleton organization	8	2.614379	0.043813	57	465	7937	2.395623	1.000000	0.794279	48.401918
INTERPRO	IPR002290:Serine/threonine protein kinase	4	1.307190	0.044567	73	112	10196	4.988258	0.999641	0.928943	42.786715
SP_PIR_KEYWORDS	kinase	5	1.633987	0.045134	83	212	12980	3.688338	0.983599	0.560485	39.428543
SP_PIR_KEYWORDS	nucleus	11	3.594771	0.046527	83	869	12980	1.979564	0.985597	0.506738	40.380491
GOTERM_BP_FAT	GO:0034331 cell junction maintenance	2	0.653595	0.048374	57	7	7937	39.784461	1.000000	0.808479	51.919839
GOTERM_CC_FAT	GO:0000775 chromosome, centromeric region	3	0.980392	0.048531	35	50	4786	8.204571	0.998367	0.998367	43.928819
GOTERM_BP_FAT	GO:0007059 chromosome segregation	4	1.307190	0.049232	57	117	7937	4.760534	1.000000	0.796945	52.556596
GOTERM_BP_FAT	GO:0051301 cell division	5	1.633987	0.056024	57	205	7937	3.396234	1.000000	0.822653	57.323395
GOTERM_BP_FAT	GO:0006020 inositol metabolic process	2	0.653595	0.061767	57	9	7937	30.943470	1.000000	0.838240	61.001556
INTERPRO	IPR001179:Peptidyl-prolyl cis-trans isomerase, FKBP-type	2	0.653595	0.061812	73	9	10196	31.038052	0.999985	0.937683	54.226500
GOTERM_CC_FAT	GO:0051233 spindle midzone	2	0.653595	0.062200	35	9	4786	30.387302	0.999748	0.984110	52.613269
GOTERM_BP_FAT	GO:0007017 microtubule-based process	7	2.287582	0.065052	57	406	7937	2.400786	1.000000	0.840306	62.970353
GOTERM_BP_FAT	GO:0048749 compound eye development	6	1.960784	0.065103	57	308	7937	2.712577	1.000000	0.827292	63.000047
UP_SEQ_FEATURE	splice variant	11	3.594771	0.065628	31	604	2975	1.747757	0.999863	0.999863	54.687469
INTERPRO	IPR000719:Protein kinase, core	5	1.633987	0.066146	73	216	10196	3.233130	0.999993	0.907593	56.750202
SP_PIR_KEYWORDS	serine/threonine-protein kinase	4	1.307190	0.067104	83	148	12980	4.226636	0.997934	0.586522	52.953043
GOTERM_BP_FAT	GO:0007423 sensory organ development	7	2.287582	0.067564	57	410	7937	2.377364	1.000000	0.826029	64.412558
SMART	SM00645:Pept_C1	2	0.653595	0.070667	33	11	4824	26.578512	0.933573	0.742265	48.214649
INTERPRO	IPR013201:Proteinase inhibitor I29, cathepsin propeptide	2	0.653595	0.075028	73	11	10196	25.394770	0.999999	0.895832	61.527166
INTERPRO	IPR000668:Peptidase C1A, papain C-terminal	2	0.653595	0.075028	73	11	10196	25.394770	0.999999	0.895832	61.527166
SP_PIR_KEYWORDS	nucleotidyltransferase	3	0.980392	0.075891	83	72	12980	6.516064	0.999110	0.584406	57.546439
GOTERM_BP_FAT	GO:0016310 phosphorylation	7	2.287582	0.077489	57	425	7937	2.293457	1.000000	0.855682	69.615101
GOTERM_MF_FAT	GO:0032553 ribonucleotide binding	10	3.267974	0.080650	47	923	7918	1.825223	0.999994	0.777557	63.059058
GOTERM_MF_FAT	GO:0032555 purine ribonucleotide binding	10	3.267974	0.080650	47	923	7918	1.825223	0.999994	0.777557	63.059058
GOTERM_MF_FAT	GO:0004672 protein kinase activity	5	1.633987	0.080874	47	283	7918	2.976468	0.999994	0.738140	63.165536
INTERPRO	IPR013128:Peptidase C1A, papain	2	0.653595	0.081567	73	12	10196	23.278539	1.000000	0.879365	64.728931
GOTERM_BP_FAT	GO:0001654 eye development	6	1.960784	0.083777	57	332	7937	2.516487	1.000000	0.867228	72.534530
GOTERM_CC_FAT	GO:0044454 nuclear chromosome part	3	0.980392	0.085553	35	69	4786	5.945342	0.999990	0.978630	64.657833
GOTERM_CC_FAT	GO:0044427 chromosomal part	5	1.633987	0.091311	35	243	4786	2.813639	0.999996	0.954408	67.160924
GOTERM_MF_FAT	GO:0004428 inositol or phosphatidylinositol kinase activity	2	0.653595	0.094394	47	17	7918	19.819775	0.999999	0.757767	69.094328
GOTERM_MF_FAT	GO:0016779 nucleotidyltransferase activity	3	0.980392	0.095918	47	90	7918	5.615603	0.999999	0.730410	69.704576
SP_PIR_KEYWORDS	nucleotide-binding	9	2.941176	0.096502	83	743	12980	1.894307	0.999880	0.633421	66.766590

Table 27: Architecture 27

Category	Term	Count	%	PValue	List Total	Pop Hits	Pop Total	Fold Enrichment	Bonferroni	Benjamini	FDR
GOTERM_CC_FAT	GO:0070013 intracellular organelle lumen	26	3.730273	1.912576e-05	91	556	4786	2.459404	0.003913	0.003913	0.024066
GOTERM_CC_FAT	GO:0043233 organelle lumen	26	3.730273	1.912576e-05	91	556	4786	2.459404	0.003913	0.003913	0.024066
GOTERM_CC_FAT	GO:0031974 membrane-enclosed lumen	26	3.730273	3.034333e-05	91	571	4786	2.394796	0.006201	0.003105	0.038179
SP_PIR_KEYWORDS	Chaperone	6	0.860832	0.001288	183	58	12980	7.337479	0.158664	0.158664	1.498201
GOTERM_MF_FAT	GO:0017076 purine nucleotide binding	28	4.017217	0.001697	122	989	7918	1.837458	0.413292	0.413292	2.259150
GOTERM_MF_FAT	GO:0000166 nucleotide binding	32	4.591105	0.001921	122	1206	7918	1.722100	0.453194	0.260536	2.553711
GOTERM_CC_FAT	GO:0031981 nuclear lumen	17	2.439024	0.001998	91	387	4786	2.310305	0.336314	0.127723	2.485194
GOTERM_MF_FAT	GO:0030554 adenyl nucleotide binding	24	3.443329	0.002476	122	812	7918	1.918275	0.540902	0.228561	3.281059
GOTERM_MF_FAT	GO:0001883 purine nucleoside binding	24	3.443329	0.002680	122	817	7918	1.906535	0.569393	0.189934	3.546234
GOTERM_MF_FAT	GO:0001882 nucleoside binding	24	3.443329	0.002988	122	824	7918	1.890339	0.609290	0.171350	3.947287
COG_ONTOLOGY	Transcription	5	0.717360	0.003065	25	33	1237	7.496970	0.030229	0.030229	1.826280
GOTERM_MF_FAT	GO:0004468 lysine N-acetyltransferase activity	4	0.573888	0.003787	122	21	7918	12.362217	0.696187	0.180087	4.977161
GOTERM_MF_FAT	GO:0004402 histone acetyltransferase activity	4	0.573888	0.003787	122	21	7918	12.362217	0.696187	0.180087	4.977161
SP_PIR_KEYWORDS	nucleotide-binding	21	3.012912	0.003837	183	743	12980	2.004722	0.402597	0.227081	4.401454
GOTERM_CC_FAT	GO:0044451 nucleoplasm part	12	1.721664	0.004958	91	240	4786	2.629670	0.639024	0.224879	6.063521
GOTERM_BP_FAT	GO:0006396 RNA processing	12	1.721664	0.005404	116	312	7937	2.631631	0.992939	0.992939	8.115650
GOTERM_MF_FAT	GO:0008080 N-acetyltransferase activity	5	0.717360	0.006012	122	48	7918	6.760587	0.849473	0.237014	7.794229
GOTERM_BP_FAT	GO:0043933 macromolecular complex subunit organization	11	1.578192	0.007428	116	281	7937	2.678457	0.998903	0.966875	10.992969
GOTERM_MF_FAT	GO:0016410 N-acyltransferase activity	5	0.717360	0.007456	122	51	7918	6.362906	0.904634	0.254540	9.580162
SP_PIR_KEYWORDS	atp-binding	17	2.439024	0.008181	183	585	12980	2.061184	0.667400	0.307148	9.170325
GOTERM_BP_FAT	GO:0000381 regulation of alternative nuclear mRNA splicing, via spliceosome	5	0.717360	0.009157	116	57	7937	6.001966	0.999777	0.939352	13.383303
GOTERM_CC_FAT	GO:0005654 nucleoplasm	12	1.721664	0.010250	91	265	4786	2.381588	0.879027	0.344553	12.160945
GOTERM_BP_FAT	GO:0043486 histone exchange	3	0.430416	0.010510	116	11	7937	18.660658	0.999936	0.910558	15.211858
GOTERM_MF_FAT	GO:0016407 acetyltransferase activity	5	0.717360	0.011668	122	58	7918	5.594969	0.974914	0.336013	14.609286
GOTERM_MF_FAT	GO:0032555 purine ribonucleotide binding	24	3.443329	0.011743	122	923	7918	1.687583	0.975501	0.309896	14.695964
GOTERM_MF_FAT	GO:0032553 ribonucleotide binding	24	3.443329	0.011743	122	923	7918	1.687583	0.975501	0.309896	14.695964
KEGG_PATHWAY	dme03018:RNA degradation	5	0.717360	0.011850	40	48	2054	5.348958	0.386603	0.386603	10.389811
GOTERM_BP_FAT	GO:0043044 ATP-dependent chromatin remodeling	3	0.430416	0.012493	116	12	7937	17.105603	0.999990	0.899552	17.827774
GOTERM_BP_FAT	GO:0048024 regulation of nuclear mRNA splicing, via spliceosome	5	0.717360	0.012926	116	63	7937	5.430350	0.999993	0.862201	18.389141
GOTERM_BP_FAT	GO:0050684 regulation of mRNA processing	5	0.717360	0.012926	116	63	7937	5.430350	0.999993	0.862201	18.389141
GOTERM_BP_FAT	GO:0006458 'de novo' protein folding	3	0.430416	0.014626	116	13	7937	15.789788	0.999999	0.853953	20.556408
GOTERM_BP_FAT	GO:0043484 regulation of RNA splicing	5	0.717360	0.015133	116	66	7937	5.183516	0.999999	0.824867	21.193057
GOTERM_BP_FAT	GO:0070647 protein modification by small protein conjugation or removal	5	0.717360	0.015919	116	67	7937	5.106150	1.000000	0.803999	22.168775
GOTERM_BP_FAT	GO:0007010 cytoskeleton organization	14	2.008608	0.016673	116	465	7937	2.060030	1.000000	0.784925	23.095480
GOTERM_MF_FAT	GO:0005524 ATP binding	20	2.869440	0.018287	122	748	7918	1.735338	0.996958	0.409525	21.990506
GOTERM_MF_FAT	GO:0032559 adenyl ribonucleotide binding	20	2.869440	0.018758	122	750	7918	1.730710	0.997384	0.390738	22.493545
GOTERM_BP_FAT	GO:0016573 histone acetylation	3	0.430416	0.019321	116	15	7937	13.684483	1.000000	0.802328	26.267507
GOTERM_CC_FAT	GO:0035267 NuA4 histone acetyltransferase complex	3	0.430416	0.020421	91	12	4786	13.148352	0.985442	0.505861	22.867906
GOTERM_BP_FAT	GO:0051276 chromosome organization	10	1.434720	0.020922	116	282	7937	2.426327	1.000000	0.800202	28.124558
GOTERM_BP_FAT	GO:0006473 protein amino acid acetylation	3	0.430416	0.021875	116	16	7937	12.829203	1.000000	0.788822	29.209781
SP_PIR_KEYWORDS	transit peptide	6	0.860832	0.022103	183	114	12980	3.733103	0.949961	0.527038	23.024734
GOTERM_BP_FAT	GO:0007052 mitotic spindle organization	8	1.147776	0.022334	116	194	7937	2.821543	1.000000	0.771140	29.727181
GOTERM_BP_FAT	GO:0016567 protein ubiquitination	4	0.573888	0.022581	116	42	7937	6.516420	1.000000	0.751344	30.003154
SP_PIR_KEYWORDS	Transcription	12	1.721664	0.022737	183	393	12980	2.165770	0.954128	0.460105	23.607246
SP_PIR_KEYWORDS	helicase	5	0.717360	0.022906	183	77	12980	4.605777	0.955180	0.404001	23.761942
GOTERM_BP_FAT	GO:0022402 cell cycle process	15	2.152080	0.026138	116	547	7937	1.876300	1.000000	0.779753	33.878195
GOTERM_BP_FAT	GO:0007059 chromosome segregation	6	0.860832	0.027198	116	117	7937	3.508842	1.000000	0.772947	34.993642
GOTERM_CC_FAT	GO:0031980 mitochondrial lumen	8	1.147776	0.027433	91	157	4786	2.679919	0.996662	0.557190	29.535081
GOTERM_CC_FAT	GO:0005759 mitochondrial matrix	8	1.147776	0.027433	91	157	4786	2.679919	0.996662	0.557190	29.535081
GOTERM_MF_FAT	GO:0051082 unfolded protein binding	5	0.717360	0.027459	122	75	7918	4.326776	0.999840	0.489582	31.247610
GOTERM_CC_FAT	GO:0034399 nuclear periphery	3	0.430416	0.027480	91	14	4786	11.270016	0.996695	0.510334	29.578149
GOTERM_BP_FAT	GO:0034621 cellular macromolecular complex subunit organization	8	1.147776	0.027754	116	203	7937	2.696450	1.000000	0.760496	35.570527
GOTERM_MF_FAT	GO:0004540 ribonuclease activity	4	0.573888	0.029267	122	44	7918	5.900149	0.999911	0.486354	32.947657

GOTERM_BP_FAT	GO:0043543 protein amino acid acylation	3	0.430416	0.030307	116	19	7937	10.803539	1.000000	0.772473	38.163518
GOTERM_BP_FAT	GO:0007049 cell cycle	16	2.295552	0.031891	116	616	7937	1.777206	1.000000	0.772626	39.722175
GOTERM_BP_FAT	GO:0032446 protein modification by small protein conjugation	4	0.573888	0.031971	116	48	7937	5.701868	1.000000	0.756892	39.800398
GOTERM_BP_FAT	GO:0007015 actin filament organization	5	0.717360	0.032045	116	83	7937	4.121832	1.000000	0.741563	39.871379
GOTERM_BP_FAT	GO:0006350 transcription	13	1.865136	0.033401	116	459	7937	1.937890	1.000000	0.740760	41.174120
GOTERM_BP_FAT	GO:0070271 protein complex biogenesis	7	1.004304	0.034063	116	167	7937	2.868005	1.000000	0.732827	41.800607
GOTERM_BP_FAT	GO:0006461 protein complex assembly	7	1.004304	0.034063	116	167	7937	2.868005	1.000000	0.732827	41.800607
GOTERM_BP_FAT	GO:0000278 mitotic cell cycle	11	1.578192	0.034402	116	358	7937	2.102365	1.000000	0.721926	42.118018
SP_PIR_KEYWORDS	nucleus	20	2.869440	0.035809	183	869	12980	1.632427	0.992452	0.502452	34.750683
GOTERM_MF_FAT	GO:0004842 ubiquitin-protein ligase activity	5	0.717360	0.037868	122	83	7918	3.909737	0.999995	0.554298	40.515162
GOTERM_CC_FAT	GO:0043232 intracellular non-membrane-bounded organelle	26	3.730273	0.039058	91	943	4786	1.450083	0.999716	0.596464	39.430825
GOTERM_CC_FAT	GO:0043228 non-membrane-bounded organelle	26	3.730273	0.039058	91	943	4786	1.450083	0.999716	0.596464	39.430825
GOTERM_CC_FAT	GO:0043189 H4/H2A histone acetyltransferase complex	3	0.430416	0.039604	91	17	4786	9.281189	0.999747	0.563250	39.862628
GOTERM_BP_FAT	GO:0006259 DNA metabolic process	8	1.147776	0.041143	116	221	7937	2.476829	1.000000	0.771662	48.117471
GOTERM_MF_FAT	GO:0016887 ATPase activity	11	1.578192	0.043257	122	353	7918	2.022431	0.999999	0.580134	44.845023
GOTERM_BP_FAT	GO:0034470 ncRNA processing	5	0.717360	0.044189	116	92	7937	3.718609	1.000000	0.783451	50.632755
GOTERM_BP_FAT	GO:0007051 spindle organization	8	1.147776	0.044606	116	225	7937	2.432797	1.000000	0.774526	50.968267
GOTERM_BP_FAT	GO:0006413 translational initiation	4	0.573888	0.045120	116	55	7937	4.976176	1.000000	0.766634	51.378640
SP_PIR_KEYWORDS	transcription regulation	11	1.578192	0.046175	183	387	12980	2.016069	0.998226	0.546993	42.506873
UP_SEQ_FEATURE	transit peptide:Mitochondrion	6	0.860832	0.046931	53	114	2975	2.954320	0.999225	0.999225	43.636616
GOTERM_MF_FAT	GO:0019787 small conjugating protein ligase activity	5	0.717360	0.046974	122	89	7918	3.646160	1.000000	0.588802	47.659939
GOTERM_BP_FAT	GO:0032774 RNA biosynthetic process	6	0.860832	0.047215	116	136	7937	3.018636	1.000000	0.770887	53.017887
INTERPRO	IPR003151:PIK-related kinase, FAT	2	0.286944	0.047487	165	3	10196	41.195960	1.000000	1.000000	48.901841
GOTERM_BP_FAT	GO:0009057 macromolecule catabolic process	9	1.291248	0.047493	116	277	7937	2.223111	1.000000	0.761792	53.231503
GOTERM_MF_FAT	GO:0016779 nucleotidyltransferase activity	5	0.717360	0.048600	122	90	7918	3.605647	1.000000	0.580671	48.849024
GOTERM_BP_FAT	GO:0006261 DNA-dependent DNA replication	4	0.573888	0.049300	116	57	7937	4.801573	1.000000	0.764022	54.598230
SP_PIR_KEYWORDS	ligase	7	1.004304	0.054400	183	193	12980	2.572553	0.999444	0.565178	48.051164
GOTERM_BP_FAT	GO:0030036 actin cytoskeleton organization	6	0.860832	0.056329	116	143	7937	2.870871	1.000000	0.799273	59.567289
GOTERM_BP_FAT	GO:0030029 actin filament-based process	6	0.860832	0.057709	116	144	7937	2.850934	1.000000	0.797681	60.480716
GOTERM_MF_FAT	GO:0003743 translation initiation factor activity	4	0.573888	0.058566	122	58	7918	4.475975	1.000000	0.631153	55.606321
SP_PIR_KEYWORDS	chromatin regulator	4	0.573888	0.060694	183	64	12980	4.433060	0.999773	0.567868	51.958285
GOTERM_MF_FAT	GO:0050662 coenzyme binding	6	0.860832	0.061403	122	139	7918	2.801510	1.000000	0.630239	57.373439
GOTERM_BP_FAT	GO:0042752 regulation of circadian rhythm	3	0.430416	0.061609	116	28	7937	7.330973	1.000000	0.809971	62.959896
GOTERM_BP_FAT	GO:0070727 cellular macromolecule localization	8	1.147776	0.063601	116	244	7937	2.243358	1.000000	0.811452	64.168995
GOTERM_MF_FAT	GO:0048037 cofactor binding	7	1.004304	0.063730	122	185	7918	2.455738	1.000000	0.626424	58.773605
GOTERM_BP_FAT	GO:0065003 macromolecular complex assembly	8	1.147776	0.065850	116	246	7937	2.225119	1.000000	0.814128	65.489499
GOTERM_MF_FAT	GO:0050660 FAD binding	4	0.573888	0.066134	122	61	7918	4.255845	1.000000	0.623400	60.175423
GOTERM_BP_FAT	GO:0000226 microtubule cytoskeleton organization	9	1.291248	0.067114	116	298	7937	2.066449	1.000000	0.811938	66.211643
SP_PIR_KEYWORDS	ubiquinone biosynthesis	2	0.286944	0.068179	183	5	12980	28.371585	0.999922	0.576930	56.254506
GOTERM_BP_FAT	GO:0006401 RNA catabolic process	3	0.430416	0.069600	116	30	7937	6.842241	1.000000	0.815607	67.591068
SMART	SM00504:Ubox	2	0.286944	0.072461	73	5	4824	26.432877	0.994429	0.994429	53.983886
GOTERM_MF_FAT	GO:0004526 ribonuclease P activity	2	0.286944	0.074126	122	5	7918	25.960656	1.000000	0.650572	64.525073
GOTERM_BP_FAT	GO:0016458 gene silencing	6	0.860832	0.074160	116	155	7937	2.648610	1.000000	0.828072	69.984907
GOTERM_MF_FAT	GO:0032183 SUMO binding	4	0.573888	0.076883	122	65	7918	3.993947	1.000000	0.648893	65.920425
GOTERM_CC_FAT	GO:0005635 nuclear envelope	5	0.717360	0.077593	91	86	4786	3.057756	1.000000	0.778035	63.812043
GOTERM_BP_FAT	GO:0010629 negative regulation of gene expression	8	1.147776	0.077813	116	256	7937	2.138200	1.000000	0.835668	71.782112
INTERPRO	IPR003152:PIK-related kinase, FATC	2	0.286944	0.077893	165	5	10196	24.717576	1.000000	1.000000	67.344173
INTERPRO	IPR014009:PIK-related kinase	2	0.286944	0.077893	165	5	10196	24.717576	1.000000	1.000000	67.344173
INTERPRO	IPR003613:U box domain	2	0.286944	0.077893	165	5	10196	24.717576	1.000000	1.000000	67.344173
GOTERM_MF_FAT	GO:0032182 small conjugating protein binding	4	0.573888	0.079684	122	66	7918	3.933433	1.000000	0.647591	67.285805
GOTERM_CC_FAT	GO:0044429 mitochondrial part	13	1.865136	0.081856	91	411	4786	1.663538	1.000000	0.767515	65.861267
UP_SEQ_FEATURE	domain:FAT	2	0.286944	0.084448	53	5	2975	22.452830	0.999998	0.998602	65.088887
UP_SEQ_FEATURE	domain:FATC	2	0.286944	0.084448	53	5	2975	22.452830	0.999998	0.998602	65.088887
UP_SEQ_FEATURE	domain:PI3K/PI4K	2	0.286944	0.084448	53	5	2975	22.452830	0.999998	0.998602	65.088887
GOTERM_MF_FAT	GO:0004386 helicase activity	5	0.717360	0.085266	122	109	7918	2.977139	1.000000	0.659153	69.857158
GOTERM_BP_FAT	GO:0006457 protein folding	5	0.717360	0.085353	116	115	7937	2.974888	1.000000	0.856516	75.178087

GOTERM_CC_FAT	GO:0000123 histone acetyltransferase complex	3	0.430416	0.090664	91	27	4786	5.843712	1.000000	0.776583	69.761398
GOTERM_BP_FAT	GO:0006091 generation of precursor metabolites and energy	7	1.004304	0.094785	116	217	7937	2.207175	1.000000	0.879572	78.888127
GOTERM_BP_FAT	GO:0016485 protein processing	3	0.430416	0.095384	116	36	7937	5.701868	1.000000	0.875364	79.105432
UP_SEQ_FEATURE	domain:Helicase C-terminal	3	0.430416	0.095581	53	30	2975	5.613208	1.000000	0.993192	69.829295
UP_SEQ_FEATURE	domain:Helicase ATP-binding	3	0.430416	0.095581	53	30	2975	5.613208	1.000000	0.993192	69.829295
GOTERM_MF_FAT	GO:0016881 acid-amino acid ligase activity	5	0.717360	0.096663	122	114	7918	2.846563	1.000000	0.693416	74.536483
GOTERM_BP_FAT	GO:0016071 mRNA metabolic process	7	1.004304	0.097976	116	219	7937	2.187018	1.000000	0.876850	80.020996
GOTERM_BP_FAT	GO:0051604 protein maturation	3	0.430416	0.099915	116	37	7937	5.547763	1.000000	0.876509	80.681370

Table 28: Architecture 28

Category	Term	Count	%	PValue	List Total	Pop Hits	Pop Total	Fold Enrichment	Bonferroni	Benjamini	FDR
SP_PIR_KEYWORDS	coiled coil	9	1.825558	9.019254e-04	122	215	12980	4.453679	0.087106	0.087106	0.998389
GOTERM_BP_FAT	GO:0006518 peptide metabolic process	3	0.608519	0.003784	69	11	7937	31.371542	0.951479	0.951479	5.652281
GOTERM_BP_FAT	GO:0034984 cellular response to DNA damage stimulus	5	1.014199	0.005929	69	85	7937	6.766411	0.991309	0.906775	8.721254
GOTERM_MF_FAT	GO:0017076 purine nucleotide binding	17	3.448276	0.009598	70	989	7918	1.944330	0.860190	0.860190	11.420989
GOTERM_BP_FAT	GO:0043933 macromolecular complex subunit organization	8	1.622718	0.009910	69	281	7937	3.274847	0.999646	0.929286	14.171708
GOTERM_BP_FAT	GO:0006366 transcription from RNA polymerase II promoter	5	1.014199	0.010083	69	99	7937	5.809545	0.999692	0.867574	14.401825
GOTERM_CC_FAT	GO:0044451 nucleoplasm part	8	1.622718	0.011515	51	240	4786	3.128105	0.884002	0.884002	13.362475
GOTERM_MF_FAT	GO:0032553 ribonucleotide binding	16	3.245436	0.011812	70	923	7918	1.960811	0.911441	0.702411	13.879367
GOTERM_MF_FAT	GO:0032555 purine ribonucleotide binding	16	3.245436	0.011812	70	923	7918	1.960811	0.911441	0.702411	13.879367
GOTERM_BP_FAT	GO:0006461 protein complex assembly	6	1.217039	0.013940	69	167	7937	4.132778	0.999986	0.893595	19.379465
GOTERM_BP_FAT	GO:0070271 protein complex biogenesis	6	1.217039	0.013940	69	167	7937	4.132778	0.999986	0.893595	19.379465
GOTERM_BP_FAT	GO:0051188 cofactor biosynthetic process	4	0.811359	0.014535	69	60	7937	7.668599	0.999992	0.857353	20.122880
SP_PIR_KEYWORDS	Chaperone	4	0.811359	0.016807	122	58	12980	7.337479	0.819486	0.575131	17.178793
GOTERM_CC_FAT	GO:0031981 nuclear lumen	10	2.028398	0.017196	51	387	4786	2.424887	0.960293	0.800734	19.331360
GOTERM_MF_FAT	GO:0015459 potassium channel regulator activity	2	0.405680	0.017354	70	2	7918	113.114286	0.971879	0.695906	19.758767
GOTERM_BP_FAT	GO:0007281 germ cell development	7	1.419878	0.017414	69	243	7937	3.313592	0.999999	0.865022	23.628917
GOTERM_BP_FAT	GO:0006367 transcription initiation from RNA polymerase II promoter	4	0.811359	0.018001	69	65	7937	7.078707	0.999999	0.836673	24.326958
GOTERM_BP_FAT	GO:0065003 macromolecular complex assembly	7	1.419878	0.018389	69	246	7937	3.273183	1.000000	0.807122	24.784328
GOTERM_CC_FAT	GO:0005654 nucleoplasm	8	1.622718	0.019046	51	265	4786	2.833000	0.972034	0.696463	21.192356
GOTERM_MF_FAT	GO:0030554 adenyl nucleotide binding	14	2.839757	0.021562	70	812	7918	1.950246	0.988285	0.671006	23.974803
GOTERM_MF_FAT	GO:0001883 purine nucleoside binding	14	2.839757	0.022570	70	817	7918	1.938311	0.990505	0.605995	24.953042
GOTERM_MF_FAT	GO:0003702 RNA polymerase II transcription factor activity	7	1.419878	0.024020	70	257	7918	3.080934	0.992986	0.562486	26.341308
GOTERM_MF_FAT	GO:0001882 nucleoside binding	14	2.839757	0.024039	70	824	7918	1.921845	0.993014	0.507926	26.359473
GOTERM_BP_FAT	GO:0006352 transcription initiation	4	0.811359	0.024426	69	73	7937	6.302958	1.000000	0.861012	31.577229
GOTERM_CC_FAT	GO:0043233 organelle lumen	12	2.434077	0.025655	51	556	4786	2.025391	0.992046	0.701359	27.521334
GOTERM_CC_FAT	GO:0070013 intracellular organelle lumen	12	2.434077	0.025655	51	556	4786	2.025391	0.992046	0.701359	27.521334
GOTERM_MF_FAT	GO:0004357 glutamate-cysteine ligase activity	2	0.405680	0.025919	70	3	7918	75.409524	0.995286	0.488113	28.123438
SP_PIR_KEYWORDS	electron transport	3	0.608519	0.025987	122	27	12980	11.821494	0.930011	0.587894	25.383129
GOTERM_MF_FAT	GO:0005524 ATP binding	13	2.636917	0.026807	70	748	7918	1.965890	0.996086	0.459849	28.942562
GOTERM_BP_FAT	GO:0006351 transcription, DNA-dependent	5	1.014199	0.026903	69	133	7937	4.324398	1.000000	0.861710	34.195091
GOTERM_MF_FAT	GO:0032559 adenyl ribonucleotide binding	13	2.636917	0.027304	70	750	7918	1.960648	0.996473	0.431493	29.397710
GOTERM_BP_FAT	GO:0032774 RNA biosynthetic process	5	1.014199	0.028891	69	136	7937	4.229007	1.000000	0.857666	36.228774
GOTERM_CC_FAT	GO:0031974 membrane-enclosed lumen	12	2.434077	0.030573	51	571	4786	1.972185	0.996897	0.684963	31.924556
GOTERM_CC_FAT	GO:0017109 glutamate-cysteine ligase complex	2	0.405680	0.031022	51	3	4786	62.562092	0.997153	0.623524	32.313559
SP_PIR_KEYWORDS	nucleus	15	3.042596	0.031400	122	869	12980	1.836481	0.960133	0.553159	29.866272
GOTERM_BP_FAT	GO:0006749 glutathione metabolic process	2	0.405680	0.033838	69	4	7937	57.514493	1.000000	0.879138	41.035625
GOTERM_BP_FAT	GO:0006750 glutathione biosynthetic process	2	0.405680	0.033838	69	4	7937	57.514493	1.000000	0.879138	41.035625
GOTERM_BP_FAT	GO:0006790 sulfur metabolic process	3	0.608519	0.039734	69	37	7937	9.326675	1.000000	0.900845	46.321860
SP_PIR_KEYWORDS	phosphoprotein	14	2.839757	0.040162	122	815	12980	1.827617	0.984078	0.563080	36.607118
GOTERM_BP_FAT	GO:0006259 DNA metabolic process	6	1.217039	0.040442	69	221	7937	3.122959	1.000000	0.888781	46.926207
GOTERM_BP_FAT	GO:0048489 synaptic vesicle transport	4	0.811359	0.041664	69	90	7937	5.112399	1.000000	0.880269	47.953282
GOTERM_BP_FAT	GO:0043043 peptide biosynthetic process	2	0.405680	0.042120	69	5	7937	46.011594	1.000000	0.867346	48.332318
GOTERM_MF_FAT	GO:0016251 general RNA polymerase II transcription factor activity	4	0.811359	0.042275	70	89	7918	5.083788	0.999851	0.551146	41.908537
GOTERM_MF_FAT	GO:0016247 channel regulator activity	2	0.405680	0.042829	70	5	7918	45.245714	0.999868	0.524863	42.330194
GOTERM_CC_FAT	GO:0005938 cell cortex	4	0.811359	0.042841	51	75	4786	5.004967	0.999710	0.687591	41.857841
GOTERM_CC_FAT	GO:0005789 endoplasmic reticulum membrane	4	0.811359	0.042841	51	75	4786	5.004967	0.999710	0.687591	41.857841
GOTERM_CC_FAT	GO:0042175 nuclear envelope-endoplasmic reticulum network	4	0.811359	0.045746	51	77	4786	4.874968	0.999835	0.663345	44.006293
GOTERM_MF_FAT	GO:0005516 calmodulin binding	3	0.608519	0.047204	70	40	7918	8.483571	0.999948	0.531770	45.558299
GOTERM_CC_FAT	GO:0005667 transcription factor complex	4	0.811359	0.050284	51	80	4786	4.692157	0.999932	0.655703	47.216515
GOTERM_MF_FAT	GO:0003906 DNA-(apurinic or apyrimidinic site) lyase activity	2	0.405680	0.051176	70	6	7918	37.704762	0.999978	0.534881	48.344093
GOTERM_MF_FAT	GO:0000166 nucleotide binding	17	3.448276	0.052130	70	1206	7918	1.594480	0.999982	0.517185	48.993828

GOTERM_BP_FAT	GO:0009108 coenzyme biosynthetic process	3	0.608519	0.052147	69	43	7937	8.025278	1.000000	0.906920	56.036466
GOTERM_BP_FAT	GO:0051726 regulation of cell cycle	5	1.014199	0.053548	69	166	7937	3.464728	1.000000	0.900883	57.022992
GOTERM_BP_FAT	GO:0007269 neurotransmitter secretion	4	0.811359	0.056686	69	102	7937	4.510941	1.000000	0.902549	59.158590
GOTERM_BP_FAT	GO:0006281 DNA repair	4	0.811359	0.056686	69	102	7937	4.510941	1.000000	0.902549	59.158590
GOTERM_BP_FAT	GO:0003001 generation of a signal involved in cell-cell signaling	4	0.811359	0.058040	69	103	7937	4.467145	1.000000	0.896907	60.049095
GOTERM_BP_FAT	GO:0030182 neuron differentiation	8	1.622718	0.059696	69	409	7937	2.249956	1.000000	0.892757	61.113181
GOTERM_BP_FAT	GO:0051235 maintenance of location	3	0.608519	0.061089	69	47	7937	7.342276	1.000000	0.887750	61.987842
GOTERM_BP_FAT	GO:0051186 cofactor metabolic process	4	0.811359	0.062195	69	106	7937	4.340716	1.000000	0.881765	62.669028
GOTERM_BP_FAT	GO:0048610 reproductive cellular process	9	1.825558	0.062639	69	500	7937	2.070522	1.000000	0.873157	62.939480
GOTERM_BP_FAT	GO:0003006 reproductive developmental process	9	1.825558	0.066237	69	506	7937	2.045970	1.000000	0.877961	65.063059
GOTERM_BP_FAT	GO:0001505 regulation of neurotransmitter levels	4	0.811359	0.067945	69	110	7937	4.182872	1.000000	0.875022	66.030960
GOTERM_BP_FAT	GO:0045464 R8 cell fate specification	2	0.405680	0.074553	69	9	7937	25.561997	1.000000	0.890095	69.544663
GOTERM_BP_FAT	GO:0006974 response to DNA damage stimulus	4	0.811359	0.075462	69	115	7937	4.001008	1.000000	0.884562	70.000205
GOTERM_MF_FAT	GO:0016881 acid-amino acid ligase activity	4	0.811359	0.076943	70	114	7918	3.968922	1.000000	0.639700	63.460446
GOTERM_BP_FAT	GO:0045941 positive regulation of transcription	4	0.811359	0.077008	69	116	7937	3.966517	1.000000	0.881351	70.760978
GOTERM_BP_FAT	GO:0016079 synaptic vesicle exocytosis	3	0.608519	0.077872	69	54	7937	6.390499	1.000000	0.875934	71.178305
GOTERM_BP_FAT	GO:0016358 dendrite development	4	0.811359	0.078568	69	117	7937	3.932615	1.000000	0.870043	71.510352
GOTERM_BP_FAT	GO:0048813 dendrite morphogenesis	4	0.811359	0.078568	69	117	7937	3.932615	1.000000	0.870043	71.510352
GOTERM_BP_FAT	GO:0010628 positive regulation of gene expression	4	0.811359	0.078568	69	117	7937	3.932615	1.000000	0.870043	71.510352
GOTERM_BP_FAT	GO:0032940 secretion by cell	4	0.811359	0.080143	69	118	7937	3.899288	1.000000	0.867354	72.248197
GOTERM_CC_FAT	GO:0016023 cytoplasmic membrane-bounded vesicle	4	0.811359	0.081837	51	98	4786	3.830332	1.000000	0.795680	65.264997
GOTERM_BP_FAT	GO:0006357 regulation of transcription from RNA polymerase II promoter	5	1.014199	0.081898	69	192	7937	2.995546	1.000000	0.865407	73.049993
GOTERM_BP_FAT	GO:0051173 positive regulation of nitrogen compound metabolic process	4	0.811359	0.084948	69	121	7937	3.802611	1.000000	0.867882	74.391442
GOTERM_BP_FAT	GO:0045935 positive regulation of nucleobase, nucleoside, nucleotide and nucleic acid metabolic process	4	0.811359	0.084948	69	121	7937	3.802611	1.000000	0.867882	74.391442
SP_PIR_KEYWORDS	nucleotide-binding	12	2.434077	0.086156	122	743	12980	1.718333	0.999888	0.780544	63.280823
SP_PIR_KEYWORDS	transcription initiation	2	0.405680	0.089435	122	10	12980	21.278689	0.999922	0.741230	64.719796
GOTERM_CC_FAT	GO:0031988 membrane-bounded vesicle	4	0.811359	0.089790	51	102	4786	3.680123	1.000000	0.796240	68.813274
GOTERM_BP_FAT	GO:0043704 photoreceptor cell fate specification	2	0.405680	0.090362	69	11	7937	20.914361	1.000000	0.877466	76.620134
GOTERM_BP_FAT	GO:0048665 neuron fate specification	2	0.405680	0.090362	69	11	7937	20.914361	1.000000	0.877466	76.620134
GOTERM_BP_FAT	GO:0010212 response to ionizing radiation	2	0.405680	0.090362	69	11	7937	20.914361	1.000000	0.877466	76.620134
GOTERM_CC_FAT	GO:0005794 Golgi apparatus	5	1.014199	0.090733	51	164	4786	2.861071	1.000000	0.771060	69.210681
GOTERM_BP_FAT	GO:0030030 cell projection organization	7	1.419878	0.091535	69	365	7937	2.206035	1.000000	0.873873	77.078718
GOTERM_BP_FAT	GO:0046903 secretion	4	0.811359	0.091544	69	125	7937	3.680928	1.000000	0.866837	77.082028
GOTERM_BP_FAT	GO:0006836 neurotransmitter transport	4	0.811359	0.093225	69	126	7937	3.651714	1.000000	0.864988	77.724482
GOTERM_BP_FAT	GO:0034621 cellular macromolecular complex subunit organization	5	1.014199	0.095757	69	203	7937	2.833226	1.000000	0.865759	78.660014
GOTERM_CC_FAT	GO:0031410 cytoplasmic vesicle	4	0.811359	0.095964	51	105	4786	3.574977	1.000000	0.763886	71.334010
GOTERM_BP_FAT	GO:0045184 establishment of protein localization	6	1.217039	0.096598	69	285	7937	2.421663	1.000000	0.861549	78.962372
GOTERM_BP_FAT	GO:0006350 transcription	8	1.622718	0.096606	69	459	7937	2.004862	1.000000	0.854899	78.965143
GOTERM_BP_FAT	GO:0048812 neuron projection morphogenesis	6	1.217039	0.097691	69	286	7937	2.413196	1.000000	0.851585	79.349610
GOTERM_BP_FAT	GO:0010557 positive regulation of macromolecule biosynthetic process	4	0.811359	0.098348	69	129	7937	3.566790	1.000000	0.847042	79.579041
GOTERM_BP_FAT	GO:0031175 neuron projection development	6	1.217039	0.098790	69	287	7937	2.404787	1.000000	0.841908	79.732378
GOTERM_BP_FAT	GO:0048667 cell morphogenesis involved in neuron differentiation	6	1.217039	0.099896	69	288	7937	2.396437	1.000000	0.838907	80.110662

Table 29: Architecture 29

Category	Term	Count	%	PValue	List Total	Pop Hits	Pop Total	Fold Enrichment	Bonferroni	Benjamini	FDR
GOTERM_CC_FAT	GO:0044429 mitochondrial part	33	1.696658	6.217632e-06	165	411	4786	2.328954	0.001510	0.001510	0.008041
GOTERM_CC_FAT	GO:0005739 mitochondrion	39	2.005141	2.985799e-05	165	571	4786	1.981149	0.007229	0.003621	0.038608
GOTERM_CC_FAT	GO:0031974 membrane-enclosed lumen	39	2.005141	2.985799e-05	165	571	4786	1.981149	0.007229	0.003621	0.038608
GOTERM_CC_FAT	GO:0043233 organelle lumen	38	1.953728	3.917359e-05	165	556	4786	1.982429	0.009474	0.003168	0.050651
GOTERM_CC_FAT	GO:0070013 intracellular organelle lumen	38	1.953728	3.917359e-05	165	556	4786	1.982429	0.009474	0.003168	0.050651
GOTERM_BP_FAT	GO:0044265 cellular macromolecule catabolic process	20	1.028278	1.320348e-04	260	225	7937	2.713504	0.153048	0.153048	0.214494
GOTERM_BP_FAT	GO:0016192 vesicle-mediated transport	28	1.439589	1.727181e-04	260	391	7937	2.186071	0.195310	0.102955	0.280498
GOTERM_BP_FAT	GO:0010608 post-transcriptional regulation of gene expression	14	0.719794	2.240849e-04	260	126	7937	3.391880	0.245674	0.089696	0.363776
GOTERM_BP_FAT	GO:0009057 macromolecule catabolic process	22	1.131105	2.713581e-04	260	277	7937	2.424521	0.289236	0.081813	0.440360
GOTERM_CC_FAT	GO:0019866 organelle inner membrane	19	0.976864	3.871724e-04	165	217	4786	2.539701	0.089809	0.023251	0.499571
GOTERM_BP_FAT	GO:0006396 RNA processing	23	1.182519	5.271013e-04	260	312	7937	2.250382	0.484835	0.124232	0.853714
GOTERM_BP_FAT	GO:0016071 mRNA metabolic process	18	0.925450	7.892407e-04	260	219	7937	2.509062	0.629632	0.152566	1.275734
GOTERM_CC_FAT	GO:0005761 mitochondrial ribosome	10	0.514139	8.531284e-04	165	74	4786	3.919738	0.187304	0.040631	1.097744
GOTERM_CC_FAT	GO:0000313 organellar ribosome	10	0.514139	8.531284e-04	165	74	4786	3.919738	0.187304	0.040631	1.097744
GOTERM_CC_FAT	GO:0031967 organelle envelope	24	1.233933	0.001331	165	344	4786	2.023679	0.276555	0.052525	1.708197
GOTERM_CC_FAT	GO:0031975 envelope	24	1.233933	0.001384	165	345	4786	2.017813	0.285835	0.046954	1.775713
GOTERM_MF_FAT	GO:0000166 nucleotide binding	65	3.341902	0.001494	295	1206	7918	1.446637	0.497314	0.497314	2.105411
SP_PIR_KEYWORDS	nucleotide-binding	42	2.159383	0.002249	452	743	12980	1.623292	0.339183	0.339183	2.745097
GOTERM_BP_FAT	GO:0006259 DNA metabolic process	17	0.874036	0.002293	260	221	7937	2.348225	0.944329	0.338082	3.664742
GOTERM_CC_FAT	GO:0005759 mitochondrial matrix	14	0.719794	0.002595	165	157	4786	2.586528	0.468099	0.075879	3.304060
GOTERM_CC_FAT	GO:0031980 mitochondrial lumen	14	0.719794	0.002595	165	157	4786	2.586528	0.468099	0.075879	3.304060
GOTERM_CC_FAT	GO:0005740 mitochondrial envelope	19	0.976864	0.002878	165	258	4786	2.136105	0.503581	0.074864	3.658704
GOTERM_BP_FAT	GO:0046907 intracellular transport	21	1.079692	0.003070	260	313	7937	2.048132	0.979106	0.383401	4.877422
GOTERM_CC_FAT	GO:0031090 organelle membrane	27	1.388175	0.003524	165	438	4786	1.788045	0.575931	0.082209	4.463025
GOTERM_CC_FAT	GO:0005654 nucleoplasm	19	0.976864	0.003841	165	265	4786	2.079680	0.607495	0.081505	4.855502
GOTERM_BP_FAT	GO:0044087 regulation of cellular component biogenesis	9	0.462725	0.004391	260	80	7937	3.434279	0.996056	0.459394	6.905587
GOTERM_CC_FAT	GO:0030529 ribonucleoprotein complex	24	1.233933	0.004653	165	379	4786	1.836795	0.678069	0.090128	5.853923
GOTERM_BP_FAT	GO:0051276 chromosome organization	19	0.976864	0.004911	260	282	7937	2.056779	0.997957	0.461693	7.693681
GOTERM_CC_FAT	GO:0000315 organellar large ribosomal subunit	7	0.359897	0.004949	165	47	4786	4.320052	0.700476	0.088565	6.214711
GOTERM_CC_FAT	GO:0005762 mitochondrial large ribosomal subunit	7	0.359897	0.004949	165	47	4786	4.320052	0.700476	0.088565	6.214711
GOTERM_BP_FAT	GO:0006281 DNA repair	10	0.514139	0.005933	260	102	7937	2.992836	0.999439	0.493661	9.223418
GOTERM_CC_FAT	GO:0031981 nuclear lumen	24	1.233933	0.006005	165	387	4786	1.798825	0.768599	0.099264	7.493894
GOTERM_CC_FAT	GO:0005753 mitochondrial proton-transporting ATP synthase complex	5	0.257069	0.007070	165	23	4786	6.305665	0.821663	0.108579	8.767462
GOTERM_CC_FAT	GO:0031966 mitochondrial membrane	17	0.874036	0.007880	165	241	4786	2.046071	0.853745	0.113213	9.725392
GOTERM_CC_FAT	GO:0005743 mitochondrial inner membrane	15	0.771208	0.008143	165	200	4786	2.175455	0.862873	0.110302	10.034463
GOTERM_MF_FAT	GO:0008641 small protein activating enzyme activity	4	0.205656	0.008687	295	12	7918	8.946893	0.981927	0.865563	11.676552
GOTERM_BP_FAT	GO:0008104 protein localization	23	1.182519	0.008904	260	392	7937	1.791120	0.999987	0.608450	13.536356
GOTERM_MF_FAT	GO:0032553 ribonucleotide binding	49	2.519280	0.009204	295	923	7918	1.424911	0.985785	0.757759	12.330320
GOTERM_MF_FAT	GO:0032555 purine ribonucleotide binding	49	2.519280	0.009204	295	923	7918	1.424911	0.985785	0.757759	12.330320
GOTERM_CC_FAT	GO:0000276 mitochondrial proton-transporting ATP synthase complex, coupling factor F(o)	4	0.205656	0.010885	165	14	4786	8.287446	0.930024	0.137358	13.198703
GOTERM_BP_FAT	GO:0034660 ncRNA metabolic process	11	0.565553	0.011498	260	133	7937	2.524783	1.000000	0.673437	17.143930
GOTERM_BP_FAT	GO:0006402 mRNA catabolic process	5	0.257069	0.012240	260	28	7937	5.451236	1.000000	0.669336	18.149303
GOTERM_BP_FAT	GO:0006974 response to DNA damage stimulus	10	0.514139	0.012654	260	115	7937	2.654515	1.000000	0.656317	18.705395
SP_PIR_KEYWORDS	atp-binding	32	1.645244	0.012771	452	585	12980	1.570834	0.906053	0.693493	14.692055
GOTERM_MF_FAT	GO:0017076 purine nucleotide binding	51	2.622108	0.013178	295	989	7918	1.384100	0.997762	0.782485	17.203368
GOTERM_BP_FAT	GO:0030163 protein catabolic process	14	0.719794	0.013760	260	200	7937	2.136885	1.000000	0.663583	20.173620
INTERPRO	IPR000086:NUDIX hydrolase domain	4	0.205656	0.013844	413	13	10196	7.596200	0.999954	0.999954	19.011809
GOTERM_CC_FAT	GO:0045259 proton-transporting ATP synthase complex	5	0.257069	0.014317	165	28	4786	5.179654	0.969928	0.168421	17.013929
GOTERM_BP_FAT	GO:0009994 oocyte differentiation	11	0.565553	0.014633	260	138	7937	2.433305	1.000000	0.664057	21.314237
GOTERM_BP_FAT	GO:0032940 secretion by cell	10	0.514139	0.014795	260	118	7937	2.587027	1.000000	0.647168	21.525421
GOTERM_BP_FAT	GO:0048278 vesicle docking	5	0.257069	0.015573	260	30	7937	5.087821	1.000000	0.646264	22.526438
GOTERM_BP_FAT	GO:0006904 vesicle docking during exocytosis	5	0.257069	0.015573	260	30	7937	5.087821	1.000000	0.646264	22.526438
GOTERM_BP_FAT	GO:0022406 membrane docking	5	0.257069	0.015573	260	30	7937	5.087821	1.000000	0.646264	22.526438

GOTERM_BP_FAT	GO:0006401 RNA catabolic process	5	0.257069	0.015573	260	30	7937	5.087821	1.000000	0.646264	22.526438
GOTERM_CC_FAT	GO:0044455 mitochondrial membrane part	11	0.565553	0.015963	165	134	4786	2.381095	0.979964	0.177586	18.788173
GOTERM_BP_FAT	GO:0016044 membrane organization	19	0.976864	0.016459	260	319	7937	1.818218	1.000000	0.647909	23.652310
GOTERM_BP_FAT	GO:0044257 cellular protein catabolic process	13	0.668380	0.016477	260	183	7937	2.168579	1.000000	0.630387	23.675935
GOTERM_BP_FAT	GO:0051603 proteolysis involved in cellular protein catabolic process	13	0.668380	0.016477	260	183	7937	2.168579	1.000000	0.630387	23.675935
GOTERM_BP_FAT	GO:0000184 nuclear-transcribed mRNA catabolic process, nonsense-mediated decay	4	0.205656	0.016641	260	17	7937	7.182805	1.000000	0.616935	23.881679
SMART	SM00271:DnaJ	6	0.308483	0.016880	180	41	4824	3.921951	0.910846	0.910846	18.238567
GOTERM_BP_FAT	GO:0007010 cytoskeleton organization	25	1.285347	0.016918	260	465	7937	1.641232	1.000000	0.606721	24.229538
INTERPRO	IPR014729:Rossmann-like alpha/beta/alpha sandwich fold	6	0.308483	0.017598	413	38	10196	3.898050	0.999997	0.998264	23.551798
GOTERM_MF_FAT	GO:0005524 ATP binding	40	2.056555	0.017791	295	748	7918	1.435330	0.999741	0.808235	22.544642
GOTERM_MF_FAT	GO:0032559 adenyl ribonucleotide binding	40	2.056555	0.018541	295	750	7918	1.431503	0.999818	0.761847	23.382723
GOTERM_CC_FAT	GO:0005637 nuclear inner membrane	4	0.205656	0.018862	165	17	4786	6.824955	0.990218	0.197757	21.828802
GOTERM_BP_FAT	GO:0007034 vacuolar transport	4	0.205656	0.019497	260	18	7937	6.783761	1.000000	0.643719	27.397901
GOTERM_BP_FAT	GO:0031647 regulation of protein stability	4	0.205656	0.019497	260	18	7937	6.783761	1.000000	0.643719	27.397901
GOTERM_BP_FAT	GO:0046903 secretion	10	0.514139	0.020817	260	125	7937	2.442154	1.000000	0.653042	28.971268
GOTERM_BP_FAT	GO:0006897 endocytosis	16	0.822622	0.021553	260	258	7937	1.893143	1.000000	0.651545	29.835162
GOTERM_BP_FAT	GO:0010324 membrane invagination	16	0.822622	0.021553	260	258	7937	1.893143	1.000000	0.651545	29.835162
GOTERM_BP_FAT	GO:0006887 exocytosis	7	0.359897	0.021589	260	67	7937	3.189380	1.000000	0.638281	29.876478
GOTERM_BP_FAT	GO:0048599 oocyte development	10	0.514139	0.021803	260	126	7937	2.422772	1.000000	0.628570	30.125393
GOTERM_CC_FAT	GO:0045263 proton-transporting ATP synthase complex, coupling factor F(o)	4	0.205656	0.022076	165	18	4786	6.445791	0.995593	0.218524	25.076629
GOTERM_BP_FAT	GO:0019941 modification-dependent protein catabolic process	12	0.616967	0.022260	260	169	7937	2.167592	1.000000	0.623386	30.654883
SP_PIR_KEYWORDS	cf(0)	3	0.154242	0.022528	452	7	12980	12.307206	0.984892	0.752788	24.549215
GOTERM_MF_FAT	GO:0031072 heat shock protein binding	6	0.308483	0.022670	295	44	7918	3.660092	0.999974	0.778404	27.844326
GOTERM_BP_FAT	GO:0043632 modification-dependent macromolecule catabolic process	12	0.616967	0.023137	260	170	7937	2.154842	1.000000	0.625299	31.659791
SMART	SM00212:UBCc	5	0.257069	0.023633	180	30	4824	4.466667	0.966498	0.816965	24.640034
INTERPRO	IPR001623:Heat shock protein DnaJ, N-terminal	6	0.308483	0.023822	413	41	10196	3.612827	1.000000	0.996831	30.559392
GOTERM_BP_FAT	GO:0006397 mRNA processing	13	0.668380	0.024846	260	194	7937	2.045619	1.000000	0.639763	33.577633
KEGG_PATHWAY	dme04120:Ubiquitin mediated proteolysis	10	0.514139	0.025136	92	97	2054	2.301658	0.765675	0.765675	22.301531
GOTERM_MF_FAT	GO:0005337 nucleoside transmembrane transporter activity	3	0.154242	0.025499	295	7	7918	11.503148	0.999993	0.773548	30.760577
GOTERM_BP_FAT	GO:0060284 regulation of cell development	10	0.514139	0.027240	260	131	7937	2.330299	1.000000	0.662349	36.180914
INTERPRO	IPR016130:Protein-tyrosine phosphatase, active site	5	0.257069	0.028055	413	29	10196	4.256492	1.000000	0.993864	34.976759
GOTERM_BP_FAT	GO:0033554 cellular response to stress	12	0.616967	0.028940	260	176	7937	2.081381	1.000000	0.673564	37.970614
GOTERM_BP_FAT	GO:0006351 transcription, DNA-dependent	10	0.514139	0.029662	260	133	7937	2.295257	1.000000	0.671794	38.716344
GOTERM_BP_FAT	GO:0016568 chromatin modification	9	0.462725	0.029712	260	112	7937	2.453056	1.000000	0.661803	38.767475
GOTERM_CC_FAT	GO:0005694 chromosome	18	0.925450	0.029943	165	304	4786	1.717464	0.999381	0.274711	32.508092
SMART	SM00355:ZnF_C2H2	20	1.028278	0.030138	180	324	4824	1.654321	0.987034	0.765074	30.369626
GOTERM_BP_FAT	GO:0045995 regulation of embryonic development	6	0.308483	0.030683	260	54	7937	3.391880	1.000000	0.663440	39.755746
INTERPRO	IPR000608:Ubiquitin-conjugating enzyme, E2	5	0.257069	0.031371	413	30	10196	4.114609	1.000000	0.989583	38.253007
SP_PIR_KEYWORDS	dna repair	5	0.257069	0.032181	452	35	12980	4.102402	0.997567	0.777907	33.261168
GOTERM_MF_FAT	GO:0015932 nucleobase, nucleoside, nucleotide and nucleic acid transmembrane transporter activity	3	0.154242	0.033176	295	8	7918	10.065254	1.000000	0.821723	38.131051
GOTERM_BP_FAT	GO:0006399 tRNA metabolic process	8	0.411311	0.033523	260	94	7937	2.598036	1.000000	0.686297	42.562815
GOTERM_BP_FAT	GO:0032774 RNA biosynthetic process	10	0.514139	0.033572	260	136	7937	2.244627	1.000000	0.677130	42.610841
GOTERM_CC_FAT	GO:0044451 nucleoplasm part	15	0.771208	0.034562	165	240	4786	1.812879	0.999806	0.299622	36.548875
SMART	SM00360:RRM	11	0.565553	0.035693	180	141	4824	2.090780	0.994264	0.724799	34.942817
GOTERM_CC_FAT	GO:0000932 cytoplasmic mRNA processing body	3	0.154242	0.035863	165	9	4786	9.668687	0.999860	0.298819	37.645241
GOTERM_MF_FAT	GO:0030554 adenyl nucleotide binding	41	2.107969	0.037281	295	812	7918	1.355256	1.000000	0.825832	41.766835
GOTERM_CC_FAT	GO:0044427 chromosomal part	15	0.771208	0.037822	165	243	4786	1.790498	0.999915	0.302567	39.264308
SP_PIR_KEYWORDS	DNA damage	5	0.257069	0.038470	452	37	12980	3.880651	0.999267	0.763934	38.429103
GOTERM_BP_FAT	GO:0007309 oocyte axis specification	9	0.462725	0.038780	260	118	7937	2.328325	1.000000	0.720791	47.437873
SP_PIR_KEYWORDS	phosphoprotein	39	2.005141	0.039095	452	815	12980	1.374179	0.999350	0.705643	38.921733
GOTERM_BP_FAT	GO:0022604 regulation of cell morphogenesis	9	0.462725	0.040453	260	119	7937	2.308759	1.000000	0.727111	48.906050
GOTERM_MF_FAT	GO:0001883 purine nucleoside binding	41	2.107969	0.040483	295	817	7918	1.346962	1.000000	0.822389	44.463279

GOTERM_BP_FAT	GO:0008360 regulation of cell shape	8	0.411311	0.040667	260	98	7937	2.491994	1.000000	0.720256	49.091608
GOTERM_BP_FAT	GO:0007314 oocyte anterior/posterior axis specification	7	0.359897	0.041462	260	78	7937	2.739596	1.000000	0.718712	49.773187
INTERPRO	IPR001412:Aminoacyl-tRNA synthetase, class I, conserved site	5	0.257069	0.042647	413	33	10196	3.740553	1.000000	0.994489	48.276351
GOTERM_BP_FAT	GO:0007308 oocyte construction	9	0.462725	0.043942	260	121	7937	2.270598	1.000000	0.731434	51.844876
GOTERM_MF_FAT	GO:0003729 mRNA binding	13	0.668380	0.043944	295	186	7918	1.875961	1.000000	0.821408	47.246865
GOTERM_BP_FAT	GO:0030036 actin cytoskeleton organization	10	0.514139	0.044050	260	143	7937	2.134750	1.000000	0.724178	51.933018
GOTERM_MF_FAT	GO:0003723 RNA binding	32	1.645244	0.044511	295	610	7918	1.408036	1.000000	0.800340	47.690475
INTERPRO	IPR012677:Nucleotide-binding, alpha-beta plait	11	0.565553	0.044754	413	134	10196	2.026598	1.000000	0.990751	49.971297
GOTERM_MF_FAT	GO:0001882 nucleoside binding	41	2.107969	0.045314	295	824	7918	1.335519	1.000000	0.782092	48.312603
GOTERM_BP_FAT	GO:0030029 actin filament-based process	10	0.514139	0.045707	260	144	7937	2.119925	1.000000	0.729609	53.270383
GOTERM_BP_FAT	GO:0048589 developmental growth	7	0.359897	0.046006	260	80	7937	2.671106	1.000000	0.724187	53.508177
GOTERM_BP_FAT	GO:0031667 response to nutrient levels	4	0.205656	0.046531	260	25	7937	4.884308	1.000000	0.720669	53.922326
GOTERM_BP_FAT	GO:0009991 response to extracellular stimulus	4	0.205656	0.046531	260	25	7937	4.884308	1.000000	0.720669	53.922326
GOTERM_BP_FAT	GO:0000956 nuclear-transcribed mRNA catabolic process	4	0.205656	0.046531	260	25	7937	4.884308	1.000000	0.720669	53.922326
GOTERM_BP_FAT	GO:0007317 regulation of pole plasm oskar mRNA localization	4	0.205656	0.046531	260	25	7937	4.884308	1.000000	0.720669	53.922326
GOTERM_CC_FAT	GO:0031965 nuclear membrane	4	0.205656	0.047181	165	24	4786	4.834343	0.999992	0.352715	46.476324
SP_PIR_KEYWORDS	ribosomal protein	11	0.565553	0.047290	452	157	12980	2.012006	0.999865	0.720120	45.058560
GOTERM_BP_FAT	GO:0046621 negative regulation of organ growth	3	0.154242	0.048043	260	11	7937	8.325524	1.000000	0.724836	55.096498
GOTERM_BP_FAT	GO:0008358 maternal determination of anterior/posterior axis, embryo	7	0.359897	0.048390	260	81	7937	2.638129	1.000000	0.720123	55.361668
GOTERM_BP_FAT	GO:0009152 purine ribonucleotide biosynthetic process	8	0.411311	0.048739	260	102	7937	2.394268	1.000000	0.715543	55.627347
GOTERM_BP_FAT	GO:0048193 Golgi vesicle transport	5	0.257069	0.050591	260	43	7937	3.549642	1.000000	0.722126	57.011304
GOTERM_BP_FAT	GO:0040008 regulation of growth	8	0.411311	0.050905	260	103	7937	2.371023	1.000000	0.717471	57.242232
INTERPRO	IPR016135:Ubiquitin-conjugating enzyme/RWD-like	5	0.257069	0.051276	413	35	10196	3.526807	1.000000	0.991004	54.896098
INTERPRO	IPR015609:Molecular chaperone, heat shock protein, Hsp40, DnaJ	5	0.257069	0.051276	413	35	10196	3.526807	1.000000	0.991004	54.896098
GOTERM_BP_FAT	GO:0006325 chromatin organization	11	0.565553	0.051284	260	170	7937	1.975271	1.000000	0.713380	57.518862
GOTERM_BP_FAT	GO:0030258 lipid modification	4	0.205656	0.051374	260	26	7937	4.696450	1.000000	0.707315	57.584077
GOTERM_BP_FAT	GO:0060281 regulation of oocyte development	4	0.205656	0.051374	260	26	7937	4.696450	1.000000	0.707315	57.584077
GOTERM_BP_FAT	GO:0006403 RNA localization	9	0.462725	0.051503	260	125	7937	2.197938	1.000000	0.701632	57.677692
GOTERM_CC_FAT	GO:0005652 nuclear lamina	3	0.154242	0.052395	165	11	4786	7.910744	0.999998	0.373156	50.143161
KEGG_PATHWAY	dme03040:Spliceosome	10	0.514139	0.053393	92	111	2054	2.011359	0.956180	0.790667	41.951718
GOTERM_BP_FAT	GO:0015992 proton transport	6	0.308483	0.054027	260	63	7937	2.907326	1.000000	0.712834	59.472633
GOTERM_BP_FAT	GO:0010627 regulation of protein kinase cascade	5	0.257069	0.054282	260	44	7937	3.468969	1.000000	0.708221	59.649925
GOTERM_BP_FAT	GO:0006818 hydrogen transport	6	0.308483	0.057116	260	64	7937	2.861899	1.000000	0.720742	61.572003
GOTERM_BP_FAT	GO:0048469 cell maturation	9	0.462725	0.057693	260	128	7937	2.146424	1.000000	0.718340	61.952773
GOTERM_BP_FAT	GO:0031047 gene silencing by RNA	5	0.257069	0.058115	260	45	7937	3.391880	1.000000	0.715016	62.228746
KEGG_PATHWAY	dme03018:RNA degradation	6	0.308483	0.058249	92	48	2054	2.790761	0.967314	0.680266	44.836637
SMART	SM00487:DEXDc	7	0.359897	0.058555	180	75	4824	2.501333	0.999810	0.819793	51.018089
INTERPRO	IPR018936: Phosphatidylinositol 3- and 4-kinase, conserved site	3	0.154242	0.059135	413	10	10196	7.406295	1.000000	0.992167	60.229037
INTERPRO	IPR000504:RNA recognition motif, RNP-1	11	0.565553	0.059393	413	141	10196	1.925987	1.000000	0.987525	60.393322
GOTERM_BP_FAT	GO:0009260 ribonucleotide biosynthetic process	8	0.411311	0.060173	260	107	7937	2.282387	1.000000	0.721920	63.548432
GOTERM_BP_FAT	GO:0009150 purine ribonucleotide metabolic process	8	0.411311	0.060173	260	107	7937	2.282387	1.000000	0.721920	63.548432
GOTERM_BP_FAT	GO:0007028 cytoplasm organization	6	0.308483	0.060305	260	65	7937	2.817870	1.000000	0.716929	63.631742
SMART	SM00146:PI3Kc	3	0.154242	0.060431	180	11	4824	7.309091	0.999857	0.771274	52.159830
GOTERM_MF_FAT	GO:0005112 Notch binding	3	0.154242	0.060576	295	11	7918	7.320185	1.000000	0.852851	58.905820
SMART	SM00490:HELICc	7	0.359897	0.061706	180	76	4824	2.468421	0.999882	0.725290	52.922350
GOTERM_BP_FAT	GO:0032268 regulation of cellular protein metabolic process	9	0.462725	0.062072	260	130	7937	2.113402	1.000000	0.721855	64.728126
GOTERM_BP_FAT	GO:0006435 threonyl-tRNA aminoacylation	2	0.102828	0.064203	260	2	7937	30.526923	1.000000	0.728645	66.008841
GOTERM_BP_FAT	GO:0051092 positive regulation of NF-kappaB transcription factor activity	2	0.102828	0.064203	260	2	7937	30.526923	1.000000	0.728645	66.008841
GOTERM_BP_FAT	GO:0016925 protein sumoylation	2	0.102828	0.064203	260	2	7937	30.526923	1.000000	0.728645	66.008841
GOTERM_BP_FAT	GO:0019950 SMT3-dependent protein catabolic process	2	0.102828	0.064203	260	2	7937	30.526923	1.000000	0.728645	66.008841
SP_PIR_KEYWORDS	Hydrogen ion transport	4	0.205656	0.065813	452	27	12980	4.254343	0.999996	0.791081	56.899416

GOTERM_BP_FAT	GO:0006418 tRNA aminoacylation for protein translation	5	0.257069	0.066204	260	47	7937	3.247545	1.000000	0.734377	67.171412
GOTERM_BP_FAT	GO:0043039 tRNA aminoacylation	5	0.257069	0.066204	260	47	7937	3.247545	1.000000	0.734377	67.171412
INTERPRO	IPR015880:Zinc finger, C2H2-like	20	1.028278	0.066687	413	324	10196	1.523929	1.000000	0.988804	64.793048
GOTERM_BP_FAT	GO:0016458 gene silencing	10	0.514139	0.066716	260	155	7937	1.969479	1.000000	0.731811	67.463390
GOTERM_BP_FAT	GO:0070647 protein modification by small protein conjugation or removal	6	0.308483	0.066981	260	67	7937	2.733754	1.000000	0.727942	67.612958
GOTERM_BP_FAT	GO:0008298 intracellular mRNA localization	6	0.308483	0.066981	260	67	7937	2.733754	1.000000	0.727942	67.612958
GOTERM_BP_FAT	GO:0045926 negative regulation of growth	4	0.205656	0.067284	260	29	7937	4.210610	1.000000	0.724346	67.783649
GOTERM_BP_FAT	GO:0050807 regulation of synapse organization	4	0.205656	0.067284	260	29	7937	4.210610	1.000000	0.724346	67.783649
GOTERM_CC_FAT	GO:0031510 SUMO activating enzyme complex	2	0.102828	0.067366	165	2	4786	29.006061	1.000000	0.442556	59.423097
GOTERM_CC_FAT	GO:0012505 endomembrane system	13	0.668380	0.069288	165	218	4786	1.729719	1.000000	0.441009	60.491338
INTERPRO	IPR000403: Phosphatidylinositol 3- and 4-kinase, catalytic	3	0.154242	0.070392	413	11	10196	6.732996	1.000000	0.987160	66.848918
GOTERM_BP_FAT	GO:0043038 amino acid activation	5	0.257069	0.070456	260	48	7937	3.179888	1.000000	0.736063	69.519915
GOTERM_BP_FAT	GO:0015931 nucleobase, nucleoside, nucleotide and nucleic acid transport	6	0.308483	0.070467	260	68	7937	2.693552	1.000000	0.731050	69.525752
UP_SEQ_FEATURE	site:Important for catalytic activity	2	0.102828	0.071299	109	2	2975	27.293578	1.000000	1.000000	62.251150
GOTERM_MF_FAT	GO:0016781 phosphotransferase activity, paired acceptors	2	0.102828	0.072887	295	2	7918	26.840678	1.000000	0.886482	65.939734
GOTERM_MF_FAT	GO:0000257 nitrilase activity	2	0.102828	0.072887	295	2	7918	26.840678	1.000000	0.886482	65.939734
GOTERM_MF_FAT	GO:0004829 threonine-tRNA ligase activity	2	0.102828	0.072887	295	2	7918	26.840678	1.000000	0.886482	65.939734
GOTERM_MF_FAT	GO:0019948 SUMO activating enzyme activity	2	0.102828	0.072887	295	2	7918	26.840678	1.000000	0.886482	65.939734
GOTERM_MF_FAT	GO:0004756 selenide, water dikinase activity	2	0.102828	0.072887	295	2	7918	26.840678	1.000000	0.886482	65.939734
GOTERM_MF_FAT	GO:0016815 hydrolase activity, acting on carbon-nitrogen (but not peptide) bonds, in nitriles	2	0.102828	0.072887	295	2	7918	26.840678	1.000000	0.886482	65.939734
GOTERM_BP_FAT	GO:0030261 chromosome condensation	4	0.205656	0.073030	260	30	7937	4.070256	1.000000	0.739107	70.863546
GOTERM_BP_FAT	GO:0009259 ribonucleotide metabolic process	8	0.411311	0.073128	260	112	7937	2.180495	1.000000	0.734685	70.913786
SP_PIR_KEYWORDS	ligase	12	0.616967	0.073846	452	193	12980	1.785501	0.999999	0.791621	61.264117
GOTERM_BP_FAT	GO:0021700 developmental maturation	9	0.462725	0.073910	260	135	7937	2.035128	1.000000	0.733721	71.310344
INTERPRO	IPR014021:Helicase, superfamily 1 and 2, ATP-binding	7	0.359897	0.074092	413	73	10196	2.367309	1.000000	0.985590	68.789935
GOTERM_BP_FAT	GO:0019094 pole plasm mRNA localization	5	0.257069	0.074846	260	49	7937	3.114992	1.000000	0.733534	71.778021
GOTERM_BP_FAT	GO:0010629 negative regulation of gene expression	14	0.719794	0.075720	260	256	7937	1.669441	1.000000	0.733062	72.208655
GOTERM_CC_FAT	GO:0043232 intracellular non-membrane-bounded organelle	41	2.107969	0.078047	165	943	4786	1.261133	1.000000	0.471113	65.039053
GOTERM_CC_FAT	GO:0043228 non-membrane-bounded organelle	41	2.107969	0.078047	165	943	4786	1.261133	1.000000	0.471113	65.039053
KEGG_PATHWAY	dme00510:N-Glycan biosynthesis	5	0.257069	0.078086	92	37	2054	3.017039	0.990287	0.686065	55.331117
INTERPRO	IPR012972:NLE	2	0.102828	0.079187	413	2	10196	24.687651	1.000000	0.985290	71.288967
INTERPRO	IPR004536:Selenide water dikinase	2	0.102828	0.079187	413	2	10196	24.687651	1.000000	0.985290	71.288967
GOTERM_BP_FAT	GO:0007316 pole plasm RNA localization	5	0.257069	0.079370	260	50	7937	3.052692	1.000000	0.745603	73.940663
SP_PIR_KEYWORDS	mrna processing	5	0.257069	0.079623	452	47	12980	3.054980	1.000000	0.782743	64.147246
GOTERM_MF_FAT	GO:0008289 lipid binding	9	0.462725	0.080520	295	121	7918	1.996414	1.000000	0.896844	69.720453
GOTERM_CC_FAT	GO:0070161 anchoring junction	5	0.257069	0.080707	165	48	4786	3.021465	1.000000	0.472191	66.321621
GOTERM_CC_FAT	GO:0034399 nuclear periphery	3	0.154242	0.081107	165	14	4786	6.215584	1.000000	0.463591	66.510536
SMART	SM00451:ZnF_U1	3	0.154242	0.081677	180	13	4824	6.184615	0.999994	0.779623	63.499796
INTERPRO	IPR014001:DEAD-like helicase, N-terminal	7	0.359897	0.082088	413	75	10196	2.304181	1.000000	0.983236	72.627087
SP_PIR_KEYWORDS	nucleus	39	2.005141	0.083554	452	869	12980	1.288787	1.000000	0.767644	65.995127
PIR_SUPERFAMILY	PIRSF036407: Selenphspht_syn	2	0.102828	0.083704	112	2	2596	23.178571	0.999995	0.999995	64.303552
GOTERM_MF_FAT	GO:0004725 protein tyrosine phosphatase activity	5	0.257069	0.084607	295	45	7918	2.982298	1.000000	0.895560	71.580194
GOTERM_BP_FAT	GO:0006417 regulation of translation	6	0.308483	0.085389	260	72	7937	2.543910	1.000000	0.767356	76.577209
INTERPRO	IPR012934:Zinc finger, AD-type	8	0.411311	0.085526	413	94	10196	2.101077	1.000000	0.981701	74.137428
GOTERM_BP_FAT	GO:0022613 ribonucleoprotein complex biogenesis	7	0.359897	0.086258	260	94	7937	2.273282	1.000000	0.766572	76.936437
INTERPRO	IPR001650:DNA/RNA helicase, C-terminal	7	0.359897	0.086259	413	76	10196	2.273863	1.000000	0.977615	74.449383
COG_ONTOLOGY	Chromatin structure and dynamics	3	0.154242	0.087864	58	11	1237	5.816614	0.855040	0.855040	50.840869
GOTERM_CC_FAT	GO:0016592 Srb-mediator complex	4	0.205656	0.088256	165	31	4786	3.742717	1.000000	0.483334	69.727954
GOTERM_BP_FAT	GO:0006793 phosphorus metabolic process	25	1.285347	0.088784	260	551	7937	1.385069	1.000000	0.772484	77.951422
GOTERM_BP_FAT	GO:0006796 phosphate metabolic process	25	1.285347	0.088784	260	551	7937	1.385069	1.000000	0.772484	77.951422

SMART	SM00397:t_SNARE	3	0.154242	0.093037	180	14	4824	5.742857	0.999999	0.785781	68.496839
GOTERM_BP_FAT	GO:0010605 negative regulation of macromolecule metabolic process	16	0.822622	0.094085	260	317	7937	1.540791	1.000000	0.788553	79.947245
INTERPRO	IPR003604:Zinc finger, U1-type	3	0.154242	0.094718	413	13	10196	5.697150	1.000000	0.980903	77.802423
GOTERM_CC_FAT	GO:0033177 proton-transporting two-sector ATPase complex, proton-transporting domain	4	0.205656	0.095045	165	32	4786	3.625758	1.000000	0.500118	72.517201
GOTERM_MF_FAT	GO:0008270 zinc ion binding	47	2.416452	0.097192	295	1026	7918	1.229544	1.000000	0.915872	76.662466
GOTERM_BP_FAT	GO:0008105 asymmetric protein localization	4	0.205656	0.098058	260	34	7937	3.591403	1.000000	0.798679	81.330401

Table 30: Architecture 30

Category	Term	Count	%	PValue	List Total	Pop Hits	Pop Total	Fold Enrichment	Bonferroni	Benjamini	FDR
GOTERM_CC_FAT	GO:0022626 cytosolic ribosome	58	1.813634	1.841886e-87	83	92	4786	36.352541	1.086713e-85	1.086713e-85	1.839275e-84
KEGG_PATHWAY	dme03010:Ribosome	58	1.813634	2.452086e-84	61	88	2054	22.192996	1.471251e-83	1.471251e-83	1.173537e-81
SP_PIR_KEYWORDS	ribosomal protein	56	1.751094	3.111998e-83	98	157	12980	47.242948	1.960559e-81	1.960559e-81	3.151068e-80
SP_PIR_KEYWORDS	ribonucleoprotein	53	1.657286	1.021302e-81	98	131	12980	53.586228	6.434205e-80	3.217102e-80	1.034124e-78
GOTERM_MF_FAT	GO:0003735 structural constituent of ribosome	60	1.876173	1.414109e-80	87	178	7918	30.678032	9.757350e-79	9.757350e-79	1.459172e-77
GOTERM_CC_FAT	GO:0044445 cytosolic part	58	1.813634	3.190120e-76	83	127	4786	26.334124	1.882171e-74	9.410853e-75	3.185597e-73
GOTERM_BP_FAT	GO:0006412 translation	71	2.220138	2.025796e-70	85	520	7937	12.749480	5.044231e-68	5.044231e-68	2.630043e-67
GOTERM_CC_FAT	GO:0033279 ribosomal subunit	59	1.844903	2.792214e-70	83	164	4786	20.744490	1.647406e-68	5.491354e-69	2.788256e-67
GOTERM_CC_FAT	GO:0005840 ribosome	60	1.876173	1.963628e-68	83	186	4786	18.600855	1.158540e-66	2.896351e-67	1.960844e-65
GOTERM_BP_FAT	GO:0000022 mitotic spindle elongation	38	1.188243	1.384367e-55	85	78	7937	45.491101	3.447074e-53	1.723537e-53	1.797291e-52
GOTERM_CC_FAT	GO:0005829 cytosol	67	2.095059	2.470912e-55	83	437	4786	8.840727	1.457838e-53	2.915676e-54	2.467409e-52
GOTERM_BP_FAT	GO:0051231 spindle elongation	38	1.188243	2.588356e-55	85	79	7937	44.915264	6.445006e-53	2.148335e-53	3.360402e-52
GOTERM_MF_FAT	GO:0005198 structural molecule activity	60	1.876173	7.031334e-54	87	456	7918	11.975197	4.851620e-52	2.425810e-52	7.255400e-51
GOTERM_CC_FAT	GO:0022625 cytosolic large ribosomal subunit	37	1.156973	7.626894e-54	83	55	4786	38.791238	4.499867e-52	7.499779e-53	7.616081e-51
GOTERM_CC_FAT	GO:0030529 ribonucleoprotein complex	62	1.938712	2.473641e-51	83	379	4786	9.432940	1.459448e-49	2.084926e-50	2.470134e-48
GOTERM_CC_FAT	GO:0015934 large ribosomal subunit	37	1.156973	8.135687e-41	83	102	4786	20.916844	4.800055e-39	6.000069e-40	8.124154e-38
GOTERM_BP_FAT	GO:0007052 mitotic spindle organization	39	1.219512	5.800126e-40	85	194	7937	18.771559	1.444231e-37	3.610579e-38	7.530168e-37
GOTERM_BP_FAT	GO:0007051 spindle organization	39	1.219512	2.379539e-37	85	225	7937	16.185255	5.925051e-35	1.185010e-35	3.089299e-34
SP_PIR_KEYWORDS	protein biosynthesis	28	0.875547	9.834445e-37	98	93	12980	39.877112	6.195701e-35	2.065234e-35	9.957913e-34
SP_PIR_KEYWORDS	ribosome	19	0.594121	4.581065e-34	98	25	12980	100.661224	2.886071e-32	7.215177e-33	4.638578e-31
GOTERM_BP_FAT	GO:0000226 microtubule cytoskeleton organization	39	1.219512	1.556274e-32	85	298	7937	12.220411	3.875122e-30	6.458537e-31	2.020474e-29
GOTERM_CC_FAT	GO:0043232 intracellular non-membrane-bounded organelle	66	2.063790	1.100676e-31	83	943	4786	4.035774	6.493990e-30	7.215544e-31	1.099116e-28
GOTERM_CC_FAT	GO:0043228 non-membrane-bounded organelle	66	2.063790	1.100676e-31	83	943	4786	4.035774	6.493990e-30	7.215544e-31	1.099116e-28
GOTERM_BP_FAT	GO:0000278 mitotic cell cycle	39	1.219512	1.768446e-29	85	358	7937	10.172297	4.403429e-27	6.290613e-28	2.295932e-26
GOTERM_BP_FAT	GO:0007017 microtubule-based process	40	1.250782	1.169485e-28	85	406	7937	9.199652	2.912018e-26	3.640023e-27	1.518315e-25
GOTERM_CC_FAT	GO:0022627 cytosolic small ribosomal subunit	22	0.687930	1.188503e-28	83	38	4786	33.383640	7.012170e-27	7.012170e-28	1.186819e-25
GOTERM_BP_FAT	GO:0000279 M phase	40	1.250782	5.912079e-26	85	478	7937	7.813931	1.472108e-23	1.635675e-24	7.675514e-23
GOTERM_BP_FAT	GO:0022403 cell cycle phase	40	1.250782	2.378379e-25	85	496	7937	7.530361	5.922164e-23	5.922164e-24	3.087794e-22
GOTERM_BP_FAT	GO:0007010 cytoskeleton organization	39	1.219512	3.073463e-25	85	465	7937	7.831575	7.652923e-23	6.957203e-24	3.990205e-22
GOTERM_BP_FAT	GO:0022402 cell cycle process	40	1.250782	9.180059e-24	85	547	7937	6.828261	2.285835e-21	1.904862e-22	1.191826e-20
GOTERM_CC_FAT	GO:0015935 small ribosomal subunit	23	0.719199	1.006834e-23	83	67	4786	19.794641	5.940320e-22	5.400291e-23	1.005407e-20
GOTERM_BP_FAT	GO:0007049 cell cycle	40	1.250782	7.245662e-22	85	616	7937	6.063407	1.804170e-19	1.387823e-20	9.406874e-19
GOTERM_CC_FAT	GO:0005811 lipid particle	29	0.906817	1.292606e-16	83	249	4786	6.715730	6.550316e-15	5.551115e-16	1.110223e-13
GOTERM_MF_FAT	GO:0019843 rRNA binding	8	0.250156	2.964704e-09	87	23	7918	31.656172	2.045646e-07	6.818819e-08	3.059179e-06
SP_PIR_KEYWORDS	cytoplasm	19	0.594121	3.048045e-09	98	436	12980	5.771859	1.920268e-07	3.840536e-08	3.086312e-06
SP_PIR_KEYWORDS	rrna-binding	6	0.187617	5.138933e-09	98	10	12980	79.469388	3.237527e-07	5.395879e-08	5.203450e-06
SP_PIR_KEYWORDS	Initiation factor	8	0.250156	5.924288e-09	98	35	12980	30.274052	3.732301e-07	5.331859e-08	5.998665e-06
SP_PIR_KEYWORDS	rna-binding	12	0.375235	5.993297e-09	98	138	12980	11.517303	3.775776e-07	4.719721e-08	6.068540e-06
GOTERM_MF_FAT	GO:0008135 translation factor activity, nucleic acid binding	11	0.343965	1.708475e-08	87	83	7918	12.061764	1.178847e-06	2.947120e-07	1.762919e-05
GOTERM_MF_FAT	GO:0032183 SUMO binding	10	0.312695	2.675132e-08	87	65	7918	14.001768	1.845840e-06	3.691682e-07	2.760380e-05
GOTERM_MF_FAT	GO:0032182 small conjugating protein binding	10	0.312695	3.070348e-08	87	66	7918	13.789620	2.118538e-06	3.530900e-07	3.168190e-05
GOTERM_MF_FAT	GO:0003743 translation initiation factor activity	9	0.281426	1.715162e-07	87	58	7918	14.122473	1.183455e-05	1.690658e-06	1.769817e-04
GOTERM_BP_FAT	GO:0006413 translational initiation	7	0.218887	2.247314e-05	85	55	7937	11.884278	0.005580	3.996254e-04	0.029172
INTERPRO	IPR012340:Nucleic acid-binding, OB-fold	6	0.187617	5.411567e-05	97	44	10196	14.333646	0.011247	0.011247	0.068296
GOTERM_CC_FAT	GO:0005852 eukaryotic translation initiation factor 3 complex	5	0.156348	1.245759e-04	83	16	4786	18.019578	0.007323	5.652584e-04	0.124330
SP_PIR_KEYWORDS	phosphoprotein	17	0.531582	3.303124e-04	98	815	12980	2.762739	0.020598	0.002310	0.333956
GOTERM_BP_FAT	GO:0022613 ribonucleoprotein complex biogenesis	7	0.218887	4.545243e-04	85	94	7937	6.953567	0.107030	0.007518	0.588494
GOTERM_BP_FAT	GO:0042254 ribosome biogenesis	6	0.187617	5.502345e-04	85	64	7937	8.754044	0.128070	0.008529	0.712006
GOTERM_BP_FAT	GO:0006414 translational elongation	4	0.125078	9.805011e-04	85	19	7937	19.658204	0.216720	0.014266	1.265510
GOTERM_MF_FAT	GO:0003723 RNA binding	16	0.500313	0.002151	87	610	7918	2.387187	0.138079	0.018402	2.197616
SP_PIR_KEYWORDS	elongation factor	3	0.093809	0.003474	98	12	12980	33.112245	0.196893	0.021688	3.462762
SMART	SM00363:S4	2	0.062539	0.014026	18	4	4824	134.000000	0.213477	0.213477	9.721943
SMART	SM00739:KOW	2	0.062539	0.017504	18	5	4824	107.200000	0.259328	0.139377	12.002166
INTERPRO	IPR005716:Ribosomal protein S7, eukaryotic/archaeal	2	0.062539	0.018743	97	2	10196	105.113402	0.980832	0.861551	21.247838
INTERPRO	IPR000754:Ribosomal protein S9	2	0.062539	0.018743	97	2	10196	105.113402	0.980832	0.861551	21.247838

GOTERM_MF_FAT	GO:0003746 translation elongation factor activity	3	0.093809	0.019515	87	20	7918	13.651724	0.743307	0.140236	18.401870
SP_PIR_KEYWORDS	protein degradation	2	0.062539	0.022254	98	3	12980	88.299320	0.757758	0.120932	20.377684
INTERPRO	IPR001975:Ribosomal protein L40e	2	0.062539	0.027984	97	3	10196	70.075601	0.997347	0.861564	30.114663
INTERPRO	IPR001813:Ribosomal protein 60S	2	0.062539	0.027984	97	3	10196	70.075601	0.997347	0.861564	30.114663
INTERPRO	IPR002906:Ribosomal protein S27a	2	0.062539	0.027984	97	3	10196	70.075601	0.997347	0.861564	30.114663
SP_PIR_KEYWORDS	polyprotein	2	0.062539	0.029562	98	4	12980	66.224490	0.849003	0.145759	26.202559
UP_SEQ_FEATURE	domain:PCI	3	0.093809	0.036011	71	13	2975	9.669556	0.982303	0.982303	33.924510
INTERPRO	IPR002942:RNA-binding S4	2	0.062539	0.037139	97	4	10196	52.556701	0.999633	0.861578	37.983890
INTERPRO	IPR000235:Ribosomal protein S7	2	0.062539	0.037139	97	4	10196	52.556701	0.999633	0.861578	37.983890
PIR_SUPERFAMILY	PIRSF002127:ribosomal protein, S9p/S9a/S16e/organellar S9 types	2	0.062539	0.045699	61	2	2596	42.557377	0.973972	0.973972	39.030362
PIR_SUPERFAMILY	PIRSF002122:ribosomal protein, S7p/S7a/S5e/organellar S7 types	2	0.062539	0.045699	61	2	2596	42.557377	0.973972	0.973972	39.030362
INTERPRO	IPR005824:KOW	2	0.062539	0.046208	97	5	10196	42.045361	0.999949	0.861591	44.967674
UP_SEQ_FEATURE	domain:S4 RNA-binding	2	0.062539	0.046513	71	2	2975	41.901408	0.994696	0.927169	41.616031
SMART	SM00088:PINT	2	0.062539	0.048286	18	14	4824	38.285714	0.568868	0.244554	30.116287
INTERPRO	IPR004038:Ribosomal protein L7Ae/L30e/S12e/Gadd45	2	0.062539	0.055193	97	6	10196	35.037801	0.999993	0.861605	51.165572
INTERPRO	IPR019956:Ubiquitin subgroup	2	0.062539	0.055193	97	6	10196	35.037801	0.999993	0.861605	51.165572
PIR_SUPERFAMILY	PIRSF002037: polyubiquitin 10	2	0.062539	0.067773	61	3	2596	28.371585	0.995805	0.935234	52.399933
PIR_SUPERFAMILY	PIRSF002046:ubiquitin/ribosomal protein S27a	2	0.062539	0.067773	61	3	2596	28.371585	0.995805	0.935234	52.399933
UP_SEQ_FEATURE	site:Essential for function	2	0.062539	0.068963	71	3	2975	27.934272	0.999614	0.927202	55.395286
UP_SEQ_FEATURE	chain:Ubiquitin	2	0.062539	0.068963	71	3	2975	27.934272	0.999614	0.927202	55.395286
UP_SEQ_FEATURE	binding site:Activating enzyme	2	0.062539	0.068963	71	3	2975	27.934272	0.999614	0.927202	55.395286
INTERPRO	IPR019954:Ubiquitin conserved site	2	0.062539	0.072912	97	8	10196	26.278351	1.000000	0.895690	61.547267
SP_PIR_KEYWORDS	isopeptide bond	2	0.062539	0.079229	98	11	12980	24.081633	0.994485	0.329694	56.647385
SMART	SM00213:UBQ	2	0.062539	0.081423	18	24	4824	22.333333	0.763971	0.302986	45.932297
GOTERM_CC_FAT	GO:0005853 eukaryotic translation elongation factor 1 complex	2	0.062539	0.082814	83	5	4786	23.065060	0.993905	0.305321	57.820165
PIR_SUPERFAMILY	PIRSF002034:ubiquitin	2	0.062539	0.089344	61	4	2596	21.278689	0.999324	0.912256	62.841381
INTERPRO	IPR006073:GTP1/OBG	2	0.062539	0.090302	97	10	10196	21.022680	1.000000	0.915630	69.723348
SP_PIR_KEYWORDS	tandem repeat	2	0.062539	0.092952	98	13	12980	20.376766	0.997859	0.355331	62.762125
