# Supplementary material for: No Promoter Left Behind (NPLB): learn de novo promoter architectures from genome-wide transcription start sites
Source: Bioinformatics. 2015 Nov 2;32(5):779–81. doi: 10.1093/bioinformatics/btv645 (PMC4795619; doi:10.1093/bioinformatics/btv645)
Supplement: Supplementary Data [file btv645_supplementary_data.zip › btv645-supplementaryInfo.pdf]

Supplementary Information for NPLB

1 Supplementary methods

1.1 Model description

Consider a dataset $\boldsymbol{X}$ consisting of n DNA sequences, $\{\boldsymbol{X}_1, \ldots, \boldsymbol{X}_n\}$, where each sequence, $\boldsymbol{X}_i$, is of length l and represented as $X_i^1 \ldots X_i^l$. We define a probabilistic model M with parameters $\boldsymbol{\theta}$ as follows.

Each sequence in $\boldsymbol{X}$ is characterized by one of k promoter architectures (PAs), $a_1, \ldots, a_k$. Each PA a_u is characterized by a few important positions, key to that PA. These are represented with l Boolean features $f_{a_u}^1 ... f_{a_u}^l$, such that if j is an important position for a_u, $f_{a_u}^j = 1$ and 0 otherwise. Nucleotides at important positions in a_u, i.e., $j \mid f_{a_u}^j = 1$, are modeled with their own categorical distribution $\boldsymbol{\phi}_{a_u}^j = [\phi_{a_u}^j(\texttt{A}), \phi_{a_u}^j(\texttt{C}), \phi_{a_u}^j(\texttt{G}), \phi_{a_u}^j(\texttt{T})]$, where $\phi_{a_u}^j(\texttt{N})$ denotes the probability of finding nucleotide N at position j in PA a_u. Nucleotides at an unimportant position j are modeled by a categorical distribution ϕ_0^j. Note that this is like a background distribution, common across all architectures for which j is an unimportant position.

Hence the model parameters $\boldsymbol{\theta}$ are:

- Boolean features $f_{a_u}^j$, where, $1 \leq u \leq k$ and $1 \leq j \leq l$
- categorical distributions $\boldsymbol{\phi}_{a_u}^j$ of nucleotides at positions with $f_{a_u}^j = 1$
- background distributions $\boldsymbol{\phi}_0^j$ of nucleotides, where for position j, $f_{a_u}^j = 0$
- vector $\boldsymbol{y}$, where y_i is a label that denotes the PA of sequence $\boldsymbol{X}_i$; $1 \leq y_i \leq k$
- categorical distribution $\boldsymbol{\gamma}$ over $\{1, \ldots, k\}$ to model $\boldsymbol{y}$.

The likelihood of a sequence $\boldsymbol{X}_i$ is:

$$\begin{aligned} P(\boldsymbol{X}_i \mid M, \boldsymbol{\theta}) &= P(\boldsymbol{X}_i \mid M, f_{a_u}, \phi_{a_u}, \phi_0, y_i = u) \\ &= \Big(\prod_{j \mid f_{a_u}^j = 1} \phi_{a_u}^j(X_i^j) \Big) \Big(\prod_{j \mid f_{a_u}^j = 0} \phi_0^j(X_i^j) \Big) \end{aligned} \tag{1}$$

and for the full set is:

$$P(\boldsymbol{X} \mid M, \theta) = \prod_{i=1}^{n} P(\boldsymbol{X}_i \mid M, \theta) \tag{2}$$

1.2 Model learning

The model is learned by optimizing the posterior distribution:

$$P(\boldsymbol{\theta} \mid \boldsymbol{X}, M) \propto P(\boldsymbol{X} \mid M, \boldsymbol{\theta}) P(\boldsymbol{\theta} \mid M) \tag{3}$$

The priors over $\boldsymbol{\theta}$ are defined as follows (all hyperparameters can be changed by the user if required). A conjugate Dirichlet prior is used over γ and all $\boldsymbol{\phi}$. The pseudocounts used for these are 1, as default. An exponential prior parameterized by non-negative λ over the number of important positions in the model, $e^{\lambda \sum_{u=1}^{k} \sum_{j=1}^{l} f_{a_u}^j}$, is used to avoid overfitting the data. A λ set to 0 is equivalent to not having any restriction on the number of positions. In that case, theoretically, highest posterior score would be achieved when all positions are considered important. Higher the value of λ, the fewer the number of important positions.

Collapsed Gibbs sampling (Liu, 1994) is used to draw samples from the posterior distribution. The labels y_i for each sequence X_i and each Boolean feature for every position j of all architectures $f^j_{a_u}$ are sampled iteratively by integrating out $\boldsymbol{\phi}$ and γ. After every sampling stage, the score of the set is computed and the one with the maximum score is chosen as the final output of $\boldsymbol{\theta}$.

1.3 Model selection

Multiple models are learned by varying the number of architectures (k) and the regularization parameter (λ). A standard 5-fold (number of folds can be changed by the user) cross-validation is performed over the resulting models to avoid overfitting. The model with the highest cross-validation likelihood is selected.

1.4 Implementation

No Promoter Left Behind (NPLB) is written in **C** and **Python**. Models are created and learned using methods written in **C**. A **Python** wrapper is used to control multiprocessing. Multiple models are learned simultaneously and **C** methods are called for computing the cross-validation likelihoods. Upon learning the best model, the model details and additional plots are saved as required by the user. The plots are created using **gnuplot**. Details of running NPLB can be found in the Documentation.

2 Case Study

2.1 Execution Details

The dataset of 6635 sequences of *Drosophila melanogaster* carcasses was created as a 90bp neighbourhood centered on the TSSs found in file *Supp_File_S1_CAGE_Dmel_FM_carcass.bed* from `http://genome.cshlp.org/content/suppl/2014/05/21/gr.159384.113.DC1.html`.

promoterLearn was run with parameters -minarch 7 and -maxarch 18, i.e. models with number of PAs between 7 and 18 were explored. On an Intel i7-3770K desktop, it took 615 minutes to report 12 as the optimal number of PAs. promoterClassify was run using the newly created model on the same dataset and the PAs were rearranged by the median values of TSS strength of each PA (Fig. S4b), found in the fifth column of the given additional file. These PAs were named as C1,...,C12.

The sequences were extracted into 12 separate files, such that, each file represented sequences for a single PA. promoterLearn was run separately on all 12 datasets. The second and fifth PAs (C2, C5) further split into two PAs each, the third, sixth, and seventh PAs (C3, C6, C7) split into three PAs each and the eighth one (C8) split in five PAs. No splits were formed in the first, fourth, tenth and twelfth PAs (C1, C4, C10, C12). The PAs resulting from the splits were named as C2_1, C2_2, C3_1, C3_2,...

The sequences representing these newly formed PAs were again extracted into individual files and passed as arguments to promoterLearn. The datasets of C7_3, C9_2 and C11_1 were further split into two PAs each and named as C7_3_1, C7_3_2, C9_2_1,... No splits were formed thereafter, resulting in a total of thirty PAs.

The PAs were then manually arranged as C2_2, C2_1, C4, C3_1, C3_3, C3_2, C5_2, C5_1, C6_1, C6_3, C6_2, C7_1, C7_2, C7_3_2, C7_3_1, C8_1, C8_2, C8_5, C8_4, C9_1, C9_2_1, C9_2_2, C8_3, C9_3, C10, C11_2, C11_1_2, C11_1_1, C12, C1 such that C2_2 is represented as A1 and C1 is represented as A30.

2.2 Discovered PAs

A1 through A6 contain a TATA-box and are arranged in decreasing order of distance of the TATA-box from the TSS. The INR motif `TCAGTY` is present at the TSS with the `A` at the +1 location in A1 and A2 but a slight variation is observed in the relatively smaller A3 to A6. In addition to a prominent `CA` at the -1,+1 location another `CA` appears either immediately (in A3), or after one nucleotide (in A4 and

A5), or after two nucleotides (in A6). Likewise, there is downstream shift of one to three nucleotides of both the TATA-box and a downstream signal. One explanation could be that the TSS was wrongly identified by one to three bases. However, three pieces of evidence suggest that this is probably not an artifact arising from such a misannotation. First, there are a significant number of promoters in each category; it seems unlikely the TSS was wrongly identified for all of them. Second, there is a `CA` signal at the identified TSS, albeit weaker. Such a `CA` just upstream of a TSS has not been observed before. Third, we have noted such a variation in other Drosophila promoter data as well (Narlikar, 2014; Ni et al., 2010). At this point we are unable to explain the significance of this variation. It could be that in different cell-types/conditions, a different `CA` is used to begin transcription. But this needs further study.

A7 through A11 contain the characteristic INR motif at the TSS. These PAs make up more than 25% of the dataset and have variations of the DPE motif `CGGTT` downstream although no significant motif is found upstream. The differences within these PAs are also interesting. The overall downstream nucleotide content in A7 and A8 is different: it is more `T`-rich, while that in A9, A10, and A11 it is more `A`-rich. The 5' UTRs for genes in A7 and A8 are also longer than the three, although in general all five PAs have longer 5' UTRs (Fig. 1).

A12 through A15 contain Dmv4 motif `GGYCACAC` in place of the INR motif at TSS. Among these PAs, A13 has a significant presence of upstream Dmv5 motif `TGGTATTT`, others seem to have a weaker Dmv5. A12 has a a `T`-rich region downstream while the others are `A`-rich. Furthermore, PA15 has an enriched `CT` present before the Dmv4 motif, while A14 has an enriched `AC`. It is possible that these PAs are targeted by proteins with slightly different sequence specificities. Curiously, both the Dmv4 and the upstream Dmv5 motif are not conserved across fly genomes (Fig. S5).

PAs A16 through A24 have variation of motif `CAGT` at TSS and are highly enriched downstream string of `A`s. A25 is different because of its string of downstream `T`s. These downstream regions possibly confer a compensatory effect for the weaker INR motif.

A26 through A29 have novel motifs at TSS, differing significantly from the canonical `CA`. These architectures clearly warrant further experimental studies.

A30 has the ribosomal TCT motif at TSS. The GO term analysis confirms this (Table S1). Promoters with this architecture have the highest number of tags among all the discovered PAs, which can be explained by the housekeeping property and abundance of ribosomal genes.

Unsurprisingly, PAs A16 through A30 are enriched in the sequences left out in the original analysis of Chen et al. since they contain novel PEs. Even some PAs with the TATA-box were missed since they have a variation of the INR motif. However, these are exactly the PAs which are likely to yield new regulatory mechanisms and must not be left out of the analysis.

Supplementary Figures

Figure S1: Execution time versus dataset size

promoterLearn was applied to 100bp-long simulated promoter sequences with 10 PAs but of varying sizes. The run time shown is for a single model. It scales linearly with the size of the dataset. The total time taken to find the best model depends on the number of models learned, which in turn depends on the structure of the data. When promoterLearn explores the model space, it serially tries different values of the regularization parameter λ which dictates the number of important features. Generally speaking, on bacterial promoters which are typically around 1000 in number, it takes two hours, while on the fly dataset mentioned in the main text it takes about 10 hours to find the best model. All times reported are on a standard Intel i7 desktop, which has four hyperthreading cores. We expect the time to reduce linearly on machines with more cores.

Figure S2: Sequence logos for all 30 architectures

Sequence logos were constructed using a modified version of Weblogo 3.3. The positions with important features are represented by blue coloured numbers or blue asterisks. The relevant positions are all relevant positions found in the current execution of promoterLearn on the given dataset as well as the execution on the dataset which formed its superset.

Figure S3: New architectures in the four modENCODE Types and the left out promoters

(a) Four Types as categorised by Chen et al.: 1. with a combination of TATA-box (`TATAAA`), INR (`TCAGTY`), and DPE (`CGGTT`); 2. with an INR, DPE as well as a slightly higher CG content; 3. with Dmv4 (`GGYCACAC`) in the place of INR; and 4. with a Dmv5 (`TGGTATTT`) in place of TATA-box and a Dmv4 motif in place of an INR. The sequences left out from the analysis are displayed below. (b) Promoters in each Type as well as the left out category have been reordered based on the 30 NPLB-identified PAs, keeping the order the same: A1 to A30.

Figure S4: Multilevels of PA

(a) The input 6635 promoter sequences ordered according to chromosome location in adult *D. melanogaster* carcasses (Chen et al., 2014). (b) The 12 PAs discovered on running promoterLearn and then promoterClassify on the input. The dataset was then divided into 12 sets, one set for each PA. (c) The output of promoterLearn run separately on all 12 datasets, resulting in a total of 27 PAs. (d) Sequences in C2_1, C2_2, C3_1, C3_2, C3_3, C5_1, C5_2, C5_3, C6_1, C6_2, C6_3, C7_1, C7_2, C7_3, C8_1, C8_2, C8_3, C8_4, C8_5, C9_1, C9_2, C9_3, C11_1 and C11_2 were extracted and promoterLearn was executed individually on each of the datasets, to get a total of 30 PAs.

Figure S5: Conservation Plots

(a) The input 6635 promoter sequences ordered according to chromosome location as in Figure S4 and (b) sequence conservation corresponding to them. (c) 30 PAs discussed in the main text and (d) the conservation plot, reordered accordingly. The conservation data are in fact from phyloCons values constructed from 14 related genomes (Karolchik et al., 2014).

Supplementary Tables

Table S1: GO term analysis of all PAs

GO term analysis is performed individually on datasets representing each of the PAs using DAVID (Huang et al., 2007).

References

Chen, Z. et al. (2014) Comparative validation of D.melanogaster modENCODE transcriptome annotation. *Genome Res.*, **24**, 1209–1233.

Huang, D.W. et al. (2007) DAVID Bioinformatics Resources: expanded annotation database and novel algorithms to better extract biology from large gene lists. *Nucleic Acids Res.*, **35**, W169–175.

Karolchik, D. et al. (2014) The UCSC Genome Browser database: 2014 update. *Nucleic Acids Res.*, **42**, D764–770.

Liu, J. (1994) The collapsed gibbs sampler with applications to a gene regulation problem. *J. Am. Stat. Assoc.*, **89**, 958–966.

Narlikar, L. (2014) Multiple novel promoter-architectures revealed by decoding the hidden heterogeneity within the genome. *Nucleic Acids Res.*, **42**, 12388–12403.

Ni, T. et al. (2010) A paired-end sequencing strategy to map the complex landscape of transcription initiation. *Nat. Methods*, **7**, 521–527.
