# Supplementary material for: No Promoter Left Behind (NPLB): learn de novo promoter architectures from genome-wide transcription start sites
Source: Bioinformatics. 2015 Nov 2;32(5):779–81. doi: 10.1093/bioinformatics/btv645 (PMC4795619; doi:10.1093/bioinformatics/btv645)
Supplement: Supplementary Data [file btv645_supplementary_data.zip › FigureS2.pdf]

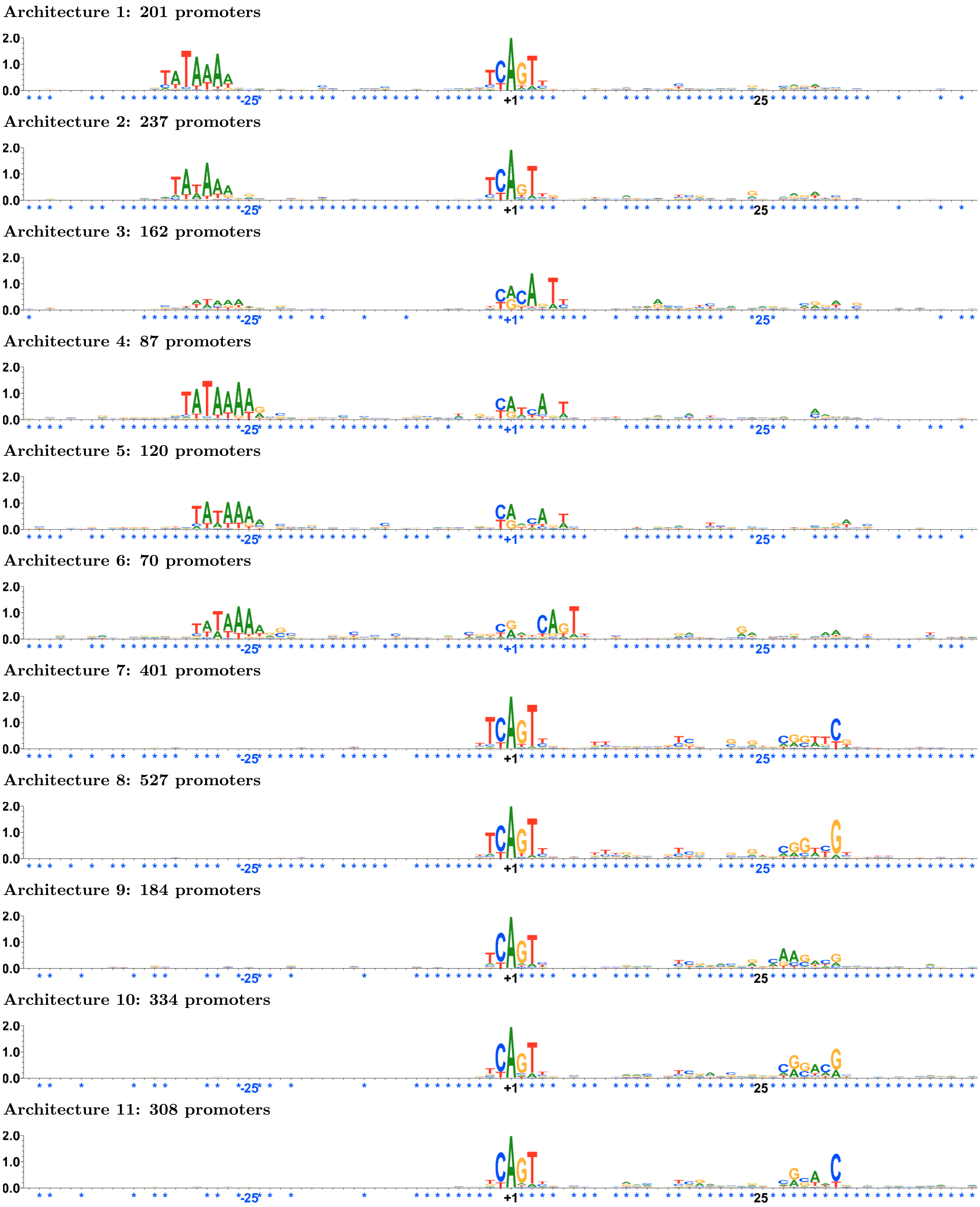
Architecture 1: 201 promoters
Architecture 2: 237 promoters
Architecture 3: 162 promoters
Architecture 4: 87 promoters
Architecture 5: 120 promoters
Architecture 6: 70 promoters
Architecture 7: 401 promoters
Architecture 8: 527 promoters
Architecture 9: 184 promoters
Architecture 10: 334 promoters
Architecture 11: 308 promoters
2.0
1.0
0.0
-25
+1
25

Architecture 12: 135 promoters

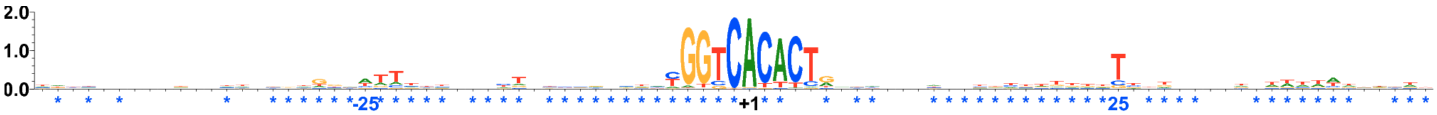

Architecture 13: 91 promoters

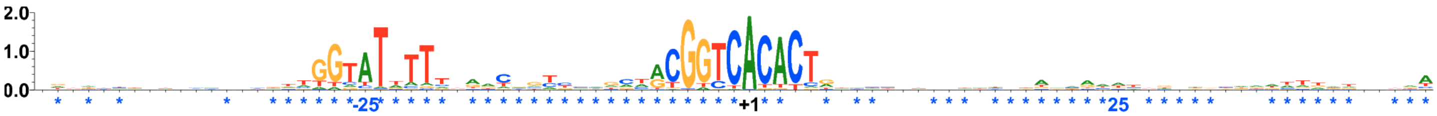

Architecture 14: 70 promoters

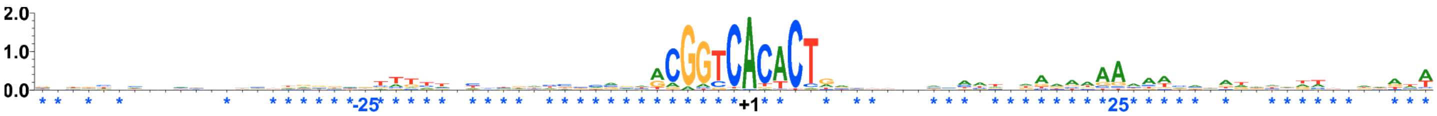

Architecture 15: 56 promoters

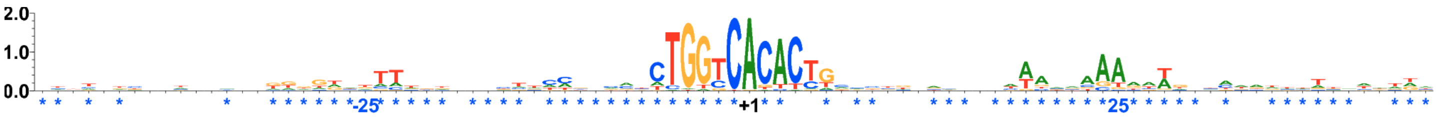

Architecture 16: 98 promoters

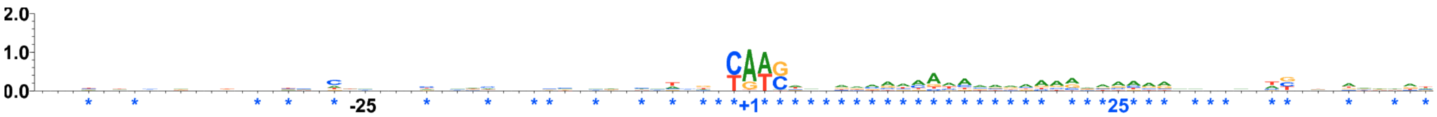

Architecture 17: 153 promoters

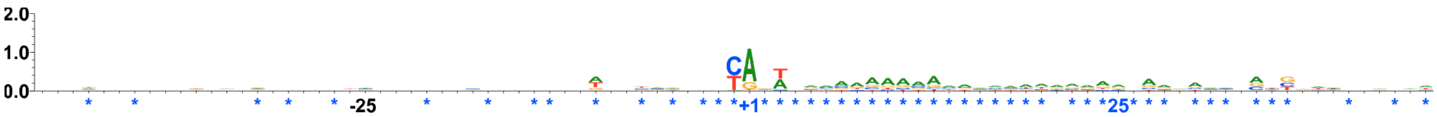

Architecture 18: 104 promoters

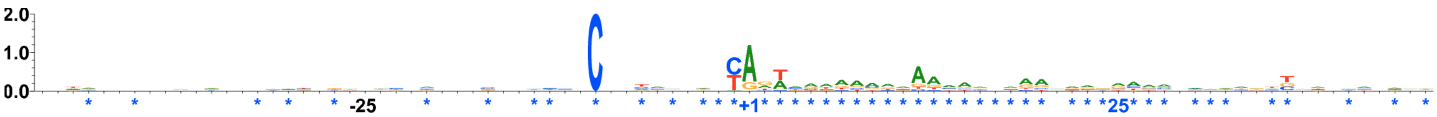

Architecture 19: 61 promoters

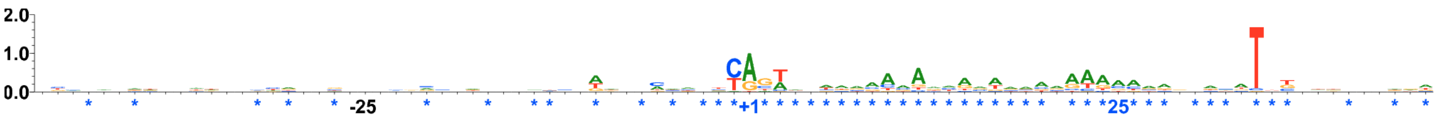

Architecture 20: 303 promoters

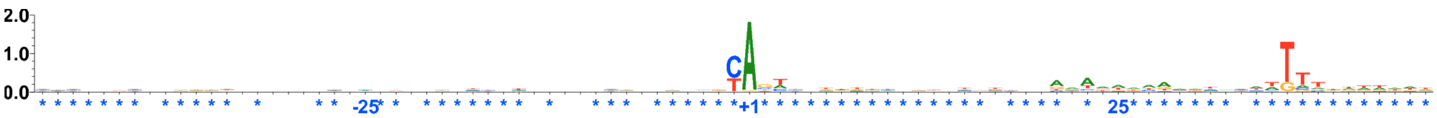

Architecture 21: 228 promoters

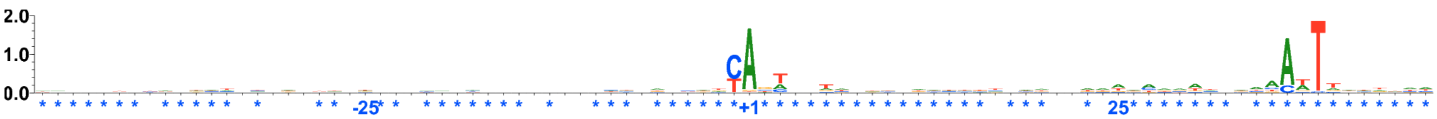

Architecture 22: 139 promoters

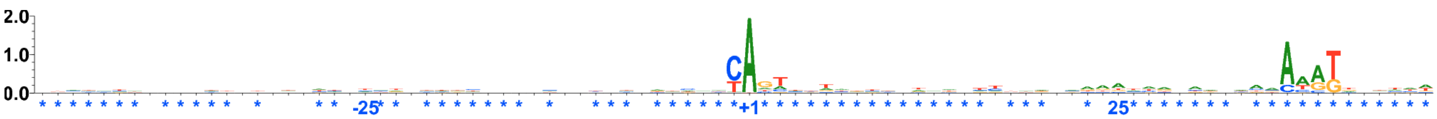

Architecture 23: 187 promoters

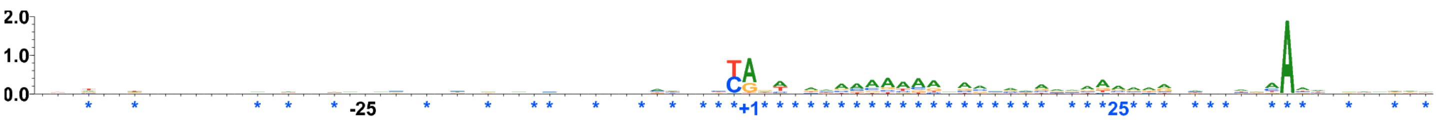

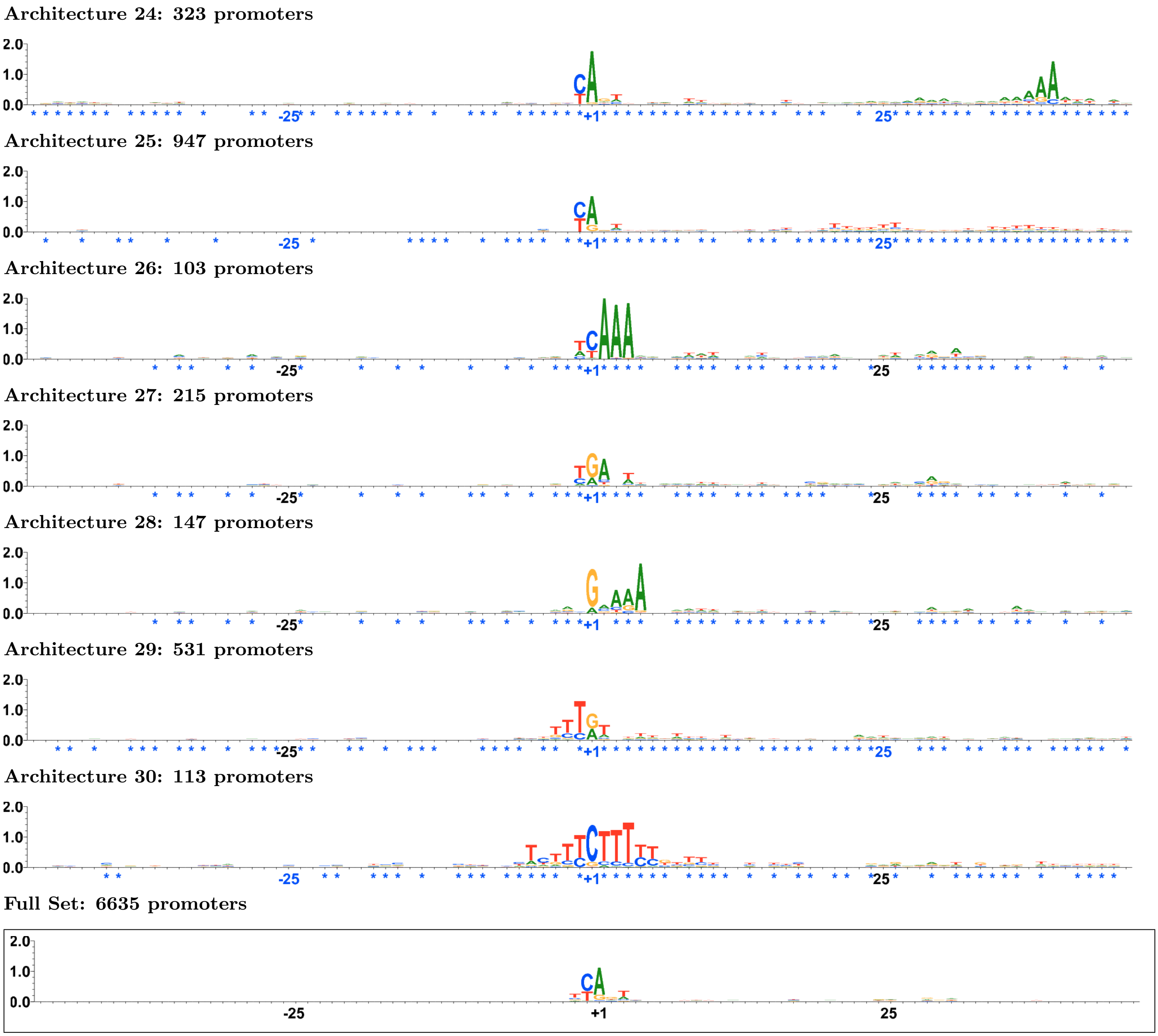

Architecture 24: 323 promoters
Architecture 25: 947 promoters
Architecture 26: 103 promoters
Architecture 27: 215 promoters
Architecture 28: 147 promoters
Architecture 29: 531 promoters
Architecture 30: 113 promoters
Full Set: 6635 promoters
2.0
1.0
0.0
-25
+1
25

Figure S2
