# Supplementary figures and images for: No Promoter Left Behind (NPLB): learn de novo promoter architectures from genome-wide transcription start sites
Source: Bioinformatics. 2015 Nov 2;32(5):779–81. doi: 10.1093/bioinformatics/btv645 (PMC4795619; doi:10.1093/bioinformatics/btv645)

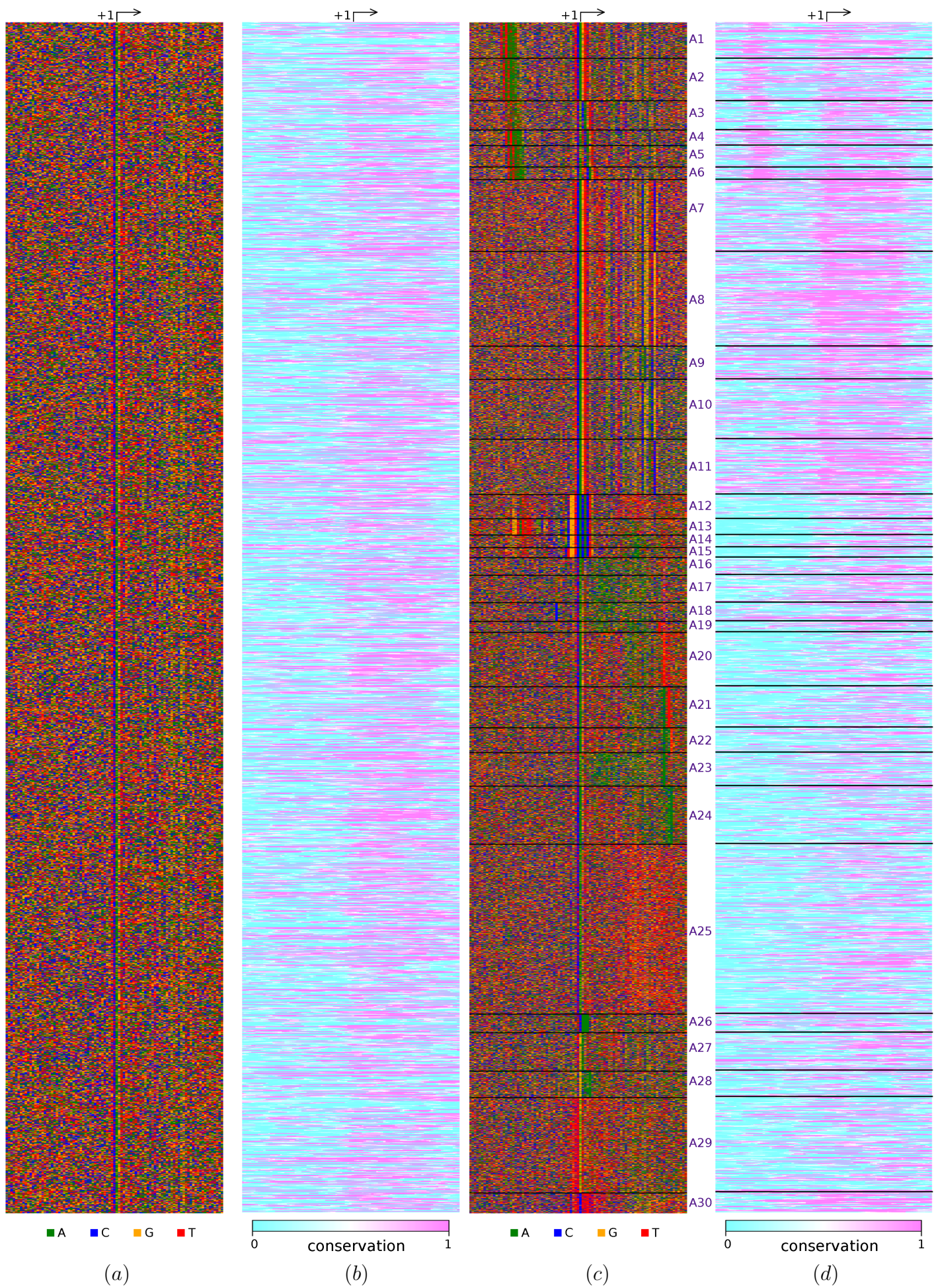

Figure S5

Supplement: Supplementary Data [file btv645_supplementary_data.zip › FigureS5.pdf]

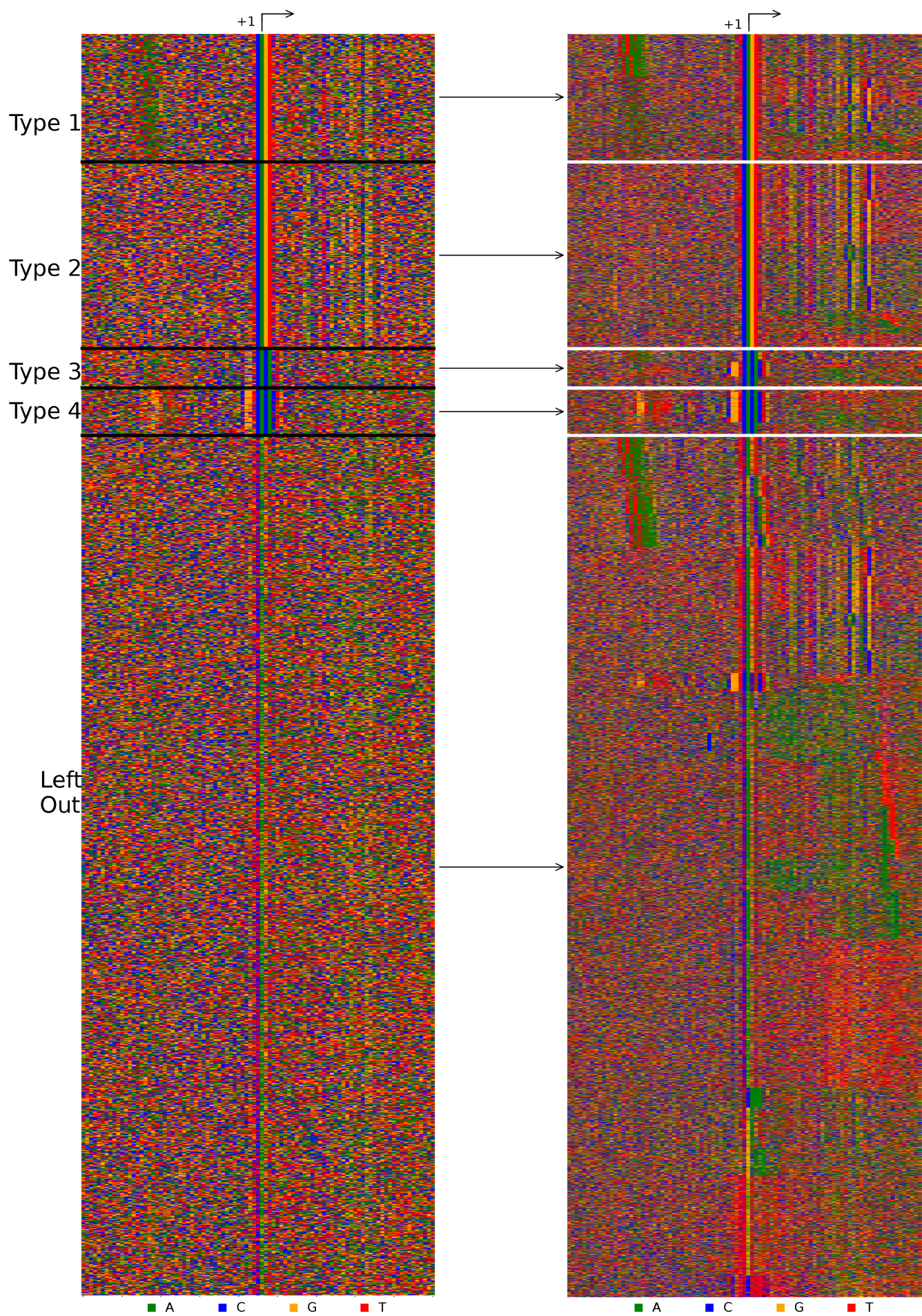

Figure S3

Supplement: Supplementary Data [file btv645_supplementary_data.zip › FigureS3.pdf]

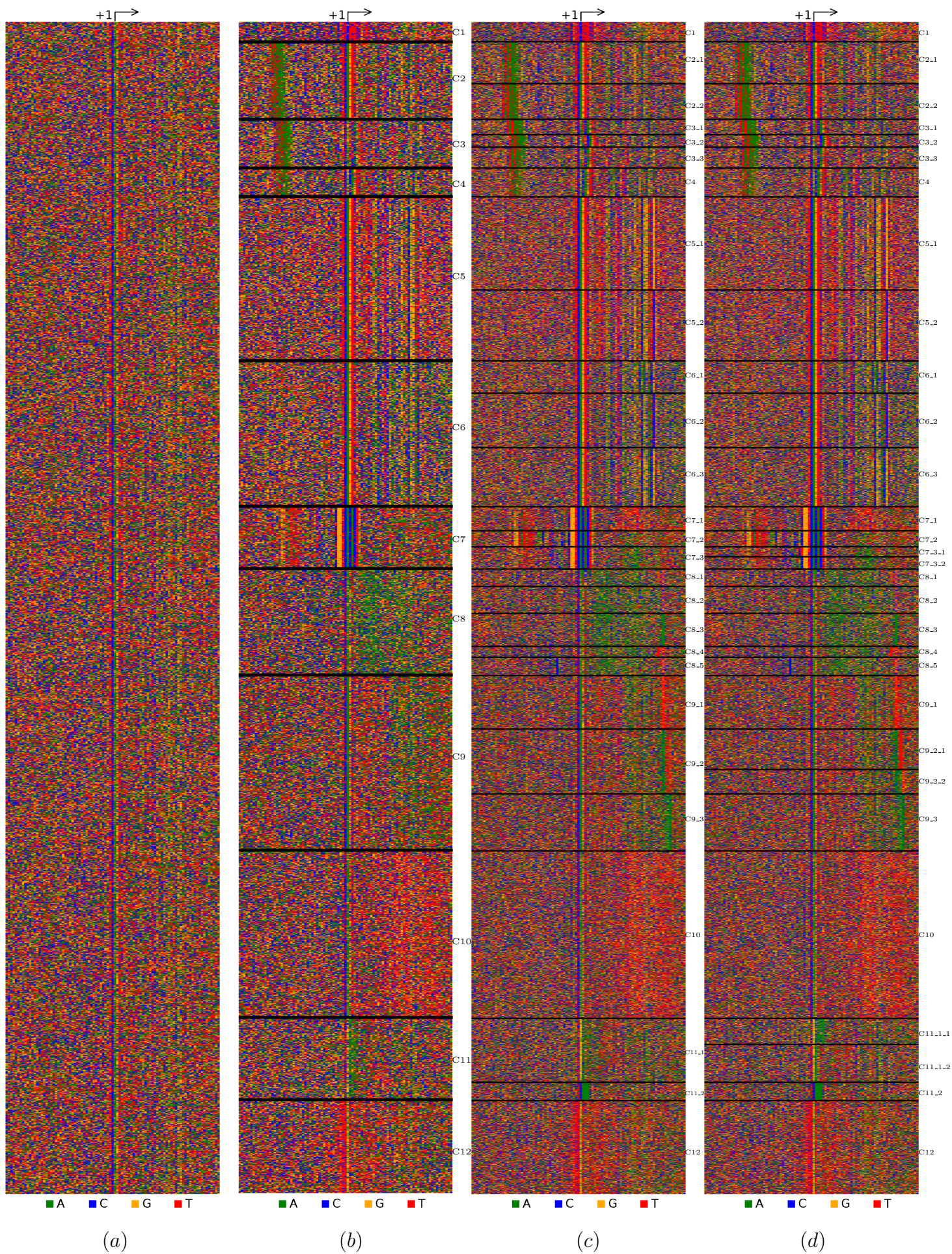

Figure S4

Supplement: Supplementary Data [file btv645_supplementary_data.zip › FigureS4.pdf]

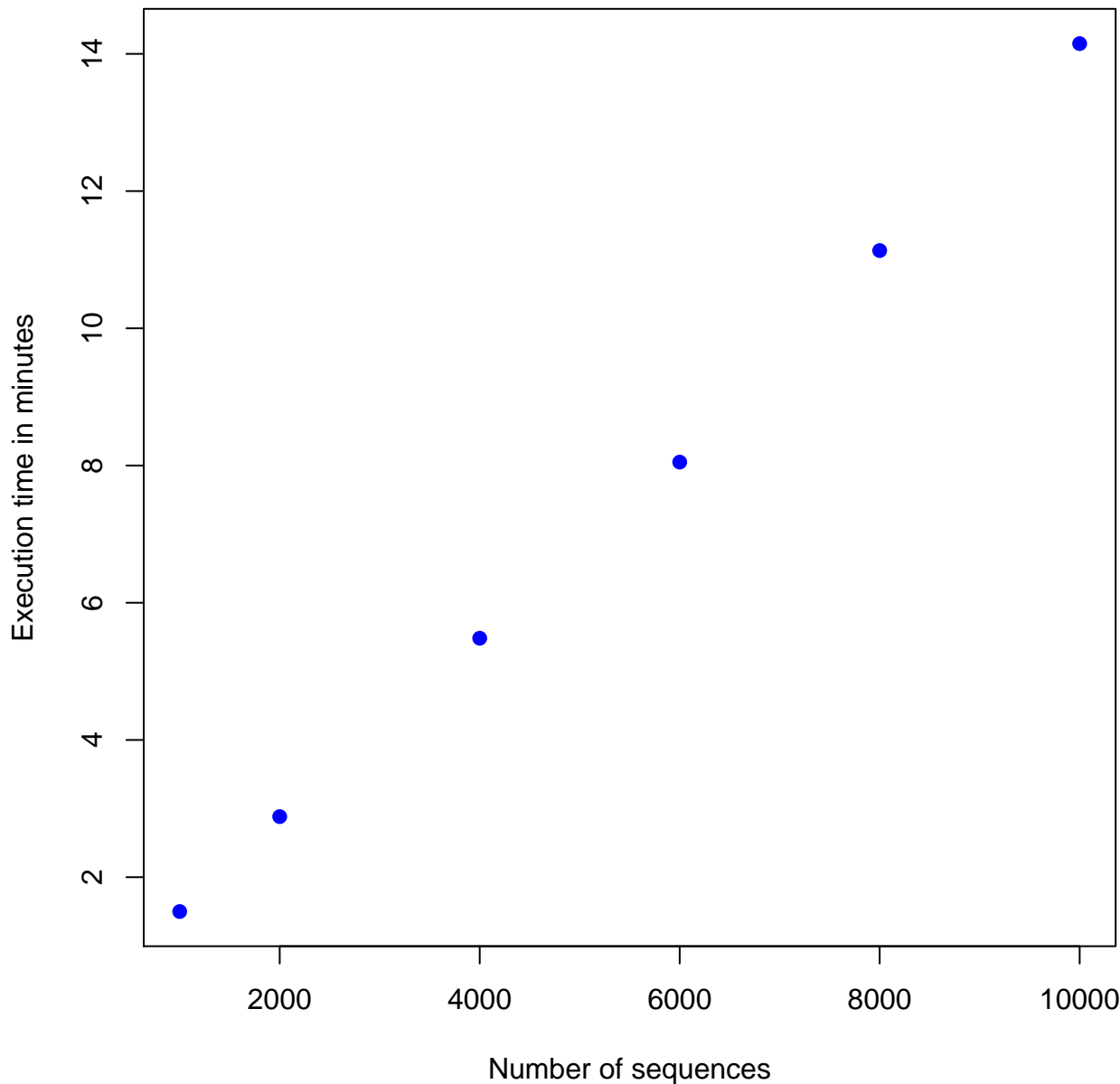

**Figure S1**

Supplement: Supplementary Data [file btv645_supplementary_data.zip › FigureS1.pdf]
